# Analysis of transcribed human endogenous retrovirus W *env *loci clarifies the origin of multiple sclerosis-associated retrovirus *env *sequences

**DOI:** 10.1186/1742-4690-6-37

**Published:** 2009-04-15

**Authors:** Georg Laufer, Jens Mayer, Benedikt F Mueller, Nikolaus Mueller-Lantzsch, Klemens Ruprecht

**Affiliations:** 1 Institute of Virology, Saarland University Hospital, Homburg, Germany; 2 Department of Human Genetics, Saarland University Hospital, Homburg, Germany; 3 Department of Neurology, Saarland University Hospital, Homburg, Germany

## Abstract

**Background:**

Multiple sclerosis-associated retrovirus (MSRV) RNA sequences have been detected in patients with multiple sclerosis (MS) and are related to the multi-copy human endogenous retrovirus family type W (HERV-W). Only one HERV-W locus (ERVWE1) codes for a complete HERV-W Env protein (Syncytin-1). Syncytin-1 and the putative MSRV Env protein have been involved in the pathogenesis of MS. The origin of MSRV and its precise relation to HERV-W were hitherto unknown.

**Results:**

By mapping HERV-W *env *cDNA sequences (n = 332) from peripheral blood mononuclear cells of patients with MS and healthy controls onto individual genomic HERV-W *env *elements, we identified seven transcribed HERV-W *env *loci in these cells, including ERVWE1. Transcriptional activity of individual HERV-W *env *elements did not significantly differ between patients with MS and controls. Remarkably, almost 30% of HERV-W *env *cDNAs were recombined sequences that most likely arose *in vitro *between transcripts from different HERV-W *env *elements. Re-analysis of published MSRV *env *sequences revealed that all of them can be explained as originating from genomic HERV-W *env *loci or recombinations among them. In particular, a MSRV *env *clone previously used for the generation of monoclonal antibody 6A2B2, detecting an antigen in MS brain lesions, appears to be derived from a HERV-W *env *locus on chromosome Xq22.3. This locus harbors a long open reading frame for an N-terminally truncated HERV-W Env protein.

**Conclusion:**

Our data clarify the origin of MSRV *env *sequences, have important implications for the status of MSRV, and open the possibility that a protein encoded by a HERV-W *env *element on chromosome Xq22.3 may be expressed in MS brain lesions.

## Background

Multiple sclerosis (MS) is a chronic inflammatory demyelinating disease of the central nervous system thought to result from an as yet incompletely understood complex interplay of genetic and environmental factors [1]. Stimulated by the finding that human T cell leukemia virus type 1 (HTLV-1), a human exogenous retrovirus, causes a neurological disease (HTLV-1 associated myelopathy/tropical spastic paraparesis) with similarities to MS [2], retroviruses have also been searched for in MS. This led to the detection of retrovirus-like particles containing reverse transcriptase activity in the supernatants from cultured cell of patients with MS [3-5]. After molecular characterization of retroviral RNA sequences within such particles, this novel retroviral element was named MS-associated retrovirus (MSRV) [6,7]. Subsequent probing of human genomic DNA with MSRV sequences revealed endogenous retroviral sequences closely related to MSRV, the human endogenous retrovirus (HERV) family type W (HERV-W) [8]. HERVs are remnants of ancestral germ line infections by active retroviruses, which have thereafter been transmitted in a Mendelian manner. In humans, HERVs comprise approximately 8% of the genome (for review see [9,10]). They typically consist of an internal region containing *gag*, *pro*, *pol*, and *env *genes, flanked by two long terminal repeats (LTR). HERV-W is a multicopy family consisting of ~650 elements dispersed in the human genome [11]. About 280 of those elements contain internal sequences. The remaining elements lack internal regions because of recombinational deletion between the two LTRs, leaving a solo LTR [11]. Like almost all HERV families, HERV-W is highly defective due to acquisition of stop-codons, frameshift mutations, and deletions. In addition, many members of the HERV-W family represent processed pseudogenes that were generated through retrotransposition by long interspersed elements (LINE) [11-13]. Notably, no replication-competent HERV-W provirus could be identified so far [8,14]. Yet, a single HERV-W *env *locus (ERVWE1, chromosomal location 7q21.2) retained a complete open reading frame (ORF) for a functional envelope (Env) protein, termed Syncytin-1 [15]. Syncytin-1 is highly expressed in the placenta where it likely participates in the fusion of cytotrophoblast cells into the syncytiotrophoblast layer [16]. The ERVWE1 locus therefore appears to have been diverted into a *bona fide *human gene [17].

Intriguingly, in addition to the physiological function of Syncytin-1 in placental morphogenesis, several studies have provided evidence in support of a possible pathogenic role of HERV-W Env/Syncytin-1 in MS. HERV-W *env*/Syncytin-1 RNA levels were found to be higher in autopsied brain tissue from patients with MS than in brain tissue from controls [18-21]. Neuropathological investigations reported increased expression of HERV-W Env/Syncytin-1 protein in astrocytes and microglia in actively demyelinating brain lesions from patients with MS [18,21,22]. A potential pathogenic significance of HERV-W Env/Syncytin-1 expression in MS can be inferred from data showing that Syncytin-1 has indirect cytotoxic effects on oligodendrocytes *in vitro*, and that expression of Syncytin-1 in murine models results in demyelination *in vivo *[18,22].

A putative MSRV Env protein was previously reported [7,23]. The surface (SU) domain of MSRV Env (AF331500), the amino acid sequence of which is 87% identical to Syncytin-1, has been shown to stimulate production of proinflammatory cytokines in human monocytes via engagement of CD14 and toll-like receptor 4. It also triggers maturation of human dendritic cells and confers on them the potential to polarize naive T-cells into Th-1-like effector T-lymphocytes [24]. Production of IFN-γ, IL-6, and IL-12p40 following stimulation with MSRV Env SU of peripheral blood mononuclear cells (PBMC) from patients with MS is significantly higher compared to PBMC from healthy controls [25]. In addition, MSRV Env induces a polyclonal activation of T-cells bearing specific Vβ chains, reminiscent of immunopathogenic effects triggered by superantigens [23]. Altogether, those proinflammatory properties of MSRV Env appear compatible with a potential relevance of MSRV Env in the context of MS, too.

Despite the possible role of MSRV and HERV-W (Syncytin-1) in MS, the exact origin of MSRV and the precise relationship between MSRV and HERV-W have been hitherto unclear. MSRV has been defined by different overlapping cDNA clones that were generated from particle-associated RNA from plasma or supernatants of cultured cells from patients with MS [6,7,23]. Sequences of those cloned cDNAs were reported to be similar to HERV-W, but individual HERV-W proviruses from which those sequences may have originated could not be identified so far. To date, there exists no molecular clone containing a complete infectious MSRV genome, and the very nature of MSRV has thus remained uncertain [26,27]. This has further stimulated a discussion over the relative contributions of MSRV and HERV-W to the pathogenesis of MS [20,28-30].

By analogy to some well-characterized animal retroviruses (e.g. Jaagsiekte sheep retrovirus), it has been speculated that MSRV could be an exogenous member of an endogenous retrovirus family which consequently might be able to form novel proviral insertions in human genomic DNA [6,30-32]. If this was the case, it may be assumed that RNA transcripts from such proviral MSRV insertions are specifically detectable in individuals infected with MSRV. However, complicating the identification of such MSRV RNA transcripts, many of the HERV-W elements present in human genomic DNA, which all are very similar to MSRV, may produce RNA transcripts as well. We therefore asked which HERV-W loci are transcriptionally active in human cells, and whether transcripts corresponding to previously published MSRV sequences might be specifically detectable in patients with MS. Given the potential pathogenic role of MSRV/HERV-W Env proteins in MS, we focused our analysis on MSRV/HERV-W *env *sequences. Published MSRV *env *sequences were obtained from plasma of patients with MS [7,23]. Since MSRV sequences found in plasma are likely to originate from PBMC and since MSRV/HERV-W *env *sequences have previously been detected in human PBMC [18-21] we chose to analyze PBMC in this work.

Herein, we identify transcriptionally active HERV-W *env *loci in PBMC from patients with MS and healthy controls. We also demonstrate that analysis of transcribed HERV-W *env *elements is complicated by frequent recombinations that are most likely generated *in vitro*. Based on these results we show that the published MSRV *env *and *gag *sequences can be explained as originating from endogenous HERV-W loci or recombinations among them. By clarifying the origin of MSRV sequences, our data help to settle a longstanding debate, and have important implications for the status of MSRV as well as for the potential role of MSRV/HERV-W Env in the pathogenesis of MS.

## Results

### Transcriptionally active HERV-W env loci in human PBMC

**Figure 1:**
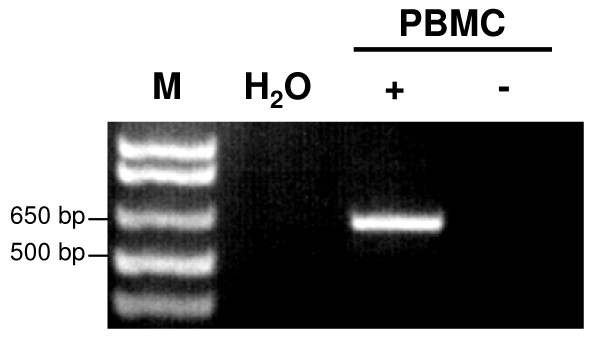
**Expression of HERV-W *env *in human PBMC**. RT-PCR using HERV-W *env*-specific primers was carried out on total RNA isolated from human PBMC which was subjected (+) or not (-) to reverse transcription. The expected size of the amplified HERV-W *env *fragment is ~640 bp. M, DNA size marker; H_2_O, PCR negative control.

**Figure 2:**
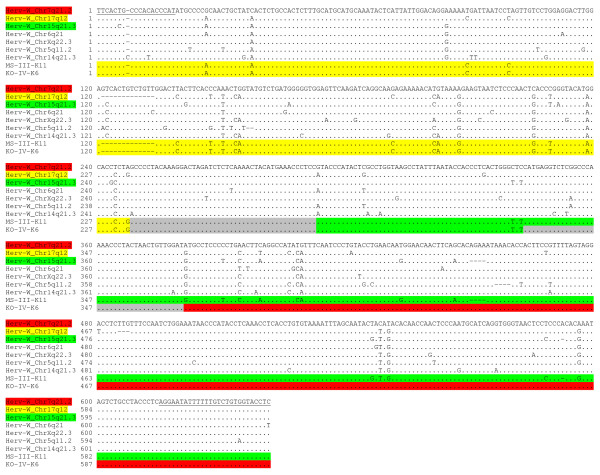
**Examples of recombined HERV-W *env *cDNA sequences**. A multiple alignment of the genomic DNA sequences (March 2006 human genome assembly) of the seven HERV-W *env *loci identified as transcriptionally active in human PBMC in this study is shown. HERV-W *env *loci are designated according to their chromosomal location. The 7q21.2 HERV-W *env *locus (ERVWE1) serves as reference sequence. Note that the 7 HERV-W *env *loci can be distiguished by unique nucleotides and/or indels. Two of the cloned HERV-W *env *cDNA sequences, MS-III-K11 (from a patient with MS) and KO-IV-K6 (from a healthy control) are shown as examples of recombined cDNAs. The proviral origin of cDNA sequence portions is indicated by a color code. Gray shaded areas represent regions in which recombination events have taken place. Sequences of the primers used in this study are underlined.

We previously described an experimental strategy to identify distinct transcriptionally active HERV loci in human tissues and cells, which we applied to detect transcribed proviral loci for the HERV-K(HML-2) family [33-35]. Using this strategy, we aimed at identifying transcribed HERV-W *env *loci and/or MSRV *env *sequences in PBMC from patients with MS and healthy controls. To this end, we performed RT-PCR on total RNA isolated from PBMC of 4 patients with MS and 4 healthy controls. An extensive DNAse digestion protocol assured the absence of contaminating genomic DNA in all samples studied (Figure 1). We employed a pair of HERV-W *env*-specific PCR primers located in the region of HERV-W *env *coding for the SU domain and generating a PCR product of about 640 bp. The HERV-W *env*-specific PCR primers were designed to amplify the previously reported MSRV *env *sequence AF331500 and the HERV-W *env *locus on chromosome 7q21.2 (ERVWE1). In addition, they potentially recognize at least eight other HERV-W *env *loci in the human genome, as determined by BLAT PCR analyses . However, according to more detailed comparisons with HERV-W *env *sequences retrieved from the human genome sequence, the actual number of HERV-W *env *loci possibly amplified by the HERV-W *env *primers is probably even higher, since further loci with few mismatches to the primers are very likely to be amplified as well. PCR-products were subsequently cloned, and individual cDNA clones were sequenced. HERV-W *env *cDNA sequences were assigned to specific HERV-W *env *loci in the human genome, based on characteristic nucleotide differences between HERV-W *env *loci (see also Figure 2).

From each individual, we generated a median of 42 (range 40–44) HERV-W *env *cDNA clones, resulting in a total of 332 cDNA clones, the sequences of which are provided in additional file 1. To map HERV-W *env *cDNA sequences onto individual genomic HERV-W *env *loci, all 332 sequences were analyzed using human BLAT searches at the UCSC Human Genome Browser [36]. We thereby identified, in total, 7 transcribed HERV-W *env *loci in human PBMC. A list of those HERV-W *env *loci and their main characteristics are provided in Table 1[37]. In particular, the previously well characterized HERV-W *env *locus on chromosome 7q21.2 (ERVWE1), that is, the gene encoding Syncytin-1, was found to be transcribed in human PBMC. The 7q21.2 locus contains a full-length HERV-W proviral copy, flanked by two complete HERV-W LTRs. As for the structure of the other 6 transcriptionally active HERV-W *env *loci, all of them display incomplete 3'LTRs ending just downstream from the poly-A signal, the expected 3' end of the LTR R-region. In addition, two of those 6 elements (located on chromosome 6q21, and 15q21.3) show a deletion of the 5' LTR's first 255 nucleotides, corresponding to the expected LTR U3 region. The four remaining elements (5q11.2, 14q21.3, 17q12, and Xq22.3) are severely truncated at the 5' end, lacking the 5'LTR, the *gag *region, and varying portions of the 5' *pol *region. Structures of transcribed HERV-W *env *loci are provided in additional file 2. In summary, except for the 7q21.2 locus, all HERV-W *env *loci found to be transcriptionally active in human PBMC show characteristic features of HERV-W pseudogenes that have been generated by LINE machinery [11]. In keeping with results obtained by others [38,39], our data therefore indicate that despite having truncated or completely missing 5'LTRs HERV-W pseudogenes can be transcribed. This implies that as yet unidentified promotors located upstream of those HERV-W pseudogenes drive their transcription.

**Table 1:** Characteristics of HERV-W *env *loci identified in this study as transcribed in human PBMC

HERV-W *env *locus	strand	location of amplicon in genome		5' LTR	3' LTR	processed pseudogene	full-length Env ORF
5q11.2	-	56852791	56853425	absent	Δ325–780	+	-

6q21	+	106788519	106789159	Δ1–255	Δ327–780	+	-

7q21.2 (ERVWE1)	-	91936808	91937448	complete	complete	-	+ (538 aa)

14q21.3	-	44559628	44559996	absent	Δ327–780	+	-

15q21.3	-	53385554	53386189	Δ1–255	Δ327–780	+	-

17q12	+	32765922	32766546	absent	Δ327–780	+	-

Xq22.3	-	106183197	106183837	absent	Δ327–780	+	-*

The chromosomal location of transcriptionally active HERV-W *env *loci is indicated in the first column. Nucleotide positions of amplicons on the respective chromosomes (human genome sequence March 2006 assembly) are indicated. Structures of the 5' and 3' LTRs of transcribed HERV-W *env *loci are given with respect to the 780-bp HERV-W LTR consensus sequence (LTR17) obtained from Repbase [37]. Missing nucleotides (Δ) in the LTRs as compared to the consensus LTR sequence are indicated. All but one locus represent processed pseudogenes. Presence of full length ORFs for HERV-W Env proteins is given in the final column.

* The Xq22.3 locus contains an ORF for a hypothetical N-terminally truncated 475 amino acid HERV-W Env protein.

In accordance with previous analyses of the coding capacity of the HERV-W family [14,15,40], except for the 7q21.2 HERV-W *env *locus, none of the transcribed HERV-W *env *loci disclosed ORFs for full-length Env proteins. Still, a transcriptionally active HERV-W *env *locus on chromosome Xq22.3 contains an almost complete *env *ORF, only interrupted by a single premature stop codon in its 5' region (codon 39) followed by several in-frame ATGs. If the longest possible *env *ORF from this transcribed locus were translated, starting at an in-frame ATG at codon 68, the Xq22.3 HERV-W *env *locus could give rise to an N-terminally truncated 475 amino acid HERV-W Env protein.

### A close inspection of HERV-W env cDNAs reveals a high number of recombined sequences

Ideally, a HERV-W *env *cDNA sequence is expected to display no nucleotide mismatches to the genomic HERV-W *env *locus that it originated from. About one third of HERV-W *env *cDNAs analyzed in this work indeed perfectly matched with genomic DNA sequences. However, the remaining two thirds of HERV-W *env *cDNAs contained between 1 and 24 nucleotide differences compared to the best matching genomic HERV-W *env *locus. Although minor nucleotide differences may well be explained by the inaccuracy of Taq polymerase, sequencing errors, or sequence variations (SNPs) in genomic HERV-W *env *loci, those possibilities seem unlikely to account for the relatively high numbers of nucleotide mismatches observed in some of the cDNA sequences. It has recently been shown that analyses of transcribed HERV sequences are complicated by recombinations between individual HERV transcripts, which most likely arise *in vitro *during reverse transcription because of template switches of reverse transcriptase and/or through PCR-mediated recombinations [41]. To investigate whether similar recombinations also occurred in the present study, we generated multiple sequence alignments of the 7 transcribed HERV-W *env *loci and the 332 HERV-W *env *cDNA sequences. A close inspection of multiple alignments unambiguously demonstrated that a high number of HERV-W *env *cDNAs, that is, 99 out of 332 (29.8%), represented recombinations between transcripts from different HERV-W *env *loci. Notably, the alleged breakpoints of recombined sequences appeared to be randomly distributed. Typical examples of recombined sequences are shown in Figure 2.

**Figure 3:**
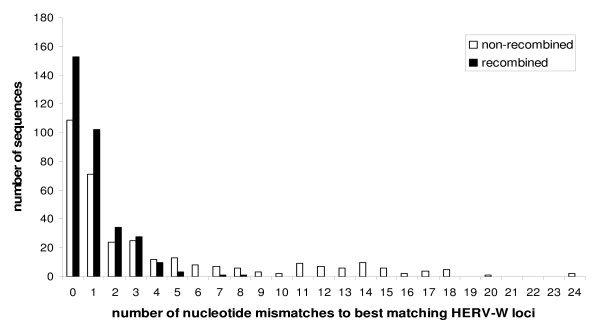
**Nucleotide mismatches between HERV-W *env *cDNAs and best matching genomic HERV-W *env *loci**. White bars represent the number of nucleotide mismatches between HERV-W *env *cDNAs (n = 332) and their best matching genomic HERV-W *env *locus without assuming the presence of recombined HERV-W *env *sequences among those cDNAs. Black bars indicate the number of nucleotide mismatches between HERV-W *env *cDNAs and their best matching genomic HERV-W *env *loci when the presence of recombination events in 99 out of 332 HERV-W *env *cDNAs (see Table 2) was taken into account.

When assuming recombinations, the number of nucleotide differences between HERV-W *env *cDNAs and the best matching genomic HERV-W *env *loci was strongly reduced compared to the number of nucleotide mismatches when recombinations were not assumed (Figure 3). Within the ~640 bp sequence analyzed, the average number of nucleotide mismatches between HERV-W *env *cDNAs and the best matching genomic HERV-W *env *loci was 3.69 per 640 bp (= 5.77/kb) when no recombinations were asssumed, as opposed to 0.98 per 640 bp (= 1.53/kb) when recombinations were assumed. The majority of recombined cDNAs (67%) resulted from one recombination event involving transcripts from two different HERV-W *env *loci. As for the other sequences, we were able to detect up to 4 recombination events involving up to five different HERV-W *env *loci (Table 2).

**Table 2:** Recombined HERV-W *env *cDNA sequences generated in this study

Recombination events	Number of involved loci	Number of cDNA sequences	Sum of involved HERV-W *env *transcripts
0	1	233	233

1	2	66	132

2	2	11	33

2	3	16	48

3	3	2	6

3	4	1	4

4	5	3	15

		Σ = 332	Σ = 471

Recombinations were detected by inspection of multiple alignments of cloned HERV-W *env *cDNA sequences and transcribed genomic HERV-W *env *loci identified in this work (for details, see Methods). Altogether, 99 out of 332 (29.8%) cDNA sequences analyzed represented recombined sequences. The sum of RNA transcripts from which recombined cDNA orignated is given in the right column. In the case that multiply recombined sequences involved the same locus more than once, it was assumed that sequences from the same locus originated from different transcripts from that locus.

### Differential transcriptional activity of HERV-W env loci in human PBMC

**Figure 4:**
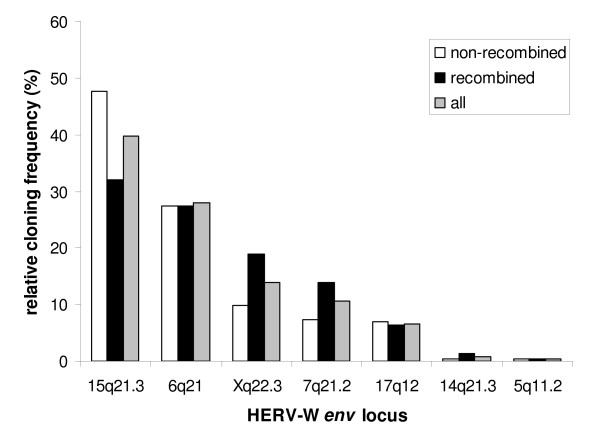
**Relative cloning frequencies of transcriptionally active HERV-W *env *loci in human PBMC**. The relative cloning frequencies are given as the number of cDNA clones from a particular HERV-W *env *locus relative to the number of all cDNA clones analyzed. Frequencies were calculated separately for all non-recombined clones (n = 233 sequences; white bars), all recombined clones (n = 99 sequences, originating from 238 transcripts [see text and Table 2]; black bars), and for non-recombined and recombined clones together (n = 332; originating from 471 transcripts [see text and Table 2]; gray bars).

Supposing that the relative cloning frequencies (the number of cDNA clones from a given individual HERV-W *env *locus relative to all cDNA clones analyzed) roughly reflect the relative abundance of RNA transcripts from individual HERV-W *env *loci in the total pool of HERV-W *env *RNAs in PBMCs, it is possible to estimate the relative transcriptional activity of individual HERV-W *env *loci in PBMC [34,35]. In a first analysis of pooled data from all 8 individuals studied, we used the 233 non-recombined cDNA sequences to calculate relative cloning frequencies. As shown in Figure 4, this demonstrated a differential transcriptional activity of expressed HERV-W *env *loci in human PBMC, with transcripts from a HERV-W *env *locus on chromosome 15q21.3 being most abundant (111 out of 233 sequences [47.6%]). In contrast, cDNAs from the 14q21.3 and 5q11.2 HERV-W *env *loci were only very rarely cloned (1 sequence from each locus out of 233 sequences [0.4%]).

To compare the transcriptional activity of different HERV-W *env *loci the PCR efficiencies with which these loci are amplified should be similar. PCR-efficiency mostly relies on the binding of primers to their target sequences. As can be seen in Figure 2, three of the seven amplified loci (7q21.2, 17q12, Xq22.3) perfectly matched to the primers and are thus expected to be amplified with similar efficiencies. The binding regions of three further loci (5q11.2, 6q21, 15q21.3) contained a single nucleotide mismatch in the 5' end of either the forward or the reverse primer. Because the 15q21.3 and the 6q21 loci were the most frequently cloned loci in our study, the single nucleotide mismatches in these two loci appear unlikely to have had a significant negative impact on amplification. In the case of the 5q11.2 locus this possibility cannot be excluded, but seems unlikely given the results for the 15q21.3 and 6q21 loci. One locus (14q21.3) displayed one additional nucleotide and one nucleotide mismatch in the binding region of the forward primer, and it seems possible that those mismatches may have negatively affected its amplification.

Since the finding of recombined HERV-W *env *cDNA sequences implies that the different HERV-W *env *loci that contributed to the recombined sequences must have been transcribed, we also estimated relative cloning frequencies based on the 99 recombined sequences, counting individually all HERV-W *env *loci that were present in the recombined sequences (see also Table 2). The relative cloning frequencies obtained in this evaluation were overall comparable to those of the non-recombined sequences (Figure 4), suggesting that the likelihood of taking part in recombinations correlates with transcript abundance.

Finally, not all transcriptionally active loci were detected as cDNA in every individual. Regarding non-recombined and recombined sequences together, transcripts from the 5q11.2 locus were detected in one, transcripts from the 14q21.3 in three, and transcripts from the 7q21.2 locus in seven of the eight individuals studied. The remaining HERV-W *env *loci (6q21, 15q21.3, 17q12, Xq22.3) were found to be transcriptionally active in the PBMC of every individual studied.

### Similar transcriptional activity of individual HERV-W env loci in PBMC from patients with MS and healthy controls

**Figure 5:**
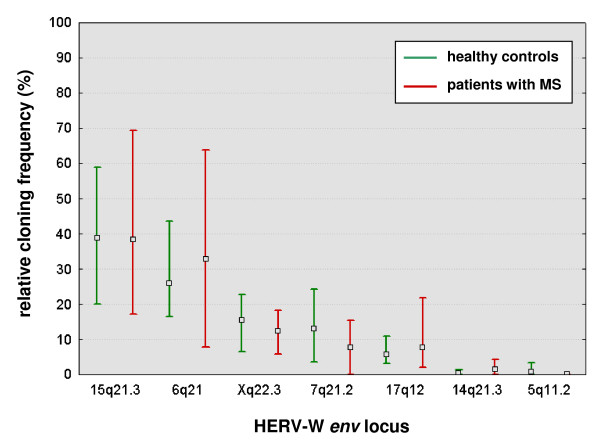
**Relative cloning frequencies of transcriptionally active HERV-W *env *loci in human PBMC from patients with MS and healthy controls**. Relative cloning frequencies were calculated for recombined and non-recombined clones together (n = 332 sequences, originating from 471 transcripts [see text and Table 2]). The box represents the mean, and the whiskers represent the minimum and maximum of the relative cloning frequencies of cDNAs from individual HERV-W *env *elements for the groups of patients with MS (n = 4) and healthy controls (n = 4). There were no statistically significant differences between patients and controls (p > 0.05; two-tailed Fisher's exact test).

We next evaluated whether the relative cloning frequencies and thus transcriptional activities of individual HERV-W *env *loci differ between patients with MS and healthy controls. Since the general pattern of transcriptional activity of individual HERV-W *env *loci was essentially the same regardless of whether recombined sequences were included in the evaluation or not (Figure 4), we analyzed data from all (non-recombined and recombined) sequences (see also Table 2). Figure 5 shows that the variability of the transcriptional activity of the different HERV-W *env *loci among the different individuals studied was overall quite high, suggesting inter-individual differences in the transcriptional activity of HERV-W *env *loci. However, there were no significant differences in the relative cloning frequencies of the different HERV-W *env *loci when the group of patients with MS was compared with the group of healthy controls (p > 0.05; two-tailed Fisher's exact test).

### MSRV sequences can be explained as originating from distinct HERV-W loci and recombinations among them

Having identified transcriptionally active HERV-W *env *loci in human PBMC, we were interested to know how previously published MSRV *env *sequences are related to those transcribed HERV-W *env *loci. Given the high frequency of *in vitro *recombinations between HERV-W *env *transcripts, we also wondered whether recombinations may be detectable in MSRV *env *sequences. To this end, we retrieved published MSRV sequences comprising parts of or the entire *env *region (n = 5) [7,23] from the NCBI database and analyzed them by BLAT searches (see Methods for details). Since all published MSRV *env *sequences are heterogeneous, it is *a priori *unlikely that all those sequences are derived from a single proviral insertion.

Quite strikingly, three of the MSRV *env *containing sequences (AF127227, AF127228, AF123882) could each be assigned to a distinct genomic HERV-W locus, namely, the HERV-W elements on chromosome 3q23, Xq22.3, and 15q21.3 (Table 3). The other two MSRV *env *sequences (AF127229 and AF331500) could be explained as recombinations between different HERV-W loci. AF127229 represents a recombination between two HERV-W loci located on chromsome 3p12.3 and 18q21.32. Likewise, AF331500 represents a recombination between two HERV-W elements on Xq22.3 and 5p12. Similar to the data shown in Figure 3, the number of nucleotide mismatches between AF127229 or AF331500 and the best matching genomic HERV-W loci was strongly reduced when recombinations were assumed (Table 3). Alignments of MSRV sequences with the respective best matching HERV-W loci are provided in additional file 3. Notably, two HERV-W elements (15q21.3 and Xq22.3) from which MSRV *env *sequences originated were found to be transcribed in human PBMC in the present work. The four other HERV-W elements (3q23, 3p12.3, 18q21.32, and 5p12) from which MSRV *env *sequences are derived were not identified as transcriptionally active in human PBMC in our investigation. However, due to various deletions the binding sites for one or both of the HERV-W *env *primers employed in our work are missing in those four HERV-W loci, indicating that corresponding cDNAs could not be amplified (data not shown). Therefore, it remains possible that those four HERV-W loci are transcriptionally active, too.

**Table 3:** Origin of previously described MSRV sequences

MSRV sequences	HERV-W source locus/loci	Number of nucleotide mismatches	
*env*		no recombinations	recombinations

AF127227 (544 bp)	3q23	1 (1.84/kb)	n.a.

AF127228 (1932 bp)	Xq22.3	4 (2.07/kb)	n.a.

AF127229 (2004 bp)	3p12.3/18q21.32	94 (46.91/kb)	3 (1.5/kb)

AF123882 (2477 bp)	15q21.3	5 (2.02/kb)	n.a.

AF331500 (1629 bp)	Xq22.3/5p12	31 (19.03/kb)	5 (3.07/kb)

*gag*			

AF123881 (1511 bp)	3q26.32	2 (1.32/kb)	n.a.

Previously published MSRV sequences were assigned by BLAT searches to corresponding HERV-W elements in the human genome. The accession number and length (base pairs, bp) of published MSRV clones are provided in the left column. The best matching HERV-W locus or loci (in case of recombined sequences) are indicated in column 2, and the number of nucleotide mismatches between MSRV sequences and the best matching genomic HERV-W elements in column 3. Note that for the recombined sequences, the number of nucleotide mismatches after assuming recombinations is markedly reduced. The average number of nucleotide differences of the 6 analyzed MSRV sequences to their best matching genomic HERV-W locus/loci was 1.97/kb. n.a., not applicable

We could also assign the formerly published MSRV *gag *sequence (AF123881) to a distinct HERV-W element on chromosome 3q26.32. This HERV-W locus has formerly been identified as transcriptionally active in human PBMC [39] and is identical to a HERV-W *gag *gene on chromosome 3 (AF156961), previously characterized by Voisset et al. [14]. Although the 3q26.32 HERV-W *gag *gene is incomplete, it contains the largest HERV-W *gag *ORF in the human genome, with a putative coding capacity for a 45 kDa HERV-W Gag protein, consisting of a complete matrix domain and a C-terminally truncated capsid, but lacking nucleocapsid [14].

Notably, the average number of nucleotide mismatches between MSRV *env *and *gag *sequences and the respective best matching genomic HERV-W loci (1.97/kb; see Table 3) were in the same range as that observed in our study of transcribed HERV-W *env *loci in human PBMC (1.53/kb). In summary, our analyses suggest that previously published MSRV sequences originated from genomic HERV-W loci, or recombinations among them.

### Relationship between Xq22.3 HERV-W env and MSRV env

**Figure 6:**
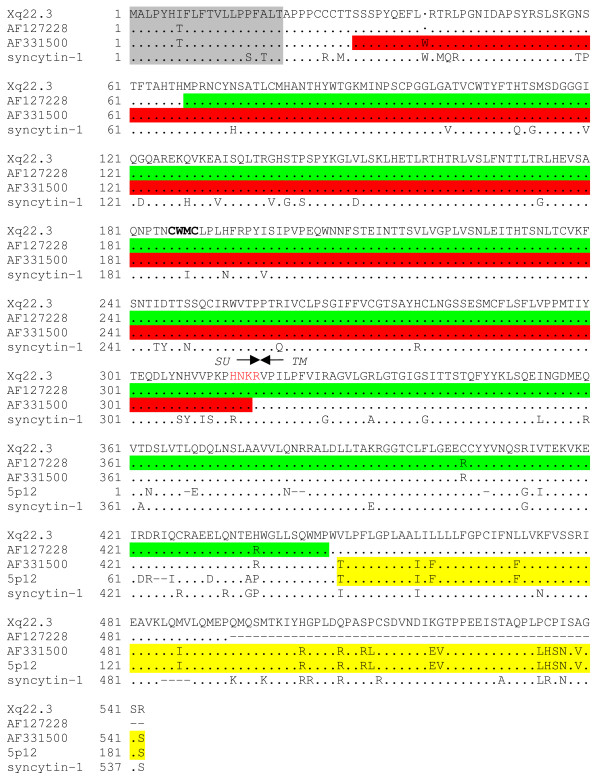
**Relationship between Xq22.3 HERV-W Env, MSRV Env, and Syncytin-1**. An amino acid sequence alignment of Xq22.3 HERV-W Env, MSRV Env (clones AF127228 and AF331500), and Syncytin-1 is shown. The sequence of a HERV-W element on chromosome 5p12 from which the C-terminal region of the MSRV *env *clone AF331500 is derived (see also Table 3 and Additional file 2) is also shown. For the sake of simplicity, only the C-terminal region of the 5p12 element is included. The region of MSRV Env (AF331500) originating from HERV-W 5p12 is highlighted in yellow. Predicted signal peptides (according to SignalP 3.0, ) are shaded in gray. The stop codon at position 39 of Xq22.3 HERV-W Env and AF127228 is indicated by a dot (·). The consensus C-X-X-C motif conserved among C-type and D-type retroviral Env proteins [42] is shown in boldface. The border between the SU and TM regions is indicated by arrows. The proteolytic cleavage site (consensus R/K-X-R/K-R) between SU and TM is highlighted in red letters. The sequences of the MSRV Env SU protein (generated using the MSRV *env *clone AF331500) studied by Rolland et al. [24] is marked in red. The fragment of the MSRV *env *clone AF127228 used for generation of the anti HERV-W Env monoclonal antibody 6A2B2 [16] is shown in green.

The MSRV *env *clone AF127228 and the SU and N-terminal TM regions of the MSRV *env *clone AF331500 correspond to the HERV-W *env *element on chromosome Xq22.3 which harbors a long ORF for a putative 475 amino acid HERV-W Env protein (Table 3). Accordingly, the amino acid sequence of a recombinant MSRV Env SU protein, which has been shown to have proinflammatory effects in various assays [24], and which was generated using the AF331500 MSRV *env *clone, is identical to the amino acid sequence of the HERV-W Env protein putatively encoded by Xq22.3 HERV-W *env *(Figure 6) [42]. However, in contrast to Xq22.3 HERV-W Env which is N-terminally truncated due to a stop codon (TGA) at position 39, this stop codon is a tryptophan residue (TGG) in the AF331500 MSRV *env *clone. Remarkably, the elimination of the stop codon at position 39 of HERV-W *env *Xq22.3 results in an uninterrupted full-length HERV-W *env *ORF, which could encode a complete HERV-W Env protein that contains a signal peptide (Figure 6). The origin of the stop codon mutation in the MSRV *env *AF331500 clone is unknown. Since several genomic HERV-W *env *elements display a TGG at the particular position, it is conceivable that a recombination event involving transcripts from the Xq22.3 locus and a short sequence stretch from one of the TGG containing HERV-W *env *loci might account for the reversal of the stop codon (data not shown).

In contrast to AF331500, the MSRV *env *clone AF127228, which likewise originates from the Xq22.3 HERV-W *env *locus, displays the stop codon at position 39. A DNA fragment comprising amino acids 68 to 446 of the HERV-W *env *ORF encoded by AF127228 has previously been used to generate the monoclonal anti HERV-W Env antibody 6A2B2 [16]. As shown in Figure 6, except for two amino acid exchanges in its C-terminus, the AF127228 amino acids 68 to 446 sequence is identical to the amino acid sequence of Xq22.3 HERV-W Env, but displays 38 mismatches to the Syncytin-1 amino acid sequence. Nevertheless, the 6A2B2 antibody may cross react with Syncytin-1 [16,43] and all previous neuropathological studies that reported a dysregulated expression of Syncytin-1 in MS lesions relied on the 6A2B2 antibody [18,21,22]. Still, assuming that HERV-W Xq22.3 *env *may have the potential to code for a HERV-W Env protein, our findings open the intriguing possibility that the protein detected by 6A2B2 in MS lesions could instead have originated from the HERV-W *env *locus on chromosome Xq22.3.

## Discussion

When studying HERV RNA expression in human diseases, it seems important to clearly dissect from which genomic HERV loci the detected HERV RNA transcripts originate [44]. Consistent with previous findings suggesting that expression of HERV transcripts is a ubiquitious phenomenon occurring in every human tissue [34,45,46], we herein show that at least seven HERV-W *env *loci are transcribed in PBMC from patients with MS and healthy controls. Since the primers used in this investigation only amplify a limited number of genomic HERV-W *env *elements our study is not exhaustive, and it seems rather likely that more than seven HERV-W *env *elements are transcriptionally active in human PBMC. Additionally, HERV-W *env *loci that are transcribed at very low rates could be missed in the cloning procedure unless much higher numbers of clones are generated. Three of the transcribed HERV-W *env *elements (15q21.3, Xq22.3, 17q12) identified in this study were previously found to be expressed in human PBMC by a cloning and sequencing approach [38,39]. Assignment of cDNAs to genomic HERV-W *env *loci in the former investigations was based on rather short sequences (30 bp, excluding primers), containing only few informative nucleotides, that is, nucleotides that are exclusively present in a single genomic HERV-W *env *locus and thereby allow unambiguous assignment of cDNAs. Usage of a ~600 bp sequence (excluding primers) in the present work resulted in a higher number of informative nucleotides and thus strengthened the accuracy of the assignment. Our finding of ERVWE1 transcripts in human PBMC is consistent with previous observations [19,20] and corroborates that although ERVWE1 expression is most abundant in placenta this locus is transcribed in non-placental tissues as well [15].

Several studies have analyzed expression of HERV-W *env *RNA in PBMC or brain tissue from patients with MS [18-21]. Lack of systematic cloning, sequencing, and assignment of cDNA sequences to genomic HERV-W *env *loci have impaired the exact identification of transcriptionally active genomic HERV-W *env *loci responsible for the observed HERV-W *env *RNA expression in these investigations. Whereas in the present detailed analysis we could identify distinct transcriptionally active HERV-W *env *loci, we did not observe significant differences in the transcriptional activity of those loci in PBMC from patients with MS versus healthy controls. Although the number of individuals studied was rather small, these data argue against a dysregulated transcription pattern of HERV-W *env *in PBMC from patients with MS. In contrast, a consistent finding of former investigations was a significantly higher global HERV-W *env *RNA expression in brain tissue from patients with MS as compared to brain tissue from patients with other neurological diseases or normal brain tissue [18-21]. Using the methodological approach of the present work, it will therefore be interesting to identify the HERV-W *env *elements underlying upregulated HERV-W *env *RNA expression in MS brain tissue.

Antony and coworkers addressed this question by designing primers that specifically amplify HERV-W *env *7q21.2 (ERVWE1) and the MSRV *env *clone AF123882 [20], which, as shown by our analyses, corresponds to a HERV-W *env *element on chromosome 15q21.3. These authors also employed a pair of degenerate (HERV-W_deg_) *env *primers that were based on the MSRV *env *clone AF331500, which, again as shown in this work, corresponds to a recombined cDNA originating from HERV-W *env *loci on Xq22.3 and 5p12. According to the Antony et al. study, elevated HERV-W *env *RNA expression in MS brain tissue originates mainly from the HERV-W *env *elements amplified by the HERV-W_deg _*env *primers and (somewhat less) from HERV-W *env *7q21.2, while HERV-W *env *15q21.3 expression was similar in patients and controls [20]. A BLAT-PCR search showed that the HERV-W_deg _*env *primer pair potentially amplifies at least three genomic HERV-W *env *loci, among them HERV-W *env *Xq22.3. It is thus tempting to speculate that HERV-W *env *Xq22.3 may significantly contribute to increased HERV-W *env *RNA expression in MS brain tissue. Again, using the methods described herein, this issue could be resolved in a straightforward manner.

Remarkably, we observed a high number (29.8%) of recombined sequences among the analyzed HERV-W *env *cDNAs. As detailed in a previous study on transcribed HERV-K(HML-2) sequences [41], those recombinant cDNA sequences very likely resulted from *in vitro *recombinations that were due to template switches of reverse transcriptase during cDNA synthesis and/or PCR-mediated recombinations. Both of these mechanisms are well-recognized and have been proven experimentally to produce chimeric sequences [41,47-53]. The percentage of recombined sequences detected in the present study was higher than that in the study on HERV-K(HML-2) in which ~5% of recombined clones were observed [41]. This is most likely explained by the fact that in the HERV-K(HML-2) study only cDNA sequences with more than 17 nucleotide mismatches to the best matching locus were analyzed for recombinations, whereas in the present work all cDNA sequences were scrutinized for recombinations. Altogether, our data indicate that during experimental studies of repetitive elements by RT-PCR, *in vitro *recombinations are relatively common and almost inevitable complications.

An important result of this investigation is that previously published MSRV *env *and *gag *sequences appear to either be derived from transcripts of specific genomic HERV-W elements or to result from recombinations among such transcripts (Table 3). Given the high frequency of *in vitro *recombinations between transcripts from different HERV loci observed in this and the study by Flockerzi et al. [41], and given that MSRV clones were generated by methodologically similar approaches, it seems possible that the recombined MSRV *env *sequences (AF127229, AF331500) have resulted from *in vitro *recombinations as well.

An alternative explanation is that the recombined MSRV *env *sequences, and the recombined HERV-W *env *sequences isolated in this study, originated from novel, recombined, genomic HERV-W insertions. Hypothetically, such insertions could have formed *in vivo *after recombination of RNA transcripts from different HERV-W *env *loci through template switches during reverse transcription. Although we cannot formally exclude this possibility, a number of points argue against it. First, all known HERV-W elements are defective and replication-incompetent [8,14]. Therefore, HERV-W is *a priori *rather unlikely to have the capacity to form new insertions in human DNA. Second, if there were novel recombined HERV-W loci in human DNA, one would expect to repeatedly observe defined recombined sequences originating from such insertions. However, this is neither the case with the 99 recombined HERV-W *env *cDNA sequences analyzed in this study nor with the published MSRV *env *clones. Third, given that about 30% of HERV-W *env *and 33% (2 of 6) of the investigated MSRV sequences represent recombinants, if all these recombinant MSRV/HERV-W *env *sequences were derived from novel proviral insertions, formation of such novel insertions would be an astonishingly frequent event. It seems very unlikely that as many recombined HERV-W loci should have been overlooked in previous genome sequencing projects.

Collectively, the most plausible and simplest explanation for the origin of MSRV *env *and *gag *sequences seems to be that those sequences originate from RNA transcripts from various endogenous HERV-W loci, or from *in vitro *recombinations among them. All of the HERV-W loci from which MSRV sequences are derived are defective and except for the 5p12 HERV-W *env *element, all of those loci resemble processed HERV-W pseudogenes. The human genome sequence was not yet available when MSRV was described, which hampered the identification of the precise origin of MSRV sequences at that time. It was, however, noted that those sequences cannot be attributed to a single replication-competent genome [7]. Nevertheless, the nature of MSRV was subsequently controversial, and it has been speculated that MSRV could be an exogenous, replication-competent retrovirus [6,30-32]. In contrast, our present data clearly suggest that the published MSRV *env *and *gag *RNA sequences are not derived from the genome of a currently replication-competent exogenous retrovirus. In the light of these results and previous observations of an increased prevalence of MSRV *pol *transcripts in plasma from patients with MS as compared to healthy controls [54,55], it may similarly be interesting to analyze which HERV-W *pol *elements those MSRV *pol *transcripts could be derived from.

Although our findings argue against MSRV being an autonomous retroviral entity, they do by no means rule out that individual HERV-W *env *loci that correspond to MSRV sequences, or the Syncytin-1 (ERVWE1) gene, could be of relevance in MS. Indeed, we show that two MSRV *env *clones (AF331500, AF127228), which have been instrumental for the characterization of proinflammatory effects of MSRV Env [24] and the generation of a monoclonal anti-MSRV/HERV-W Env antibody (6A2B2) [16], are derived from a HERV-W *env *locus on chromosome Xq22.3. This locus is highly remarkable as it is interrupted by only a single premature stop at codon position 39 and otherwise harbors a long ORF for a N-terminally truncated 475 amino acid HERV-W Env protein (Figure 6). Bonnaud and colleagues described frameshift insertions/deletions (indels), that is, indels whose length is not a multiple of three, in 33 out of 36 analyzed genomic HERV-W *env *loci. Interestingly, among the three loci without frameshift indels were the ERVWE1 gene and the Xq22.3 HERV-W *env *element [43]. We further note that the 475 amino acid Xq22.3 HERV-W *env *ORF is also present in the orthologous locus in chimpanzees (data not shown). These data may be taken as hints that selective pressure could act on the Xq22.3 HERV-W *env *locus, raising the possibility that Xq22.3 HERV-W *env *could exert a biological function. Our finding that the Xq22.3 HERV-W *env *locus is transcriptionally active in human cells indicates that it fulfills at least one essential prerequisite for a protein expression capacity *in vivo*.

Neuropathological studies revealed that the 6A2B2 anti-HERV-W Env antibody reacts with an antigen that is strongly expressed by glial cells in MS brain lesions, but not in normal control brain tissue [18,21,22]. Because Syncytin-1 has been thought to be the only HERV-W *env *locus capable of producing a HERV-W Env protein, and because 6A2B2 may crossreact with Syncytin-1 [16,43], the antigen detected by 6A2B2 in MS brain lesions was considered to be Syncytin-1. However, our analyses show that the protein against which the 6A2B2 antibody was raised is practically identical to the Xq22.3 HERV-W Env protein (Figure 6) [16]. We meanwhile cloned Xq22.3 HERV-W *env *into a eukaryotic expression vector. Transient transfection of HeLa cells with this clone showed that the Xq22.3 HERV-W *env *has retained a coding capacity and can produce a HERV-W Env protein *in vitro *which is detected by the 6A2B2 antibody in immunocytochemistry and immunoblots (C. Crusius, S. Wahl, K. Ruprecht, manuscript in preparation). These data suggest that the antigen recognized by 6A2B2 in MS lesions could likewise originate from the Xq22.3 HERV-W *env *locus, provided that this locus has a protein expression capacity *in vivo*. More elaborate studies will be required to clarify the exact nature of the HERV-W Env protein detected in MS lesions. Further characterization of the putative Xq22.3-encoded HERV-W Env protein, especially in comparison to Syncytin-1, will be necessary for such clarification.

## Conclusion

In conclusion, we demonstrate that several HERV-W *env *loci are transcribed in human PBMC, and that analysis of such transcribed HERV-W *env *elements is complicated by frequent recombinations, which are most likely generated *in vitro*. Based on these findings, we show that previously reported MSRV *env *and *gag *sequences can be explained as originating from (in some instances recombined) transcripts of defective HERV-W elements, arguing against MSRV sequences being derived from an infectious exogenous retrovirus. Our results should help to settle the issue of the nature of MSRV and contribute to the clarification of the roles of MSRV versus HERV-W Env (Syncytin-1) in MS. Indeed, our findings raise the intriguing possibility that a protein encoded by a HERV-W *env *element on chromosome Xq22.3 could be expressed in MS brain lesions.

## Methods

### Patients with MS and healthy controls

Four patients with MS (3 female, 1 male) and 4 healthy controls (2 female, 2 male) were included in this study. The median age of patients was 34 (range 29–39) and of controls 34.5 (29–41) years. Clinical data of patients with MS were obtained by review of the medical records. All patients with MS had a diagnosis of definite MS according to Poser's criteria [56]. Three patients had relapsing-remitting MS, and one patient had secondary progressive MS. The median expanded disability status scale score of patients with MS was 3.25 (1.5–6.5). One patient was treated with interferon-beta 1a, and two patients were treated with glatiramer acetate by the time of blood collection. None of the patients had been treated with glucocorticosteroids for at least 6 months before blood collection. Participants provided written informed consent, and the study was approved by the ethics committee of the faculty of medicine, Julius-Maximilians University, Würzburg. PBMC samples used in this work were collected and purified with Lymphoprep (Axis Shield, Oslo, Norway) gradient centrifugation as described before [57]. Samples were stored at -80°C prior to the present analysis.

### RT-PCR

PBMC were thawed and cultured overnight in RPMI 1640 (BioWhittaker) supplemented with 10% FCS and penicillin (100 U/ml) and streptomycin (100 μg/ml) at 37°C in a humidified 5% (v/v) CO_2 _atmosphere. Total RNA was extracted from PBMC using the RNeasy Mini kit (Qiagen) and eluted in 60 μl of distilled water. RNA concentration and purity were assessed spectrophotometrically. Contaminating DNA was removed using the TURBO DNA-free Kit (Ambion Inc.) following the protocol for rigorous DNAse treatment. In brief, 2 units of TURBO DNase were added to a 50-μl reaction containing 10 μg RNA and incubated for 30 minutes at 37°C. Another 2 units of TURBO DNase were added and the incubation was continued for 30 minutes at 37°C. DNAse was removed using 10 μl of the provided DNAse inactivation reagent. Subsequently, 0.3–0.5 μg of DNase digested cellular RNA was reverse transcribed in a 20-μl reaction using Superscript II (Invitrogen) and 25 μM random hexamer primers (MWG-Biotech AG) according to the protocol of the manufacturer. Negative controls were generated in parallel for each sample by omission of Superscript II from the reaction. PCR primer sequences for amplification of HERV-W *env *were as follows: forward primer 5'-TTCACTGCCCACACCCAT-3'; reverse primer 5'-GAGGTACCACAGACAAAAAATATTCCT-3'. Conventional PCR was performed in a 50-μl reaction containing 1 μl of cDNA, 0.5 μM of each primer, 200 μM of each dNTP, reaction buffer (10 mM Tris-HCl, 50 mM KCl, 1.5 mM MgCl_2_), and 0.05 units/μl of *Taq *DNA Polymerase (D1806, Sigma). Cycling parameters were as follows: 3 minutes at 95°C; 40 cycles of 50 sec at 95°C, 50 sec at 58°C, and 1 minute at 72°C; and 10 minutes at 72°C.

### Cloning of HERV-W env transcripts and assignment to proviral HERV-W loci

PCR products were excised from agarose gels, purified (NucleoSpin Extract II, Macherey-Nagel), and ligated into the pGEM-T vector (Promega). Plasmid DNA from randomly selected insert-containing clones was purified with the QIAprep Miniprep kit (Qiagen) and sequenced on an Applied Biosystems 3730x Capillary Sequencer using vector-specific primers (Institut für Immunologie und Genetik, Kaiserlautern, Germany). The quality of chromatograms was assessed by visual inspection. Poor-quality reads (< 0.1% of all sequences) were excluded from the analysis.

Assignment of cDNA sequences to corresponding HERV-W *env *loci is based on random and thus characteristic nucleotide differences between the various genomic HERV-W *env *loci. The proviral HERV-W *env *locus with no or very few nucleotide mismatches to a HERV-W *env *cDNA sequence can be assumed to represent the origin of this cDNA, if all other alternative loci displayed more nucleotide differences. A detailed discussion of the sequence assignment strategy has recently been provided [34].

To assign HERV-W *env *cDNA clones to specific HERV-W *env *loci in the human genome, HERV-W *env *cDNA sequences were first analyzed by BLAT searches (; March 2006 human genome assembly). To further study recombinations between different HERV-W *env *loci in HERV-W *env *cDNA sequences, sequences of the seven transcribed HERV-W *env *loci were retrieved from the human genome sequence (March 2006 assembly) at the Human Genome Browser and multiply aligned with HERV-W *env *cDNA sequences using Muscle 3.6 [58]. Candidate HERV-W *env *cDNA sequences were then inspected for recombination events.

### Analysis of MSRV sequences

Previously published MSRV *env *and *gag *sequences were retrieved from GenBank and analyzed by BLAT searches to identify endogenous HERV-W loci with similarities to MSRV sequences. Alignments of the MSRV sequences with the best matching HERV-W locus were manually inspected for evidence of recombination events. In recombined sequence portions, nucleotide mismatches between MSRV sequences and the best matching HERV-W sequence usually clustered in defined subregions. Presumably recombined subregions were used as probe sequences for another BLAT search to detect their best matching HERV-W locus. Sequences of thus identified HERV-W loci were again retrieved from the Human Genome Browser and aligned with the corresponding MSRV sequences.

## Competing interests

The authors declare that they have no competing interests.

## Authors' contributions

JM, KR, and NML conceived of the study, participated in its design, and provided funding. GL, BFM, and KR carried out the molecular genetic studies. GL, KR, and JM analyzed the data. KR drafted the manuscript. All authors read and approved the final manuscript.

## Supplementary Material

Additional file 1 **Sequences of the 332 HERV-W *env *cDNAs analyzed in this study**. This file contains raw sequence data of the 332 HERV-W *env *cDNAs analyzed in this work. Click here for file

Additional file 2 **Pustell matrix comparisons of the seven HERV-W *env *loci identified as transcriptionally active in human PBMC in this study**. This file contains Pustell matrix comparisons of the Repbase  HERV-W reference sequence with the seven HERV-W *env *loci identified as transcriptionally active in human PBMC in this work. Click here for file

Additional file 3 **Alignments of previously published MSRV *env *and *gag *sequences with their corresponding genomic HERV-W elements**. This file contains annotated alignments of previously published MSRV *env *and *gag *sequences with the genomic HERV-W elements from which the respective MSRV sequences are most likely derived. Click here for file
