# Supplementary material for: Analysis of transcribed human endogenous retrovirus W env loci clarifies the origin of multiple sclerosis-associated retrovirus env sequences
Source: Retrovirology. 2009 Apr 15;6:37. doi: 10.1186/1742-4690-6-37 (PMC2672075; doi:10.1186/1742-4690-6-37)
Supplement: Additional file 1 — Sequences of the 332 HERV-W env cDNAs analyzed in this study. This file contains raw sequence data of the 332 HERV-W env cDNAs analyzed in this work. [file 1742-4690-6-37-S1.doc]

**Laufer et al. additional file 1**

Sequences of the 332 HERV-W env cDNAs analyzed in this study. Sequences from each of the eight individuals studied are grouped. Patients with MS are designated MS-I to MS-IV and healthy controls KO-I to KO-IV. Note that data represent raw sequence data and therefore some sequences are in reverse complementary order.

**MS-I**

>GL-1-K1_A03

TTCACTGCCCACACCCATATGCCCCACAACTGCTATAACTCTGCCACTCTTTGCATGCGTGCAAATACTCATTATTGGACAGGGAAAATGATTAATCCTAGTTGTCCTGGAGGACTTGGAGCCACTGTCTGTTGGACTTAGTTCACCCATACTGGTATGTCTGATAGGGGTGGAGTTCAAGATCAGGCAAGAGAAAAACACGTAAAGGAAGTAATCTCCCAACTGACCTGAGTACATAGCACGCCTAGCCCCTACAAAGGACTAGATCTCTCAAAACTACATGAAACCCTCCGTACCCATACTCGCCTGGTAAGCCTATTTAATACCACCCTCACTGGGTTTCATGAGGTCTCGGCCCAAAACCCTGCTAACTGTTGGATGTGCCTCCCTCTGCACTTAAGGCCATACATTTCAATCCCTGTACCTGAACAAGGGAACAACTTCAACACATAAACACCACTTCCGTTTTAGTAGGACCTCTTGTTTCCAATCTGGAAATAACCCATACCTCAAACCTCACCTGTGTAAAATTTAGCAATACGATAGACACAACCAACTCCCAATGCATCAGGTGGGTAACTCCCCCCCCACGAATAGTCTGCCTACCCTCAGGAATATTTTTTGTCTGTGGTACCTC

>GL-1-K2_B03

GAGGTACCACAGACAAAAAATATTCCTGAGGGTAGGCAGACTATTCGTGTGGGAGGTGTTACCCACCTGATGCATTGGGAGCTGGTTGTGTCTATAGTATTGCTAAATTTTACACAGGTGAGGTTTGAGGTATGGGTTATTTCCAGATTGGAAACAAGAGGTCATACTAAAACGGAAGTGGTGTTTATTTCTGTGCTGAAGTTGTTCCATTGTTCAGGAACAGGGATTGAAATGTATGGCCTGAAGTGCAGGGGGAGGCACATCCAACAGTTAGTAGGGTTTTGGGCTGAGACCTCATGGAGCCGAGTGAGGGTGGTATTAAATAGGCTCACCAGGCGAGTATGGGTACGGAGGGTTTCATGTAGTTTTGAGAGAACTAGTCCTTTGTAGGGGCTAGGGGTGCTATGTCCCCGGGTCAGTTGGGAGATTGCTTCCTTTACTTGTTTTTCTCTTGCCTGACCTTGAATTCCACCCCCATCAGACATACTGGTATGGGTGAAGTAAGTCCAACAGACAGTGGCTCCAAGTCCTCCAGGACAACTAGGATTAATCATTTTCCCTGTCCAATAATGAGTATTTGCATGCATGCAAAGAGTGGCAGAGTTATAGCAGTTGCGGGGCATATGGGTGTGGGCAGTGAA

>GL-1-K4_C03

TTCACTGCCCACACCCATATGCCCCACAACTGCTATAACTCTGCCACTCTTTGCATGCATGCAAATACTCATTATTGGACAGGGAAAATGATTAATCCTAGTTGTCCTGGAAGACTTGGAGCCACTGTCTGTCGGACTTACTTCACCCATACTGGTATGTCTGAGGGGGGTGGAGTTCAAGATCAGGCAAGAGAAAAACATGTAAAGGAAGTAACCTCCCAACTGACCCGGGTACATAGCACCCCTAGCCCCTACAAAGGACTAGATCTCTTAAAACTACATGAAACCCTCCATACCCATACTTGCCTGGTAAGCCTATTTAATACCACCCTCACTGGGCTCCATGAGGTCTCGGCCCAAAACCCTACTAACTGTTGGATGTGCCTCCCCCTGTATTTCAGGCCATGCATTTCAATCCCTGTACCTGAACAATGGAACAACTACAGCACAGAAATAAACACCACTTCCGTTTTAGTAGGACCTCTTGTTTCCAATCTGGAAATAACCCATACCTCAAACCTCACCTGTGTAAAATTTAGCAATACTGTAGACACAACCAACTCCCAATGCATCAGGTGGGTAACTCCTCCCACACGAATAGTCTGCCTACCCTCAGGAATATTTTTTGTCTGTGGTACCTC

>GL-1-K5_D03

TTCACTGCCCACACCCATATGCCCCACAACTGCTATAACTCTGCCACTCTTTGCATGCATGCAAATACTCATTATTGGACAGGGAAAATGATTAATCCTAGTTGTCCTGGAAGACTTGGAGCCACTGTCTGTCGGACTTACTTCACCCATACTGGTATGTCTGAGGGGGGTGGAGTTCAAGATCAGGCAAGAGAAAAACATGCAAAGGAAGTAACCTCCCAACTGACCCGGGTACATAGCACCCCTAGCCCCTACAAAGGACTAGATCTCTTAAAACTACATGAAACCCTCCATACCCATACTTGCCTGGTAAGCCTATTTAATACCACCCTCACTGGGCTCCATGAGGTCTCGGCCCAAAACCCTACTAACTGTTGGATGTGCCTCCCCCTGTATTTCAGGCCATGCATTTCAATCCCTGTACCTGAACAATGGAACAACCACAGCACAGAAATAAACACCACTTCCGTTTTAGTAGGACCTCTTGTTTCCAATCTGGAAATAACCCATACCTCAAACCTCACCTGTGTAAAATTTAGCAATACGATAGACACAACCAACTCCCAATGCATCAGGTGGGTAACTCCCCCCCACGAATAGTCTGCCTACCCTCAGGAATATTTTTTGTCTGTGGTACCTC

>GL-1-K7_E03

GAGGTACCACAGACAAAAAATATTCCTGAGGGTAGGCAGACTATTCGTGTGGGAGGTGTTACCCACCTGATGCATTGGGAGCTGGTTGTGTCTATAGTATTGCTAAATTTTACACAGGTGAGGTTTGAGGTATGGGTTATTTCCAGATTGGAAACAAGAGGTCATACTAAAACGGAAGTGGTGTTTATTTCTGTGCTGTAGTTGTTCCATTGTTCAGGTACAGGGATTGAAATGCATGGCCTGAAATACAGGGGGAGGCACATCCAACAGTTAGTAGGGTTTTGGGCCGAGACCTCATGGAGCCCAGTGAGGGTGGTATTAAATAGGCTTACCAGGCAAGTATGGGTATGGAGGGTTTCATGTAGTTTTAAGAGATCTAGTCCTTTGTAGGGGCTAGGGGTGCTATGTACCCGGGTCAGTTGGGAGGTTACTTCCTTTACATGTTTTTCTCTTGCCTGATCTTGAACTCCACCCCCCTCAGACATACCAGTATGGGTGAAGTAAGTCCGACAGACAGTGGCTCCAAGTCTTCCAGGACAACTAGGATTAATCATTTTCCCTGTCCAATAATGAGTATTTGCATGCATGCAAAGAGTGGCAGAGTTATAGCAGTTGTGGGGCATATGGGTGTGGGCAGTGAA

>GL-1-K9_F03

GAGGTACCACAGACAAAAAATATTCCTGAGGGTAGGCAGACTATTCGTGTGGGAGGTGTTACCCACCTGATGCATTGGGAGCTGGTTGTGTCTATAGTATTGCTAAATTTTACACAGGTGAGGTTTGAGGTATGGGTTATTTCCAGATTGGAAACAAGAGGTCCTACTAAAACGGAAGTGGTGTTTATTTCTGTGCTGAAGTTGTTCCATTGTTCAGGAACAGGGATTGAAATGTATGGCCTGAAGTGCAGGGGGAGGCACATCCAACAGTTAGTAGGGTTTTGGGCTGAGACCTCATGGAGCCGAGTGAGGGTGGTATTAAATAGGCTCACCAGGCGAGTATGGGTACGGAGGGTTTCATGTAGTTTTGAGAGAACTAGTCCTTTGTAGGGGCTAGGGGTGCTATGTCCCCGGGTCAGTTGGGAGATTGCTTCCTTTACTTGTTTTTCTCTTGCCTGACCTTGAATTCCACCCCCATCAGACATACTGGTATGGGTGAAGTAAGTCCAACAGACAGTGGCTCCAAGTCCTCCAGGACAACTAGGATTAATCATTTTTCCTGTCCAATAATGAGTATTTGCATGCATGCAAAGAGTGGCAGAGTGATAGCAGTTGCGGGGCATATGGGTGTGGGCAGTGAA

>GL-1-K10_G03

GAGGTACCACAGACAAAAAATATTCCTGAGGGTAGGCAGACTATTCGTGTGGGAGGAGTTACCCACCTGATGCATTGGGAGTTGGCTGTGTCTATAGTATTGCTAAATTTTACACAGGTGAGGTTTGAGGTATGGGTTATTTCCAGATTGGAAAGAGGACCTACTAAAACAGAAGTGGTGTTTATTTCTGTGCTGAAGTTGTTCCATTGTTCAGGTATAGGGATTGAAATGTATGGCCTAAAGCGCAGGGGGAGGCACATCCAACAGTTAGTAGGGTTTTGGGCCGAGACCTCATGGAGCCCAGTCAGGGTGGTATTAAATAGGCTTACCAGGCCAGTATGGGTATGGAGGGTTTCATGTAGTTTTGAGAGATCTAGTCCTTTGTAGGGGCCAGGGGTGCTATGTACCCAGGTCAGTTGGGAGGTTACTTCCTTTACATGTTTTTCTCTTGCCTGATCTTGAACTCCACCCCCCTCAGGCATACCAGTATGGGTGAAGTAAGTCCGACAGACAGTGGCTCCAAGTCTTCCAGGACAACTAGGATTAATCATTTTCCCTGTCCAATAATGAGTATTTGCATGCATGCAAAGAGTGGCAGAGTTATAGCAGTTGTGGGGCATATGGGTGTGGGCAGTGAA

>GL-1-K11_H03

TTCACTGCCCACACCCATATGCCCCACAACTGCTATAACTCTGCCACTCTTTGCATGCATGCAAATACTCATTATTGGACAGGGAAAATGATTAATCCTAGTTGTCCTGGAAGACTTGGAGCCACTGTCTGTCGGACTTACTTCACCCATACTGGTATGTCTGAGGGGGGTGGAGTTCAAGATCAGGCAAGAGAAAAACATGTAAAGGAAGTAACCTCCCAACTGACCCGGGTACATAGCACCCCTAGCCCCTACAAAGGACTAGATCTCTTAAAACTACATGAAACCCTCCATACCCATACTTGCCTGGTAAGCCTATTTAATACCACCCTCACTGGGCTCCATGAGGTCTCGGCCCAAAACCCTACTAACTGTTGGATGTGCCTCCCCCTGTATTTCAGGCCATGCATTTCAATCCCTGTACCTGAACAATGGGACAACTACAGCACAGAAATAAACACCACTTCCGTTTTAGTAGGACCTCTTGTTTCCAATCTGGAAATAACCCATACCTCAAACCTCACCTGTGTAAAATTTAGCAATACTGTAGACACAACCAACTCCCAATGCATCAGGTGGGTAACTCCTCCCACACGAATAGTCTGCCTACCCTCAGGAATATTTTTTGTCTGTGGTACCTC

>GL-1-K12_A04

GAGGTACCACAGACAAAAAATATTCCTGAGGGTAGGCAGACTATTCGTGTGGGAGGTGTTACCCACCTGATGCATTGGGAGCTGGTTGTGTCTATAGTATTGCTAAATTTTACACAGGTGAGGTTTGAGGTATGGGTTATTTCCAGATTGGAAACAAGAGGTCATACTAAAACGGAAGTGGTGTTTATTTCTGTGCTGAAGTTGTTCCATTGTTCAGGAACAGGGATTGAAATGTATGGCCTGAAGTGCAGGGGGAGGCACATCCAACAGTTAGTAGGGTTTTGGGCTGAGACCTCATGGAGCCGAGTGAGGGTGGTATTAAATAGGCTCACCAGGCGAGTATGGGTACGGAGGGTTTCATGTAGTTTTGAGAGAACTAGTCCTTTGTAGGGGCTAGGGGTGCTATGTCCCCGGGTCAGTTGGGAGATTGCTTCCTTTACTTGTTTTTCTCTTGCCTGACCTTGAATTCCACCCCCATCAGACATACTGGTATGGGTGAAGTAAGTCCAACAGACAGTGGCTCCAAGTCCTCCAGGACAACTAGGATTAATCATTTTCCCTGTCCAATAATGAGTATTTGCATGCATGCAAAGAGTGGCAGAGTTATAGCAGTTGCGGGGCATATGGGTGTGGGCAGTGAA

>GL-1-K13_D09

GAGGTACCACAGACAAAAAATATTCCTGAGGGTAGGCAGACTATTCGTGTGGGAGGAGTTACCCACCTGATGCATTGGGAGTTGGTTGTGTCTACAGTATTGCTAAATTTTACACAGGTGAGGTTTGAGGTATGGGTTATTTCCAGATTGGAAACAAGAGGTCCTACTAAAACGGAAGTGGTGTTTATTTCTGTGCTGTAGTTGTTCCATTGTTCAGGTACAGGGATTGAAATGCATGGCCTGAAATACAGGGGGAGGCACATCCAACAGTTAGTAGGGTTTTGGGCCGAGACCTCATGGAGCCCAGTGAGGGTGGTATTAAATAGGCTTACCAGGCAAGTATGGGTATGGAGGGTTTCATGTAGTTTTAAGAGATCTAGTCCTTTGTAGGGGCTAGGGGTGCTATGTACCCGGGTCAGTTGGGAGGTTACTTCCTTTACATGTTTTTCTCTTGCCTGATCTTGAACTCCACCCCCCTCAGACATACCAGTATGGGTGAAGTAAGTCCGACAGACAGTGGCTCCAAGTCTTCCAGGACAACTAGGATTAATCATTTTCCCTGTCCAGTAATGAGTATTTGCATGCATGCAAAGAGTGGCAGAGTTATAGCAGTTGTGGGGCATATGGGTGTGGGCAGTGAA

>GL-1-K14_E09

GAGGGTAGGCAGACTATTCGTGTGGGAGGAGTTACCCACCTGATGCATTGGGAGTTGGTTGTGTCTACAGTATTGCTAAATTTTACACAGGTGAGGTTTGAGGTATGGGTTATTTCCAGATTGGAAACAAGAGGTCCTACTAAAACGGAAGTGGTGTTTATTTCTGTGCTGTAGTTGTTCCATTGTTCAGGTACAGGGATTGAAATGCATGGCCTGAAATACAGGGGGAGGCACATCCAACAGTTAGTAGGGTTTTGGGCCGAGACCTCATGGAGCCCAGTGAGGGTGGTATTAAATAGGCTTACCAGGCAAGTATGGGTATGGAGGGTTTCATGTAGTTTTAAGAGATCTAGTCCTTTGTAGGGGCTAGGGGTGCTATGTTCCCGGGTCAGTTAGGAGGTTACTTCCTTTACATGTTTTTCTCTTGCCTGATCTTGAACTCCACCCCCCTCAGACATACCAGTATGGGTGAAGTAAGTCCGACAGACAGTGGCTCCAAGTCTTCCAGGACAACTAGGATTAATCATTTTCCCTGTCCAATAATGAGTATTTGCATGCATGCAAAGAGTGGCAGAGTTATAGCAGTTGTGGGGCATATGGGTGTGGGCAGTGAA

>GL-1-K15_F09

TTCACTGCCCACACCCATATGCCCCACAACTGCTATAACTCTGCCACTCTATGCATGCATGCAAATACTCATTATTGGACAGGGAAAATGATTAATCCTAGTTGTCCTGGAAGACTTGGAGCCACTGTCTGTCGGACTTACTTCACCCATACTGGTATGTCTGAGGGGGGTGGAGTTCAAGATCAGGCAAGAGAAAAACATGTAAAGGAAGTAACCTCCCAACTGACCCGGGTACATAGCACCCCTAGCCCCTACAAAGGACTAGATCTCTTAAAACTACATGAAACCCTCCATACCCATACTTGCCTGGTAAGCCTATTTAATACCACCCTCACTGGGCTCCATGAGGTCTCGGCCCAAAACCCTACTAACTGTTGGATGTGCCTCCCCCTGTATTTCAGGCCATGCATTTCAATCCCTGTACCTGAACAATGGAACAACTACAGCACAGAAATAAACACCACTTCCGTTTTAGTAGGACCTCTTGTTTCCAATCTGGAAATAACCCATACCTCAAACCTCACCTGTGTAAAATTTAGCAATACTGTAGACACAACCAACTCCCAATGCATCAGGTGGGTAACTCCCCCCACGCGAATAGTCTGCCTACCCTCAGGAATATTTTTTGTCTGTGGTACCTC

>GL-1-K16_G09

TTCACTGCCCACACCCATATGCCCCACAACTGCTATAACTCTGCCACTCTTTGCATGCATGCAAATACTCATTATTGGACAGGGAAAATGATTAATCCTAGTTGTCCTGGAAGACTTGGAGCCACTGTCTGTCGGACTTACTTCACCCATACTGGTATGTCTGAGGGGGGTGGAGTTCAAGATCAGGCAAGAGAAAAACATGTAAAGGAAGTAACCTCCCAACTGACCCGGGTACATAGCACCCCTAGCCCCTACAAAGGACTAGATCTCTTAAAACTACATGAAACCCTCCATACCCATACTTGCCTGGTAAGCCTATTTAATACCACCCTGACTGGGCTCCATGAGGTCTCGGCCCAAAACCCTACTAACTGTTGGATGTGCCTCCCCCTGCACTTTAGGCCATACATTTCAATCCCTATACCTGAACAATGGAACAACTTCAGCACAGAAATAAACACCACTTCTGTTTTAGTAGGTCCTCTTTCCAATCTGGAAATAACCCATACCTCAAACCTCACCTGTGTAAAATTTAGCAATACTATAGACACAGCCAACCCCCAATGCATCAGGTGGGTAACTCCTCCCACACGAATAGTCTGCCTACCCTCAGGAATATTTTTTGTCTGTGGTACCTC

>GL-1-K17_H09

TTCACTGCCCACACCCATATGCCCCACAACTGCTATAACTCTGCCACTCTTTGCATGCATGCAAATACTCATTATTGGACAGGGAAAATGATTAATCCTAGTTGTCCTGGAAGACTTGGAGCCACTGTCTGTCGGACTTACTTCACCCATACTGGTATGTCTGAGGGGGGTGGAGTTCAAGATCAGGCAAGAGAAAAACATGTAAAGGAAGTAACCTCCCAACTGACCCGGGTACATAGCACCCCTAGCCCCTACAAAGGACTAGATCTCTTAAAACTACATGGAACCCTCCATACCCATACTTGCCTGGTAAGCCTATTTAATACCACCCTCACTGGGCTCCATGAGGTCTCGGCCCAAAACCCTACTAACTGTTGGATGTGCCTCCCCCTGTATTTCAGGCCATGCATTTCAATCCCTGTACCTGAACAATGGAACAACTACAGCACAGAAATAAACACCACTTCCGTTTTAGTAGGACCTCTTGTTTCCAATCTGGAAATAACCCATACCTCAAACCTCACCTGTGTAAAATTTAGCAATACTGTAGACACAACCAACTCCCAATGCATCAGGTGGGTAACTCCTCCCACACGAATAGTCTGCCTACCCTCAGGAATATTTTTTGTCTGTGGTACCTC

>GL-1-K19_A10

GAGGTACCACAGACAAAAAATATTCCTGAGGGTAGGCAGACTATTCGTGGGGGGGAGTTACCCACCTGATGTATTGGGAGTTGGTTGTGTCTATCGTATTGCTAAATTTTACACAGGTGAGGTTTGAGGTATGGGTTATTTCCAGATTGGAAACAAGAGGTCCTACTAAAACGGAAGTGGTGTTTATGTGTTGAAGTTGTTCCCTTGTTCAGGTACAGGGATTGAAATGTATGGCCTTAAGTGCAGAGGGAGGCACATCCAACAGTTAGTAGGGTTTTGGGCCGAGACCTCATGAAACCCAGTGAGGGTGGTATTAAATAGGCTTACCAGGCGAGTATGGGTACGGAGGGTTTCATGTAGTTTTGAGAGATCTAATCCTTTGTAGGGGCTAGGCGTGCTATGTACTCAGGTCAGTTGGGAGATTACTTCCTTTACGTGTTTTTCTCTTGCCTGATCTTGAACTCCACCCCTATCACACATACCAGTATGGGTGAACTAAGTCCAACAGACAGTGGCTCCAAGTCCTCCAGGACAACTAGGATTAATCATTTTCCCTGTCCAATAATGAGTATTTGCATGCATGCAAAGAGTGGCAGAGTTATAGCAGTTGTGGGGCATATGGGTGTGGGCAGTGAA

>GL-1-K20_B10

TTCACTGCCCACACCCATATGCCCCACAACTGCTATAACTCTGCCACTCTTTGCATGCATGCAAATACTCATTATTGGACAGGAAAAACGATTAATCCCAGTTGTCCTGGAGGACTTGGAGGACTCACTTCACTCATACCAGTATGTCTGATGGGGGTGGAGTTCAAGATCAGGCAACAGAAAAACACATAAAGGAAGTAATCTCCCAACTGACCTGGGTACATAGCACCCCTGGCCCCTACAAAGGACTAGATCTCTCAAAACTACATGAAACCCTCCATACCCATACTGGCCTGGTAAGCCTATTTAATACCACCCTGACTGGGCTCCATGAGGTCTCGGCCCAAAACCCTACTAACTGTTGGATGTGCCTCCCCCTGCACTTCAGGCCATACATTTCAATCCCTATACCTGAACAATGGAACAACTTCAGCACAGAAATAAACACCACTTCTGTTTTAGTAGGTCCTCTTTCCAATCTGGAAATAACCCATACCTCAAACCTCACCTGTGTAAAATTTAGCAATACTATAGACACAGCCAACTCCCAATGCATCAGGTGGGTAACTCCTCCCACACGAATAGTCTGCCTACCCTCAGGAATATTTTTTGTCTGTGGTACCTC

>GL-1-K21_C10

GAGGTACCACAGACAAAAAATATTCCTGAGGGTAGGCAGACTATTCGTGTGGGAGGAGTTACCCACCTGATGCATTGGGAGTTGGCTGTGTCTATAGTATTGCTAAATTTTACACAGGTGAGGTTTGAGGTATGGGTTATTTCCAGATTGGAAAGAGGACCTACTAAAACAGAAGTGGTGTTTATTTCTGTGCTGAAGTTGTTCCATTGTTCAGGTATAGGGATTGAAATGTATGGCCTAAAGTGCAGGGGGAGGCACATCCAACAGTTAGTAGGGTTTTGGGCCGAGACCTCATGGAGCCCAGTCAGGGTGGTATTAAATAGGCTTACCAGGCCAGTATGGGTATGGAGGGTTTCATGTAGTTTTGAGAGATCTAGTCCTTTGTAGGGGCCAGGGGTGCTATGTACCCAGGTCAGTTGGGGGATTACTTCCTTTATGTGTTTTTCTGTTGCCTGATCTTGAACTCCACCCCCATCAGACATACTGGTATGAGTGAAGTGAGTCCTCCAAGTCCTCCAGGACAACTGGGATTAATCGTTTTTCCTGTCCAATAATGAGTATTTGCATGCATGCAAAGAGTGGCAGAGTTATAGCAGTTGTGGGGCATATGGGTGTGGGCAGTGAA

>GL-1-K22_D10

GAGGTACCACAGACAAAAAATATTCCTGAGGGTAGGCGGACTATTTGTGTGGGAGGAGTTACCCACCTGATGCATTGGGAGTTGGTTGTGTATGTAGTATTGCTAAATTTTACACAGGTGAGGTTTGAGGTATGGGTTATTTCCAGATTGGAAACAAGAGGTCCTACTAAAACGGAAGTGGTGTTTATTTCTGTGCTGAAGTTGTTCCATTGTTCAGGTACAGGGATTGAAACATATGGCCTGAAGTTCAGGGGGAGGCATATCCAACAGTTAGTAGGGTTTTGGGCCGAGACCTCATGGAGCCCAGTGAGGGTGGTATTAAATAGGCTTACCAGGCGAGTATGGGTACGGAGGGTTTCATGTAGTTTTGAGAGATCTAGTCCTTTGTAGGGGCTAGAGGTGCCATGTACCCGGGTGAGTTGGGAGATTACTTCTTTTACATGTTTTTCTCTTGCCTGATCTTGAACTCCACCCCCATCAGACATACCAGTTTGGGTGAAGTAAGTCCAACAGACAGTGACTCCAAGTCCTCCAGGACAACTAGGATTAATCACTTTTCCTGTCCAATAATGAGTATTTGCATGCATGCAAAGAGTGGCAGAGTGATAGCAGTTGCGGGGCATATGGGTGTGGGCAGTGAA

>GL-1-K26_T7_B05

GAGGTACCACAGACAAAAAATATTCCTGAGGGTAGGCAGACTATTCGTGTGGGAGGTGTTACCCACCTGATGCATTGGGAGCTGGTTGTGTCTATAGTATTGCTAAATTTTACACAGGTGAGGTTTGAGGTATGGGTTATTTCCAGATTGGAAACAAGAGGTCCTACTAAAACGGAAGTGGTGTTTATTTCTGTGCTGAAGTTGTTCCATTGTTCAGGAACAGGGATTGAAATGTATGGCCTGAAGTGCAGGGGGAGGCACATCCAACAGTTAGTAGGGTTTTGGGCTGAGACCTCATGGAGCCGAGTGAGGGTGGTATTAAATAGGCTCACCAGGCGAGTATGGGTACGGAGGGTTTCATGTAGTTTTGAGAGAACTAGTCCTTTGTAGGGGCTAGGGGTGCTATGTCCCCGGGTCAGTTGGGAGATTGCTTCCTTTACTTGTTTTTCTCTTGCCTGACCTTGAATTCCACCCCCATCAGACATACTGGTATGGGTGAAGTAAGTCCAACAGACAGTGGCTCCAAGTCCTCCAGGACAACTAGGATTAATCATTTTCCCTGTCCAATAATGAGTATTTGCATGCATGCAAAGAGTGGCAGAGTTATAGCAGTTGCGGGGCATATGGGTGTGGGCAGTGAA

>GL-1-K27_T7_C05

GAGGTACCACAGACAAAAAATATTCCTGAGGGTAGGCAGACTATTCGTGTGGGAGGTGTTACCCACCTGATGCATTGGGAGCTGGTTGTGTCTATAGTATTGCTAAATTTTACACAGGTGAGGTTTGAGGTATGGGTTATTTCCAGATTGGAAACAAGAGGTCCTACTAAAACGGAAGTGGTGTTTATTTCTGTGCTGAAGTTGTTCCATTGTTCAGGAACAGGGATTGAAATGTATGGCCTGAAGTGCAGGGGGAGGCACATCCAACAGTTAGTAGGGTTTTGGGCTGAGACCTCATGGAGCCGAGTGAGGGTGGTATTAAATAGGCTCACCAGGCGAGTATGGGTACGGAGGGTTTCATGTAGTTTTGAGAGAACTAGTCCTTTGTAGGGGCTAGGGGTGCTATGTCCCCGGGTCAGTTGGGAGATTGCTTCCTTTACTTGTTTTTCTCTTGCCTGACCTTGAATTCCACCCCCATCAGACATACTGGTATGGGTGAAGTAAGTCCAACAGACAGTGGCTCCAAGTCCTCCAGGACAACTAGGATTAATCATTTTCCCTGTCCAATAATGAGTATTTGCATGCATGCAAAGAGTGGCAGAGTTATAGCAGTTGCGGGGCATATGGGTGTGGGCAGTGAA

>GL-1-K29_T7_D05

GAGGTACCACAGACAAAAAATATTCCTGAGGGTAGGCAGACTATTCGTGGGGGGGAGTTACCCACCTGATGCATTGGGAGTTGGTTGTGTCTATCGTATTGCTAAATTTTACACAGGTGAGGTTTGAGGTATGGGTTATTTCCAGATTGGAAACAAGAGGTCCTACTAAAACGGAAGTGGTGTTTATGTGTTGAAGTTGTTCCCTTGTTCAGGTACAGGGATTGAAATGTATGGCCTTAAGTGCAGAGGGAGGCACATCCAACAGTTAGTAGGGTTTTGGGCCGAGACCTCATGAAACCCAGTGAGGGTGGTATTAAATAGGCTTACCAGGCGAGTATGGGTACGGAGGGTTTCATGTAGTTTTGAGAGATCTAGTCCTTTGTAGGGGCTAGGCGTGCTATGTACTCAGGTCAGTTGGGAGATTACTTCCTTTACGTGTTTTTCTCTTGCCTGATCTTGAACTCCACCCCTATCAGACATACCAGTATGGGTGAACTAAGTCCAACAGACAGTGGCTCCAAGTCCTCCAGGACAACTAGGATTAATCATTTTCCCTGTCCAATAATGAGTATTTGCATGCATGCAAAGAGTGGCAGAGTTTATAGCAGTTGTGGGGCATATGGGTGTGGGCAGTGAA

>GL-1-K30_T7_E05

GAGGTACCACAGACAAAAAATATTCCTGAGGGTAGGCAGACTATTTGTGTGGGAGGAGTTACCCACCTGATGCATTGGGAGTTGGTTGTGTATGTAGTATTGCTAAATTTTACACAGGTGAGGTTTGAGGTATGGGTTATTTCCAGATTGGAAAGAGGACCTACTAAAACAGAAGTGGTGTTTATTTCTGTGCTGAAGTTGTTCCATTGTTCAGGTATAGGGATTGAAATGTATGGCCTAAAGTGCAGGGGGAGGCACATCCAACAGTTAGTAGGGTTTTGGGCCGAGACCTCATGGAGCCCAGTCAGGGTGGTATTAAATAGGCTTACCAGGCCAGTATGGGTATGGAGGGTTTCATGTAGTTTTGAGAGATCTAGTCCTTTGTAGGGGCCAGGGGTGCTATGTACCCAGGTCAGTTGGGAGATTACTTCCTTTATGTGTTTTTCTGTTGCCTGATCTTGAACTCCACCCCCATCAGACATACTGGTATGAGTGAAGTGAGTCCTCCAAGTCCTCCAGGACAACTGGGATTAATCGTTTTTCCTGTCCAATAATGAGTATTTGCATGCATGCAAAGAGTGGCAGAGTTATAGCAGTTGTGGGGCATATGGGTGTGGGCAGTGAA

>GL-1-K31_T7_F05

TTCACTGCCCACACCCATATGCCCCACAACTGCTATAACTCTGCCACTCTTTGCATGCATGCAAATACTCATTATTGGACAGGGAAAATGATTAATCCTAGTTGTCCTGGAAGACTTGGAGCCACTGTCTGTCGGACTTACTTCACCCATACTGGTATGTCTGAGGGGGGTGGAGTTCAAGATCAGGCAAGAGAAAAACATGTAAAGGAAGTAACCTCCCAACTGACCCGGGTACATAGCACCCCTAGCCCCTACAAAGGACTAGATCTCTTAAAACTACATGAAACCCTCCATACCCATACTTGCCTGGTAAGCCTATTTAATACCACCCTCACTGGGCTCCATGAGGTCTCGGCCCAAAACCCTACTAACTGTTGGATGTGCCTCCCCCTGTATTTCAGGCCATGCATTTCAATCCCTGTACCTGAACAATGGAACAACTACAGTACAGAAATAAACACCACTTCCGTTTTAGTAGGACCTCTTGTTTCCAATCTGGAAATAACCCATACCTCAAACCTCACCTGTGTAAAATTTAGCAATACTGTAGACACAACCAACTCCCAATGCATCAGGTGGGTAACTCCTCCCACACGAATAGTCTGCCTACCCTCAGGAATATTTTTTGTCTGTGGTACCTC

>GL-1-K32_T7_G05

GAGGTACCACAGACAAAAAATATTCCTGAGGGTAGGCAGACTATTCGTGTGGGAGGAGTTACCCACCTGATGCATTGGGAGTTGGTTGTGTCTACGGTATGGCTAAATTTTACACAGGTGAGGTTTGAGGTATGGGTTATTTCCAGATTGGAAACAAGAGGTCCTACTAAAACGGAAGTGGTGTTTATTTCTGTGCTGTAGTTGTTCCATTGTTCAGGTACAGGGATTGAAATGCATGGCCTGAAATACAGGGGGAGGCACATCCAACAGTTAGTAGGGTTTTGGGCCGAGACCTCATGGAGCCCAGTGAGGGTGGTATTAAATAGGCTTACCAGGCAAGTATGGGTATGGAGGGTTTCATGTAGTTTTAAGAGATCTAGTCCTTTGTAGGGGCTAGGGGTGCTATGTACCCGGGTCAGTTGGGAGGTTACTTCCTTTACATGTTTTTCTCTTGCCTGATCTTGAACTCCACCCCCCTCAGACATACCAGTATGGGTGAAGTAAGTCCGACAGACAGTGGCTCCAAGTCTTCCAGGACAACTAGGATTAATCATTTTCCCTGTCCAATAATGAGTATTTGCATGCATGCAAAGAGTGGCAGAGTTTATAGCAGTTGTGGGGCATATTGGGTGTGGGCAGTGAA

>Gl-1-K33_T7_H05

GAGGTACCACAGACAAAAAATATTCCTGAGGGTAGGCAGACTATTCGTGTGGGAGGAGTTACCCACCTGATGCATTGGGAGTTGGCTGTGTCTATAGTATTGCTAAATTTTACACAGGTGAGGTTTGAGGTATGGGTTATTTCCAGATTGGAAAGAGGACCTACTAAAACAGAAGTGGTGTTTATTTCTGTGCTGAAGTTGTTCCATTGTTCAGGTATAGGGATTGAAATGTATGGCCTAAAGTGCAGGGGGAGGCACATCCAACAGTTAGTAGGGTTTTGGGCCGAGACCTCATGGAGCCCAGTCAGGGTGGTATTAAATAGGCTTACCAGGCCAGTATGGGTATGGAGGGTTTCATGTAGTTTTGAGAGATCTAGTCCTTTGTAGGGGCCAGGGGTGCTATGTACCCAGGTCAGTTGGGAGATTACTTCCTTTATGTGTTTTTCTGTTGCCTGATCTTGAACTCCACCCCCATCAGACATACTGGTATGAGTGAAGTGAGTCCTCCAAGTCCTCCAGGACAACTGGGATTAATCGTTTTTCCTGTCCAATAATGAGTATTTGCATGCATGCAAAGGGTGGCATGATTTTATAGCA

>GL-1-K34_T7_A06

TTCACTGCCCACACCCATATGCCCCGCAACTGCTATAACTCTGCCACTCTTTGCATGCATGCAAATACTCATTATTGGACAGGGAAAATGATTAATCCTAGTTGTCCTGGAGGACTTGGAGCCACTGTCTGTTGGACTTACTTCACCCATACCAGTATGTCTGATGGGGGTGGAATTCAAGGTCAGGCAAGAGAAAAACAAGTAAAGGAAGCAATCTCCCAACTGACCCGGGGACATAGCACCCCTAGCCCCTACAAAGGACTAGTTCTCTCAAAACTACATGAAACCCTCCGTACCCATACTCGCCTGGTAAGCCTATTTAATACCACCCTCACTGGGCTCCATGAGGTCTCGGCCCAAAACCCTACTAACTGTTGGATATGCCTCCCCCTGAACTTCAGGCCATATGTTTCAATCCCTGTACCTGAACAATGGAACAACTTCAGCACAGAAATAAACACCACTTCCGTTTTAGTAGGACCTCTTGTTTCCAATCTGGAAATAACCCATACCTCAAACCTCACCTGTGTAAAATTTAGCAATACTACATACACAACCAACTCCCAATGCATCAGGTGGGTAACTCCTCCCACACAAATAGTCTGCCTACCCTCAGGAATATTTTTTGTCTGTGGTACCTC

>GL-1-K35_T7_B06

TTCACTGCCCACACCCATATGCCCCACAACTGCTATAACTCTGCCACTCTTTGCATGCATGCAAATACTCATTATTGGACAGGGAAAATGATTAATCCTAGTTGTCCTGGAAGACTTGGAGCCACTGTCTGTCGGACTTACTTCACCCATACTGGTATGTCTGAGGGGGGTGGAGTTCAAGATCAGGCAAGAGAAAAACATGTAAAGGAAGTAACCTCCCAACTGACCCGGGTACATAGCACCCCTAGCCCCTACAAAGGACTAGATCTCTTAAAACTACATGAAACCCTCCATACCCATACTTGCCTGGTAAGCCTATTTAATACCACCCTCACTGGGCTCCATGAGGTCTCGGCCCAAAACCCTACTAACTGTTGGATGTGCCTCCCCCTGTATTTCAGGCCATGCATTTCAATCCCTGTACCTGAACAATGGAACAACTACAGCACAGAAATAAACACCACTTCCGTTTTAGTAGGACCTCTTGTTTCCAATCTGGAAATAACCCATACCTCAAACCTCACCTGTGTAAAATTTAGCAATACTGTAGACACAACCAACTCCCAATGCATCAGGTGGGTAACTCCTCCCACACGAATAGTCTGCCTACCCTCAGGAATATTTTTTGTCTGTGGTACCTC

>GL-1-K36_T7_C06

GAGGTACCACAGACAAAAAATATTCCTGAGGGTAGGCAGACTATCCGTGGGGGGGAGTTACCCACCTGATGCATTGGGAGTTGGTTGTGTCTATCGTATTGCTAAATTTTACACAGGTGAGGTTTGAGGTATGGGTTATTTCCAGATTGGAAACAAGAGGTCCTACTAAAACGGAAGTGGTGTTTATGTGTTGAAGTTGTTCCCTTGTTCAGGTACAGGGATTGAAATGTATGGCCTTAAGTGCAGAGGGAGGCACATCCAACAGTTAGTAGGGTTTTGGGCCGAGACCTCATGAAACCCAGTGAGGGTGGTATTAAATAGGCTTACCAGGCGAGTATGGGTACGGAGGGTTTCATGTAGTTTTGAGAGATCTAGTCCTTTGTAGGGGCTAGGCGTGCTATGTACTCAGGTCAGTTGGGAGATTACTTCCTTTACGTGTTTTTCTCTTGCCTGATCTTGAACTCCACCCCTATCAGACATACCAGTATGGGTGAACTAAATCCAACAGACAGTGGCTCCAAGTCCTCCAGGACAACTAGGATTAATCATTTTCCCTGTCCAATAATGAGTATTTGCATGCATGCAAAGAGTGGCAGAGTTATAGCAGTTTGTGGGGCATATGGGTGTGGGCAGTGAA

>GL-1-K37_T7_D06

GAGGTACCACAGACAAAAAATATTCCTGAGGGTAGGCAGACCATTCGTGTGGGAGGAGTTACCCACCTGATGCATTGGGAGTTGGCTGTGTCTATAGTATTGCTAAATTTTACACAGGTGAGGTTTGAGGTATGGGTTATTTCCAGATTGGAAAGAGGACCTACTAAAACAGAAGTGGTGTTTATTTCTGTGCTGAAGTTGTTCCATTGTTCAGGTATAGGGATTGAAATGTATGGCCTAAAGTGCAGGGGGAGGCACATCCAACAGTTAGTAGGGTTCTGGGCCGAGACCTCATGGAGCCCAGTCAGGGTGGTATTAAATAGGCTTACCAGGCCAGTATGGGTATGGAGGGTTTCATGTAGTTTTGAGAGATCTAGTCCTTTGTAGGGGCCAGGGGTGCTATGTACCCAGGTCAGTTGGGAGATTACTTCCTTTATGTGTTTTTCTGTTGCCTGATCTTGAACTCCACCCCCATCAGACATACTGGTATGAGTGAAGTGAGTCCTCCAAGTCCTCCAGGACAACTGGGATTAATCGTTTTTTCCTGTCCAATAATGAGTATTTGCATGCATGCAAGAGTGGCAGAGTTATAGCAGTTGTGGGGCATATGGGTGTGGGCAGTGAA

>GL-1-K40_T7_E06

TTCACTGCCCACACCCATATGCCCCACAACTGCTATAACTCTGCCACTCTTTGCATGCATGCAAATACTCATTATTGGACAGGGAAAGTGATTAATCCTAGTTGTCCTGGAAGACTTGGAGCCACTGTCTGTCGGACTTACTTCACCCATACTGGTATGTCTGAGGGGGGTGGAGTTCAAGATCAGGCAAGAGAAAAACATGTAAAGGAAGTAACCTCCCAACTGACCCGGGTACATAGCACCCCTAGCCCCTACAAAGGACTAGATCTCTTAAAACTACATGAAACCCTCCATACCCATACTTGCCTGGTAAGCCTATTTAATACCACCCTGACTGGGCTCCATGAGGTCTCGGCCCAAAACCCTACTAACTGTTGGATGTGCCTCCCCCTGCACTTTAGGCCATACATTTCAATCCCTATACCTGAACAATGGAACAACTTCAGCACAGAAATAAACACCACTTCTGTTTTAGTAGGTCCTCTTTCCAATCTGGAAATAACCCATACCTCAAACCTCACCTGTGTAAAATTTAGCAATACTATAGACACAGCCAACTCCCAATGCATCAGGTGGGTAACTCCTCCCACACGAATAGTCTGCCTACCCTCAGGAATATTTTTTGTCTGTGGTACCTC

>GL-1-K41_T7_F06

GAGGTACCACAGACAAAAAATATTCCTGAGGGTAGGCAGACTATTCGTGTGGGAGGAGTTACCCACCTGATGCATTGGGAGTTGGTTGTGTCTACAGTATTGCTAAATTTTACACAGGTGAGGTTTGAGGTATGGGTTATTTCCAGATTGGAAACAAAAGGTCCTACTAAAACGGAAGTGGTGTTTATTTCTGTGCTGTATTTGTTCCATTGTTCAGGTACAGGGATTGAAATGCATGGCCTGAAATACAGGGGGAGGCACATCCAACATTTATTAGGGTTTTGGGCCGAAACCTCTTGGAGCCCAGTGAGGGTGGTATTAAATAGGCTTACCAGGCAAGTATGGGTATGGAGGGTTTCATGTAGTTTTAAGAGATCTATTCCTCTGTAGGGGCTAGGGGTGCTATGTACCCGGGTCATTTGGGAGGTTACTTCCTTTACATGTTTTTCTCTTGCCTGATCTTGAATTCCACCCCCCTC

>GL-1-K42_T7_G06

TTCACTGCCCACACCCATATGCCCCGCAACTGCTTTCACTCTGCCTCTCTTTGCATGCATGCAAATACTCATTATTGGACAGGAAAAATGATTAATCCTAGTTGTCCTGGAGGACTTGGAGTCACTGTCTGTTGGACTTACTTCACCCAAACTGGTATGTCTGATGGGGGTGGAGTTCAAGATCAGGCAAGAGAAAAACATGTAAAAGAAGTAATCTCCCAACTCACCCGGGTACATGGCACCCCTAGCCCCTACAAAGGACTAGATCTCTCAAAACTACATGAAACCCTCCGTACCCATACTCGCCTGGTGAGCCTATTTAATACCACCCTCACTCGGCTCCATGAGGTCTCAGCCCAAAACCCTACTAACTGTTGGATGTGCCTCCCCCTGCACTTCAGGCCATACATTTCAATCCCTGTTCCTGAACAATGGAACAACTTCAGCACAGAAATAAACACCACTTCCGTTTTAGTAGGACCTCTTGTTTCCAATCTGGAAATAACCCATACCTCAAACCTCACCTGTGTAAAACTTAGCAATACTATAGACACAATC

>GL-1-K49_T7_A07

GAGGTACCACAGACAAAAAATATTCCTGAGGGTAGGCAGACTATTCGTGTGGGAGGAGTTACCCACCTGATGCATTGGGAGTTGGCTGTGTCTATAGTATTGCTAAATTTTACACAGGTGAGGTTTGAGGTATGGGTTATTTCCAGATTGGAAAGAGGACCTACTAAAACAGAAGTGGTGTTTACTTCTGTGCTGAAGTTGTTCCATTGTTCAGGTATAGGGATTGAAATGTATGGCCTAAAGTGCAGGGGGAGGCACATCCAACAGTTAGTAGGGTTTTGGGCCGAGACCTCATGGAGCCCAGTCAGGGTGGTATTAAATAGGCTTACCAGGCCAGTATGGGTATGGAGGGTTTCATGTAGTTTTGAGAGATCTAGTCCTTTGTAGGGGCCAGGGGTGCTATGTACCCAGGTCAGTTGGGAGATTACTTCCTTTATGTGTTTTTCTGTTGCCTGATCTTGAACTCCACCCCCATCAGACATACTGGTATGAGTGAAGTGAGTCCTCCAAGTCCTCCAGGACAACTGGGATTAATCGTTTTTCCTGTCCAATAATGAGTATTTGCATGCATGCAAAGAGTGGCAGAGTTATAGCAGTTGTGGGGCATATGGGTGTGGGCAGTGAA

>GL-1-K51_T7_B07

TTCACTGCCCACACCCATATGCCCCACAACTGCTATAACTCTGCCACTCTTTGCATGCATGCAAATACTCATTATTGGACAGGGAAAATGATTAATCCTAGTTGTCCTGGAAGACTTGGAGCCACTGTCTGTCGGACTTACTTCACCCATACTGGTATGTCTGAGGGGGGTGGAGTTCAAGATCAGGCAAGAGAAAAACATGTAAAGGAAGTAACCTCCCAACTGACCCGGGTACATAGCACCCCTAGCCCCTACAAAGGACTAGATCTCTTAAAACTACATGAAACCCTCCATACCCATACTTGCCTGGTAAGCCTATTTAATACCACCCTCACTGGGCTCCATGAGGTCTCGGCCCAAAACCCTACTAACTGTTGGATGTGCCTCCCCCTGTATTTCAGGCCATGCATTTCAATCCCTGTACCTGAACAATGGAACAACTACAGCACAGAAATAAACACCACTTCCGTTTTAGTAGGACCTCTTGTTTCCAATCTGGAAATAACCCATACCTCAAACCTCACCTGTGTAAAATTTAGCAATACTGTAGACACAACCAACTCCCAATGCATCAGGTGGGTAACTCCTCCCACACGAATAGTCTGCCTACCCTCAGGAATATTTTTTGTCTGTGGTACCTC

>GL-1-K52_T7_C07

GAGGTACCACAGACAAAAAATATTCCTGAGGGTAGGCAGACTATTCGTGGGGGGGAGTTACCCACCTGATGCATTGGGAGTTGGTTGTGTCTATCGTATTGCTAAATTTTACACAGGTGAGGTTTGAGGTATGGGTTATTTCCAGATTGGAAACAAGAGGTCCTACTAAAACGGAAGTGGTGTTTATGTGTTGAAGTTGTTCCCTTGTTCAGGTACAGGGATTGAAATGTATGGCCTTAAGTGCAGAGGGAGGCACATCCAACAGTTAGTAGGGTTTTGGGCCGAGACCTCATGAAACCCAGTGAGGGTGGTATTAAATAGGCTTACCAGGCGAGTATGGGTACGGAGGGTTTCATGTAGTTTTGAGAGATCTAGTCCTTTGTAGGGGCTAGGCGTGCTATGTACTCAGGTCAGTTGGGAGATTACTTCCTTTACGTGTTTTTCTCTTGCCTGATCTTGAACTCCACCCCTATCAGACATACCAGTATGGGTGAACTAAGTCCAACAGACAGTGGCTCCAAGTCCTCCAGGACAACTAGGATTAATCATTTTCCCTGTCCAATAATGAGTATTTGCATGCATGCAAAGAGTGGCAGAGTTATAGCAGTTGTGGGGCATATGGGTGTGGGCAGTGAA

>GL-1-K53_T7_D07

GAGGTACCACAGACAAAAAATATTCCTGAGGGTAGGCAGACTATTCGTGGGGGGGAGTTACCCACCTGATGCATTGGGAGTTGGTTGTGTCTATCGTATTGCTAAATTTTACACAGGTGAGGTTTGAGGTATGGGTTATTTCCAGATTGGAAACAAGAGGTCCTACTAAAACGGAAGTGGTGTTTATGTGTTGAAGTTGTTCCCTTGTTCAGGTACAGGGATTGAAATGTATGGCCTTAAGTGCAGAGGGAGGCACATCCAACAGTTAGTAGGGTTTTGGGCCGAGACCTCATGAAACCCAGTGAGGGTGGTATTAAATAGGCTTACCAGGCGAGTATGGGTACGGAGGGTTTCATGTAGTTTTGAGAGATCTAGTCCTTTGTAGGGGCTAGGGGTGCTATGTCCCCGGGTCAGTTGGGAGATTGCTTCCTTTACTTGTTTTTCTCTTGCCTGACCTTGAATTCCACCCCCATCAGACATACTGGTATGAGTGAAGTAAGTCCAACAGACAGTGGCTCCAAGTCCTCCAGGACAACTAGGATTAATCATTTTCCCTGTCCAATAATGAGTATTTGCATGCATGCAAAGAGTGGCAGAGTTATAGCAGTTGCGGGGCATATGGGTGTGGGCAGTGAA

>GL-1-K54_T7_E07

TTCACTGCCCACACCCATATGCCCCACAACTGCTATAACTCTGCCACTCTTTGCATGCATGCAAATACTCATTATTGGACAGGAAAAACGATTAATCCCAGTTGTCCTGGAGGACTTGGAGGACTCACTTCACTCATACCAGTATGTCTGATGGGGGTGGAGTTCAAGATCAGGCAACAGAAAAACACATAAAGGAAGTAATCTCCCAACTGACCTGGGTACATAGCACCCCTGGCCCCTACAAAGGACTAGATCTCTCAAAACTACATGAAACCCTCCATACCCATACTGGCCTGGTAAGCCTATTTAATACCACCCTGACTGGGCTCCATGAGGTCTCGGCCCAAAACCCTACTAACTGTTGGATGTGCCTCCCCCTGCACTTTAGGCCATACATTTCAATCCCTATACCTGAACAATGGAACAACTTCAGCACAGAAATAAACACCACTTCTGTTTTAGTAGGTCCTCTTTCCAATCTGGAAATAACCCATACCTCAAACCTCACCTGTGTAAAATTTAGCAATACTATAGACACAGCCAACTCCCAATGCATCAGGTGGGTAACTCCTCCCACACGAATAGTCTGCCTACCCTCAGGAATATTTTTTGTCTGTGGTACCTC

>GL-1-K55_T7_F07

TTCACTGCCCACACCCATATGCCCCACAACTGCTATAACTCTGCCACTCTTTGCATGCATGCAAATACTCATTATTGGACAGGGAAAATGATTAATCCTAGTTGTCCTGGAAGACTTGGAGCCACTGTCTGTCGGACTTACTTCACCCATACTGGTATGTCTGAGGGGGGTGGAGTTCAAGATCAGGCAAGAGAAAAACATGTAAAGGAAGTAACCTCCCAACTGACCCGGGTACATAGCACCCCTAGCCCCTACAAAGGACTAGATCTCTTAAAACTACATGAAACCCTCCATACCCATACTTGCCTGGTAAGCCTATTTAATACCACCCTCACTGGGCTCCATGAGGTCTCGGCCCAAAACCCTACTAACTGTTGGATGTGCCTCCCCCTGTATTTCAGGCCATGCATTTCAATCCCTGTACCTGAACAATGGAACAACTACAGCACAGAAATAAACACCACTTCCGTTTTAGTAGGACCTCTTGTTTCCAATCTGGAAATAACCCATACCTCAAACCTCACCTGTGTAAAATTTAGCAATACTGTAGACACAACCAACTCCCAATGCATCAGGTGGGTAACTCCTCCCACACGAATAGTCTGCCTACCCTCAGGAATATTTTTTGTCTGTGGTACCTC

>GL-1-K57_T7_G07

TTCACTGCCCACACCCATATGCCCCACAACTGCTATAACTCTGCCACTCTTTGCATGCATGCAAATACTCATTATTGGACAGGGAAAATGATTAATCCTAGTTGTCCTGGAAGACTTGGAGCCACTGTCTGTCGGACTTACTCCACCCATACTGGTATGTCTGAGGGGGGTGGAGTTCAAGATCAGGCAAGAGAAAAACATGTAAAGGAAGTAACCTCCCAACTGACCCGGGTACATAGCACCCCTAGCCCCTACAAAGGACTAGATCTCTTAAAACTACATGAAACCCTCCATACCCATACTTGCCTGGTAAGCCTATTTAATACCACCCTCACTGGGCTCCATGAGGTCTCGGCCCAAAACCCTACTAACTGTTGGATGTGCCTCCCCCTGTATTTCAGGCCATGCATTTCAATCCCTGTACCTGAACAATGGAACAACTACAGCACAGAAATAAACACCACTTCCGTTTTAGTAGGACCTCTTGTTTCCAATCTGGAAATAACCCATACCTCAAACCTCACCTGTGTAAAATTTAGCAATACTGTAGACACAACCAACTCCCAATGCATCAGGTGGGTAACTCCTCCCACACGAATAGTCTGCCTACCCTCAGGAATATTTTTTGTCTGTGGTACCTC

>GL-1-K62_T7_H07

GAGGTACCACAGACAAAAAATATTCCTGAGGGTAGGCAGACTATTCGTGGGGGGGAGTTACCCACCTGATGCATTGGGAGTTGGTTGTGTCTATCGTATTGCTAAATTTTACACAGGTGAGGTTTGAGGTATGGGTTATTTCCAGATTGGAAACAAGAGGTCCTACTAAAACGGAAGTGGTGTTTATGTGTTGAAGTTGTTCCCTTGTTCAGGTACAGGGATTGAAATGTATGGCCTTAAGTGCAGAGGGAGGCACATCCAACAGTTAGTAGGGTTTTGGGCCGAGACCTCATGAAACCCAGTGAGGGTGGTATTAAATAGGCTTACCAGGCGAGTATGGGTACGGAGGGTTTCATGTAGTTTTGAGAGATCTAGTCCTTTGTAGGGGCTAGGCGTGCTATGTACTCAGGTCAGTTGGGAGATTACTTCCTTTACGTGTTTTTCTCTTGCCTGATCTTGAACTCCACCCCTATCAGACATACCAGTATGGGTGAACTAAGTCCAACAGACAGTGGCTCCAAGTCCTCCAGGACAACTAGGATTAATCATTTTCCCTGTCCAATAATGAGTATTTGCATGCATGCAAAGAGTGGCAGAGTTATAGCAGTTGTGGGGCATATGGGTGTGGGCAGTGAA

>GL-1-K64_T7_A08

GAGGTACCACAGACAAAAAATATTCCTGAGGGTAGGCAGACTATTCGTGGGGGGGAGTTACCCACCTGATGCATTGGGAGTTGGTTGTGTCTATCGTATTGCTAAATTTTACACAGGTGAGGTTTGAGGTATGGGTTATTTCCAGATTGGAAACAAGAGGTCCTACTAAAACGGAAGTGGTGTTTATGTGTTGAAGTTGTTCCCTTGTTCAGGTACAGGGATTGAAATGTATGGCCTAAAGTGCAGGGGGAGGCACATCCAACAGTTAGTAGGGTTTTGGGCCGAGACCTCATGGAGCCCAGTCAGGGTGGTATTAAATAGGCTTACCAGGCCAGTATGGGTATGGAGGGTTTCATGTAGTTTTGAGAGATCTAGTCCTTTGTAGGGGCCAGGGGTGCTATGTACCCAGGTCAGTTGGGAGATTACTTCCTTTATGTGTTTTTCTGTTGCCTGATCTTGAACTCCACCCCCATCAGACATACTGGTATGAGTGAAGTGAGTCCTCCAAGTCCTCCAGGACAACTGGGATTAATCGTTTTTCCTGTCCAATAATGAGTATTTGCATGCATGCAAAGAGTGGCAGAGTTATAGCAGTTGTGGGGCATATGGGTGTGGGCAGTGAA

>GL-1-K70_T7_B08

GAGGTACCACAGACAAAAAATATTCCTGAGGGTAGGCAGACTATTCGTGGGGGGGAGTTACCCACCTGATGCATTGGGAGTTGGTTGTGTCTATCGTATTGCTAAATTTTACACAGGTGAGGTTTGAGGTATGGGTTATTTCCAGATTGGAAACAAGAGGTCCTACTAAAACGGAAGTGGTGTTTATGTGTTGAAGTTGTTCCCTTGTTCAGGTACAGGGATTGAAATGTATGGCCTTAAGTGCAGAGGGAGGCACATCCAACAGTTAGTAGGGTTTTGGGCCGAGACCTCATGAAACCCAGTGAGGGTGGTATTAAATAGGCTTACCAGGCGAGTATGGGTACGGAGGGTTTCATGTAGTTTTGAGAGATCTAGTCCTTTGTAGGGGCTAGGCGTGCTATGTACTCAGGTCAGTTGGGAGATTACTTCCTTTACGTGTTTTTCTCTTGCCTGATCTTGAACTCCACCCCTATCAGACATACCAGTATGGGTGAACTAAGTCCAACAGACAGTGGCTCCAAGTCCTCCAGGACAACTAGGATTAATCATTTTCCCTGTCCAATAATGAGTATTTGCATGCATGCAAAGAGTGGCAGAGTTATAGCAGTTGTGGGGCATATGGGTGTGGGCAGTGAA

**MS-II**

>GL-12-K1_T7_D03

GAGGTACCACAGACAAAAAATATTCCTGAGGGTAGGCAGACTATTCGTGTGGGAGGTGTTACCCACCTGATGCATTGGGAGCTGGTTGTGTCTATAGTATTGCTAAATTTTACACAGGTGAGGTTTGAGGTATGGGTTATTTCCAGATTGGAAACAAGAGGTCCTACTAAAACGGAAGTGGTGTTTATGTGTTGAAGTTGTTCCCTTGTTCAGGTACAGGGATTGAAATGTATGGCCTTAAGTGCAGAGGGAGGCACATCCAACAGTTAGTAGGGTTTTGGGCCGAGACCTCATGAAACCCAGTGAGGGTGGTATTAAATAGGCTTACCAGGCGAGTATGGGTACGGAGGGTTTCATGTAGTTTTGAGAGATCTAGTCCTTTGTAGGGGCTAGGCGTGCTATGTACTCAGGTCAGTTGGGAGATTACTTCCTTTACTTGTTTTTCTCTTGCCTGACCTTGAATTCCACCCCCATCAGACATACTGGTATGGGTGAAGTAAGTCCAACAGACAGTGGCTCCAAGTCCTCCAGGACAACTAGGATTAATCATTTTCCCTGTCCAATAATGAGTATTTGCATGCATGCAAAGAGTGGCAGAGTTATAGCAGTTGCGGGGCATATGGGTGTGGGCAGTGAA

>GL-12-K2_T7_E03

CTGAGGTACCACAGACAAAAAATATTCCTGAGGGTAGGCAGACTATTCGTGTGGGAGGTGTTACCCACCTGATGCATTGGGAGCTGGTTGTGTCTATAGTATTGCTAAATTTTACACAGGTGAGGTTTGAGGTATGGGTTATTTCCAGATTGGAAACAAGAGGTCCTACTAAAACGGAAGTGGTGTTTATTTCTGTGCTGAAGTTGTTCCATTGTTCAGGAACAGGGATTGAAATGTATGGCCTGAAGTGCAGGGGGAGGCACATCCAACAGTTAGTAGGGTTTTGGGCTGAGACCTCATGGAGCCGAGTGAGGGTGGTATTAAATAGGCTTACCAGGCGAGTATGGGTACGGAGGGTTTCATGTAGTTTTGAGAGAACTAGTCCTTTGTAGGGGCTAGGGGTGCTATGTCCCCGGGTCAGTTGGGAGATTGCTTCCTTTACTTGTTTTTCTCTTGCCTGACCTTGAATTCCACCCCCATCAGACATACTGGTATGGGTGAAGTAAGTCCAACAGACAGTGGCTCCAAGTCCTCCAGGACAACTAGGATTAATCATTTTCCCTGTCCAATAATGAGTATTTGCATGCATGCAAAGAGTGGCAGAGTTATAGCAGTTGTGGGGCATATGGGTGTGGGCAGTGAA

>GL-12_K3_T7_F03

GAGGTACCACAGACAAAAAATATTCCTGAGGGTAGGCAGACTATTCGTGGGGGGGAGTTACCCACCTGATGCATTGGGAGTTGGTTGTGTCTATCGTATTGCTAAATTTTACACAGGTGAGGTTTGAGGTATGGGTTATTTCCAGATTGGAAACAAGAGGTCCTACTAAAACGGAAGTGGTGTTTATTTCTGTGCTGTAGTTGTTCCATTGTTCAGGTACAGGGATTGAAATGCATGGCCTGAAATACAGGGGGAGGCACATCCAACAGTTAGTAGGGTTTTGGGCCGAGACCTCATGGAGCCCAGTGAGGGTGGTATTAAATAGGCTTACCAGGCAAGTATGGGTATGGAGGGTTTCATGTAGTTTTAAGAGATCTAGTCCTTTGTAGGGGCTAGGGGTGCTATGTACCCGGGTCAGTTGGGAGGTTACTTCCTTTACATGTTTTTCTCTTGCCTGATCTTGAACTCCACCCCCCTCAGACATACCAGTATGGGTGAAGTAAGTCCGACAGACAGTGGCTCCAAGTCTTCCAGGACAACTAGGATTAATCATTTTCCCTGTCCAATAATGAGTATTTGCATGCATGCAAAGAGTGGCAGAGTTATAGCAGTTGCGGGGCATATGGGTGTGGGCAGTGAA

>GL-12_K4_T7_G03

GAGGTACCACAGACAAAAAATATTCCTGAGGGTAGGCAGACTATTCGTGTGGGAGGAGTTACCCACCTGATGCATTGGGAGTTGGTTGTGTCTATAGTATTGCTAAATTTTACACAGGTGAGGTTTGAGGTATGGGTTATTTCCAGATTGGAAAGAGGACCTACTAAAACAGAAGTGGTGTTTATTTCTGTGCTGAAGTTGTTCCATTGTTCAGGTATAGGGATTGAAATGTGTGGCCTAAAGTGCAGGGGGAGGCACATCCAACAGTTAGTAGGGTTTTGGGCCGAGACCTCATGGAGCCCAGTCAGGGTGGTATTAAATAGGCTTACCAGGCCAGTATGGGTATGGAGGGTTTCATGTAGTTTTGAGAGATCTAGTCCTTTGTAGGGGCCAGGGGTGCTATGTACCCAGGTCAGTTGGGAGATTACTTCCTTTATGTGTTTTTCTGTTGCCTGATCTTGAACTCCACCCCCATCAGACATACTGGTATGAGTGAAGTGAGTCCTCCAAGTCCTCCAGGACAACTGGGATTAATCGTTTTTCCTGTCCAATAATGAGTATTTGCATGCATGCAAAGAGTGGCAGAGTTATAGCAGTTGTGGGGCATATGGGTGTGGGCAGTGAA

>GL-12_K5_T7_H03

GAGGTACCACAGACAAAAAATATTCCTGAGGGTAGGCAGACTATTCGTGTGGGAGGAGTTACCCACCTGATGCATTGGGAGTTGGTTGTGTCTACAGTATTGCTAAATTTTACACAGGTGAGGTTTGAGGTATGGGTTATTTCCAGATCGGAAACAAGAGGTCCTACTAAAACGGAAGTGGTGTTTATGTGTTGAAGTTGTTCCCTTGTTCAGGTACAGGGATTGAAATGTATGGCCTTAAGTGCAGAGGGAGGCACATCCAACAGTTAGTAGGGTTTTGGGCCGAGACCTCATGAAACCCAGTGAGGGTGGTATTAAATAGGCTTACCAGGCGAGTATGGGTACGGAGGGTTTCATGTAGTTTTGAGAGATCTAGTCCTTTGTAGGGGCTAGGCGTGCTATGTACTCAGGTCAGTTGGGAGATTACTTCCTTTACATGTTTTTCTCTTGCCTGATCTTGAACTCCACCCCCCTCAGACATACCAGTATGGGTGAAGTAAGTCCGACAGACAGTGGCTCCAAGTCTTCCAGGACAACTAGGATTAATCATTTTCCCTGTCCAATAATGAGTATTTGCATGCATGCAAAGAGTGGCAGAGTTATAGCAGTTGCGGGGCATATGGGTGTGGGCAGTGAA

>GL-12_K6_T7_A04

TTCACTGCCCACACCCATATGCCCCGCAACTGCTATAACTCTGCCACTCTTTGCATGCATGCAAATACTCATTATTGGACAGGGAAAATGATTAATCCTAGTTGTCCTGGAGGACTTGGAGCCACTGTCTGTTGGACTTACTTCACCCATACCAGTATGTCTGATGGGGGTGGAATTCAAGGTCAGGCAAGAGAAAAACAAGTAAAGGAAGCAATCTCCCAACTGACCCGGGGACATAGCACCCCTAGCCCCTACAAAGGACTAGTTCTCTCAAAACTACATGAAACCCTCCGTACCCATACTCGCCTGGTGAGCCTATTTAATACCACCCTCACTCGGCTCCATGAGGTCTCAGCCCAAAACCCTACTAACTGTTGGATGTGCCTCCCCCTGCACTTCAGGCCATACATTTCAATCCCTGTTCCTGAACAATGGAACAACTTCAGCACAGAAATAAACACCACTTCCGTTTTAGTAGGACCTCTTGTTTCCAATCTGGAAATAACCCATACCTCAAACCTCACCTGTGTAAAATTTAGCAATACTATAGACACAACCAGCTCCCAATGCATCAGGTGGGTAACACCTCCCACACGAATAGTCTGCCTACCCTCAGGAATATTTTTTGTCTGTGGTACCTC

>GL-12_K8_T7_B04

GAGGTACCACAGACAAAAAATATTCCTGAGGGTAGGCAGACTATTCGTGTGGGAGGTGTTACCCACCTGATGCATTGGGAGCTGGTTGTGTCTATAGTATTGCTAAATTTTACACAGGTGAGGTTTGAGGTATGGGTTATTTCCAGATTGGAAACAAGAGGTCCTACTAAAACGGAAGTGGTGTTTATGTGTTGAAGTTGTTCCCTTGTTCAGGTACAGGGATTGAAATGTATGGCCTTAAGTGCAGAGGGAGGCACATCCAACAGTTAGTAGGGTTTTGGGCCGAGACCTCATGAAACCCAGTGAGGGTGGTATTAAATAGGCTTACCAGGCGAGTATGGGTACGGAGGGTTTCATGTAGTTTTGAGAGATCTAGTCCTTTGTAGGGGCTAGGCGTGCTATGTACTCAGGTCAGTTGGGAGATTACTTCCTTTACGTGTTTTTCTCTTGCCTGATCTTGAACTCCACCCCTATCAGACATACCAGTATGGGTGAACTAAGTCCAACAGACAGTGGCTCCAAGTCCTCCAGGACAACTAGGATTAATCATTTTCCCTGTCCAATAATGAGTATTTGCATGCATGCAAAGAGTGGCAGAGTTATAGCAGTTGTGGGGCATATGGGTGTGGGCAGTGAA

>GL-12_K9_T7_C04

GAGGTACCACAGACAAAAAATATTCCTGAGGGTAGGCAGACTATTTGTGTGGGAGGAGTTACCCATCTGATGCACTGGGAGTTGGTTGTGTCTATAGTATTGCTAAATTTTACACGGGTGAGGTTTGAGGTATGGGTTATTTCCAGATTGGAAACAAGAGGTCCTACTAAAATGGAAGTGGTGTTTATTTCTGTGCTGAAGTTGTTCCATTGTTCAGGAACAGGGATTGAAATGTATGGCCTGAAGTGCAGGGGGAGGCACATCCAACAGTTAGTAGGGTTTTGGGCCGAGACCTCATGAAACCCAGTGAGGGTGGTATTAAATAGGCTTACCAGGCGAGTATGGGTACGGAGGGTTTCATGTAGTTTTGAGAGATCTAGTCCTTTGTAGGGGCTAGGCGTGCTATGTACTCAGGTCAGTTGGGAGATTACTTCCTTTACGTGTTTTTCTCTTGCCTGATCTTGAACTCCACCCCTATCAGACATACCAGTATGGGTGAACTAAGTCCAACAGACAGTGGCTCCAAGTCCTCCAGGACAACTAGGATTAATCATTTTCCCTGTCCAATAATGAGTATTTGCATGCATGCAAAGAGTGGCAGAGTTATAGCAGTTGTGGGGCATATGGGTGTGGGCAGTGAA

>GL-12_K11_T7_D04

GAGGTACCACAGACAAAAAATATTCCTGAGGGTAGGCAGACTATTCGTGGGGGGGAGTTACCCACCTGATGCATTGGGAGTTGGTTGTGTCTATCGTATTGCTAAATTTTACACAGGTGAGGTTTGAGGTATGGGTTATTTCCAGATTGGAAACAAGAGGTCCTACTAAAACGGAAGTGGTGTTTATGTGTTGAAGTTGTTCCCTTGTTCAGGTACAGGGATTGAAATGTATGGCCTTAAGTGCAGAGGGAGGCACATCCAACAGTTAGTAGGGTTTTGGGCCGAGACCTCATGAAACCCAGTGAGGGTGGTATTAAATAGGCTTACCAGGCGAGTATGGGTACGGAGGGTTTCATGCAGTTTTGAGAGATCTAGTCCTTTGTAGGGGCTAGGCGTGCTATGTACTCAGGTCAGTTGGGAGATTACTTCCTTTACGTGTTTTTCTCTTGCCTGATCTTGAACTCCACCCCTATCAGACATACCAGTATGGGTGAACTAAGTCCAACAGACAGTGGCTCCAAGTCCTCCAGGACAACTAGGATTAATCATTTTCCCTGTCCAATAATGAGTATTTGCATGCATGCAAAGAGTGGCAGAGTTATAGCAGTTGTGGGGCATATGGGTGTGGGCAGTGAA

>GL-12_K12_T7_E04

TTCACTGCCCACACCCATATGCCCCACAACTGCTATAACTCTGCCACTCTTTGCATGCATGCAAATACTCATTATTGGACAGGGAAAATGATTAATCCTAGTTGTCCTGGAAGACTTGGAGCCACTGTCTGTCGGACTTACTTCACCCATACTGGTATGTCTGAGGGGGGTGGAGTTCAAGATCAGGCAAGAGAAAAACATGTAAAGGAAGTAATCTCCCAACTGACCCGGGTACATAGCACCCCTAGCCCCTACAAAGGACTAGATCTCCTAAAACTACATGAAACCCTCCGTACCCATACTCGCCTGGTAAGCCTATTTAATACCACCCTCACTGGGTTTCATGAGGTCTCGGCCCAAAACCCTACTAACTGTTGGATGTGCCTCCCTCTGCACTTAAGGCCATACATTTCAATCCCTGTACCTGAACAAGGGAACAACTTCAACACATAAACACCACTTCCGTTTTAGTAGGACCTCTTGTTTCCAATCTGGAAATAACCCATACCTCAAACCTCACCTGTGTAAAATTTAGCAATACGATAGACACAACCAACTCCCAATGCATCAGGTGGGTAACTCCCCCCCACGAATAGTCTGCCTACCCTCAGGAATATTTTTTGTCTGTGGTACCTC

>GL-12_K13_T7_F04

GAGGTACCACAGACAAAAAATATTCCTGAGGGTAGGCAGACTATTTGTGTGGGAGGAGTTACCCACCTGATGCATTGGGAGTTGGTTGTGTATGTAGTATTGCTAAATTTTACACAGGTGAGGTTTGAGGTATGGGTTATTTCCAGATTGGAAACAAGAGGTCCTACTAAAACGGAAGTGGTGTTTATTTCTGTGCTGAAGTTGTTCCATTGTTCAGGTACAGGGATTGAAACATATGGCCTGAAGTTCAGGGGGAGGCATATCCAACAGTTAGTAGGGTTTTGGGCCGAGACCTCATGAAACCCAGTGAGGGTGGTATTAAATAGGCTTACCAGGCGAGTATGGGTACGGAGGGTTTCATGTAGTTTTGAGAGATCTAGTCCTTTGTAGGGGCTAGGCGTGCTATGTACTCAGGTCAGTTGGGAGATTACTTCCTTTACGTGTTTTTCTCTTGCCTGATCTTGAACTCCACCCCTATCAGACATACCAGTATGGGTGAACTAAGTCCAACAGACAGTGGCTCCAAGTCCTCCAGGACAACTAGGATTAATCATTTTCCCTGTCCAATAATGAGTATTTGCATGCATGCAAAGAGTGGCAGAGTTATAGCAGTTGTGGGGCATATGGGTGTGGGCAGTGAA

>GL-12_K14_T7_G04

GAGGTACCACAGACAAAAAATATTCCTGAGGGTAGGCAGACTATTCGTGTGGGAGGAGTTACCCACCTGATGCATTGGGAGTTGGTTGTGTCTACAGTATTGCTAAATTTTACACAGGTGAGGTTTGAGGTATGGGTTATTTCCAGATTGGAAACAAGAGGTCCTACTAAAACGGAAGTGGTGTTTATGTGTTGAAGTTGTTCCCTTGTTCAGGTACAGGGATTGAAATGTATGGCCTTAAGTGCAGAGGGAGGCACATCCAACAGTTAGTAGGGTTTTGGGCCGAGACCTCATGAAACCCAGTGAGGGTGGTATTAAATAGGCTTACCAGGCGAGTATGGGTACGGAGGGTTTCATGTAGTTTTGAGAGATCTAGTCCTTTGTAGGGGCTAGGCGTGCTATGTACTCAGGTCAGTTGGGAGATTACTTCCTTTACGTGTTTTTCTCTTGCCTGATCTTGAACTCCACCCCTATCAGACATACCAGTATGGGTGAACTAAGTCCAACAGACAGTGGCTCCAAGTCCTCCAGGACAACTAGGATTAATCATTTTCCCTGTCCAATAATGAGTATTTGCATGCATGCAAAGAGTGGCAGAGTTATAGCAGTTGTGGGGCATATGGGTGTGGGCAGTGAA

>GL-12_K15_T7_H04

GAGGTACCACAGACAAAAAATATTCCTGAGGGTAGGCAGACTATTCGTGGGGGGGAGTTACCCACCTGATGCATTGGGAGTTGGTTGTGTCTATCGTATTGCTAAATTTTACACAGGTGAGGTTTGAGGTATGGGTTATTTCCAGATTGGAAACAAGAGGTCCTACTAAAACGGAAGTGGTGTTTATGTGTTGAAGTTGTTCCCTTGTTCAGGTACAGGGATTGAAATGTATGGCCTTAAGTGCAGAGGGAGGCACATCCAACAGTTAGTAGGGTTTTGGGCCGAGACCTCATGAAACCCAGTGAGGGTGGTATTAAATAGGCTTACCAGGCGAGTATGGGTACGGAGGGTTTCATGTAGTTTTGAGAGATCTAGTCCTTTGTAGGGGCTAGGCGTGCTATGTACTCAGGTCAGTTGGGAGATTACTTCCTTTACGTGTTTTTCTCTTGCCTGATCTTGAACTCCACCCCTATCAGACATACCAGTATGGGTGAACTAAGTCCAACAGACAGTGGCTCCAAGTCCTCCAGGACAACTAGGATTAATCATTTTCCCTGTCCAATAATGAGTATTTGCATGCATGCAAAGAGTGGCAGAGTTATAGCAGTTGCGGGGCATATGGGTGTGGGCAGTGAA

>GL-12_K16_T7_A05

TTCACTGCCCACACCCATATGCCCCACAACTGCTATAACTCTGCCACTCTTTGCATGCATGCAAATACTCATTATTGGACAGGGAAAATGATTAATCCTAGTTGTCCTGGAAGACTTGGAGCCACTGTCTGTCGGACTTACTTCACCCATACTGGTATGTCTGAGGGGGGTGGAGTTCAAGATCAGGCAAGAGAAAAACATGTAAAGGAAGTAACCTCCCAACTGACCCGGGTACATAGCACCCCTAGCCCCTACAAAGGACTAGATCTCTTAAAACTACATGAAACCCTCCATACCCATACTTGCCTGGTAAGCCTATTTAATACCACCCTCACTGGGCTCCATGAGGTCTCGGCCCAAAACCCTACTAACTGTTGGATGTGCCTCCCCCTGTATTTCAGGCCATGCATTTCAATCCCTGTACCTGAACAATGGAACAACTACAGCACAGAAATAAACACCACTTCCGTTTTAGTAGGACCTCTTGTTTCCAATCTGGAAATAACCCATACCTCAAACCTCACCTGTGTAAAATTTAGCAATACGATAGACACAACCAACTCCCAATGCATCAGGTGGGTAACACCTCCCACACGAATAGTCTGCCTACCCTCAGGAATATTTTTTGTCTGTGGTACCTC

>GL-12_K17_T7_B05

GAGGTACCACAGACAAAAAATATTCCTGAGGGTAGGCAGACTATTCGTGGGGGGGAGTTACCCACCTGATGCATTGGGAGTTGGTTGTGTCTATCGTATTGCTAAATTTTACACAGGTGAGGTTTGAGGTATGGGTTATTTCCAGATTGGAAACAAGAGGTCCTACTAAAACGGAAGTGGTGTTTATGTGTTGAAGTTGTTCCCTTGTTCAGGTACAGGGATTGAAATGTATGGCCTTAAGTGCAGAGGGAGGCACATCCAACAGTTAGTAGGGTTTTGGGCCGAGACCTCATGAAACCCAGTGAGGGTGGTATTAAATAGGCTTACCAGGCGAGTATGGGTACGGAGGGTTTCATGTAGTTTTGAGAGATCTAGTCCTTTGTAGGGGCTAGGCGTGCTATGTACTCAGGTCAGTTGGGAGATTACTTCCTTTACGTGTTTTTCTCTTGCCTGATCTTGAACTCCACCCCTATCAGACATACCAGTATGGGTGAACTAAGTCCAACAGACAGTGGCTCCAAGTCCTCCAGGACAACTAGGATTAATCATTTTCCCTGTCCAATAATGAGTATTTGCATGCATGCAAAGAGTGGCAGAGTTATAGCAGTTGTGGGGCATATGGGTGTGGGCAGTGAA

>GL-12_K18_T7_C05

GAGGTACCACAGACAAAAAATATTCCTGAGGGTAGGCAGACTATTCGTGGGGGGGAGTTACCCACCTGATGCATTGGGAGTTGGTTGTGTCTATCGTATTGCTAAATTTTACACAGGTGAGGTTTGAGGTATGGGTTATTTCCAGATTGGAAACAAGAGGTCCTACTAAAACGGAAGTGGTGTTTATGTGTTGAAGTTGTTCCCTTGTTCAGGTACAGGGATTGAAATGTATGGCCTTAAGTGCAGAGGGAGGCACATCCAACAGTTAGTAGGGTTTTGGGCCGAGACCTCATGAAACCCAGTGAGGGTGGTATTAAATAGGCTTACCAGGCGAGTATGGGTACGGAGGGTTTCATGTAGTTTTGAGAGATCTAGTCCTTTGTAGGGGCTAGGCGTGCTATGTACTCAGGTCAGTTGGGAGATTACTTCCTTTACGTGTTTTTCTCTTGCCTGATCTTGAACTCCACCCCTATCAGACATACCAGTATGGGTGAACTAAGTCCAACAGACAGTGGCTCCAAGTCCTCCAGGACAACTAGGATTAATCATTTTCCCTGTCCAATAATGAGTATTTGCATGCATGCAAAGAGTGGCAGAATTATAGCAGTTGTGGGGCATATGGGTGTGGGCAGTGAA

>GL-12_K19_T7_D05

GAGGTACCACAGACAAAAAATATTCCTGAGGGTAGGCAGACTATTCGTGGGGGGGGAGTTACCCACCTGATGCATTGGGAGTTGGTTGTGTCTATCGTATTGCTAAATTTTACACAGGTGAGGTTTGAGGTATGGGTTATTTCCAGATTGGAAACAAGAGGTCCTACTAAAACGGAAGTGGTGTTTATGTGTTGAAGTTGTTCCCTTGTTCAGGTACAGGGATTGAAATGTATGGCCTTAAGTGCAGAGGGAGGCACATCCAACAGTTAGTAGGGTTTTGGGCCGAGACCTCATGAAACCCAGTGAGGGTGGTATTAAATAGGCTTACCAGGCGAGTATGGGTACGGAGGGTTTCATGTAGTTTTGAGAGATCTAGTCCTTTGTAGGGGCTAGGCGTGCTATGTACTCAGGTCAGTTGGGAGATTACTTCCTTTACGTGTTTTTCTCTTGCCTGATCTTGAACTCCACCCCCATCAGACATACCAGTTTGGGTGAAGTAAGTCCAACAGACAGTGACTCCAAGTCCTCCAGGACAACTAGGATTAATCATTTTTCCTGTCCAATAATGAGTATTTGCATGCATGCAAAGAGTGGCAGAGTGATGGCAGTTGCGGGGCATATGGGTGTGGGCAGTGAA

>GL-12_K21_T7_E05

GAGGTACCACAGACAAAAAATATTCCTGAGGGTAGGCAGACTATTCGTGGGGGGGAGTTACCCACCTGATGCATTGGGAGTTGGTTGTGTCTATCGTATTGCTAAATTTTACACAGGTGAGGTTTGAGGTATGGGTTATTTCCAGATTGGAAACAAGAGGTCCTACTAAAACGGAAGTGGTGTTTATGTGTTGAAGTTGTTCCCTTGTTCAGGTACAGGGATTGAAATGTATGGCCTTAAGTGCAGAGGGAGGCACATCCAACAGTTAGTAGGGTTTTGGGCCGAGACCTCATGAAACCCAGTGAGGGTGGTATTAAATAGGCTTACCAGGCGAGTATGGGTACGGAGGGTTTCATGTAGTTTTGAGAGATCTAGTCCTTTGTAGGGGCTAGGCGTGCTATGTACTCAGGTCAGTTGGGAGATTACTTCCTTTACGTGTTTTTCTCTTGCCTGATCTTGAACTCCACCCCTATCAGACATACCAGTATGGGTGAACTAAGTCCAACAGACAGTGGCTCCAAGTCCTCCAGGACAACTAGGATTAATCATTTTCCCTGTCCAATAATGAGTATTTGCATGCATGCAAAGAGTGGCAGAGTTATAGCAGTTGTGGGGCATATGGGTGTGGGCAGTGAA

>GL-12_K22_T7_F05

GAGGTACCACAGACAAAAAATATTCCTGAGGGTAGGCAGACTATTCGTGTGGGGGAGTTACCCACCTGATGCATTGGGAGTTGGTTGTGTCTATCGTATTGCTAAATTTTACACAGGTGAGGTTTGAGGTATGGGTTATTTCCAGATTGGAAACAAGAGGTCCTACTAAAACGGAAGTGGTGTTTATGTGTTGAAGTTGTTCCCTTGTTCAGGTACAGGGATTGAAATGTATGGCCTTAAGTGCAGAGGGAGGCACATCCAACAGTTAGTAGGGTTTTGGGCCGAGACCTCATGGAGCCCAGTGAGGGTGGTATTAAATAGGCTTACCAGGCGAGTATGGGTACGGAGGGTTTCATGTAGTTTTGAGAGATCTAGTCCTTTGTAGGGGCTAGAGGTGCCATGTACCCGGGTGAGTTGGGAGATTACTTCTTTTACATGTTTTTCTCTTGCCTGATCTTGAACTCCACCCCCATCAGACATACCAGTTTGGGTGAAGTAAGTCCAACAGACAGTGACTCCAAGTCCTCCAGGACAACTAGGATTAATCATTTTTCCTGTCCAATAATGAGTATTTGCATGCATGCAAAGAGTGGCAGAGTGATAGCAGTTGCGGGGCATATGGGTGTGGGCAGTGAA

>GL-12_K23_T7_G05

GAGGTACCACAGACAAAAAATATTCCTGAGGGTAGGCAGACTATTCGTGTGGGAGGAGTTACCCACCTGATGCATTGGGAGTTGGCTGTGTCTATAGTATTGCTAAATTTTACACAGGTGAGGTTTGAGGTACGGGTTATTTCCAGATTGGAAAGAGGACCTACTAAAACAGAAGTGGTGTTTATTTCTGTGCTGAAGTTGTTCCATTGTTCAGGTATAGGGATTGAAATGTATGGCCTAAAGTGCAGGGGGAGGCACATCCAACAGTTAGTAGGGTTTTGGGCCGAGACCTCATGGAGCCCAGTCAGGGTGGTATTAAATAGGCTTACCAGGCCAGTATGGGTATGGAGGGTTTCATGTAGTTTTGAGAGATCTAGTCCTTTGTAGGGGCCAGGGGTGCTATGTACCCAGGTCAGTTGGGAGATTACTTCCTTTATGTGTTTTTCTGTTGCCTGATCTTGAACTCCACCCCCATCAGACATACTGGTATGAGTGAAGTGAGTCCTCCAAGTCCTCCAGGACAACTGGGATTAATCGTTTTTCCTGTCCAATAATGAGTATTTGCATGCATGCAAAGAGTGGCAGAGTTATAGCAGTTGTGGGGCATATGGGTGTGGGCAGTGAA

>GL-12_K24_T7_H05

TTCACTGCCCACACCCATATGCCCCACAACTGCTATAACTCTGCCACTCTTTGCATGCATGCAAATACTCATTATTGGACAGGGAAAATGATTAATCCTAGTTGTCCTGGAGGACTTGGAGCCACTGTCTGTTGGACTTAGTTCACCCATACTGGTATGTCTGATAGGGGTGGAGTTCAAGATCGGGCAAGAGAAAAACACGTAAAGGAAGTAATCTCCCAACTGACCTGAGTACATAGCACGCCTAGCCCCTACAAAGGACTAGATCTCTCAAAACTACATGAAACCCTCCGTACCCATACTCGCCTGGTAAGCCTATTTAATACCACCCTCACTGGGTTTCATGAGGTCTCGGCCCAAAACCCTACTAACTGTTGGATGTGCCTCCCTCTGCACTTAAGGCCATACATTTCAATCCCTGTACCTGAACAAGGGAACAACTTCAACACATAAACACCACTTCCGTTTTAGTAGGACCTCTTGTTTCCAATCTGGAAATAACCCATACCTCAAACCTCACCTGTGTAAAATTTAGCAATACGATAGACACAACCAACTCCCAATGCATCAGGTGGGTAACTCCCCCCCACGAATAGTCTGCCTACCCTCAGGAATATTTTTTGTCTGTGGTACCTC

>GL-12_K25_T7_A06

GAGGTACCACAGACAAAAAATATTCCTGAGGGTAGGCAGACTATTCGTGGGGGGGAGTTACCCACCTGATGCATTGGGAGTTGGTTGTGTCTATCGTATTGCTAAATTTTACACAGGTGAGGTTTGAGGTATGGGTTATTTCCAGATTGGAAACAAGAGGTCCTACTAAAACGGAAGTGGTGTTTATGTGTTGAAGTTGTTCCCTTGTTCAGGTACAGGGATTGAAATGTATGGCCTTAAGTGCAGAGGGAGGCACATCCAACAGTTAGTAGGGTTTTGGGCCGAGACCTCATGAAACCCAGTGAGGGTGGTATTAAATAGGCTTACCAGGCGAGTATGGGTACGGAGGGTTTCATGTAGTTTTGAGAGATCTAGTCCTTTGTAGGGGCTAGGCGTGCTATGTACTCAGGTCAGTTGGGAGATTACTTCCTTTACGTGTTTTTCTCTTGCCTGATCTTGAACTCCACCCCTATCAGACATACCAGTATGGGTGAACTAAGTCCAACAGACAGTGGCTCCAAGTCCTCCAGGACAACTAGGATTAATCATTTTCCCTGTCCAATAATGAGTATTTGCATGCATGCAAAGAGTGGCAGAGTTATAGCAGTTGTGGGGCATATGGGTGTGGGCAGTGAA

>GL-12_K27_T7_B06

TTCACTGCCCACACCCATATGCCCCACAACTGCTATAACTCTGCCACTCTTTGCATGCATGCAAATACTCATTATTGGACAGGGAAAATGATTAATCCTAGTTGTCCTGGAAGACTTGGAGCCACTGTCTGTCGGACTTACTTCACCCATACTGGTATGTCTGAGGGGGGTGGAGTTCAAGATCAGGCAAGAGAAAAACATGTAAAGGAAGTAACCTCCCAACTGACCCGGGTACATAGCACCCCTAGCCCCTACAAAGGACTAGATCTCTTAAAACTACATGAAACCCTCCATACCCATACTTGCCTGGTAAGCCTATTTAATACCACCCTCACTGGGCTCCATGAGGTCTCGGCCCAAAACCCTACTAACTGTTGGATGTGCCTCCCCCTGTATTTCAGGCCATGCATTTCAATCCCTGTACCTGAACAATGGAACAACTACAGCACAGAAATAAACACCACTTCCGTTTTAGTAGGACCTCTTGTTTCCAATCTGGAAATAACCCATACCTCAAACCTCACCTGTGTAAAATTTAGCAATACTGTAGACACAACCAACTCCCAATGCATCAGGTGGGTAACTCCTCCCACACGAATAGTCTGCCTACCCTCAGGAATATTTTTTGTCTGTGGTACCTC

>GL-12_K29_T7_C06

GAGGTACCACAGACAAAAAATATTCCTGAGGGTAGGCAGACTATTCGTGGGGGGGAGTTACCCACCTGATGCATTGGGAGTTGGTTGTGTCTATCGTATTGCTAAATTTTACACAGGTGAGGTTTGAGGTATGGGTTATTTCCAAATTGGAAACAAGAGGTCCTACTAAAACGGAAGTGGTGTTTATGTGTTGAAGTTGTTCCCTTGTTCAGGTACAGGGATTGAAATGTATGGCCTTAAGTGCAGAGGGAGGCACATCCAACAGTTAGTAGGGTTTTGGGCCGAGACCTCATGAAACCCAGTGAGGGTGGTATTAAATAGGCTTACCAGGCGAGTATGGGTACGGAGGGTTTCATGTAGTTTTGAGAGATCTAGTCCTTTGTAGGGGCTAGAGGTGCCATGTACCCGGGTGAGTTGGGAGATTACTTCTTTTACATGTTTTTCTCTTGCCTGATCTTGAACTCCACCCCCCTCAGACATACCAGTATGGGTGAAGTAAGTCCGACAGACAGTGGCTCCAAGTCTTCCAGGACAACTAGGATTAATCATTTTCCCTGTCCAATAATGAGTATTTGCATGCATGCAAAGAGTGGCAGAGTTATAGCAGTTGTGGGGCATATGGGTGTGGGCAGTGAA

>GL-12_K30_T7_D06

GAGGTACCACAGACAAAAAATATTCCTGAGGGTAGGCAGACTATTCGTGGGGGGGAGTTACCCACCTGATGCATTGGGAGTTGGTTGTGTCTATCGTATTGCTAAATTTTACACAGGTGAGGTTTGAGGTATGGGTTATTTCCAGATTGGAAACAAGAGGTCCTACTAAAACGGAAGTGGTGTTTATGTGTTGAAGTTGTTCCCTTGTTCAGGTACAGGGATTGAAATGTATGGCCTTAAGTGCAGAGGGAGGCACATCCAACAGTTAGTAGGGTTTTGGGCCGAGACCTCATGAAACCCAGTGAGGGTGGTATTAAATAGGCTTACCAGGCGAGTATGGGTACGGAGGGTTTCATGTAGTTTTGAGAGATCTAGTCCTTTGTAGGGGCTAGGCGTGCTATGTACTCAGGTCAGTTGGGAGATTACTTCCTTTACGTGTTTTTCTCTTGCCTGATCTTGAACTCCACCCCTATCAGACATACCAGTATGGGTGAACTAAGTCCAACAGACAGTGGCTCCAAGTCCTCCAGGACAACTAGGATTAATCATTTTCCCTGTCCAATAATGAGTATTTGCATGCATGCAAAGAGTGGCAGAGTTATAGCAGTTGTGGGGCATATGGGTGTGGGCAGTGAA

>GL-12_K32_T7_F06

GAGGTACCACAGACAAAAAATATTCCTGAGGGTAGGCAGACTATTCGTGGGGGGGAGTTACCCACCTGATGCATTGGGAGTTGGTTGTGTCTATCGTATTGCTAAATTTTACACAGGTGAGGTTTGAGGTATGGGTTATTTCCAGATTGGAAACAAGAGGTCCTACTAAAACGGAAGTGGTGTTTATGTGTTGAAGTTGTTCCCTTGTTCAGGTACAGGGATTGAAATGTATGGCCTTAAGTGCAGAGGGAGGCACATCCAACAGTTAGTAGGGTTTTGGGCCGAGACCTCATGAAACCCAGTGAGGGTGGTATTAAATAGGCTTACCAGGCGAGTATGGGTACGGAGGGTTTCATGTAGTTTTGAGAGATCTAGTCCTTTGTAGGGGCTAGGCGTGCTATGTACTCAGGTCAGTTGGGAGATTACTTCCTTTACGTGTTTTTCTCTTGCCTGATCTTGAACTCCACCCCTATCAGACATACCAGTATGGGTGAACTAAGTCCAACAGACAGTGGCTCCAAGTCCTCCAGGACAACTAGGATTAATCATTTTCCCTGTCCAATAATGAGTATTTGCATGCATGCAAAGAGTGGCAGAGTTATAGCAGTTGTGGGGCATATGGGTGTGGGCAGTGAA

>GL-12_K33_T7_G06

GAGGTACCACAGACAAAAAATATTCCTGAGGGTAGGCAGACTATTCGTGGGGGGGAGTTACCCACCTGATGCATTGGGAGTTGGTTGTGTCTATCGTATTGCTAAATTTTACACAGGTGAGGTTTGAGGTATGGGTTATTTCCAGATTGGAAACAAGAGGTCCTACTAAAACGGAAGTGGTGTTTATGTGTTGAAGTTGTTCCCTTGTTCAGGTACAGGGATTGAAATGTATGGCCTTAAGTGCAGAGGGAGGCACATCCAACAGTTAGTAGGGTTTTGGGCCGAGACCTCATGAAACCCAGTGAGGGTGGTATTAAATAGGCTTACCAGGCGAGTATGGGTACGGAGGGTTTCATGTAGTTTTGAGAGATCTAGTCCTTTGTAGGGGCTAGGCGTGCTATGTACTCAGGTCAGTTGGGAGATTACTTCCTTTACGTGTTTTTCTCTTGCCTGATCTTGAACTCCACCCCTATCAGACATACCAGTATGGGTGAACTAAGTCCAACAGACAGTGGCTCCAAGTCCTCCAGGACAACTAGGATTAATCATTTTCCCTGTCCAATAATGAGTATTTGCATGCATGCAAAGAGTGGCAGAGTTATAGCAGTTGTGGGGCATATGGGTGTGGGCAGTGAA

>GL-12_K34_T7_H06

TTCACTGCCCACACCCATATGCCCCACAACTGCTATAACTCTGCCACTCTTTGCATGCATGCAAATACTCATTATTGGACAGGGAAAATGATTAATCCTAGTTGTCCTGGAAGACTTGGAGCCACTGTCTGTCGGACTTACTTCACCCATACTGGTATGTCTGAGGGGGGTGGAGTTTAAGATCAGGCAAGAGAAAAACATGTAAAGGAAGTAACCTCCCAACTGACCCGGGTACATAGCACCCCTAGCCCCTACAAAGGACTAGATCTCTTAAAACTACATGAAACCCTCCATACCCATACTTGCCTGGTAAGCCTATTTAATACCACCCTCACTGGGCTCCATGAGGTCTCGGCCCAAAACCCTACTAACTGTTGGATGTGCCTCCCCCTGTATTTCAGGCCATGCATTTCAATCCCTGTACCTGAACAATGGAACAACTACAGCACAGAAATAAACACCACTTCCGTTTTAGTAGGACCTCTTGTTTCCAATCTGGAAATAACCCATACCTCAAACCTCACCTGTGTAAAATTTAGCAATACTGTAGACACAACCAACTCCCAATGCATCAGGTGGGTAACTCCTCCCACACGAATAGTCTGCCTACCCTCAGGAATATTTTTTGTCTGTGGTACCTC

>GL-12_K35_T7_A07

GAGGTACCACAGACAAAAAATATTCCTGAGGGTAGGCAGACTATTCGTGGGGGGGAGTTACCCACCTGATGCATTGGGAGTTGGTTGTGTCTATCGTATTGCTAAATTTTACACAGGTGAGGTTTGAGGTATGGGTTATTTCCAGATTGGAAACAAGAGGTCCTACTAAAACGGAAGTGGTGTTTATTTCTGTGCTGTAGTTGTTCCATTGTTCAGGTACAGGGATTGAAATGCATGGCCTGAAATACAGGGGGAGGCACATCCAACAGTTAGTAGGGTTTTGGGCCGAGACCTCATGGAGCCCAGTGAGGGTGGTATTAAATAGGCTTACCAGGCAAGTATGGGTATGGAGGGTTTCATGTAGTTTTAAGAGATCTAGTCCTTTGTAGGGGCTAGGGGTGCTATGTACCCGGGTCAGTTGGGAGGTTACTTCCTTTACATGTTTTTCTCTTGCCTGATCTTGAACTCCACCCCCCTCAGACATACCAGTATGGGTGAAGTAAGTCCGACAGACAGTGGCTCCAAGTCTTCCAGGACAACTAGGATTAATCATTTTCCCTGTCCAATAATGAGTATTTGCATGCATGCAAAGAGTGGCAGAGTTATAGCAGTTGCGGGGCATATGGGTGTGGGCAGTGAA

>GL-12_K36_T7_B07

GAGGTACCACAGACAAAAAATATTCCTGAGGGTAGGCAGACTATTCGTGTGGGAGGAGTTACCCACCTGATGCATTGGGAGTTGGCTGTGTCTATAGTATTGCTAAATTTTACACAGGTGAGGTTTGAGGTATGGGTTATTTCCAGATTGGAAAGAGGACCTACTAAAACAGAAGTGGTGTTTATTTCTGTGCTGAAGTTGTTCCATTGTTCAGGTATAGGGATTGAAATGTATGGCCTAAAGTGCAGGGGGAGGCACATCCAACAGTTAGTAGGGTTTTGGGCCGAGACCTCATGGAGCCCAGTCAGGGTGGTATTAAATAGGCTTACCAGGCCAGTATGGGTATGGAGGGTTTCATGTAGTTTTGAGAGATCTAGTCCTTTGTAGGGGCCAGGGGTGCTATGTACCCAGGTCAGTTGGGAGATTACTTCCTTTATGTGTTTTTCTGTTGCCTGATCTTGAACTCCACCCCCATCAGACATACTGGTATGAGTGAAGTGAGTCCTCCAAGTCCTCCAGGACAACTGGGATTAATCGTTTTTCCTGTCCAATAATGAGTATTTGCATGCATGCAAAGAGTGGCAGAGTTATAGCAGTTGCGGGGCATATGGGTGTGGGCAGTGAA

>GL-12_K37_T7_C07

GAGGTACCACAGACAAAAAATATTCCTGAGGGTAGGCAGACTATTCGTGTGGGAGGAGTTACCCACCTGATGCATTGGGAGTTGGTTGTGTCTACAGTATTGCTAAATTTTACACAGGTGAGGTTTGAGGTATGGGTTATTTCCAGATTGGAAACAAGAGGTCCTACTAAAACGGAAGTGGTGTTTATTTCTGTGCTGTAGTTGTTCCATTGTTCAGGTACAGGGATTGAAATGCATGGCCTGAAATACAGGGGGAGGCACATCCAACAGTTAGTAGGGTTTTGGGCCGAGACCTCATGGAGCCCAGTGAGGGTGGTATTAAATAGGCTTACCAGGCGAGTATGGGTACGGAGGGTTTCATGTAGTTTTGAGAGATCTAGTCCTTTGTAGGGGCTAGGCGTGCTATGTACTCAGGTCAGTTGGGAGATTACTTCCTTTACGTGTTTTTCTCTTGCCTGATCTTGAACTCCACCCCTATCAGACATACCAGTATGGGTGAAGTAAGTCCGACAGACAGTGGCTCCAAGTCTTCCAGGACAACTAGGATTAATCATTTTCCCTGTCCAATAATGAGTATTTGCATGCATGCAAAGAGTGGCAGAGTTATAGCAGTTGCGGGGCATATGGGTGTGGGCAGTGAA

>GL-12_K40_T7_D07

GAGGTACCACAGACAAAAAATATTCCTGAGGGTAGGCAGACTATTCGTGGGGGGGAGTTACCCACCTGATGCATTGGGAGTTGGTTGTGTCTATCGTATTGCTAAATTTTACACAGGTGAGGTTTGAGGTATGGGTTATTTCCAGATTGGAAACAAGAGGTCCTACTAAAACGGAAGTGGTGTTTATGTGTTGAAGTTGTTCCCTTGTTCAGGTACAGGGATTGAAATGTATGGCCTTAAGTGCAGAGGGAGGCACATCCAACAGTTAGTAGGGTTTTGGGCCGAGACCTCATGAAACCCAGTGAGGGTGGTATTAAATAGGCTTACCAGGCGAGTATGGGTACGGAGGGTTTCATGTAGTTTTAGGAGATCTAGTCCTTTGTAGGGGCTAGGGGTGCTATGTACCCGGGTCAGTTGGGAGATTACTTCCTTTACATGTTTTTCTCTTGCCTGATCTTGAACTCCACCCCCCTCAGACATACCAGTATGGGTGAACTAAGTCCAACAGACAGTGGCTCCAAGTCCTCCAGGACAACTAGGATTAATCATTTTCCCTGTCCAATAATGAGTATTTGCATGCATGCAAAGAGTGGCAGAGTTATAGCAGTTGTGGGGCATATGGGTGTGGGCAGTGAA

>GL-12_K41_T7_E07

GAGGTACCACAGACAAAAAATATTCCTGAGGGTAGGCAGACTATTCGTGGGGGGGAGTTACCCACCTGATGCATTGGGAGTTGGTTGTGTCTATCGTATTGCTAAATTTTACACAGGTGAGGTTTGAGGTATGGGTTATTTCCAGATTGGAAACAAGAGGTCCTACTAAAACGGAAGTGGTGTTTATGTGTTGAAGTTGTTCCCTTGTTCAGGTACAGGGATTGAAATGTATGGCCTTAAGTGCAGAGGGAGGCACATCCAACAGTTAGTAGGGTTTTGGGCCGAGACCTCATGAAACCCAGTGAGGGTGGTATTAAATAGGCTTACCAGGCGAGTATGGGTACGGAGGGTTTCATGTAGTTTTGAGAGATCTAGTCCTTTGTAGGGGCTAGGCGTGCTATGTACTCAGGTCAGTTGGGAGATTACTTCCTTTACGTGTTTTTCTCTTGCCTGATCTTGAACTCCACCCCTATCAGACATACCAGTATGGGTGAACTAAGTCCAACAGACAGTGGCTCCAAGTCCTCCAGGACAACTAGGATTAATCATTTTCCCTGTCCAATAATGAGTATTTGCATGCATGCAAAGAGTGGCAGAGTTATAGCAGTTGTGGGGCATATGGGTGTGGGCAGTGAA

>GL-12_K42_T7_F07

GAGGTACCACAGACAAAAAATATTCCTGAGGGTAGGCAGACTATTCGTGTGGGAGGAGTTACCCACCTGATGCATTGGGAGTTGGTTGTGTCTACAGTATTGCTAAATTTTACACAGGTGAGGTTTGAGGTATGGGTTATTTCCAGATTGGAAACAAGAGGTCCTACTAAAACGGAAGTGGTGTTTATTTCTGTGCTGTAGTTGTTCCATTGTTCAGGTACAGGGATTGAAATGCATGGCCTGAAATACAGGGGGAGGCACATCCAACAGTTAGTAGGGTTTTGGGCCGAGACCTCATGGAGCCCAGTGAGGGTGGTATTAAATAGGCTTACCAGGCAAGTATGGGTATGGAGGGTTTCATGTAGTTTTAAGAGATCTAGTCCTTTGTAGGGGCTAGGGGTGCTATGTACCCGGGTCAGTTGGGAGGTTACTTCCTTTACATGTTTTTCTCTTGCCTGATCTTGAACTCCACCCCCCTCAGACATACCAGTATGGGTGAAGTAAGTCCGACAGACAGTGGCTCCAAGTCTTCCAGGACAACTAGGATTAATCATTTTCCCTGTCCAATAATGAGTATTTGCATGCATGCAAAGAGTGGCAGAGTTATAGCAGTTGTGGGGCATATGGGTGTGGGCAGTGAA

>GL-12_K44_T7_G07

GAGGTACCACAGACAAAAAATATTCCTGAGGGTAGGCAGACTATTCGTGTGGGAGGTGTTACCCACCTGATGCATTGGGAGCTGGTTGTGTCTATAGTATTGCTAAATTTTACACAGGTGAGGTTTGAGGTATGGGTTATTTCCAGATTGGAAACAAGAGGTCCTACTAAAACGGAAGTGGTGTTTATGTGTTGAAGTTGTTCCCTTGTTCAGGTACAGGGATTGAAATGTATGGCCTTAAGTGCAGAGGGAGGCACATCCAACAGTTAGTAGGGTTTTGGGCCGAGACCTCATGGAGCCCAGTGAGGGTGGTATTAAATAGGCTTACCAGGCAAGTATGGGTATGGAGGGTTTCATGTAGTTTTAGGAGATCTAGTCCTTTGTAGGGGCTAGGGGTGCTATGTACCCGGGTCAGTTGGGAGATTACTTCCTTTACATGTTTTTCTCTTGCCTGATCTTGAACTCCACCCCCCTCAGACATACCAGTATGGGTGAAGTAAGTCCGACAGACAGTGGCTCCAAGTCTTCCAGGACAACTAGGATTAATCATTTTCCCTGTCCAATAATGAGTATTTGCATGCATGCAAAGAGTGGCAGAGTTATAGCAGTTGTGGGGCATATGGGTGTGGGCAGTGAA

>GL-12_K46_T7_H07

GAGGTACCACAGACAAAAAATATTCCTGAGGGTAGGCAGACTATTCGTGGGGGGGAGTTACCCACCTGATGCATTGGGAGTTGGTTGTGTCTATCGTATTGCTAAATTTTACACAGGTGAGGTTTGAGGTATGGGTTATTTCCAGATTGGAAACAAGAGGTCCTACTAAAACGGAAGTGGTGTTTATGTGTTGAAGTTGTTCCCTTGTTCAGGTACAGGGATTGAAATGTATGGCCTTAAGTGCAGAGGGAGGCACATCCAACAGTTAGTAGGGTTTTGGGCCGAGACCTCATGAAACCCAGTGAGGGTGGTATTAAATAGGCTTACCAGGCGAGTATGGGTACGGAGGGTTTCATGTAGTTTTGAGAGATCTAGTCCTTTGTAGGGGCTAGGCGTGCTATGTACTCAGGTCAGTTGGGAGATTACTTCCTTTACGTGTTTTTCTCTTGCCTGATCTTGAACTCCACCCCTATCAGACATACCAGTATGGGTGAACTAAGTCCAACAGACAGTGGCTCCAAGTCCTCCAGGACAACTAGGATTAATCATTTTCCCTGTCCAATAATGAGTATTTGCATGCATGCAAAGAGTGGCAGAGTTATAGCAGTTGTGGGGCATATGGGTGTGGGCAGTGAA

>GL-12_K47_T7_A08

TTCACTGCCCACACCCATATGCCCCACAACTGCTATAACTCTGCCACTCTTTGCATGCATGCAAATACTCATTATTGGACAGGGAAAATGATTAATCCTAGTTGTCCTGGAGGACTTGGAGCCACTGTCTGTTGGACTTAGTTCACCCATACTGGTATGTCTGATAGGGGTGGAGTTCAAGATCAGGCAAGAGAAAAACACGTAAAGGAAGTAATCTCCCAACTGACCTGAGTACATAGCACGCCTAGCCCCTACAAAGGACTAGATCTCTCAAAACTACATGAAACCCTCCGTACCCATACTCGCCTGGTAAGCCTATTTAATACCACCCTCACTGGGTTTCATGAGGTCTCGGCCCAAAACCCTACTAACTGTTGGATGTGCCTCCCTCTGCACTTAAGGCCATACATTTCAATCCCTGTACCTGAACAAGGGAACAACTTCAACACATAAACACCACTTCCGTTTTAGTAGGACCTCTTGTTTCCAATCTGGAAATAACCCATACCTCAAACCTCACCTGTGTAAAATTTAGCAATACGATAGACACAACCAACTCCCAATGCATCAGGTGGGTAACTCCCCCCCACGAATAGTCTGCCTACCCTCAGGAATATTTTTTGTCTGTGGTACCTC

>GL-12_K48_T7_B08

TTCACTGCCCACACCCATATGCCCCACAACTGCTATAACTCTGCCACTCTTTGCATGCATGCAAATACTCATTATTGGACAGGGAAAATGATTAATCCTAGTTGTCCTGGAAGACTTGGAGCCACTGTCTGTCGGACTTACTTCACCCATACTGGTATGTTTGAGGGGGGTGGAGTTCAAGATCAGGCAAGAGAAAAACATGTAAAGGAAGTAACCTCCCAACTGACCCGGGTACATAGCACCCCTAGCCCCTACAAAGGACTAGATCTCTTAAAACTACATGAAACCCTCCATACCCATACTTGCCTGGTAAGCCTATTTAATACCACCCTCACTGGGCTCCATGAGGTCTCGGCCCAAAACCCTACTAACTGTTGGATGTGCCTCCCCCTGTATTTCAGGCCATGCATTTCAATCCCTGTACCTGAACAATGGAACAACTACAGCACAGAAATAAACACCACTTCCGTTTTAGTAGGACCTCTTGTTTCCAATCTGGAAATAACCCATACCTCAAACCTCACCTGTGTAAAATTTAGCAATACTGTAGACACAACCAACTCCCAATGCATCAGGTGGGTAACTCCTCCCACACGAATAGTCTGCCTACCCTCAGGAATATTTTTTGTCTGTGGTACCTC

>GL-12_K53_T7_C08

GAGGTACCACAGACAAAAAATATTCCTGAGGGTAGGCAGACTATTCGTGTGGGAGGAGTTACCCACCTGATGCATTGGGAGTTGGTTGTGTCTACAGTATTGCTAAATTTTACACAGGTGAGGTTTGAGGTATGGGTTATTTCCAGATTGGAAACAAGAGGTCCTACTAAAACGGAAGTGGTGTTTATTTCTGTGCTGTAGTTGTTCCATTGTTCAGGTACAGGGATTGAAATGCATGGCCTGAAATACAGGGGGAGGCACATCCAACAGTTAGTAGGGTTTTGGGCCGAGACCTCATGGAGCCCAGTGAGGGTGGTATTAAATAGACTTACCAGGCAAGTATGGGTATGGAGGGTTTCATGTAGTTTTAGGAGATCTAGCCCTTTGTAGGGGCTAGGGGTGCTATGTACCCGGGTCAGTTGGGAGATTACTTCCTTTACATGTTTTTCTCTTGCCTGATCTTGAACTCCACCCCCCTCAGACATACCAGTATGGGTGAAGTAAGTCCGACAGACAGTGGCTCCAAGTCTTCCAGGACAACTAGGATTAATCATTTTCCCTGTCCAATAATGAGTATTTGCATGCATGCAAAGAGTGGCAGAGTTATAGCAGTTGTGGGGCATATGGGTGTGGGCAGTGAA

>GL-12_K55_T7_D08

GAGGTACCACAGACAAAAAATATTCCTGAGGGTAGGCAGACTATTCGTGGGGGGGAGTTACCCACCTGATGCATTGGGAGTTGGTTGTGTCTATCGTATTGCTAAATTTTACACAGGTGAGGTTTGAGGTATGGGTTATTTCCAGATTGGAAACAAGAGGTCCTACTAAAACGGAAGTGGTGTTTATGTGTTGAAGTTGTTCCCTTGTTCAGGTACAGGGATTGAAATGTATGGCCTTAAGTGCAGAGGGAGGCACATCCAACAGTTAGTAGGGTTTTGGGCCGAGACCTCATGAAACCCAGTGAGGGTGGTATTAAATAGGCTTACCAGGCGAGTATGGGTACGGAGGGTTTCATGTAGTTTTGAGAGATCTAGTCCTTTGTAGGGGCTAGGCGTGCTATGTACTCAGGTCAGTTGGGAGATTACTTCCTTTACGTGTTTTTCTCTTGCCTGATCTTGAACTCCACCCCTATCAGACATACCAGTATGGGTGAACTAAGTCCAACAGACAGTGGCTCCAAGTCCTCCAGGACAACTAGGATTAATCATTTTCCCTGTCCAATAATGAGTATTTGCATGCATGCAAAGAGTGGCAGAGTTATAGCAGTTGTGGGGCATATGGGTGTGGGCAGTGAA

**MS-III**

>GL-14-K1_G03

TTCACTGCCCACACCCATATGCCCCACAACTGCTATAACTCTGCCACTCTTTGCATGCATGCAAATACTCATTATTGGACAGGGAAAATGATTAATCCTAGTTGTCCTGGAGGACTTGGAGCCACTGTCTGTTGGACTTAGTTCACCCATACTGGTATGTCTGATAGGGGTGGAGTTCAAGATCAGGCAAGAGAAAAACACGTAAAGGAAGTAATCTCCCAACTGACCTGAGTACATAGCACGCCTAGCCCCTACAAAGGACTAGATCTCTCAAAACTACATGAAACCCTCCGTACCCATACTCGCCTGGTAAGCCTATTTAATACCACCCTCACTGGGCTCCATGAGGTCTCGGCCCAAAACCCTACTAACTGTTGGATGTGCCTCCCCCTGTATTTCAGGCCATGCATTTCAATCCCTGTACCTGAACAATGGAACAACTACAGCACAGAAATAAACACCACTTCCGTTTTAGTAGGACCTCTTGTTTCCAATCTGGAAATAACCCATACCTCAAACCTCACCTGTGTAAAATTTAGCAATACTGTAGACACAACCAACTCCCAATGCATCAGGTGGGTAACTCCTTCCACACGAATAGTCTGCCTACCCTCAGGAATATTTTTTGTCTGTGGTACCTC

>GL-14-K2_H03

TTCACTGCCCACACCCATATGCCCCACAACTGCTATAACTCTGCCACTCTTTGCATGCATGCAAATACTCATTATTGGACAGGGAAAATGATTAATCCTAGTTGTCCTGGAGGACTTGGAGCCACTGTCTGTTGGACTTAGTTCACCCATACTGGTATGTCTGATAGGGGTGGAGTTCAAGATCAGGCAAGAGAAAAACACGTAGAGGAAGTAATCTCCCAACTGACCTGAGTACATAGCACGCCTAGCCCCTACAAAGGACTAGATCTCTCAAAACTACATGAAACCCTCCGTACCCATACTCGCCTGGTAAGCCTATTTAATACCACCCTCACTGGGTTTCATGAGGTCTCGGCCCAAAACCCTACTAACTGTTGGATGTGCCTCCCTCTGCACTTAAGGCCATACATTTCAATCCCTGTACCTGAACAAGGGAACAACTTCAACACATAAACACCACTTCCGTTTTAGTAGGACCTCTTGTTTCCAATCTGGAAATAACCCATACCTCAAACCTCACCTGTGTAAAATTTAGCAATACGATAGACACAACCAACTCCCAATGCATCAGGTGGGTAACTCCCCCCCACGAATAGTCTGCCTACCCTCAGGAATATTTTTTGTCTGTGGTACCTC

>GL-14-K3_A04

TTCACTGCCCACACCCATATGCCCCACAACTGCTATAACTCTGCCACTCTTTGCATGCATGCAAATACTCATTATTGGACAGGGAAAATGATTAATCCTAGTTGTCCTGGAGGACTTGGAGCCACTGTCTGTTGGACTTAGTTCACCCATACTGGTATGTCTGATAGGGGTGGAGTTCAAGATCAGGCAAGAGAAAAACACGTAAAGGAAGTAATCTCCCAACTGACCTGAGTACATAGCACGCCTAGCCCCTACAAAGGACTAGATCTCTCAAAACTACATGAAACCCTCCGTACCCATACTCGCCTGGTAAGCCTATTTAATACCACCCTCACTGGGTTTCATGAGGTCTCGGCCCAAAACCCTACTAACTGTTGGATGTGCCTCCCTCTGCACTTAAGGCCATACATTTCAATCCCTGTACCTGAACAAGGGAACAACTTCAACACATAAACACCACTTCCGTTTTAGTAGGACCTCTTGTTTCCAATCTGGAAATAACCCATACCTCAAACCTCACCTGTGTAAAATTTAGCAATACGATAGACACAACCAACTCCCAATGCATCAGGTGGGTAACTCCCCCCCACGAATAGTCTGCCTACCCTCAGGAATATTTTTTGTCTGTGGTACCTC

>GL-14-K4_B04

GAGGTACCACAGACAAAAAATATTCCTGAGGGTAGGCAGACTATTCGTGGGGGGGAGTTACCCACCTGATGCATTGGGAGTTGGTTGTGTCTATCGTATTGCTAAATTTTACACAGGTGAGGTTTGAGGTATGGGTTATTTCCAGATTGGAAACAAGAGGTCCTACTAAAACGGAAGTGGTGTTTATGTGTTGAAGTTGTTCCCTTGTTCAGGTACAGGGATTGAAATGTATGGCCTTAAGTGCAGAGGGAGGCACATCCAACAGTTAGTAGGGTTTTGGGCCGAGACCTCATGAAACCCAGTGAGGGTGGTATTAAATAGGCTTACCAGGCGAGTATGGGTACGGAGGGTTTCATGTAGTTTTGAGAGATCTAGTCCTTTGTAGGGGCTAGGCGTGCTATGTACTCAGGTCAGTTGGGAGATTACTTCCTTTACGTGTTTTTCCCTTGCCTGATCTTGAACTCCACCCCTATCAGACATACCAGTATGGGTGAACTAAGTCCAACAGACAGTGGCTCCAAGTCCTCCAGGACAACTAGGATTAATCATTTTCCCTGTCCAATAATGAGTATTTGCATGCATGCAAAGAGTGGCAGAGTTATAGCAGTTGTGGGGCATATGGGTGTGGGCAGTGAA

>GL-14-K6_D04

GAGGTACCACAGACAAAAAATATTCCTGAGGGTAGGCAGACTATTCGTGGGGGGGAGTTACCCACCTGATGCATTGGGAGTTGGTTGTGTCTATCGTATTGCTAAATTTTACACAGGTGAGGTTTGAGGTATGGGTTATTTCCAGATTGGAAACAAGAGGTCCTACTAAAACGGAAGTGGTGTTTATGTGTTGAAGTTGTTCCCTTGTTCAGGTACAGGGATTGAAATGTATGGCCTTAAGTGCAGAGGGAGGCACATCCAACAGTTAGTAGGGTTTTGGGCCGAGACCTCATGAAACCCAGTGAGGGTGGTATTAAATAGGCTTACCAGGCGAGTATGGGTACGGAGGGTTTCATGTAGTTTTGAGAGATCTAGTCCTTTGTAGGGGCTAGGCGTGCTATGTACTCAGGTCAGTTGGGAGATTACTTCCTTTACGTGTTTTTCTCTTGCCTGATCTTGAACTCCACCCCTATCAGACATACCAGTATGGGTGAACTAAGTCCAACAGACAGTGGCTCCAAGTCCTCCAGGACAACTAGGATTAATCATTTTCCCTGTCCAATAATGAGTATTTGCATGCATGCAAAGAGTGGCAGAGTTATAGCAGTTGCGGGGCATATGGGTGTGGGCAGTGAA

>GL-14-K7_E04

TTCACTGCCCACACCCATATGCCCCGCAACTGCTATAACTCTGCCACTCTTTGCATGCATGCAAATACTCATTATTGGACAGGGAAAATGATTAATCCTAGTTGTCCTGGAGGACTTGGAGCCACTGTCTGTTGGACTTACTTCACCCATACCAGTATGTCTGATGGGGGTGGAATTCAAGGTCAGGCAAGAGAAAAACAAGTAAAGGAAGCAATCTCCCAACTGACCCGGGGACATAGCACCCCTAGCCCCTACAAAGGACTAGTTCTCTCAAAACTACATGAAACCCTCCGTACCCATACTCGCCTGGTAAGCCTATTTAATACCACCCTCACTGGGCTCCATGAGGTCTCGGCCCAAAACCCTACTAACTGTTGGATATGCCTCCCCCTGAACCTCAGGCCATATGTTTCAATCCCTGTACCTGAACAATGGAACAACTTCAGCACAGAAATAAACACCACTTCCGTTTTAGTAGGACCTCTTGTTTCCAATCTGGAAATAACCCATACCTCAAACCTCACCTGTGTAAAATTTAGCAATACGATAGACACAACCAACTCCCAATGCATCAGGTGGGTAACTCCCCCCCACGAATAGTCTGCCTACCCTCAGGAATATTTTTTGTCTGTGGTACCTC

>GL-14-K8_F04

GAGGTACCACAGACAAAAAATATTCCTGAGGGTAGGCAGACTATTTGTGTGGGAGGAGTTACCCACCTGATGCATTGGGAGTTGGTTGTGTATGTAGTATTGCTAAATTTTACACAGGTGAGGTTTGAGGTATGGGTTATTTCCAGATTGGAAACAAGAGGTCCTACTAAAACGGAAGTGGTGTTTATTTCTGTGCTGAAGTTGTTCCATTGTTCAGGTACAGGGATTGAAACATATGGCCTGAAGTTCAGGGGGAGGCATATCCAACAGTTAGTAGGGTTTTGGGCCGAGACCTCATGGAGCCCAGTGAGGGTGGTATTAAATAGGCTTACCAGGCGAGTATGGGTACGGAGGGTTTCATGTAGTTTTGAGAGATCTAGTCCTTTGTAGGGGCTAGAGGTGCCATGTACCCGGGTGAGTTGGGAGATTACTTCTTTTACATGTTTTTCTCTTGCCTGATCTTGAACTCCACCCCCATCAGACATACCAGTTTGGGTGAAGTAAGTCCAACAGACAGTGACTCCAAGTCCTCCAGGACAACTAGGATTAATCATTTTTCCTGTCCAATAATGAGTATTTGCATGCATGCAAAGAGTGGCAGAGTGATAGCAGTTGCGGGGCATATGGGTGTGGGCAGTGAA

>GL-14-K11_G04

TTCACTGCCCACACCCATATGCCCCACAACTGCTATAACTCTGCCACTCTTTGCATGCATGCAAATACTCATTATTGGACAGGAAAAACGATTAATCCCAGTTGTCCTGGAGGACTTGGAGGACTCACTTCACTCATACCAGTATGTCTGATGGGGGTGGAGTTCAAGATCAGGCAACAGAAAAACACATAAAGGAAGTAATCTCCCAACTGACCTGGGTACATAGCACCCCTGGCCCCTACAAAGGACTAGATCTCTCAAAACTACATGAAACCCTCCGTACCCATACTCGCCTGGTAAGCCTATTTAATACCACCCTCACTGGGTTTCATGAGGTCTCGGCCCAAAACCCTACTAACTGTTGGATGTGCCTCCCTCTGCACTTAAGGCCATACATTTCAATCCCTGTACCTGAACAAGGGAACAACTTCAACACATAAACACCACTTCCGTTTTAGTAGGACCTCTTGTTTCCAATCTGGAAATAACCCATACCTCAAACCTCACCTGTGTAAAATTTAGCAATACGATAGACACAACCAACTCCCAATGCATCAGGTGGGTAACTCCCCCCCACGAATAGTCTGCCTACCCTCAGGAATATTTTTTGTCTGTGGTACCTC

>GL-14-K14_H04

TTCACTGCCCACACCCATATGCCCCACAACTGCTATAACTCTGCCACTCTTTGCATGCATGCAAATACTCATTATTGGACAGGGAAAATGATTAATCCTAGTTGTCCTGGAAGACTTGGAGCCACTGTCTGTCGGACTTACTTCACCCATACTGGTATGTCTGAGGGGGGTGGAGTTCGAGATCAGGCAAGAGAAAAACATGTAAAGGAAGTAACCTCCCAACTGACCCGGGTACATAGCACCCCTAGCCCCTACAAAGGACTAGATCTCTTAAAACTACATGAAACCCTCCATACCCATACTTGCCTGGTAAGCCTATTTAATACCACCCTCACTGGGCTCCATGAGGTCTCGGCCCAAAACCCTACTAACTGTTGGATGTGCCTCCCCCTGTATTTCAGGCCATGCATTTCAATCCCTGTACCTGAACAATGGAACAACTACAGCACAGAAATAAACACCACTTCCGTTTTAGTAGGACCTCTTGTTTCCAATCTGGAAATAACCCATACCTCAAACCTCACCTGTGTAAAATTTAGCAATACTGTAGACACAACCAACTCCCAATGCATCAGGTGGGTAACTCCTCCCACACGAATAGTCTGCCTACCCTCAGGAATATTTTTTGTCTGTGGTACCTC

>GL-14-K17_A05

GAGGTACCACAGACAAAAAATATTCCTGAGGGTAGGCAGACTATTCGTGGGGGGGAGTTACCCACCTGATGCATTGGGAGTTGGTTGTGTCTATCGTATTGCTAAATTTTACACAGGTGAGGTTTGAGGTATGGGTTATTTCCAGATTGGAAACAAGAGGTCCTACTAAAACGGAAGTGGTGTTTATGTGTTGAAGTTGTTCCCTTGTTCAGGTACAGGGATTGAAATGTATGGCCTTAAGTGCAGAGGGAGGCACATCCAACAGTTAGTAGGGTTTTGGGCCGAGACCTCATGAAACCCAGTGAGGGTGGTATTAAATAGGCTTACCAGGCGAGTATGGGTACGGAGGGTTTCATGTAGTTTTGAGAGATCTAGTCCTTTGTAGGGGCTAGGCGTGCTATGTACTCAGGTCAGTTGGGAGATTACTTCCTTTACGTGTTTTTCTCTTGCCTGATCTTGAACTCCACCCCTATCAGACATACCAGTATGGGTGAACTAAGTCCAACAGACAGTGGCTCCAAGTCCTCCAGGACAACTAGGATTAATCATTTTCCCTGTCCAATAATGAGTATTTGCATGCATGCAAAGAGTGGCAGAGTTATAGCAGTTGTGGGGCATATGGGTGTGGGCAGTGAA

>GL-20-K1_B05

GAGGTACCACAGACAAAAAATATTCCTGAGGGTAGGCAGACTATTCGTGGGGGGGAGTTACCCACCTGATGCATTGGGAGTTGGTTGTGTCTATCGTATTGCTAAATTTTACACAGGTGAGGTTTGAGGTATGGGTTATTTCCAGATTGGAAACAAGAGGTCCTACTAAAACGGAAGTGGTGTTTATGTGTTGAAGTTGTTCCCTTGTTCAGGTACAGGGATTGAAATGTATGGCCTTAAGTGCAGAGGGAGGCACATCCAACAGTTAGTAGGGTTTTGGGCCGAGACCTCATGAAACCCAGTGAGGGTGGTATTAAATAGGCTTACCAGGCGAGTATGGGTACGGAGGGTTTCATGTAGTTTTGAGAGATCTAGTCCTTTGTAGGGGCTAGGCGTGCTATGTACTCAGGTCAGTTGGGAGATTACTTCCTTTACGTGTTTTTCTCTTGCCTGATCTTGAACTCCACCCCTATCAGACATACCAGTATGGGTGAACTAAGTCCAACAGACAGTGGCTCCAAGTCCTCCAGGACAACTAGGATTAATCATTTTCCCTGTCCAATAATGAGTATTTGCATGCATGCAAAGAGTGGCAGAGTTATAGCAGTTGTGGGGCATATGGGTGTGGGCAGTGAA

>GL-20-K3_C05

GAGGTACCACAGACAAAAAATATTCCTGAGGGTAGGCAGACTATTCGTGGGGGGGAGTTACCCACCTGATGCATTGGGAGTTGGTTGTGTCTATCGTATTGCTAAATTTTACACAGGTGAGGTTTGAGGTATGGGTTATTTCCAGATTGGAAACAAGAGGTCCTACTAAAACGGAAGTGGTGTTTATGTGTTGAAGTTGTTCCCTTGTTCAGGTACAGGGATTGAAATGTATGGCCTTAAGTGCAGAGGGAGGCACATCCAACAGTTAGTAGGGTTTTGGGCCGAGACCTCATGAAACCCAGTGAGGGTGGTATTAAATAGGCTTACCAGGCGAGTATGGGTACGGAGGGTTTCATGTAGTTTTGAGAGATCTAGTCCTTTGTAGGGGCTAGGCGTGCTATGTACTCAGGTCAGTTGGGAGATTACTTCCTTTACGTGTTTTTCTCTTGCCTGATCTTGAACTCCACCCCTATCAGACATACCAGTATGGGTGAACTAAGTCCAACAGACAGTGGCTCCAAGTCCTCCAGGACAACTAGGATTAATCATTTTCCCTGTCCAATAATGAGTATTTGCATGCATGCAAAGAGTGGCAGAGTTATAGCAGTTGTGGGGCATATGGGTGTGGGCAGTGAA

>GL-20-K4_D05

GAGGTACCACAGACAAAAAATATTCCTGAGGGTAGGCAGACTATTCGTGGGGGGGAGTTACCCACCTGATGCATTGGGAGTTGGTTGTGTCTATCGTATTGCTAAATTTTACACAGGTGAGGTTTGAGGTATGGGTTATTTCCAGATTGGAAACAAGAGGTCCTACTAAAACGGAAGTGGTGTTTATGTGTTGAAGTTGTTCCCTTGTTCAGGTACAGGGATTGAAATGTATGGCCTTAAGTGCAGAGGGAGGCACATCCAACAGTTAGTAGGGTTTTGGGCCGAGACCTCATGAAACCCAGTGAGGGTGGTATTAAATAGGCTTACCAGGCGAGTATGGGTACGGAGGGTTTCATGTAGTTTTGAGAGATCTAGTCCTTTGTAGGGGCTAGGCGTGCTATGTACTCAGGTCAGTTGGGAGATTACTTCCTTTACGTGTTTTTCTCTTGCCTGACCTTGAACTCCACCCCTATCAGACATACCAGTATGGGTGAACTAAGTCCAACAGACAGTGGCTCCAAGTCCTCCAGGACAACTAGGATTAATCATTTTCCCTGTCCAATAATGAGTATTTGCATGCATGCAAAGAGTGGCAGAGTTATAGCAGTTGTGGGGCATATGGGTGTGGGCAGTGAA

>GL-20-K5_E05

GAGGTACCACAGACAAAAAATATTCCTGAGGGTAGGCAGACTATTCGTGGGGGGGAGTTACCCACCTGATGCATTGGGAGTTGGTTGTGTCTATCGTATTGCTAAATTTTACACAGGTGAGGTTTGAGGTATGGGTTATTTCCAGATTGGAAACAAGAGGTCCTACTAAAACGGAAGTGGTGTTTATGTGTTGAAGTTGTTCCCTTGTTCAGGTACAGGGATTGAAATGTATGGCCTTAAGTGCAGAGGGAGGCACATCCAACAGTTAGTAGGGTTTTGGGCCGAGACCTCATGAAACCCAGTGAGGGTGGTATTAAATAGGCTTACCAGGCGAGTATGGGTACGGAGGGTTTCATGTAGTTTTGAGAGATCTAGTCCTTTGTAGGGGCTAGGCGTGCTATGTACTCAGGTCAGTTGGGAGATTACTTCCTTTACGTGTTTTTCTCTTGCCTGATCTTGAACTCCACCCCTATCAGACATACCAGTATGGGTGAACTAAGTCCAACAGACAGTGGCTCCAAGTCCTCCAGGACAACTAGGATTAATCATTTTCCCTGTCCAATAATGAGTATTTGCATGCATGCAAAGAGTGGCAGAGTTATAGCAGTTGTGGGGCATATGGGTGTGGGCAGTGAA

>GL-20-K6_F05

TTCACTGCCCACACCCATATGCCCCACAACTGCTATAACTCTGCCACTCTTTGCATGCATGCAAATACTCATTATTGGACAGGGAAAATGATTAATCCTAGTTGTCCTGGAAGACTTGGAGCCACTGTCTGTCGGACTTACTTCACCCATACTGGTATGTCTGAGGGGGGTGGAGTTCAAGATCAGGCAAGAGAAAAACATGTAAAGGAAGTAACCTCCCAACTGACCCGGGTACATAGCACCCCTAGCCCCTACAAAGGACTAGATCTCTTAAAACTACATGAAACCCTCCATACCCATACTTGCCTGGTAAGCCTATTTAATACCACCCTCACTGGGCTCCATGAGGTCTCGGCCCAAAACCCTACTAACTGTTGGATGTGCCTCCCCCTGTATTTCAGGCCATGCATTTCAATCCCTGTACCTGAACAATGGAACAACTACAGCACAGAAATAAACACCACTTCCGTTTTAGTAGGACCTCTTGTTTCCAATCTGGAAATAACCCATACCTCAAACCTCACCTGTGTAAAATTTAGCAATACTGTAGACACAACCAACTCCCAATGCATCAGGTGGGTAACTCCTCCCACACGAATAGTCTGCCTACCCTCAGGAATATTTTTTGTCTGTGGTACCTC

>GL-20-K7_G05

GAGGTACCACAGACAAAAAATATTCCTGAGGGTAGGCAGACTATTCGTGGGGGGGAGTTACCCACCTGATGCATTGGGAGTTGGTTGTGTCTATCGTATTGCTAAATTTTACACAGGTGAGGTTTGAGGTATGGGTTATTTCCAGATTGGAAACAAGAGGTCCTACTAAAACGGAAGTGGTGTTTATGTGTTGAAGTTGTTCCCTTGTTCAGGTACAGGGATTGAAATGTATGGCCTTAAGTGCAGAGGGAGGCACATCCAACAGTTAGTAGGGTTTTGGGCCGAGACCTCATGAAACCCAGTGAGGGTGGTATTAAATAGGCTTACCAGGCGAGTATGGGTACGGAGGGTTTCATGTAGTTTTGAGAGATCTAGTCCTTTGTAGGGGCTAGGCGTGCTATGTACTCAGGTCAGTTGGGAGATTACTTCCTTTACGTGTTTTTCTCTTGCCTGATCTTGAACTCCACCCCTATCAGACATACCAGTATGGGTGAACTAAGTCCAACAGACAGTGGCTCCAAGTCCTCCAGGACAACTAGGATTAATCATTTTCCCTGTCCAATAATGAGTATTTGCATGCATGCAAAGAGTGGCAGAGTTATAGCAGTTGTGGGGCATATGGGTGTGGGCAGTGAA

>GL-20-K8_H05

GAGGTACCACAGACAAAAAATATTCCTGAGGGTAGGCAGACTATTCGTGGGGGGGAGTTACCCACCTGATGCATTGGGAGTTGGTTGTGTCTATCGTATTGCTAAATTTTACACAGGTGAGGTTTGAGGTATGGGTTATTTCCAGATTGGAAACAAGAGGTCCTACTAAAACGGAAGTGGTGTTTATGTGTTGAAGTTGTTCCCTTGTTCAGGTACAGGGATTGAAATGTATGGCCTTAAGTGCAGAGGGAGGCACATCCAACAGTTAGTAGGGTTTTGGGCCGAGACCTCATGAAACCCAGTGAGGGTGGTATTAAATAGGCTTACCAGGCGAGTATGGGTACGGAGGGTTTCATGTAGTTTTGAGAGATCTAGTCCTTTGTAGGGGCTAGGCGTGCTATGTACTCAGGTCAGTTGGGAGATTACTTCCTTTACGTGTTTTTCTCTTGCCTGATCTTGAACTTCACCCCTATCAGACATACCAGTATGGGTGAACTAAGTCCAACAGACAGTGGCTCCAAGTCCTCCAGGACAACTAGGATTAATCATTTTCCCTGTCCAATAATGAGTATTTGCATGCATGCAAAGAGTGGCAGAGTTATAGCAGTTGTGGGGCATATGGGTGTGGGCAGTGAA

>GL-20-K9_A06

GAGGTACCACAGACAAAAAATATTCCTGAGGGTAGGCAGACTATTCGTGGGGGGGAGTTACCCACCTGATGCATTGGGAGTTGGTTGTGTCTATCGTATTGCTAAATTTTACACAGGTGAGGTTTGAGGTATGGGTTATTTCCAGATTGGAAACAAGAGGTCCTACTAAAACGGAAGTGGTGTTTATGTGTTGAAGTTGTTCCCTTGTTCAGGTACAGGGATTGAAATGTATGGCCTTAAGTGCAGAGGGAGGCACATCCAACAGTTAGTAGGGTTTTGGGCCGAGACCTCATGAAACCCAGTGAGGGTGGTATTAAATAGGCTTACCAGGCGAGTATGGGTACGGAGGGTTTCATGTAGTTTTGAGAGATCTAGTCCTTTGTAGGGGCTAGGCGTGCTATGTACTCAGGTCAGTTGGGAGATTACTTCCTTTACGTGTTTTTCTCTTGCCTGATCTTGAACTCCACCCCTATCAGACATACCAGTATGGGTGAACTAAGTCCAACAGACAGTGGCTCCAAGTCCTCCAGGACAACTAGGATTAATCATTTTCCCTGTCCAATAATGAGTATTTGCATGCATGCAAAGAGTGGCAGAGTTATAGCAGTTGTGGGGCATATGGGTGTGGGCAGTGAA

>GL-20-K10_B06

GAGGTACCACAGACAAAAAATATTCCTGAGGGTAGGCAGACTATTCGTGGGGGGGAGTTACCCACCTGATGCATTGGGAGTTGGTTGTGTCTATCGTATTGCTAAATTTTACACAGGTGAGGTTTGAGGTATGGGTTATTTCCAGATTGGAAACAAGAGGTCCTACTAAAACGGAAGTGGTGTTTATGTGTTGAAGTTGTTCCCTTGTTCAGGTACAGGGATTGAAATGTATGGCCTTAAGTGCAGAGGGAGGCACATCCAACAGTTAGTAGGGTTTTGGGCCGAGACCTCATGAAACCCAGTGAGGGTGGTATTAAATAGGCTTACCAGGCGAGTATGGGTACGGAGGGTTTCATGTAGTTTTGAGAGATCTAGTCCTTTGTAGGGGCTAGGCGTGCTATGTACTCAGGTCAGTTGGGAGATTACTTCCTTTACGTGTTTTTCTCTTGCCTGATCTTGAACTCCACCCCTATCAGACATACCAGTATGGGTGAACTAAGTCCAGCAGACAGTGGCTCCAAGTCCTCCAGGACAACTAGGATTAATCATTTTCCCTGTCCAATAATGAGTATTTGCATGCATGCAAAGAGTGGCAGAGTTATAGCAGTTGTGGGGCATATGGGTGTGGGCAGTGAA

>GL-20-K13_D06

GAGGTACCACAGACAAAAAATATTCCTGAGGGTAGGCAGACTATTCGTGGAGGGGAGTTACCCACCTGATGCATTGGGAGTTGGTTGTGTCTATCGTATTGCTAAATTTTACACAGGTGAGGTTTGAGGTATGGGTTATTTCCAGATTGGAAACAAGAGGTCCTACTAAAACGGAAGTGGTGTTTATGTGTTGAAGTTGTTCCCTTGTTCAGGTACAGGGATTGAAATGTATGGCCTTAAGTGCAGAGGGAGGCACATCCAACAGTTAGTAGGGTTTTGGGCCGAGACCTCATGAAACCCAGTGAGGGTGGTATTAAATAGGCTTACCAGGCGAGTATGGGTACGGAGGGTTTCATGTAGTTTTGAGAGATCTAGTCCTTTGTAGGGGCTAGGCGTGCTATGTACTCAGGTCAGTTGGGAGATTACTTCCTTTACGTGTTTTTCTCTTGCCTGATCTTGAACTCCACCCCTATCAGACATACCAGTATGGGTGAACTAAGTCCAACAGACAGTGGCTCCAAGTCCTCCAGGACAACTAGGATTAATCATTTTCCCTGTCCAATAATGAGTATTTGCATGCATGCAAAGAGTGGCAGAGTTATAGCAGTTGTGGGGTATATGGGTGTGGGCAGTGAA

>GL-20-K14_E06

GAGGTACCACAGACAAAAAATATTCCTGAGGGTAGGCAGACTATTCGTGGGGGGGAGTTACCCACCTGATGCATTGGGAGTTGGTTGTGTCTATCGTATTGCTAAATTTTACACAGGTGAGGTTTGAGGTATGGGTTATTTCCGGATTGGAAACAAGAGGTCCTACTAAAACGGAAGTGGTGTTTATGTGTTGAAGTTGTTCCCTTGTTCAGGTACAGGGATTGAAATGTATGGCCTTAAGTGCAGAGGGAGGCACATCCAACAGTTAGTAGGGTTTTGGGCCGAGACCTCATGAAACCCAGTGAGGGTGGTATTAAATAGGCTTACCAGGCGAGTATGGGTACGGAGGGTTTCATGTAGTTTTGAGAGATCTAGTCCTTTGTAGGGGCTAGGCGTGCTATGTACTCAGGTCAGTTGGGAGATTACTTCCTTTACGTGTTTTTCTCTTGCCTGATCTTGAACTCCACCCCTATCAGACATACCAGTATGGGTGAACTAAGTCCAACAGACAGTGGCTCCAAGTCCTCCAGGACAACTAGGATTAATCATTTTCCCTGTCCAATAATGAGTATTTGCATGCATGCAAAGAGTGGCAGAGTTATAGCAGTTGTGGGGCATATGGGTGTGGGCAGTGAA

>GL-20-K15_F06

GAGGTACCACAGACAAAAAATATTCCTGAGGGTAGGCAGACTATTCGTGGGGGGAGTTACCCACCTGATGCATTGGGAGTTGGTTGTGTCTATCGTATTGCTAAATTTTACACAGGTGAGGTTTGAGGTATGGGTTATTTCCAGATTGGAAACAAGAGGTCCTACTAAAACGGAAGTGGTGTTTATGTGTTGAAGTTGTTCCCTTGTTCAGGTACAGGGATTGAAATGTATGGCCTTAAGTGCAGAGGGAGGCACATCCAACAGTTAGTAGGGTTTTGGGCCGAGACCTCATGAAACCCAGTGAGGGTGGTATTAAATAGGCTTACCAGGCGAGTATGGGTACGGAGGGTTTCATGTAGTTTTGAGAGATCTAGTCCTTTGTAGGGGCTAGGCGTGCTATGTACTCAGGTCAGTTGGGAGATTACTTCCTTTACGTGTTTTTCTCTTGCCTGATCTTGAACTCCACCCCTATCAGACATACCAGTATGGGTGAACTAAGTCCAACAGACAGTGGCTCCAAGTCCTCCAGGACAACTAGGATTAATCATTTTCCCTGTCCAATAATGAGTATTTGCGTGCATGCAAAGAGTGGCAGAGTTATAGCAGTTGTGGGGCATATGGGTGTGGGCAGTGAA

>GL-20-K16_G06

GAGGTACCACAGACAAAAAATATTCCTGAGGGTAGGCAGACTATTCGTGGGGGGGAGTTACCCACCTGATGCATTGGGAGTTGGTTGTGTCTATCGTATTGCTAAATTTTACACAGGTGAGGTTTGAGGTATGGGTTATTTCCAGATTGGAAACAAGAGGTCCTACTAAAACGGAAGTGGTGTTTATGTGTTGAAGTTGTTCCCTTGTTCAGGTACAGGGATTGAAATGTATGGCCTTAAGTGCAGAGGGAGGCACATCCAACAGTTAGTAGGGTTTTGGGCCGAGACCTCATGAAACCCAGTGAGGGTGGTATTAAATAGGCTTACCAGGCGAGTATGGGTACGGAGGGTTTCATGTAGTTTTGAGAGATCTAGTCCTTTGTAGGGGCTAGGCGTGCTATGTACTCAGGTCAGTTGGGAGATTACTTCCTTTACGTGTTTTTCTCTTGCCTGATCTTGAACTCCACCCCTATCAGACATACCAGTATGGGTGAACTAAGTCCAACAGACAGTGGCTCCAAGTCCTCCAGGACAACTAGGATTAATCATTTTCCCTGTCCAATAATGAGTATTTGCATGCATGCAAAGAGTGGCAGAGTTATAGCAGTTGTGGGGCATATGGGTGTGGGCAGTGAA

>GL20-K17_H06

GAGGTACCACAGACAAAAAATATTCCTGAGGGTAGGCAGACTATTTGTGTGGGAGGAGTTACCCACCTGATGCATTGGGAGTTGGTTGTGTATGTAGTATTGCTAAATTTTACACAGGTGAGGTTTGAGGTATGGGTTATTTCCAGATTGGAAACAAGAGGTCCTACTAAAACGGAAGTGGTGTTTATGTGTTGAAGTTGTTCCCTTGTTCAGGTACAGGGATTGAAATGTATGGCCTTAAGTGCAGAGGGAGGCACATCCAACAGTTAGTAGGGTTTTGGGCCGAGACCTCATGAAACCCAGTGAGGGTGGTATTAAATAGGCTTACCAGGCGAGTATGGGTACGGAGGGTTTCATGTAGTTTTGAGAGATCTAGTCCTTTGTAGGGGCTAGGCGTGCTATGTACTCAGGTCAGTTGGGAGATTACTTCCTTTACGTGTTTTTCTCTTGCCTGATCTTGAACTCCACCCCTATCAGACATACCAGTATGGGTGAACTAAGTCCAACAGACAGTGGCTCCAAGTCCTCCAGGACAACTAGGATTAATCATTTTCCCTGTCCAATAATGAGTATTTGCATGCATGCAAAGAGTGGCAGAGTTATAGCAGTCGTGGGGCATATGGGTGTGGGCAGTGAA

>GL-20-K18_A07

GAGGTACCACAGACAAAAAATATTCCTGAGGGTAGGCAGACTATTCGTGGGGGGGAGTTACCCACCTGATGCATTGGGAGTTGGTTGTGTCTATCGTATTGCTAAATTTTACACAGGTGAGGTTTGAGGTATGGGTTATTTCCAGATTGGAAACAAGAGGTCCTATTAAAACGGAAGTGGTGTTTATGTGTTGAAGTTGTTCCCTTGTTCAGGTACAGGGATTGAAATGTATGGCCTTAAGTGCAGAGGGAGGCACATCCAACAGTTAGTAGGGTTTTGGGCCGAGACCTCATGAAACCCAGTGAGGGTGGTATTAAATAGGCTTACCAGGCGAGTATGGGTACGGAGGGTTTCATGTAGTTTTGAGAGATCTAGTCCTTTGTAGGGGCTAGGCGTGCTATGTACTCAGGTCAGTTGGGAGATTACTTCCTTTACGTGTTTTTCTCTTGCCTGATCTTGAACTCCACCCCTATCAGACATACCAGTATGGGTGAACTAAGTCCAACAGACAGTGGCTCCAAGTCCTCCAGGACAACTAGGATTAATCATTTTCCCTGTCCAATAATGAGTATTTGCATGCATGCAAAGAGTGGCAGAGTTATAGCAGTTGTGGGGCATATGGGTGTGGGCAGTGAA

>GL-20-K19_B07

GAGGTACCACAGACAAAAAATATTCCTGAGGGTAGGCAGACTATTCGTGGGGGGGAGTTACCCACCTGATGCATTGGGAGTTAGTTGTGTCTATCGTATTGCTAAATTTTACACAGGTGAGGTTTGAGGTATGGGTTATTTCCAGATTGGAAACAAGAGGTCCTACTAAAACGGAAGTGGTGTTTATGTGTTGAAGTTGTTCCCTTGTTCAGGTACAGGGATTGAAATGTATGGCCTTAAGTGCAGAGGGAGGCACATCCAACAGTTAGTAGGGTTTTGGGCCGAGACCTCATGAAACCCAGTGAGGGTGGTATTAAATAGGCTTACCAGGCGAGTATGGGTACGGAGGGTTTCATGTAGTTTTGAGAGATCTAGTCCTTTGTAGGGGCTAGGCGTGCTATGTACTCAGGTCAGTTGGGAGATTACTTCCTTTACGTGTTTTTCTCTTGCCTGATCTTGAACTCCACCCCTATCAGACATACCAGTATGGGTGAACTAAGTCCAACAGACAGTGGCTCCAAGTCCTCCAGGACAACTAGGATTAATCATTTTCCCTGTCCAATAATGAGTATTTGCATGCATGCAAAGAGTGGCAGAGTTATAGCAGTTGTGGGGCATATGGGTGTGGGCAGTGAA

>GL-20-K20_C07

GAGGTACCACAGACAAAAAATATTCCTGAGGGTAGGCAGACTATTCGTGGGGGGGAGTTACCCACCTGATGCATTGGGAGTTGGTTGTGTCTATCGTATTGCTAAATTTTACACAGGTGAGGTTTGAGGTATGGGTTATTTCCAGATTGGAAACAAGAGGTCCTACTAAAACGGAAGTGGTGTTTATGTGTTGAAGTTGTTCCCTTGTTCAGGTACAGGGATTGAAATGTATGGCCTTAAGTGCAGAGGGAGGCACATCCAACAGTTAGTAGGGTTTTGGGCCGAGACCTCATGAAACCCAGTGAGGGTGGTATTAAATAGGCTTACCAGGCGAGTATGGGTACGGAGGGTTTCATGTAGTTTTGAGAGATCTAGTCCTTTGTAGGGGCTAGGCGTGCTATGTACTCAGGTCAGTTGGGAGATTACTTCCTTTACGTGTTTTTCTCTTGCCTGATCTTGAACTCCACCCCTATCAGACATACCAGTATGGGTGAACTAAGTCCAACAGACAGTGGCTCCAAGTCCTCCAGGACAACTAGGATTAATCATTTTCCCTGTCCAATAATGAGTATTTGCATGCATGCAAAGAGTGGCAGAGTTATAGCAGTTGTGGGGCATATGGGTGTGGGCAGTGAA

>GL-20-K21_D07

GAGGTACCACAGACAAAAAATATTCCTGAGGGTAGGCAGACTATTCGTGGGGGGGAGTTACCCACCTGATGCATTGGGAGTTGGTTGTGTCTATCGTATTGCTAAATTTTACACAGGTGAGGTTTGAGGTATGGGTTATTTCCAGATTGGAAACAAGAGGTCCTACTAAAACGGAAGTGGTGTTTATGTGTTGAAGTTGTTCCCTTGTTCAGGTACAGGGATTGAAATGTATGGCCTTAAGTGCAGAGGGAGGCACATCCAACAGTTAGTAGGGTTTTGGGCCGAGACCTCATGAAACCCAGTGAGGGTGGTATTAAATAGGCTTACCAGGCGAGTATGGGTACGGAGGGTTTCATGTAGTTTTGAGAGATCTAGTCCTTTGTAGGGGCTAGGCGTGCTATGTACTCAGGTCAGTTGGGAGATTACTTCCTTTACGTGTTTTTCTCTTGCCTGATCTTGAACTCCACCCCTATCAGACATACCAGTATGGGTGAACTAAGTCCAACAGACAGTGGCTCCAAGTCCTCCAGGACAACTAGGATTAATCATTTTCCCTGTCCAATAATGAGTATTTGCATGCATGCAAAGAGTGGCAGAGTTATAGCAGTTGTGGGGCATATGGGTGTGGGCAGTGAA

>GL-20-K24_E07

GAGGTACCACAGACAAAAAATATTCCTGAGGGTAGGCAGACTATTCGTGGGGGGGAGTTACCCACCTGATGCATTGGGAGTTGGTTGTGTCTATCGTATTGCTAAATTTTACACAGGTGAGGTTTGAGGTATGGGTTATTTCCAGATTGGAAACAAGAGGTCCTACTAAAACGGAAGTGGTGTTTATGTGTTGAAGTTGTTCCCTTGTTCAGGTACAGGGATTGAAATGTATGGCCTTAAGTGCAGAGGGAGGCACATCCAACAGTTAGTAGGGTTTTGGGCCGAGACCTCATGAAACCCAGTGAGGGTGGTATTAAATAGGCTTACCAGGCGAGTATGGGTACGGAGGGTTTCATGTAGTTTTGAGAGATCTAGTCCTTTGTAGGGGCTAGGCGTGCTATGTACTCAGGTCAGTTGGGAGATTACTTCCTTTACGTGTTTTTCTCTTGCCTGATCTTGAACTCCACCCCTATCAGACATACCAGTATGGGTGAACTAAGTCCAACAGACAGTGGCTCCAAGTCCTCCAGGACAACTAGGATTAATCATTTTCCCTGTCCAATAATGAGTATTTGCATGCATGCAAAGAGTGGCAGAGTTATAGCAGTTGTGGGGCATATGGGTGTGGGCAGTGAA

>GL-20-K25_F07

GAGGTACCACAGACAAAAAATATTCCTGAGGGTAGGCAGACTATTCGTGGGGGGGAGTTACCCACCTGATGCATTGGGAGTTGGTTGTGTCTATCGTATTGCTAAATTTTACACAGGTGAGGTTTGAGGTATGGGTTATTTCCAGATTGGAAACAAGAGGTCCTACTAAAACGGAAGTGGTGTTTATGTGTTGAAGTTGTTCCCTTGTTCAGGTACAGGGATTGAAATGTATGGCCTTAAGTGCAGAGGGAGGCACATCCAACAGTTAGTAGGGTTTTGGGCCGAGACCTCATGAAACCCAGTGAGGGTGGTATTAAATAGGCTTACCAGGCGAGTATGGGTACGGAGGGTTTCATGTAGTTTTGAGAGATCTAGTCCTTTGTAGGGGCTAGGCGTGCTATGTACTCAGGTCAGTTGGGAGATTACTTCCTTTACGTGTTTTTCTCTTGCCTGATCTTGAACTCCACCCCTATCAGACATACCAGTATGGGTGAACTAAGTCCAACAGACAGTGGCTCCAAGTCCTCCAGGACAACTAGGATTAATCATTTTCCCTGTCCAATAATGAGTATTTGCATGCATGCAAAGAGTGGCAGAGTTATAGCAGTTGTGGGGCATATGGGTGTGGGCAGTGAA

>GL-20-K26_G07

GAGGTACCACAGACAAAAAATATTCCTGAGGGTAGGCAGACTATTTGTGTGGGAGGAGTTACCCACCTGATGCATTGGGAGTTGGTTGTGTATGTAGTATTGCTAAATTTTACACAGGTGAGGTTTGAGGTATGGGTTATTTCCAGATTGGAAACAAGAGGTCCTACTAAAACGGAAGTGGTGTTTATGTGTTGAAGTTGTTCCATTGTTCAGGAACAGGGATTGAAATGTACGGCCTGAAGTGCAGGGGGAGGCACATCCAACAGTTAGTAGGGTTTTGGGCTGAGACCTCATGGAGCCGAGTGAGGGTGGTATTAAATAGGCTCACCAGGCGAGTATGGGTACGGAGGGTTTCATGTAGTTTTGAGAGAACTAGTCCTTTGTAGGGGCTAGGGGTGCTATGTCCCCGGGTCAGTTGGGAGATTGCTTCCTTTACTTGTTTTTCTCTTGCCTGACCTTGAATTCCACCCCCATCAGACATACTGGTATGGGTGAAGTAAGTCCAACAGACAGTGGCTCCAAGTCCTCCAGGACAACTAGGATTAATCATTTTCCCTGTCCAATAATGAGTATTTGCATGCATGCAAAGAGTGGCAGAGTTATAGCAGTTGCGGGGCATATGGGTGTGGGCAGTGAA

>GL-20-K27_H07

GAGGTACCACAGACAAAAAATATTCCTGAGGGTAGGCAGACTATTCGTGGGGGGGAGTTACCCACCTGATGCATTGGGAGTTGGTTGTGTCTATCGTATTGCTAAATTTTACACAGGTGAGGTTTGAGGTATGGGTTATTTCCAGATTGGAAACAAGAGGTCCTACTAAAACGGAAGTGGTGTTTATGTGTTGAAGTTGTTCCCTTGTTCAGGTACAGGGATTGAAATGTATGGCCTTAAGTGCAGAGGGAGGCACATCCAACAGTTAGTAGGGTTTTGGGCCGAGACCTCATGAAACCCAGTGAGGGTGGTATTAAATAGGCTTACCAGGCGAGTATGGGTACGGAGGGTTTCATGTAGTTTTGAGAGATCTAGTCCTTTGTAGGGGCTAGGCGTGCTATGTACTCAGGTCAGTTGGGAGATTACTTCCTTTACGTGTTTTTCTCTTGCCTGATCTTGAACTCCACCCCTATCAGACATACCAGTATGGGTGAACTAAGTCCAACAGACAGTGGCTCCAAGTCCTCCAGGACAACTAGGATTAATCATTTTCCCTGTCCAATAATGAGTATTTGCATGCATGCAAAGAGTGGCAGAGTTATAGCAGTTGTGGGGCATATGGGTGTGGGCAGTGAA

>GL-20-K29_A08

TTCACTGCCCACACCCATATGCCCCGCAACTGCTATAACTCTGCCACTCTTTGCATGCATGCAAATACTCATTATTGGACAGGGAAAATGATTAATCCTAGTTGTCCTGGAAGACTTGGAGCCACTGTCTGTCGGACTTACTTCACCCATACTGGTATGTCTGAGGGGGGTGGAGTTCAAGATCAGGCAAGAGAAAAACATGTAAAGGAAGTAACCTCCCAACTGACCCGGGTACATAGCACCCCTAGCCCCTACAAAGGACTAGATCTCTTAAAACTACATGAAACCCTCCATACCCATACTTGCCTGGTAAGCCTATTTAATACCACCCTCACTGGGCTCCATGAGGTCTCGGCCCAAAACCCTACTAACTGTTGGATGTGCCTCCCCCTGTATTTCAGGCCATGCATTTCAATCCCTGTACCTGAACAATGGAACAACTACAGCACAGAAATAAACACCACTTCCGTTTTAGTAGGACCTCTTGTTTCCAATCTGGAAATAACCCATACCTCAAACCTCACCTGTGTAAAATTTAGCAATACTACATACACAACCAACTCCCAATGCATCAGGTGGGTAACTCCTCCCACACAAATAGTCTGCCTACCCTCAGGAATATTTTTTGTCTGTGGTACCTC

>GL-20-K30_B08

GAGGTACCACAGACAAAAAATATTCCTGAGGGTAGGCAGACTATTCGTGGGGGGGAGTTACCCACCTGATGCATTGGGAGTTGGTTGTGTCTATCGTATTGCTAAATTTTACACAGGTGAGGTTTGAGGTATGGGTTATTTCCAGATTGGAAACAAGAGGTCCTACTAAAACGGAAGTGGTGTTTATGTGTTGAAGTTGTTCCCTTGTTCAGGTACAGGGATTGAAATGTATGGCCTTAAGTGCAGAGGGAGGCACATCCAACAGTTAGTAGGGTTTTGGGCCGAGACCTCATGAAACCCAGTGAGGGTGGTATTAAATAGGCTTACCAGGCGAGTATGGGTACGGAGGGTTTCATGTAGTTTTGAGAGATCTAGTCCTTTGTAGGGGCTAGGCGTGCTATGTACTCAGGTCAGTTGGGAGATTACTTCCTTTACGTGTTTTTCTCTTGCCTGATCTTGAACTCCACCCCTATCAGACATACCAGTATGGGTGAACTAAGTCCAACAGACAGTGGCTCCAAGTCCTCCAGGACAACTAGGATTAATCATTTTCCCTGTCCAATAATGAGTATTTGCATGCATGCAAAGAGTGGCAGAGTTATAGCAGTTGTGGGGCATATGGGTGTGGGCAGTGAA

>GL-20-K31_C08

GAGGTACCACAGACAAAAAATATTCCTGAGGGTAGGCAGACTATTCGTGGGGGGGAGTTACCCACCTGATGCATTGGGAGTTGGTTGTGTCTATCGTATTGCTAAATTTTACACAGGTGAGGTTTGAGGTATGGGTTATTTCCAGATTGGAAACAAGAGGTCCTACTAAAACGGAAGTGGTGTTTATGTGTTGAAGTTGTTCCCTTGTTCAGGTACAGGGATTGAAATGTATGGCCTTAAGTGCAGAGGGAGGCACATCCAACAGTTAGTAGGGTTTTGGGCCGAGACCTCATGAAACCCAGTGAGGGTGGTATTAAATAGGCTTACCAGGCGAGTATGGGTACGGAGGGTTTCATGTAGTTTTGAGAGATCTAGTCCTTTGTAGGGGCTAGAGGTGCCATGTACCCGGGTGAGTTGGGAGATTACTTCTTTTACATGTTTTTCTCTTGCCTGATCTTGAACTCCACCCCCATCAGACATACCAGTTTGGGTGAAGTAGGTCCAACAGACAGTGACTCCAAGTCCTCCAGGACAACTAGGATTAATCATTTTTCCTGTCCAATAATGAGTATTTGCATGCATGCAAAGAGTGGCAGAGTGATAGCAGTTGCGGGGCATATGGGTGTGGGCAGTGAA

>GL-20-K32_D08

GAGGTACCACAGACAAAAAATATTCCTGAGGGTAGGCAGACTATTCGTGGGGGGGAGTTACCCACCTGATGCATTGGGAGTTGGTTGTGTCTATCGTATTGCTAAATTTTACACAGGTGAGGTTTGAGGTATGGGTTATTTCCAGATTGGAAACAAGAGGTCCTACTAAAACGGAAGTGGTGTTTATGTGTTGAAGTTGTTCCCTTGTTCAGGTACAGGGATTGAAATGTATGGCCTTAAGTGCAGAGGGAGGCACATCCAACAGTTAGTAGGGTTTTGGGCCGAGACCTCATGAAACCCAGTGAGGGTGGTATTAAATAGGCTTACCAGGCGAGTATGGGTACGGAGGGTTTCATGTAGTTTTGAGAGATCTAGTCCTTTGTAGGGGCTAGAGGTGCCATGTACCCGGGTGAGTTGGGAGATTACTTCTTTTACATGTTTTTCTCTTGCCTGATCTTGAACTCCACCCCCATCAGACATACCAGTTTGGGTGAAGTAAGTCCAACAGACAGTGACTCCAAGTCCTCCAGGACAACTAGGATTAATCATTTTTCCTGTCCAATAATGAGTATTTGCATGCATGCAAAGAGTGGCAGAGTGATAGCAGTTGCGGGGCATATGGGTGTGGGCAGTGAA

>GL-20-K33_E08

GAGGTACCACAGACAAAAAATATTCCTGAGGGTAGGCAGACTATTCGTGGGGGGGAGTTACCCACCTGATGCATTGGGAGTTGGTTGTGTCTATCGTATTGCTAAATTTTACACAGGTGAGGTTTGAGGTATGGGTTATTTCCAGATTGGAAACAAGAGGTCCTACTAAAACGGAAGTGGTGTTTGTGTGTTGAAGTTGTTCCCTTGTTCAGGTACAGGGATTGAAATGTATGGCCTTAAGTGCAGAGGGAGGCACATCCAACAGTTAGTAGGGTTTTGGGCCGAGACCTCATGAAACCCAGTGAGGGTGGTATTAAATAGGCTTACCAGGCGAGTATGGGTACGGAGGGTTTCATGTAGTTTTGAGAGATCTAGTCCTTTGTAGGGGCTAGGCGTGCTATGTACTCAGGTCAGTTGGGAGATTACTTCCTTTACGTGTTTTTCTCTTGCCTGATCTTGAACTCCACCCCTATCAGACATACCAGTATGGGTGAACTAAGTCCAACAGACAGTGGCTCCAAGTCCTCCAGGACAACTAGGATTAATCATTTTCCCTGTCCAATAATGAGTATTTGCATGCATGCAAAGAGTGGCAGAGTTATAGCAGTTGTGGGGCATATGGGTGTGGGCAGTGAA

>GL-20-K34_F08

GAGGTACCACAGACAAAAAATATTCCTGAGGGTAGGCAGACTATTTGTGTGGGAGGAGTTACCCACCTGATGCATTGGGAGTTGGTTGTGTATGTAGTATTGCTAAATTTTACACAGGTGAGGTTTGAGGTATGGGTTATTTCCAGATTGGAAACAAGAGGTCCTACTAAACGGAAGTGGTGTTTATTTCTGTGCTGAAGTTGTTCCATTGTTCAGGTACAGGGATTGAAACATATGGCCTGAAGTTCAGGGGGAGGCATATCCAACAGTTAGTAGGGTTTTGGGCCGAGACCTCATGGAGCCCAGTGAGGGTGGTATTAAATAGGCTTACCAGGCGAGTATGGGTACGGAGGGTTTCATGTAGTTTTGAGAGATCTAGTCCTTTGTAGGGGCTAGAGGTGCCATGTACCCGGGTGAGTTGGGAGATTACTTCTTTTACATGTTTTTCTCTTGCCTGATCTTGAACTCCACCCCCATCAGACATACCAGTTTGGGTGAAGTAAGTCCAACAGACAGTGACTCCAAGTCCTCCAGGACAACTAGGATTAATCATTTTTCCTGTCCAATAATGAGTATTTGCATGCATGCAAAGAGTGGCAGAGTGATAGCAGTTGCGGGGCATATGGGTGTGGGCAGTGAA

>GL-20-K35_G08

GAGGTACCACAGACAAAAAATATTCCTGAGGGTAGGCAGACTATTCGTGGGGGGGAGTTACCCACCTGATGCATTGGGAGTTGGTTGTGTCTATCGTATTGCTAAATTTTACACAGGTGAGGTTTGAGGTATGGGTTATTTCCAGATTGGAAACAAGAGGTCCTATTAAAACGGAAGTGGTGTTTATGTGTTGAAGTTGTTCCCTTGTTCAGGTACAGGGATTGAAATGTATGGCCTTAAGTGCAGAGGGAGGCACATCCAACAGTTAGTAGGGTTTTGGGCCGAGACCTCATGAAACCCAGTGAGGGTGGTATTAAATAGGCTTACCAGGCGAGTATGGGTACGGAGGGTTTCATGTAGTTTTGAGAGATCTAGTCCTTTGTAGGGGCTAGGCGTGCTATGTACTCAGGTCAGTTGGGAGATTACTTCCTTTACGTGTTTTTCTCTTGCCTGATCTTGAACTCCACCCCTATCAGACATACCAGTATGGGTGAACTAAGTCCAACAGACAGTGGCTCCAAGTCCTCCAGGACAACTAGGATTAATCATTTTCCCTGTCCAATAATGAGTATTTGCATGCATGCAAAGAGTGGCAGAGTTATAGCAGTTGTGGGGCATATGGGTGTGGGCAGTGAA

>GL-20-K38_H08

TTCACTGCCCACACCCATATGCCCCACAACTGCTATAACTCTGCCACTCTTTGCATGCATGCAAATACTCATTATTGGACAGGGAAAATGATTAATCCTAGTTGTCCTGGAGGACTTGGAGCCACTGTCTGTTGGACTTAGTTCACCCATACTGGTATGTCTGATAGGGGTGGAGTTCAAGATCAGGCAAGAGAAAAACACGTAAAGGAAGCAATCTCCCAACTGACCTGAGTACATAGCACGCCTAGCCCCTACAAAGGACTAGATCTCTCAAAACTACATGAAACCCTCCGTACCCATACTCGCCTGGTAAGCCTATTTAATACCACCCTCACTGGGTTTCATGAGGTCTCGGCCCAAAACCCTACTAACTGTTGGATGTGCCTCCCTCTGCACTTAAGGCCATACATTTCAATCCCTGTACCTGAACAAGGGAACAACTTCAACACATAAACACCACTTCCGTTTTAGTAGGACCTCTTGTTTCCAATCTGGAAATAACCCATACCTCAAACCTCACCTGTGTAAAATTTAGCAATACGATAGACACAACCAACTCCCAATGCATCAGGTGGGTAACTCCCCCCCACGAATAGTCTGCCTACCCTCAGGAATATTTTTTGTCTGTGGTACCTC

>GL-20-K40_A09

GAGGTACCACAGACAAAAAATATTCCTGAGGGTAGGCAGACTATTCGTGGGGGGGAGTTACCCACCTGATGCATTGGGAGTTGGTTGTGTCTATCGTATTGCTAAATTTTACACAGGTGAGGTTTGAGGTATGGGTTATTTCCAGATTGGAAACAAGAGGTCCTACTAAAACGGAAGTGGTGTTTATGTGTTGAAGTTGTTCCCTTGTTCAGGTACAGGGATTGAAATGTATGGCCTTAAGTGCAGAGGGAGGCACATCCAACAGTTAGTAGGGTTTTGGGCCGAGACCTCATGAAACCCAGTGAGGGTGGTATTAAATAGGCTTACCAGGCGAGTATGGGTACGGAGGGTTTCATGTAGTTTTGAGAGATCTAGTCCTTTGTAGGGGCTAGGCGTGCTATGTACTCAGGTCAGTTGGGAGATTACTTCCTTTACGTGTTTTTCTCTTGCCTGATCTTGAACTCCACCCCTATCAGACATACCAGTATGGGTGAACTAAGTCCAACAGACAGTGGCTCCAAGTCCTCCAGGACAACTAGGATTAATCATTTTCCCTGTCCAATAATGAGTATTTGCATGCATGCAAAGAGTGGCAGAGTTATAGCAGTTGTGGGGCATATGGGTGTGGGCAGTGAA

**MS-IV**

>GL-37_K2_A01

GAGGTACCACAGACAAAAAATATTCCTGAGGGTAGGCAGACTATTCGTGGGGGGGAGTTACCCACCTGATGCATTGGGAGTTGGTTGTGTCTATCGTATTGCTAAATTTTACACAGGTGAGGTTTGAGGTATGGGTTATTTCCAGATTGGAAACAAGAGGTCCTACTAAAACGGAAGTGGTGTTTATGTGTTGAAGTTGTTCCCTTGTTCAGGTACAGGGATTGAAATGTATGGCCTTAAGTGCAGAGGGAGGCACATCCAACAGTTAGTAGGGTTTTGGGCCGAGACCTCATGAAACCCAGTGAGGGTGGTATTAAATAGGCTTACCAGGCGAGTATGGGTACGGAGGGTTTCATGTAGTTTTGAGAGATCTAGTCCTTTGTAGGGGCTAGGCGTGCTATGTACTCAGGTCAGTTGGGAGATTACTTCCTTTACGTGTTTTTCTCTTGCCTGATCTTGAACTCCACCCCTATCAGACATACCAGTATGGGTGAACTAAGTCCAACAGACAGTGGCTCCAAGTCCTCCAGGACAACTAGGATTAATCATTTTCCCTGTCCAATAATGAGTATTTGCATGCATGCAAAGAGTGGCAGAGTTATAGCAGTTGTGGGGGCATATGGGTGTGGGCAGTGAA

>GL-37-K3_B01

GAGGTACCACAGACAAAAAATATTCCTGAGGGTAGGCAGACTATTCGTGTGGGAGGAGTTACCCACCTGATGCATTGGGAGTTGGTTGTGTCTACAGTATTGCTAAATTTTACACAGGTGAGGTTTGAGGTATGGGTTATTTCCAGATTGGAAACAAGAGGTCCTACTAAAACGGAAGTGGTGTTTATTTCTGTGCTGTAGTTGTTCCATTGTTCAGGTACAGGGATTGAAATGCATGGCCTGAAATACAGGGGGAGGCACATCCAACAGTTAGTAGGGTTTTGGGCCGAGACCTCATGGAGCCCAGTGAGGGTGGTATTAAATAGGCTTACCAGGCAAGTATGGGTATGGAGGGTTTCATGTAGTTTTAAGAGATCTAGTCCTTTGTAGGGGCTAGGGGTGCTATGTACCCGGGTCAGTTGGGAGGTTACTTCCTTTACATGTTTTTCTCTTGCCTGATCTTGAACTCCACCCCCCTCAGACATACCAGTATGGGTGAAGTAAGTCCGACAGACAGTGGCTCCAAGTCTTCCAGGACAACTAGGATTAATCATTTTCCCTGTCCAATAATGAGTATTTGCATGCATGCAAAGAGTGGCAGAGTTATAGCAGTTGTGGGGCATATGGGTGTGGGCAGTGAA

>GL-37-K4_C01

GAGGTACCACAGACAAAAAATATTCCTGAGGGTAGGCAGACTATTCGTGTGGGAGGAGTTACCCACCTGATGCATTGGGAGTTGGTTGTGTCTACAGTATTGCTAAATTTTACACAGGTGAGGTTTGAGGTATGGGTTATTTCCAGATTGGAAACAAGAGGTCCTACTAAAACGGAAGTGGTGTTTATTTCTGTGCTGTAGTTGTTCCATTGTTCAGGTACAGGGATTGAAATGCATGGCCTGAAATACAGGGAGAGGCACATCCAACAGTTAGTAGGGTTTTGGGCCGAGACCTCATGGAGCCCAGTGAGGGTGGTATTAAATAGGCTTACCAGGCAAGTATGGGTATGGAGGGTTTCATGTAGTTTTAAGAGATCTAGTCCTTTGTAGGGGCTAGGGGTGCTATGTACCCGGGTCAGTTGGGAGGTTACTTCCTTTACATGTTTTTCTCTTGCCTGATCTTGAACTCCACCCCCCTCAGACATACCAGTATGGGTGAAGTAAGTCCGACAGACAGTGGCTCCAAGTCTTCCAGGACAACTAGGATTAATCATTTTCCCTGTCCAATAATGAGTATTTGCATGCATGCAAAGAGTGGCAGAGTTATAGCAGTTGTGGGGCATATGGGTGTGGGCAGTGAA

>GL-37-K5_D01

GAGGTACCACAGACAAAAAATATTCCTGAGGGTAGGCAGACTATTCGTGGGGGGGAGTTACCCACCTGATGCATTGGGAGTTGGTTGTGTCTATCGTATTGCTAAATTTTACACAGGTGAGGTTTGAGGTATGGGTTATTTCCAGATTGGAAACAAGAGGTCCTACTAAAACGGAAGTGGTGTTTATGTGTTGAAGTTGTTCCCTTGTTCAGGTACAGGGATTGAAATGTATGGCCTTAAGTGCAGAGGGAGGCACATCCAACAGTTAGTAGGGTTTTGGGCCGAGACCTCATGAAACCCAGTGAGGGTGGTATTAAATAGGCTTACCAGGCGAGTATGGGTACGGAGGGTTTCATGTAGTTTTGAGAGATCTAGTCCTTTGTAGGGGCTAGGCGTGCTATGTACTCAGGTCAGTTGGGAGATTACTTCCTTTACGTGTTTTTCTCTTGCCTGATCTTGAACTCCACCCCTATCAGACATACCAGTATGGGTGAACTAAGTCCAACAGACAGTGGCTCCAAGTCCTCCAGGACAACTAGGATTAATCATTTTCCCTGTCCAATAATGAGTATTTGCATGCATGCAAAGAGTGGCAGAGTTATAGCAGTTGTGGGGCATATGGGTGTGGGCAGTGAA

>GL-37-K7_E01

GAGGTACCACAGACAAAAAATATTCCTGAGGGTAGGCAGACTATTCGTGTGGGAGGAGTTACCCACCTGATGCATTGGGAGTTGGTTGTGTCTACAGTATTGCTAAATTTTACACAGGTGAGGTTTGAGGTATGGGTTATTTCCAGATTGGAAACAAGAGGTCCTACTAAAACGGAAGTGGTGTTTATTTCTGTGCTGTAGTTGTTCCATTGTTCAGGTACAGGGATTGAAATGCATGGCCTGAAATACAGGGGGAGGCACATCCAACAGTTAGTAGGGTTTTGGGCCGAGACCTCATGGAGCCCAGTGAGGGTGGTATTAAATAGGCTTACCAGGCAAGTATGGGTATGGAGGGTTTCATGTAGTTTTAAGAGATCTAGTCCTTTGTAGGGGCTAGGGGTGCTATGTACCCGGGTCAGTTGGGAGGTTACTTCCTTTACATGTTTTTCTCTTGCCTGATCTTGAACTCCACCCCCCTCAGACATACCAGTATGGGTGAAGTAAGTCCGACAGACAGTGGCTCCAAGTCTTCCAGGACAACTAGGATTAATCATTTTCCCTGTCCAATAATGAGTATTTGCATGCATGCAAAGAGTGGCAGAGTTATAGCAGTTGCGGGGCATATGGGTGTGGGCAGTGAA

>GL-37-K8_F01

GAGGTACCACAGACAAAAAATATTCCTGAGGGTAGGCAGACTATTCGTGTGGAAGGAGTTACCCACCTGATGCATTGGGAGTTGGTTGTGTCTACAGTATTGCTAAATTTTACACAGGTGAGGTTTGAGGTATGGGTTATTTCCAGATTGGAAACAAGAGGTCCTACTAAAACGGAAGTGGTGTTTATTTCTGTGCTGTAGTTGTTCCATTGTTCAGGTACAGGGATTGAAATGCATGGCCTGAAATACAGGGGGAGGCACATCCAACAGTTAGTAGGGTTTTGGGCCGAGACCTCATGGAGCCCAGTGAGGGTGGTATTAAATAGGCTTACCAGGCAAGTATGGGTATGGAGGGTTTCATGTAGTTTTAAGAGATCTAGTCCTTTGTAGGGGCTAGGGGTGCTATGTACCCGGGTCAGTTGGGAGGTTACTTCCTTTACATGTTTTTCTCTTGCCTGATCTTGAACTCCACCCCCCTCAGACATACCAGTATGGGTGAAGTAAGTCCGACAGACAGTGGCTCCAAGTCTTCCAGGACAACTAGGATTAATCATTTTCCCTGTCCAATAATGAGTATTTGCATGCATGCAAAGAGTGGCAGAGTTATAGCAGTTGTGGGGCATATGGGTGTGGGCAGTGAA

>GL-37-K9_G01

GAGGTACCACAGACAAAAAATATTCCTGAGGGTAGGCAGACTATTCGTGGGGGGGAGTTACCCACCTGATGCATTGGGAGTTGGTTGTGTCTATCGTATTGCTAAATTTTACACAGGTGAGGTTTGAGGTATGGGTTATTTCCAGATTGGAAACAAGAGGTCCTACTAAAACGGAAGTGGTGTTTATGTGTTGAAGTTGTTCCCTTGTTCAGGTACAGGGATTGAAATGTATGGCCTTAAGTGCAGGGGGAGGCACATCCAACAGTTAGTAGGGTTTTGGGCCGAGACCTCATGAAACCCAGTGAGGGTGGTATTAAATAGGCTTACCAGGCGAGTATGGGTACGGAGGGTTTCATGTAGTTTTGAGAGATCTAGTCCTTTGTAGGGGCTAGGCGTGCTATGTACTCAGGTCAGTTGGGAGATTACTTCCTTTACGTGTTTTTCTCTTGCCTGATCTTGAACTCCACCCCTATCAGACATACCAGTATGGGTGAACTAAGTCCAACAGACAGTGGCTCCAAGTCCTCCAGGACAACTAGGGTTAATCATTTTCCCTGTCCAATAATGAGTATTTGCATGCATGCAAAGAGTGGCAGAGTTATAGCAGTTGTGGGGCATATGGGTGTGGGCAGTGAA

>GL-37-K10_H01

TTCACTGCCCACACCCATATGCCCCGCAACTGCTATAACTCTGCCACTCTTTGCATGCATGCAAATACTCATTATTGGACAGGGAAAATGATTAATCCTAGTTGTCCTGGAGGACTTGGAGCCACTGTCTGTTGGACTTACTTCACCCATACCAGTATGTCTGATGGGGGTGGAATTCAAGGTCAGGCAAGAGAAAAACAAGTAAAGGAAGCAATCTCCCAACTGACCCGGGGACATAGCACCCCTAGCCCCTACAAAGGACTAGTTCTCTCAAAACTACATGAAACCCTCCGTACCCATACTCGCCTGGTGAGCCTATTTAATACCACCCTCACTCGGCTCCATGAGGTCTCAGCCCAAAACCCTACTAACTGTTGGATGTGCCTCCCCCTGCACTTCAGGCCATACATTTCAATCCCTGTTCCTGAACAATGGAACAACTTCAGCACAGAAATAAACACCACTTCCGTTTTAGTAGGACCTCTTGTTTCCAATCTGGAAATAACCCATACCTCAAACCTCACCTGTGTAAAATTTAGCAATACTGTAGACACAACCAACTCCCAATGCATCAGGTGGGTAACTCCTCCCACACGAATAGTCTGCCTACCCTCAGGAATATTTTTTGTCTGTGGTACCTC

>GL-37-K12_B02

GAGGTACCACAGACAAAAAATATTCCTGAGGGTAGGCAGACTATTCGTGTGGGAGGTGTTACCCACCTGATGCATTGGGAGCTGGTTGTGTCTATAGTATTGCTAAATTTTACACAGGTGAGGTTTGAGGTATGGGTTATTTCCAGATTGGAAACAAGAGGTCCTACTAAAACGGAAGTGGTGTTTATTTCTGTGCTGAAGTTGTTCCATTGTTCAGGAACAGGGATTGAAATGTATGGCCTGAAGTGCAGGGGGAGGCACATCCAACAGTTAGTAGGGTTTTGGGCTGAGACCTCATGGAGCCGAGTGAGGGTGGTATTAAATAGGCTCACCAGGCGAGTATGGGTACGGAGGGTTTCATGTAGTTTTGAGAGAACTAGTCCCTTGTAGGGGCTAGGGGTGCTATGTCCCCGGGTCAGTTGGGAGATTGCTTCCTTTACTTGTTTTTCTCTTGCCTGACCTTGAATTCCACCCCCATCAGACATACTGGTATGGGTGAAGTAAGTCCAACAGACAGTGGCTCCAAGTCCTCCAGGACAACTAGGATTAATCATTTTCCCTGTCCAATAATGAGTATTTGCATGCATGCAAAGAGTGGCAGAGTTATAGCAGTTGCGGGGCATATGGGTGTGGGCAGTGAA

>GL-37-K15_D02

TTCACTGCCCACACCCATATGCCCCACAACTGCTATAACTCTGCCACTCTTTGCATGCATGCAAATACTCATTATTGGACAGGGAAAATGATTAATCCTAGTTGTCCTGGAGGACTTGGAGCCACTGTCTGTTGGACTTAGTTCACCCATACTGGTATGTCTGATAGGGGTGGAGTTCAAGATCAGGCAAGAGAAAAACACGTAAAGGAAGTAATCTCCCAACTGACCTGAGTACATAGCACGCCTAGCCCCTACAAAGGACTAGATCTCTCAAAACTACATGAAACCCTCCGTACCCATACTCGCCTGGTAAGCCTATTTAATACCACCCTCACTGGGTTTCATGAGGTCTCGGCCCAAAACCCTACTAACTGTTGGATGTGCCTCCCTCTGCACTTAAGGCCATACATTTCAATCCCTGTACCTGAACAAGGGAACAACTTCAACACATAAACACCACTTCCGTTTTAGTAGGACCTCTTGTTTCCAATCTGGAAATAACCCATACCTCAAACCTCACCTGTGTAAAATTTAGCAATACTATAGACACAACCAGCTCCCAATGCATCAGGTGGGTAGCACCTCCCACACGAATAGTCTGCCTACCCTCAGGAATATTTTTTGTCTGTGGTACCTC

>GL-37-K18_F02

GAGGTACCACAGACAAAAAATATTCCTGAGGGTAGGCAGACTATTCGTGTGGGAGGAGTTACCCACCTGATGCATTGGGAGTTGGTTGTGTCTACAGTATTGCTAAATTTTACACAGGTGAGGTTTGAGGTATGGGTTATTTCCAGATTGGAAACAAGAGGTCCTACTAAAACGGAAGTGGTGTTTATTTCTGTGCTGTAGTTGTTCCATTGTTCAGGTACAGGGATTGAAATGCATGGCCTGAAATACAGGGGGAGGCACATCCAACAGTTAGTAGGGTTTTGGGCCGAGACCTCATGGAGCCCAGTGAGGGTGGTATTAAATAGGCTTACCAGGCAAGTATGGGTATGGAGGGTTTCATGTAGTTTTAAGAGATCTAGTCCTTTGTAGGGGCTAGGGGTGCTATGTACCCGGGTCAGTTGGGAGGTTACTTCCTTTACATGTTTTTCTCTTGCCTGATCTTGAACTCCACCCCCCTCAGACATACCAGTATGGGTGAAGTAAGTCCGACAGACAGTGGCTCCAAGTCTTCCAGGACAACTAGGATTAATCATTTTCCCTGTCCAATAATGAGTATTTGCATGCATGCAAAGAGTGGCAGAGTTATAGCAGTTGTGGGGCATATGGGTGTGGGCAGTGAAACACGC

>GL-37-K19_G02

GAGGTACCACAGACAAAAAATATTCCTGAGGGTAGGCAGACTATTCGTGTGGGAGGAGTTACCCACCTGATGCATTGGGAGTTGGTTGTGTCTACAGTATTGCTAAATTTTACACAGGTGAGGTTTGAGGTATGGGTTATTTCCAGATTGGAAACAAGAGGTCCTACTAAAACGGAAGTGGTGTTTATTTCTGTGCTGTAGTTGTTCCATTGTTCAGGTACAGGGATTGAAATGCATGGCCTGAAATACAGGGGGAGGCACATCCAACAGTTAGTAGGGTTTTGGGCCGAGACCTCATGGAGCCCAGTGAGGGTGGTATTAAATAGGCTTACCAGGCAAGTATGGGTATGGAGGGTTTCATGTAGTTTTAAGAGATCTAGTCCTTTGTAGGGGCTAGGGGTGCTATGTACCCGGGTCAGTTGGGAGGTTACTTCCTTTACATGTTTTTCTCTTGCCTGATCTTGAACTCCACCCCCCTCAGACATACCAGTATGGGTGAAGTAAGTCCGACAGACAGTGGCTCCAAGTCTTCCAGGACAACTAGGATTAATCATTTTCCCTGTCCAATAATGAGTATTTGCATGCATGCAAAGAGTGGCAGAGTTATAGCAGTTGTGGGGCATATGGGTGTGGGCAGTGAA

>GL-37-K20_H02

GAGGTACCACAGACAAAAAATATTCCTGAGGGTAGGCAGACTATTCGTGTGGGAGGAGTTACCCACCTGATGCATTGGGAGTTGGTTGTGTCTACAGTATTGCTAAATTTTACACAGGTGAGGTTTGAGGTATGGGTTATTTCCAGATTGGAAACAAGAGGTCCTACTAAAACGGAAGTGGTGTTTATTTCTGTGCTGTAGTTGTTCCATTGTTCAGGTACAGGGATTGAAATGCATGGCCTGAAATACAGGGGGAGGCACATCCAACAGTTAGTAGGGTTTTGGGCCGAGACCTCATGGAGCCCAGTGAGGGTGGTATTAAATAGGCTTACCAGGCAAGTATGGGTATGGAGGGTTTCATGTAGTTTTAAGAGATCTAGTCCTTTGTAGGGGCTAGGGGTGCTATGTACCCGGGTCAGTTGGGAGGTTACTTCCTTTACATGTTTTTCTCTTGCCTGATCTTGAACTCCACCCCCCTCAGACATACCAGTATGGGTGAAGTAAGTCCGACAGACAGTGGCTCCAAGTCTTCCAGGACAACTAGGATTAATCATTTTCCCTGTCCAATAATGAGTATTTGCATGCATGCAAAGAGTGGCAGAGTTATAGCAGTTGTGGGGCATATGGGTGTGGGCAGTGAA

>GL-37-K21_A03

GAGGTACCACAGACAAAAAATATTCCTGAGGGTAGGCAGACTATTCGTGTGGGAGGAGTTACCCACCTGATGCATTGGGAGTTGGTTGTGTCTACAGTATTGCTAAATTTTACACAGGTGAGGTTTGAGGTATGGGTTATTTCCAGATTGGAAACAAGAGGTCCTACTAAAACGGAAGTGGTGTTTATTTCTGTGCTGTAGTTGTTCCATTGTTCAGGTACAGGGATTGAAATGCATGGCCTGAAATACAGGGGGAGGCACATCCAACAGTTAGTAGGGTTTTGGGCCGAGACCTCATGGAGCCCAGTGAGGGTGGTATTAAATAGGCTTACCAGGCAAGTATGGGTATGGAGGGTTTCATGTAGTTTTAAGAGATCTAGTCCTTTGTAGGGGCTAGGGGTGCTATGTACCCGGGTCAGTTGGGAGGTTACTTCCTTTACATGTTTTTCTCTTGCCTGATCTTGAACTCCACCCCCCTCAGACATACCAGTATGGGTGAAGTAAGTCCGACAGACAGTGGCTCCAAGTCTTCCAGGACAACTAGGATTAATCATTTTCCCTGTCCAATAATGAGTATTTGCATGCATGCAAAGAGTGGCAGAGTTATAGCAGTTGTGGAGCATATGGGTGTGGGCAGTGAA

>GL-37-K23_B03

GAGGTACCACAGACAAAAAATATTCCTGAGGGTAGGCAGACTATTCGTGTGGGAGGAGTTACCCACCTGATGCATTGGGAGTTGGTTGTGTCTACAGTATTGCTAAATTTTACACAGGTGAGGTTTGAGGTATGGGTTATTTCCAGATTGGAAACAAGAGGTCCTACTAAAACGGAAGTGGTGTTTATTTCTGTGCTGTAGTTGTTCCATTGTTCAGGTACAGGGATTGAAATGCATGGCCTGAAATACAGGGGGAGGCACATCCAACAGTTAGTAGGGTTTTGGGCCGAGACCTCATGGAGCCCAGTGAGGGTGGTATTAAATAGGCTTACCAGGCAAGTATGGGTATGGAGGGTTTCATGTAGTTTTAAGAGATCTAGTCCTTTGTAGGGGCTAGGGGTGCTATGTACCCGGGTCAGTTGGGAGGTTACTTCCTTTACATGTTTTTCTCTTGCCTGATCTTGAACTCCACCCCCCTCAGACATACCAGTATGGGTGAAGTAAGTCCGACAGACAGTGGCTCCAAGTCTTCCAGGACAACTAGGATTAACCATTTTCCCTGTCCAATAATGAGTATTTGCATGCATGCAAAGAGTGGCAGAGTTATAGCAGTTGTGGGGCATATGGGTGTGGGCAGTGAA

>GL-37-K25_C03

GAGGTACCACAGACAAAAAATATTCCTGAGGGTAGGCAGACTATTCGTGTGGGAGGAGTTACCCACCTGATGCATTGGGAGTTGGTTGTGTCTACAGTATTGCTAAATTTTACACAGGTGAGGTTTGAGGTATGGGTTATTTCCAGATTGGAAACAAGAGGTCCTACTAAAACGGAAGTGGTGTTTATTTCTGTGCTGTAGTTGTTCCATTGTTCAGGTACAGGGATTGAAATGCATGGCCTGAAATACAGGGGGAGGCACATCCAACAGTTAGTAGGGTTTTGGGCCGAGACCTCATGGAGCCCAGTGAGGGTGGTATTAAATAGGCTTACCAGGCAAGTATGGGTATGGAGGGTTTCATGTAGTTTTAAGAGATCTAGTCCTTTGTAGGGGCTAGGGGTGCTATGTACCCGGGTCAGTTGGGAGGTTACTTCCTTTACATGTTTTTCTCTTGCCTGATCTTGAACTCCACCCCCCTCAGACATACCAGTATGGGTGAAGTAAGTCCGACAGACAGTGGCTCCAAGTCTTCCAGGACAACTAGGATTAATCATTTTCCCTGTCCAATAATGAGTATTTGCATGCATGCAAAGAGTGGCAGAGTTATAGCAGTTGTGGGGCATATGGGTGTGGGCAGT

>GL-37-K26_D03

GAGGTACCACAGACAAAAAATATTCCTGAGGGTAGGCAGACTATTCGTGTGGGAGGAGTTACCCACCTGATGCATTGGGAGTTGGTTGTGTCTATAGTATTGCTAAATTTTACACAGGTGAGGTTTGAGGTATGGGTTATTTCCAGATTGGAAAGAGGACCTACTAAAACAGAAGTGGTGTTTATTTCTGTGCTGAAGTTGTTCCATTGTTCAGGTATAGGGATTGAAATGTGTGGCCTAAAGTGCAGGGGGAGGCACATCCAACAGTTAGTAGGGTTTTGGGCCGAGACCTCATGGAGCCCAGTCAGGGTGGTATTAAATAGGCTTACCAGGCCAGTATGGGTATGGAGGGTTTCATGTAGTTTTGAGAGATCTAGTCCTTTGTAGGGGCCAGGGGTGCTATGTACCCAGGTCAGTTGGGAGATTACTTCCTTTATGTGTTTTTCTGTTGCCTGATCTTGAACTCCACCCCCATCAGACATACTGGTATGGGTGAAGTAAGTCCGACAGACAGTGGCTCCAAGTCTTCCAGGACAACTAGGATTAATCATTTTCCCTGTCCAATAATGAGTATTTGCATGCATGCAAAGAGTGGCAGAGTTATAGCAGTTGTGGGGCATATGGGTGTGGGCAGTGAA

>GL-37-K29_F03

GAGGTACCACAGACAAAAAATATTCCTGAGGGTAGGCAGACTATTTGTGTGGGAGGAGTTACCCATCTGATGCACTGGGAGTTGGTTGTGTCTATAGTATTGCTAAATTTTACACGGGTGAGGTTTGAGGTATGGGTTATTTCCAGATTGGAAACAAGAGGTCCTACTAAAATGGAAGTGGTGTTTATTTCTGTGCTGAAGTTGTTCCATTGTTCAGGTACAGGGATTGAAATATGTGGCCTGTAGTGCAGGGGGAGGCACATTCAACAGTTAGTAGGGTTTTGGGCCAAGGCCTCATGGAGCCCAGTGAGGGTGGTATTAAATAGGCTTACCTGGTGAGTATGGGTATGGAGGGTTTCATGTAGTTTT

>GL-37-K30_G0

GAGGTACCACAGACAAAAAATATTCCTGAGGGTAGGCAGACTATTCGTGGGGGGGAGTTACCCACCTGATGCATTGGGAGTTGGTTGTGTCTATCGTATTGCTAAATTTTACACAGGTGAGGTTTGAGGTATGGGTTATTTCCAGATTGGAAACAAGAGGTCCTACTAAAACGGAAGTGGTGTTTATGTGTTGAAGTTGTTCCCTTGTTCAGGTACAGGGATTGAAATGTATGGCCTTAAGTGCAGAGGGAGGCACATCCAACAGTTAGTAGGGTTTTGGGCCGAGACCTCATGAAACCCAGTGAGGGTGGTATTAAATAGGCTTACCAGGCGAGTATGGGTACGGAGGGTTTCATGTAGTTTTGAGAGATCTAGTCCTTTGTAGGGGCTAGGCGTGCTATGTACTCAGGTCAGTTGGGAGATTACTTCCTTTACGTGTTTTTCTCTTGCCTGATCTTGAACTCCACCCCAATCAGACATACCAGTATGGGTGAACTAAGTCCAACAGACAGTGGCTCCAAGTCCTCCAGGACAACTAGGATTAATCATTTTCCCTGTCCAATAATGAGTATTTGCATGCATGCAAAGAGTGGCAGAGTTATAGCAGTTGTGGGGCATATGGGTGTGGGCAGTGAA

>GL-37-K31_H03

GAGGTACCACAGACAAAAAATATTCCTGAGGGTAGGCAGACTATTCGTGTGGGAGGAGTTACCCACCTGATGCATTGGGAGTTGGTTGTGTCTACAGTATTGCTAAATTTTACACAGGTGAGGTTTGAGGTATGGGTTATTTCCAGATTGGAAACAAGAGGTCCTACTAAAACGGAAGTGGTGTTTATTTCTGTGCTGTAGTTGTTCCATTGTTCAGGTACAGGGATTGAAATGCATGGCCTGAAATACAGGGGGAGGCACATCCAACAGTTAGTAGGGTTTTGGGCCGAGACCTCATGGAGCCCAGTGAGGGTGGTATTAAATAGGCTTACCAGGCAAGTATGGGTATGGAGGGTTTCATGTAGTTTTAAGAGATCTAGTCCTTTGTAGGGGCTAGGGGTGCTATGTACCCGGGTCAGTTGGGAGGTTACTTCCTTTACATGTTTTTTCTCTTGCCTGATCTTGAACTCCACCCCCCTCAGACATACCAGTATGGGTGAAGTAAGTCCGACAGACAGTGGCTCCAAGTCTTCCAGGACAACTAGGATTAATCATTTTCCCTGTCAATAATGAGTATTTTGCATGCATGCAAGAGTGGCAGAGTTATAGCAGTGGGGGCATATTGGGTGTGGGCAGTGAA

>GL-37-K33_A04

GAGGTACCACAGACAAAAAATATTCCTGAGGGTAGGCAGACTATTCGTGTGGGAGGAGTTACCCACCTGATGCATTGGGAGTTGGTTGTGTCTACAGTATTGCTAAATTTTACACAGGTGAGGTTTGAGGTATGGGTTATTTCCAGATTGGAAACAAGAGGTCCTACTAAAACGGAAGTGGTGTTTATTTCTGTGCTGTAGTTGTTCCATTGTTCAGGTACAGGGATTGAAATGCATGGCCTGAAATACAGGGGGAGGCACATCCAACAGTTAGTAGGGTTTTGGGCCGAGACCTCATGGAGCCCAGTGAGGGTGGTATTAAATAGGCTTACCAGGCAAGTATGGGTATGGAGGGTTTCATGTAGTTTTAAGAGATCTAGTCCTTTGTAGGGGCTAGGGGTGCTATGTACCCGGGTCAGTTGGGAGGTTACTTCCTTTACATGTTTTTCTCTTGCCTGATCTTGAACTCCACCCCCCTCAGACATACCAGTATGGGTGAAGTAAGTCCGGCAGACAGTGGCTCCAAGTCTTCCAGGACAACTAGGATTAATCATTTTCCCTGTCCAATAATGAGTATTTGCATGCATGCAAAGAGTGGCAGAGTTATAGCAGTTGTGGGGCATATGGGTGTGGGCAGTGAA

>GL-37-K34_B04

GAGGTACCACAGACAAAAAATATTCCTGAGGGTAGGCAGACTATTCGTGTGGGAGGAGTTACCCACCTGATGCATTGGGAGTTGGTTGTGTCTACAGTATTGCTAAATTTTACACAGGTGAGGTTTGAGGTATGGGTTATTTCCAGATTGGAAACAAGAGGTCCTACTAAAACGGAAGTGGTGTTTATTTCTGTGCTGTAGTTGTTCCATTGTTCAGGTACAGGGATTGAAATGCATGGCCCTGAAATAC

>GL-37-K35_C04

GAGGTACCACAGACAAAAAATATTCCTGAGGGTAGGCAGACTATTCGTGTGGGAGGAGTTACCCACCTGATGCATTGGGAGTTGGTTGTGTCTACAGTATTGCTAAATTTTACACAGGTGAGGTTTGAGGTATGGGTTATTTCCAGATTGGAAACAAGAGGTCCTACTAAAACGGAAGTGGTGTTTATTTCTGTGCTGTAGTTGTTCCATTGTTCAGGTACAGGGATTGAAATGCATGGCCTGAAATACAGGGGGAGGCACATCCAACAGTTAGTAGGGTTTTGGGCCGAGACCTCATGGAGCCCAGTGAGGGTGGTATTAAATAGGCTTACCAGGCAAGTATGGGTATGGAGGGTTTCATGTAGTTTTAAGAGATCTAGTCCTTTGTAGGGGCTAGGGGTGCTATGTACCCGGGTCAGTTGGGAGGTTACTTCCTTTACATGTTTTTCTCTTGCCTGATCTTGAACTCCACCCCCCTCAGACATACCAGTATGGGTGAAGTAAGTCCGACAGACAGTGGCTCCAAGTCGTCCAGGACAACTAGGATTAATCATTTTCCCTGTCCAATAATGAGTATTTGCATGCATGCAAAGAGTGGCAGAGTTATAGCAGTTGTGGGGCATATGGGTGTGGGCAGTGAA

>GL-37-K36_D04

GAGGTACCACAGACAAAAAATATTCCTGAGGGTAGGCAGACTATTCGTGTGGGAGGAGTTACCCACCTGATGCATTGGGAGTTGGTTGTGTCTACAGTATTGCTAAATTTTACACAGGTGAGGTTTGAGGTATGGGTTATTTCCAGATTGGAAACAAGAGGTCCTACTAAAACGGAAGTGGTGTTTATTTCTGTGCTGTAGTTGTTCCATTGTTCAGGTACAGGGATTGAAATGCATGGCCTGAAATACAGGGGGAGGCACATCCAACAGTTAGTAGGGTTTTGGGCCGAGACCTCATGGAGCCCAGTGAGGGTGGTATTAAATAGGCTTACCAGGCAAGTATGGGTATGGAGGGTTTCATGTAGTTTTAAGAGATCTAGTCCTTTGTAGGGGCTAGGGGTGCTATGTACCCGGGTCAGTTGGGAGGTTACTTCCTTTACATGTTTTTCTCTTGCCTGATCTTGAACTCCACCCCCCTCAGACATACCAGTATGGGTGAAGTAAGTCCGACAGACAGTGGCTCCAAGTCTTCCAGGACAACTAGGATTAATCATTTTCCCTGTCCAATAATGAGTATTTGCATGCATGCAAAGAGTGGCAGAGTTATAGCAGTTGTGGGGCATATGGGTGTGGGCAGTGAA

>GL-37-K39_E04

GAGGTACCACAGACAAAAAATATTCCTGAGGGTAGGCAGACTATTCGTGTGGGAGGAGTTACCCACCTGATGCATTGGGAGTTGGTTGTGTCTACAGTATTGCTAAATTTTACACAGGTGAGGTTTGAGGTATGGGTTATTTCCAGATTGGAAACAAGAGGTCCTACTAAAACGGAAGTGGTGTTTATTTCTGTGCTGTAGTTGTTCCATTGTTCAGGTACAGGGATTGAAATGCATGGCCTGAAATACAGGGGGAGGCACATCCAACAGTTAGTAGGGTTTTGGGCCGAGACCTCATGGAGCCCAGTGAGGGTGGTATTAAATAGGCTTACCAGGCAAGTATGGGTATGGAGGGTTTCATGTAGTTTTAAGAGATCTAGTCCTTTGTAGGGGCTAGGGGTGCTATGTACCCCGGGTCAGTTGGGAGGTTACTTCCTTTACATGTTTTTCTCTTGCCTGATCTTGAACTCCACCCCCCTCAGAC

>GL-37-K43_F04

GAGGTACCACAGACAAAAAATATTCCTGAGGGTAGGCAGACTATTCGTGTGGGAGGAGTTACCCACCTGATGCATTGGGAGTTGGTTGTGTCTACAGTATTGCTAAATTTTACACAGGTGAGGTTTGAGGTATGGGTTATTTCCAGATTGGAAACAAGAGGTCCTACTAAAACGGAAGTGGTGTTTATTTCTGTGCTGTAGTTGTTCCATTGTTCAGGTACAGGGATTGAAATGCATGGCCTGAAATACAGGGGGAGGCACATCCAACAGTTAGTAGGGTTTTGGGCCGAGACCTCATGGAGCCCAGTGAGGGTGGTATTAAATAGGCTTACCAGGCAAGTATGGGTATGGAGGGTTTCATGTAGTTTTAAGAGATCTAGTCCTTTGTAGGGGCTAGGGGTGCTATGTACCCGGGTCAGTTGGGAGGTTACTTCCTTTACATGTTTTTCTCTTGCCTGATCTTGAACTCCACCCCCCTCA

>GL-37-K44_G04

GAGGTACCACAGACAAAAAATATTCCTGAGGGTAGGCAGACTATTCGTGTGGGAGGAGTTACCCACCTGATGCATTGGGAGTTGGTTGTGTCCACAGTATTGCTAAATTTTACACAGGTGAGGTTTGAGGTATGGGTTATTTCCAGATTGGAAACAAGAGGTCCTACTAAAACGGAAGTGGTGTTTATTTCCGTGCTGTAGTTGTTCCATTGTTCAGGTACAGGGATTGAAATGCATGGCCTGAAATACAGGGGGAGGCACATCCAACAGTTAGTAGGGTTTTGGGCCGAGACCTCATGGAGCCCAGTGAGGGTGGTATTAAATAGGCTTACCAGGCAAGTATGGGTATGGAGGGTTTCATGTAGTTTTAAGAGATCTAGTCCTTTGTAGGGGCTAGGGGTGCTATGTACCCGGGTCAGTTGGGAGGTTACTTCCTTTACATGTTTTCCTCTTGCCTGATCTTGAACTCCACCCCCCTCAGACATACCAGTATGGGTGAAGTAAGTCCGACAGACAGTGGCTCCAAGTCTTCCAGGACAACTAGGATTAATCATTTTCCCTGTCCAATAATGAGTATTTGCATGCATGCAAAGAGTGGCAGAGTTATAGCAGTTGTGGGGCATATGGGTGTGGGCAGTGAA

>GL-37-K47_H04

GAGGTACCACAGACAAAAAATATTCCTGAGGGTAGGCAGACTATTCGTGTGGGAGGAGTTACCCACCTGATGCATTGGGAGTTGGTTGTGTCTACAGTATTGCTAAATTTTACACAGGTGAGGTTTGAGGTATGGGTTATTTCCAGATTGGAAACAAGAGGTCCTACTAAAACGGAAGTGGTGTTTATTTCTGTGCTGTAGTTGTTCCATTGTTCAGGTACAGGGATTGAAATGCATGGCCTGAAATACAGGGGGAGGCACATCCAACAGTTAGTAGGGTTTTGGGCCGAGACCTCATGGAGCCCAGTGAGGGTGGTATTAAATAGGCTTACCAGGCAAGTATGGGTATGGAGGGTTTCATGTAGTTTTAAGAGATCTAGTCCTTTGTAGGGGCTAGGGGTGCTATGTACCCGGGTCAGTTGGGAGGTTACTTCCTTTACATGTTTTTCTCTTGCCTGATCTTGAACTCCACCCCCCTCAGACATACCAGTATGGGTGAAGTAAGTCCGACAGACAGTGGCTCCAAGTCTTCCAGGACAACTAGGATTAATCATTTTCCCTGTCCAATAATGAGTATTTGCATGCATGCAAAGAGTGGCAGAGTTATAGCAGTTGTGGGGCATATGGGTGTGGGCAGTGAA

>GL-37-K49_B05

GAGGTACCACAGACAAAAAATATTCCTGAGGGTAGGCAGACTATTCGTGTGGGAGGTGTTACCCACCTGATGCATTGGGAGCTGGTTGTGTCTATAGTATTGCTAAATTTTACACAGGTGAGGTTTGAGGTATGGGTTATTTCCAGATTGGAAACAAGAGGTCCTACTAAAACGGAAGTGGTGTTTATTTCTGTGCTGAAGTTGTTCCATTGTTCAGGAACAGGGATTGAAATGTATGGCCTGAAGTGCAGGGGGAGGCACATCCAACAGTTAGTAGGGTTTTGGGCTGAGACCTCATGGAGCCGAGTGAGGGTGGTATTAAATAGGCTCACCAGGCGAGTATGGGTACGGAGGGTTTCATGTAGTTTTGAGAGAACTAGTCCTTTGTAGGGGCTAGGGGTGCTATGTCCCCGGGTCAATTGGGAGATTGCTTCCTTTACTTGTTTTTCTCTTGCCTGACCTTGAATTCCACCCCCATCAGACATACTGGTATGGGTGAAGTAAGTCCAACAGACAGTGGCTCCAAGTCCTCCAGGACAACTAGGATTAATCATTTTCCCTGTCCAATAATGAGTATTTGCATGCATGCAAAGAGTGGCAGAGTTATAGCAGTTGCGGGGCATATGGGTGTGGGCAGTGAA

>GL-37.K51_D05

GAGGTACCACAGACAAAAAATATTCCTGAGGGTAGGCAGACTATTCGTGTGGGAGGAGTTACCCACCTGATGCATTGGGAGTTGGTTGTGTCTACAGTATTGCTAAATTTTACGCAGGTGAGGTTTGAGGTATGGGTTATTTCCAGATTGGAAACAAGAGGTCCTACTAAAACGGAAGTGGTGTTTATTTCTGTGCTGTAGTTGTTCCATTGTTCAGGTACAGGGATTGAAATGCATGGCCTGAAATACAGGGGGAGGCACATCCAACAGTTAGTAGGGTTTTGGGCCGAGACCTCATGGAGCCCAGTGAGGGTGGTATTAAATAGGCTTACCAGGCAAGTATGGGTATGGAGGGTTTCATGTAGTTTTAAGAGATCTAGTCCTTTGTAGGGGCTAGGGGTGCTATGTACCCGGGTCAGTTGGGAGGTTACTTCCTTTACATGTTTTTCTCTTGCCTGATCTTGAACTCCACCCCCCTCAGACATACCAGTATGGGTGAAGTAAGTCCGACAGACAGTGGCTCCAAGTCTTCCAGGACAACTAGGATTAATCATTTTCCCTGTCCAATAATGAGTATTTGCATGCATGCAAAGAGTGGCAGAGTTATAGCAGTTGTGGGGCATATGGGTGTGGGCAGTGAA

>GL-37-K52_E05

GAGGTACCACAGACAAAAAATATTCCTGAGGGTAGGCAGACTATTCGTGTGGGAGGAGTTACCCACCTGATGCATTGGGAGTTGGTTGTGTCTACAGTATTGCTAAATTTTACACAGGTGAGGTTTGAGGTATGGGTTATTTCCAGATTGGAAACAAGAGGTCCTACTAAAACGGAAGTGGTGTTTATTTCTGTGCTGTAGTTGTTCCATTGTTCAGGTACAGGGATTGAAATGCATGGCCTGAAATACAGGGGGAGGCACATCCAACAGTTAGTAGGGTTTTGGGCCGAGACCTCATGGAGCCCAGTGAGGGTGGTATTAAATAGGCTTACCAGGCAAGTATGGGTATGGAGGGTTTCATGTAGTTTTAAGAGATCTAGTCCTTTGTAGGGGCTAGGGGTGCTATGTACCCGGGTCAGTTGGGAGGTTACTTCCTTTACATGTTTTTCTCTTGCCTGATCTTGAACTCCACCCCCCTCAGACATACCAGTATGGGTGAAGTAAGTCCGACAGACAGTGGCTCCAAGTCTTCCAGGACAACTAGGATTAATCATTTTCCCTGTCCAATAATGAGTATTTGCATGCATGCAAAGAGTGGCAGAGTTATAGCAGTTGTGGGGCATATGGGTGTGGGCAGTGAA

>GL-37-K53_F05

GAGGTACCACAGACAAAAAATATTCCTGAGGGTAGGCAGACTATTCGTGGGGGGGGAGTTACCCACCTGATGCATTGGGAGTTGGTTGTGTCTATCGTATTGCTAAATTTTACACAGGTGAGGTTTGAGGTATGGGTTATTTCCAGATTGGAAACAAGAGGTCCTACTAAAACGGAAGTGGTGTTTATGTGTTGAAGTTGTTCCCTTGTTCAGGTACAGGGATTGAAATGTATGGCCTTAAGTGCAGAGGGAGGCACATCCAACAGTTAGTAGGGTTTTGGGCCGAGACCTCATGAAACCCAGTGAGGGTGGTATTAAATAGGCTTACCAGGCGAGTATGGGTACGGAGGGTTTCATGTAGTTTTAAGAGATTTAGTCCTTTGTAGGGGCTAGGGGTGCTATGTACCCGGGTCAGTTGGGAAGGTACTCCTTTACATGTTTTTCTCTTGCTGATCTGAACTTCACCCCCCTC

>GL-37-K54_G05

TTCACTGCCCACACCCATATGCCCCGCAACTGCTATAACTCTGCCACTCTTTGCATGCATGCAAATACTCATTATTGGACAGGGAAAATGATTAATCCTAGTTGTCCTGGAGGACTTGGAGCCACTGTCTGTTGGACTTACTTCACCCATACCAGTATGTCTGATGGGGGTGGAATTCAAGGTCAGGCAAGAGAAAAACATGTAAAGGAAGTAACCTCCCAACTGACCCGGGTACATAGCACCCCTAGCCCCTACAAAGGACTAGATCTCTTAAAACTACATGAAACCCTCCATACCCATACTTGCCTGGTAAGCCTATTTAATACCACCCTCACTGGGCTCCATGAGGTCTCGGCCCAAAACCCTACTAACTGTTGGATGTGCCTCCCCCTGTATTTCAGGCCATGCATTTCAATCCCTGTACCTGAACAATGGAACAACTACAGCACAGAAATAAACACCACTTCCGTTTTAGTAGGACCTCTTGTTTCCAATCTGGAAATAACCCATACCTCAAACCTCACCTGTGTAAAATTTAGCAATACTGTAGACACGACCAACTCCCAATGCATCAGGTGGGTAACTCCTCCCACACGAATAGTCTGCCTACCCTCAGGAATATTTTTTGTCTGTGGTACCTC

>GL-37-K55_H05

GAGGTACCACAGACAAAAAATATTCCTGAGGGTAGGCAGACTATTCGTGTGGGAGGAGTTACCCACCTGATGCATTGGGAGTTGGTTGTGTCTACAGTATTGCTAAATTTTACACGGGTGAGGTTTGAGGTATGGGTTATTTCCAGATTGGAAACAAGAGGTCCTACTAAAACGGAAGTGGTGTTTATTTCTGTGCTGTAGTTGTTCCATTGTTCAGGTACAGGGATTGAAATGCATGGCCTGAAATACAGGGGGAGGCACATCCAACAGTTAGTAGGGTTTTGGGCCGAGACCTCATGGAGCCCAGTGAGGGTGGTATTAAATAGGCTTACCAGGCAAGTATGGGTATGGAGGGTTTCATGTAGTTTTAAGAGATCTAGTCCTTTGTAGGGGCTAGGGGTGCTATGTCCCCGGGTCAGTTGGGAGGTTACTTCCTTTACATGTTTTTCTCTTGCCTGATCTTGAACTCCACCCCCCTCAGACATACCAGTATGGGGTGAAGTAAGTCCGAC

>GL-37-K56_A06

GAGGTACCACAGACAAAAAATATTCCTGAGGGTAGGCAGACTATTCGTGGGGGGGAGTTACCCACCTGATGCATTGGGAGTTGGTTGTGTCTATCGTATTGCTAAATTTTACACAGGTGAGGTTTGAGGTATGGGTTATTTCCAGATTGGAAACAAGAGGTCCTACTAAAACGGAAGTGGTGTTTATGTGTTGAAGTTGTTCCCTTGTTCAGGTACAGGGATTGAAATGTATGGCCTTAAGTGCAGAGGGAGGCACATCCAACAGTTAGTAGGGTTTTGGGCCGAGACCTCATGAAACCCAGTGAGGGTGGTATTAAATAGGCTTACCAGGCGAGTATGGGTACGGAGGGTTTCATGTAGTTTTGAGAGATCTAGTCCTTTGTAGGGGCTAGGCGTGCTATGTACTCAGGTCAGTTGGGAGATTACTTCCTTTACGTGTTTTTCTCTTGCCTGATCTTGAACTCCACCCCTATCAGACATACCAGTATGGGTGAACTAAGTCCAACAGACAGTGGCTCCAAGTCCTCCAGGACAACTAGGATTAATCATTTTCCCTGTCCAATAATGAGTATTTGCATGCATGCAAAGAGTGGCAGAGTTATAGCAGTTGTGGGGCATATGGGTGTGGGCAGTGAA

>GL-37-K57_B06

GAGGTACCACAGACAAAAAATATTCCTGAGGGTAGGCAGACTATTCGTGGGGGGGAGTTACCCACCTGATGCATTGGGAGTTGGTTGTGTCTATCGTATTGCTAAATTTACACAGGTGAGGTTTGAGGTATGGGTTATTTCCAGATTGGAAACAAGAGGTCCTACTAAAACGGAAGTGGTGTTTATGTGTTGAAGTTGTTCCCTTGTTCAGGTACAGGGATTGAAATGTATGGCCTTAAGTGCAGAGGGAGGCACATCCAACAGTTAGTAGGGTTTTGGGCCGAGACCTCATGAAACCCAGTGAGGGTGGTATTAAATAGGCTTACCAGGCGAGTATGGGTACGGAGGGTTTCATGTAGTTTTGAGAGATCTAGTCCTTTGTAGGGGCTAGGCGTGCTATGTACTCAGGTCAGTTGGGAGATTACTTCCTTTACGTGTTTTTCTCTTGCCTGATCTTGAACTCCACCCCTATCAGACATACCAGTATGGGTGAACTAAGTCCAACAGACAGTGGCTCCAAGTCCTCCAGGACAACTAGGATTAATCATTTTCCCTGTCCAATAATGAGTATTTGCATGCATGCAAAGAGTGGCAGAGTTATAGCAGTTGTGGGGCATATGGGTGTGGGCAGTGAA

>GL-37-K58_C06

TTCACTGCCCACACCCATATGCCCCACAACTGCTATAACCCTGCCACTCTTTGCATGCATGCAAATACTCATTATTGGACAGGGAAAATGATTAATCCTAGTTGTCCTGGAAGACTTGGAGCCACTGTCTGTCGGACTTACTTCACCCATACTGGTATGTCTGAGGGGGGTGGAGTTCAAGATCAGGCAAGAGAAAAACATGTAAAGGAAGTAACCTCCCAACTGACCCGGGTACATAGCACCCCTAGCCCCTACAAAGGACTAGATCTCTTAAAACTACATGAAACCCTCCATACCCATACTTGCCTGGTAAGCCTATTTAATACCACCCTCGCTGGGCTCCATGAGGTCTCGGCCCAAAACCCTACTAACTGTTGGATGTGCCTCCCCCTGTATTTCAGGCCATGCACTTCAATCCCTGTACCTGAACAATGGAACAACTACAGCACAGAAATAAACACCACTTCCGTTTTAGTAGGACCTCTTGTTTCCAATCTGGAAATAACCCATACCTCAAACCTCACCTGTGTAAAATTTAGCAATACTGTAGACACAACCAACTCCCAATGCATCAGGTGGGTAACTCCTCCCACACGAATAGTCTGCCTACCCTCAGGAATATTTTTTGTCTGTGGTACCTC

>GL-37-K60_D06

GAGGTACCACAGACAAAAAATATTCCTGAGGGTAGGCAGACTATTCGTGTGGAAGGAGTTACCCACCTGATGCATTGGGAGTTGGTTGTGTCTACAGTATTGCTAAATTTTACACAGGTGAGGTTTGAGGTATGGGTTATTTCCAGATTGGAAACAAGAGGTCCTACTAAAACGGAAGTGGTGTTTATTTCTGTGCTGTAGTTGTTCCATTGTTCAGGTACAGGGATTGAAATGCATGGCCTGAAATACAGGGGGAGGCACATCCAACAGTTAGTAGGGTTTTGGGCCGAGACCTCATGGAGCCCAGTGAGGGTGGTATTAAATAGGCTTACCAGGCAAGTATGGGTATGGAGGGTTTCATGTAGTTTTAAGAGATCTAGTCCTTTGTAGGGGCTAGGGGTGCTATGTACCCGGGTCAGTTGGGAGGTTACTTCCTTTACATGTTTTTCTCTTGCCTGATCTTGAACTCCACCCCCCTCAGACATACCAGTATGGGTGAAGTAAGTCCGACAGACAGTGGCTCCAAGTCTTCCAGGACAACTAGGATTAATCATTTTCCCTGTCCAATAATGAGTATTTGCATGCATGCAAAGAGTGGCAGAGTTATAGCAGTTGTGGGGCATATGGGTGTGGGCAGTGAA

>GL-37-K63_E06

GAGGTACCACAGACAAAAAATATTCCTGAGGGTAGGCAGACTATTCGTGTGGGAGGAGTTACCCACCTGATGCATTGGGAGTTGGTTGTGTCTACAGTATTGCTAAATTTTACACAGGTGAGGTTTGAGGTATGGGTTATTTCCAGATTGGAAACAAGGGGTCCTACTAAAACGGAAGTGGTGTTTATTTCTGTGCTGTAGTTGTTCCATTGTTCAGGTACAGGGATTGAAATGCATGGCCTGAAATACAGGGGGAGGCACATCCAACAGTTAGTAGGGTTTTGGGCCGAGACCTCATGGAGCCCAGTGAGGGTGGTATTAAATAGGCTTACCAGGCAAGTATGGGTATGGAGGGCTTCATGTAGTTTTAAGAGATCTAGTCCTTTGTAGGGGCTAGGGGTGCTATGTACCCGGGTCAGTTGGGAGGTTACTTCCTTTACATGTTTTTCTCTTGCCTGATCTTGAACTCCACCCCCCTCAGACATACCAGTATGGGTGAAGTAAGTCCGACAGACAGTGGCTCCAAGTCTTCCAGGACAACTAGGATTAATCATTTTCCCTGTCCAATAATGAGTATTTGCATGCATGCAAAGAGTGGCAGAGTTATAGCAGTTGTGGGGCATATGGGTGTGGGCAGTGAA

**KO-I**

>GL-9-KH1_B02

GAGGTACCACAGACAAAAAATATTCCTGAGGGTAGGCAGACTATTCGTGGGGGGGAGTTACCCACCTGATGCATTGGGAGTTGGTTGTGTCTATCGTATTGCTAAATTTTACACAGGTGAGGTTTGAGGTATGGGTTATTTCCAGATTGGAAACAAGAGGTCCTACTAAAACGGAAGTGGTGTTTATGTGTTGAAGTTGTTCCCTTGTTCAGGTACAGGGATTGAAATGTATGGCCTTAAGTGCAGAGGGAGGCACGTCCAACAGTTAGTAGGGTTTTGGGCCGAGACCTCATGAAACCCAGTGAGGGTGGTATTAAATAGGCTTACCAGGCGAGTATGGGTACGGAGGGTTTCATGTAGTTTTGAGAGATCTAGTCCTTTGTAGGGGCTAGGCGTGCTATGTACTCAGGTCAGTTGGGAGATTACTTCCTTTACGTGTTTTTCTCTTGCCTGATCTTGAACTCCACCCCTATCAGACATACCAGTATGGGTGAACTAAGTCCAACAGACAGTGGCTCCAAGTCCTCCAGGACAACTAGGATTAATCATTTTCCCTGTCCAATAATGAGTATTTGCATGCATGCAAAGAGTGGCAGAGTTATAGCAGTTGTGGGGCATATGGGTGTGGGCAGTGAA

>GL-9-KH2_C02

TTCACTGCCCACACCCATATGCCCCACAACTGCTATAACTCTGCCACTCTTTGCATGCATGCAAATACTCATTATTGGACAGGGAAAATGATTAATCCTAGTTGTCCTGGAGGACTTGGAGCCACTGTCTGTTGGACTTAGTTCACCCATACTGGTATGTCTGATAGGGGTGGAGTTCAAGATCAGGCAAGAGAAAAACACGTAAAGGAAGTAATCTCCCAACTGACCTGAGTACATAGCACGCCTAGCCCCTACAAAGGACTAGATCTCTCAAAACTACATGAAACCCTCCGTACCCATACTCGCCTGGTAAGCCTATTTAATACCACCCTCACTGGGTTTCATGAGGTCTCGGCCCAAAACCCTACTAACTGTTGGATGTGCCTCCCTCTGCACTTAAGGCCATACATTTCAATCCCTGTACCTGAACAAGGGAACAACTTCAACACATAAACACCACTTCCGTTTTAGTAGGACCTCTTGTTTCCAATCTGGAAATAACCCATACCTCAAACCTCACCTGTGTAAAATTTAGCAATACGATAGACACAACCAACTCCCAATGCATCAGGTGGGTAACTCCCCCCCACGAATAGTCTGCCTACCCTCAGGAATATTTTTTGTCTGTGGTACCTC

>GL-9-KH3_D02

GAGGTACCACAGACAAAAAATATTCCTGAGGGTAGGCAGACTATTCGTGGGGGGGAGTTACCCACCTGATGCATTGGGAGTTGGTTGTGTCTATCGTATTGCTAAATTTTACACAGGTGAGGTTTGAGGTATGGGTTATTTCCAGATTGGAAACAAGAGGTCCTACTAAAACGGAAGTGGTGTTCATGTGTTGAAGTTGTTCCCTTGTTCAGGTACAGGGATTGAAATGTATGGCCTGAAGTGCAGGGGGAGGCACATCCAACAGTTAGTAGGGTTTTGGGCTGAGACCTCATGGAGCCGAGTGAGGGTGGTATTAAATAGGCTCACCAGGCGAGTATGGGTACGGAGGGTTTCATGTAGTTTTGAGAGAACTAGTCCTTTGTAGGGGCTAGGGGTGCTATGTCCCCGGGTCAGTTGGGAGATTGCTTCCTTTACTTGTTTTTCTCTTGCCTGACCTTGAATTCCACCCCCATCAGACATACTGGTATGGGTGAAGTAAGTCCAACAGACAGTGGCTCCAAGTCCTCCAGGACAACTAGGATTAATCATTTTCCCTGTCCAATAATGAGTATTTGCATGCATGCAAAGAGTGGCAGAGTTATAGCAGTTGCGGGGCATATGGGTGTGGGCAGTGAA

>GL-9-KH4_E02

GAGGTACCACAGACAAAAAATATTCCTGAGGGTAGGCAGACTATTCGTGTGGGAGGAGTTACCCACCTGATGCATTGGGAGTTGGCTGTGTCTATAGTATTGCTAAATTTTACACAGGTGAGGTTTGAGGTATGGGTTATTTCCAGATTGGAAACAAGAGGTCCTACTAAAACGGAAGTGGTGTTTATGTGTTGAAGTTGTTCCCTTGTTCAGGTACAGGGATTGAAATGTATGGCCTTAAGTGCAGAGGGAGGCACATCCAACAGTTAGTAGGGTTTTGGGCCGAGACCTCATGAAACCCAGTGAGGGTGGTATTAAATAGGCTTACCAGGCGAGTATGGGTACGGAGGGTTTCATGTAGTTTTGAGAGATCTAGTCCTTTGTAGGGGCTAGGCGTGCTATGTACTCAGGTCAGTTGGGAGATTACTTCCTTTACGTGTTTTTCTCTTGCCTGATCTTGAACTCCACCCCTATCAGACATACCAGTATGGGTGAACTAAGTCCAACAGACAGTGGCTCCAAGTCCTCCAGGACAACTAGGATTAATCATTTTCCCTGTCCAATAATGAGTATTTGCATGCATGCAAAGAGTGGCAGAGTTATAGCAGTTGTGGGGCATATGGGTGTGGGCAGTGAA

>GL-9-KH5_F02

TTCACTGCCCACACCCATATGCCCCACAACTGCTATAACTCTGCCACTCTTTGCATGCATGCAAATACTCATTATTGGACAGGGAAAATGATTAATCCTAGTTGTCCTGGAGGACTTGGAGCCACTGTCTGTTGGACTTAGTTCACCCATACTGGTATGTCTGGTAGGGGTGGAGTTCAAGATCAGGCAAGAGAAAAACACGTAAAGGAAGTAATCTCCCAACTGACCTGAGTACATAGCACGCCTAGCCCCTACAAAGGACTAGATCTCTCAAAACTACATGAAACCCTCCGTACCCATACTCGCCTGGTAAGCCTATTTAATACCACCCTCACTGGGTTTCATGAGGTCTCGGCCCAAAACCCTACTAACTGTTGGATGTGCCTCCCTCTGCACTTAAGGCCATACATTTCAATCCCTGTACCTGAACAAGGGAACAACTTCAACACATAAACACCACTTCCGTTTTAGTAGGACCTCTTGTTTCCAATCTGGAAATAACCCATACCTCAAACCTCACCTGTGTAAAATTTAGCAATACGATAGACACAACCAACTCCCAATGCATCAGGTGGGTAACTCCCCCCCACGAATAGTCTGCCTACCCTCAGGAATATTTTTTGTCTGTGGTACCTC

>GL-9-KH6_G02

GAGGTACCACAGACAAAAAATATTCCTGAGGGTAGGCAGACTATTCGTGGGGGGGAGTTACCCACCTGATGCATTGGGAGTTGGTTGTGTCTATCGTATTGCTAAATTTTACACAGGTGAGGTTTGAGGTATGGGTTATTTCCAGATTGGAAACAAGAGGTCCTACTAAAACGGAAGTGGTGTTTATGTGTTGAAGTTGTTCCCTTGTTCAGGTACAGGGATTGAAATGTATGGCCTTAAGTGCAGAGGGAGGCACATCCAACAGTTAGTAGGGTTTTGGGCCGAGACCTCATGAAACCCAGTGAGGGTGGTATTAAATAGGCTTACCAGGCGAGTATGGGTACGGAGGGTTTCATGTAGTTTTGAGAGATCTAGTCCTTTGTAGGGGCTAGGCGTGCTATGTACTCAGGTCAGTTGGGAGATTACTTCCTTTACGTGTTTTCCTCTTGCCTGATCTTGAACTCCACCCCCATCAGACATACCAGTATGGGTGAACTAAGTCCAACAGACAGTGGCTCCAAGTCCTCCAGGACAACTAGGATTAATCATTTTCCCTGTCCAATAATGAGTATTTGCATGCATGCAAAGAGTGGCAGAGTTATAGCAGTTGTGGGGCATATGGGTGTGGGCAGTGAA

>GL-9-KH7_H02

GAGGTACCACAGACAAAAAATATTCCTGAGGGTAGGCAGACTATTCGTGTGGGAGGTGTTACCCACCTGATGCATTGGGAGCTGGTTGTGTCTATAGTATTGCTAAATTTTACACAGGTGAGGTTTGAGGTATGGGTTATTTCCAGATTGGAAACAAGAGGTCCTACTAAAACGGAAGTGGTGTTTATTTCTGTGCTGAAGTTGTTCCATTGTTCAGGAACAGGGATTGAAATGTATGGCCTGAAGTGCAGGGGGAGGCATATCCAACAGTTAGTAGGGTTTTGGGCCGAGACCTCATGGAGCCCAGTGAGGGTGGTATTAAATAGGCTTACCAGGCAAGTATGGGTATGGAGGGTTTCATGTAGTTTTAAGAGATCTAGTCCTTTGTAGGGGCTAGGGGTGCTATGTACCCGGGTCAGTTGGGAGGTTACTTCCTTTACATGTTTTTCTCTTGCCTGATCTTGAACTCCACCCCTATCAGACATACCAGTATGGGTGAACTAAGTCCAACAGACAGTGGCTCCAAGTCCTCCAGGACAACTAGGATTAATCATTTTCCCTGTCCAATAATGAGTATTTGCATGCATGCAAAGAGTGGCAGAGTTATAGCAGTTGTGGGGCATATGGGTGTGGGCAGTGAA

>GL-9-KH8_A03

TTCACTGCCCACACCCATATGCCCCACAACTGCTATAACTCTGCCACTCTTTGTATGCATGCAAATATTCATTATTGGACAGGAAAAACGATTAATCCCAGTTGTCCTGGAGGACTTGGAGGACTCACTTCACTCATACCAGTATGTCTGATGGGGGTGGAGTTCAAGATCAGGCAACAGAAAAACACATAAAGGAAGTAATCTCCCAACTGACCTGGGTACATAGCACCCCTGGCCCCTACAAAGGACTAGATCTCTCAAAACTACATGAAACCCTCCATACCCATACTGGCCTGGTAAGCCTATTTAATACCACCCTGACTGGGCTCCATGAGGTCTCGGCCCAAAACCCTACTAACTGTTGGATGTGCCTCCCCCTGCACTTTAGGCCATACATTTCAATCCCTATACCTGAACAATGGAACAACTTCAGCACAGAAATAAACACCACTTCTGTTTTAGTAGGTCCTCTTTCCAATCTGGAAATAACCCATACCTCAAACCTCACCTGTGTAAAATTTAGCAATACTATAGACACAGCCAACTCCCAATGCATCAGGTGGGTAACTCCTCCCACACGAATAGTCTGCCTACCCTCAGGAATATTTTTTGTCTGTGGTACCTC

>GL-9-KH9_B03

GAGGTACCACAGACAAAAAATATTCCTGAGGGTAGGCAGACTATTCGTGGGGGGGAGTTACCCACCTGATGCATTGGGAGTTGGTTGTGTCTATCGTATTGCTAAATTTTACACAGGTGAGGTTTGAGGTATGGGTTATTTCCAGATTGGAAACAAGAGGTCCTACTAAAACGGAAGTGGTGTTTATGTGTTGAAGTTGTTCCCTTGTTCAGGTACAGGGATTGAAATGTATGGCCTTAAGTGCAGAGGGAGGCACATCCAACAGTTAGTAGGGTTTTGGGCCGAGACCTCATGAAACCCAGTGAGGGTGGTATTAAATAGGCATACCAGGCGAGTATGGGTACGGAGGGTTTCATGTAGTTTTGAGAGATCTAGTCCTTTGTAGGGGCTAGGCGTGCTATGTACTCAGGTCAGTTGGGAGATTACTTCCTTTACGTGTTTTTCTCTTGCCTGATCTTGAACTCCACCCCCCTCAGACATACCAGTATGGGTGAAGTAAGTCCGACAGACAGTGGCTCCAAGTCTTCCAGGACAACTAGGATTAATCATTTTCCCTGTCCAATAATGAGTATTTGCATGCATGCAAAGAGTGGCAGAGTTATAGCAGTTGTGGGGCATATGGGTGTGGGCAGTGAA

>GL-9-KH10_C03

GAGGTACCACAGACAAAAAATATTCCTGAGGGTAGGCAGACTATTCGTGGGGGGGAGTTACCCACCTGATGCATTGGGAGTTGGTTGTGTCTATCGTATTGCTAAATTTTACACAGGTGAGGTTTGAGGTATGGGTTATTTCCAGATTGGAAACAAGAGGTCCTACTAAAACGGAAGTGGTGTTTATTTCTGTGCTGTAGTTGTTCCATTGTTCAGGTACAGGGATTGAAATGCATGGCCAGAAATACAGGGGGAGGCACATCCAACAGTTAGTAGGGTTTTGGGCCGAGACCTCATGAAACCCAGTGAGGGTGGTATTAAATAGGCTTACCAGGCGAGTATGGGTACGGAGGGTTTCATGTAGTTTTGAGAGATCTAGTCCTTTGTAGGGGCTAGGCGTGCTATGTACTCAGGTCAGTTGGGAGATTACTTCCTTTACGTGTTTTTCTCTTGCCTGATCTTGAACTCCACCCCTATCAGACATACCAGTATGGGTGAACTAAGTCCAACAGACAGTGGCTCCAAGTCCTCCAGGACAACTAGGATTAATCATTTTCCCTGTCCAATAATGAGTATTTGCATGCATGCAAAGAGTGGCAGAGTTATAGCAGTTGTGGGGCATATGGGTGTGGGCAGTGAA

>GL-9-KH11_D03

GAGGTACCACAGACAAAAAATATTCCTGAGGGTAGGCAGACTATTCGTGGGGGGGAGTTACCCACCTGATGCATTGGGAGTTGGTTGTGTCTATCGTATTGCTAAATTTTACACAGGTGAGGTTTGAGGTATGGGTTATTTCCAGATTGGAAACAAGAGGTCCTACTAAAACGGAAGTGGTGTTTATGTGTTGAAGTTGTTCCCTTGTTCAGGTACAGGGATTGAAATGTATGGCCTTAAGTGCAGAGGGAGGCACATCCAACAGTTAGTAGGGTTTTGGGCCGAGACCTCATGAAACCCAGTGAGGGTGGTATTAAATAGGCTTACCAGGCGAGTATGGGTACGGAGGGTTTCATGTAGTTTTGAGAGATCTAGTCCTTTGTAGGGGCTAGGCGTGCTATGTACTCAGGTCAGTTGGGAGATTACTTCCTTTACGTGTTTTTCTCTTGCCTGATCTTGAACTCCACCCCTATCAGACATACCAGTATGGGTGAACTAAGTCCAACAGACAGTGGCTCCAAGTCCTCCAGGACAACTAGGATTAATCATTTTCCCTGTCCAATAATGAGTATTTGCATGCATGCAAAGAGTGGCAGAGTTATAGCAGTTGTGGGGCATATGGGTGTGGGCAGTGAA

>GL-9-N2_F03

TTCACTGCCCACACCCATATGCCCCACAACTGCTATAACTCTGCCACTCTTTGCATGCATGCAAATACTCATTATTGGACAGGGAAAATGATTAATCCTAGTTGTCCTGGAGGACTTGGAGCCACTGTCTGTTGGACTTAGTTCACCCATACTGGTATGTCTGATAGGGGTGGAGTTCAAGATCAGGCAAGAGAAAAACACGTAAAGGAAGTAATCTCCCAACTGACCTGAGTACATAGCACGCCTAGCCCCTACAAAGGACTAGATCTCTCAAAACTACATGAAACCCTCCGTACCCATACTCGCCTGGTAAGCCTATTTAATACCACCCTCACTGGGTTTTCATGAGGTCTCGGCCCAAACCCTACTAACTGTTGGATGTGCCTCCCTCTGCACTTAAGCCATACATTTCATCCCTGTACCTG

>GL-9-KN4_G03

GAGGTACCACAGACAAAAAATATTCCTGAGGGTAGGCAGACTATTCGTGGGGGGGAGTTACCCACCTGATGCATTGGGAGTTGGTTGTGTCTATCGTATTGCTAAATTTTACACAGGTGAGGTTTGAGGTATGGGTTATTTCCAGATTGGAAACAAGAGGTCCTACTAAAACGGAAGTGGTGTTTATGTGTTGAAGTTGTTCCCTTGTTCAGGTACAGGGATTGAAATGTATGGCCTTAAGTGCAGAGGGAGGCACATCCAACAGTTAGTAGGGTTTTGGGCCGAGACCTCATGAAACCCAGTGAGGGTGGTATTAAATAGGCTTACCAGGCGAGTATGGGTACGGAGGGTTTCATGTAGTTTTGAGAGATCTAGTCCTTTGTAGGGGCTAGGCGTGCTATGTACTCAGGTCAGTTGGGAGATTACTTCCTTTACGTGTTTTTCTCTTGCCTGATCTTGAACTCCACCCCTATCAGACATACCAGTATGGGTGAACTAAGTCCAACAGACAGTGGCTCCAAGTCCTCCAGGACAACTAGGATTAATCATTTTCCCTGTCCAATAATGAGTATTTGCATGCATGCAAAGAGTGGCAGAGTTATAGCAGTTGTGGGGCATATGGGTGTGGGCAGTGAA

>GL-9-KN7_H03

GAGGTACCACAGACAAAAAATATTCCTGAGGGTAGGCAGACTATTCGTGTGGGAGGTGTTACCCACCTGATGCATTGGGAGCTGGTTGTGTCTATAGTATTGCTAAATTTTACACAGGTGAGGTTTGAGGTATGGGTTATTTCCAGATTGGAAACAAGAGGTCCTACTAAAACGGAAGTGGTGTTTATTTCTGTGCTGAAGTTGTTCCATTGTTCAGGAACAGGGATTGAAATGTATGGCCTTAAGTGCAGAGGGAGGCACATCCAACAGTTAGTAGGGTTTTGGGCCGAGACCTCATGAAACCCAGTGAGGGTGGTATTAAATAGGCTTACCAGGCGAGTATGGGTACGGAGGGTTTCATGTAGTTTTGAGAGATCTAGTCCTTTGTAGGGGCTAGGCGTGCTATGTACTCAGGTCAGTTGGGAGATTACTTCCTTTACGTGTTTTTCTCTTGCCTGATCTTGAACTCCACCCCTATCAGACATACCAGTATGGGCGAACTAAGTCCAACAGACAGTGGCTCCAAGTCCTCCAGGACAACTAGGATTAATCATTTTCCCTGTCCAATAATGAGTATTTGCATGCATGCAAAGAGTGGCAGAGTTATAGCAGTTGTGGGGCATATGGGTGTGGGCAGTGAA

>GL-9-KN8_A04

GAGGTACCACAGACAAAAAATATTCCTGAGGGTAGGCAGACTATTCGTGGGGGGGAGTTACCCACCTGATGCATTGGGAGTTGGTTGTGTCTATCGTATTGCTAAATTTTACACAGGTGAGGTTTGAGGTATGGGTTATTTCCAGATTGGAAACAAGAGGTCCTACTAAAACGGAAGTGGTGTTTATGTGTTGAAGTTGTTCCCTTGTTCAGGTACAGGGATTGAAATGTATGGCCTTAAGTGCAGAGGGAGGCACATCCAACAGTTAGTAGGGTTTTGGGCCGAGACCTCATGAAACCCAGTGAGGGTGGTATTAAATAGGCTTACCAGGCGAGTATGGGTACGGAGGGTTTCATGTAGTTTTGAGAGATCTAGTCCTTTGTAGGGGCTAGGCGTGCTATGTACTCAGGTCAGTTGGGAGATTACTTCCTTTACGTGTTTTTCTCTTGCCTGATCTTGAACTCCACCCCTATCAGACATACCAGTATGGGTGAACTAAGTCCAACAGACAGTGGCTCCAAGTCCTCCAGGACAACTAGGATTAATCATTTTCCCTGTCCAATAATGAGTATTTGCATGCATGCAAAGAGTGGCAGAGTTATAGCAGTTGTGGGGCATATGGGTGTGGGCAGTGAA

>GL-9-KN9_B04

GAGGTACCACAGACAAAAAATATTCCTGAGGGTAGGCAGACTATTCGTGGGGGGGAGTTACCCACCTGATGCATTGGGAGTTGGTTGTGTCTATCGTATTGCTAAATTTTACACAGGTGAGGTTTGAGGTATGGGTTATTTCCAGATTGGAAACAAGAGGTCCTACTAAAACGGAAGTGGTGTTTATGTGTTGAAGTTGTTCCCTTGTTCAGGTACAGGGATTGAAATGTATGGCCTTAAGTGCAGAGGGAGGCACATCCAACAGTTAGTAGGGTTTTGGGCCGAGACCTCATGAAACCCAGTGAGGGTGGTATTAAATAGGCTTACCAGGCGAGTATGGGTACGGAGGGTTTCATGTAGTTTTGAGAGATCTAGTCCTTTGTAGGGGCTAGGCGTGCTATGTACTCAGGTCAGTTGGGAGATTACTTCCTTTACGTGTTTTTCTCTTGCCTGATCTTGAACTCCACCCCTATCAGACATACCAGTATGGGTGAACTAAGTCCAACAGACAGTGGCTCCAAGTCCTCCAGGACAACTAGGATTAATCATTTTCCCTGTCCAATAATGAGTATTTGCATGCATGCAAAGAGTGGCAGAGTTATAGCAGTTGTGGGGCATATGGGTGTGGGCAGTGAA

>GL-9-KN10_C04

GAGGTACCACAGACAAAAAATATTCCTGAGGGTAGGCAGACTATTCGTGGGGGGGAGTTACCCACCTGATGCATTGGGAGTTGGTTGTGTCTATCGTATTGCTAAATTTTACACAGGTGAGGTTTGAGGTATGGGTTATTTCCAGATTGGAAACAAGAGGTCCTACTAAAACGGAAGTGGTGTTTATGTGTTGAAGTTGTTCCCTTGTTCAGGTACAGGGATTGAAATGTATGGCCTTAAGTGCAGAGGGAGGCACATCCAACAGTTAGTAGGGTTTTGGGCCGAGACCTCATGAAACCCAGTGAGGGTGGTATTAAATAGGCTTACCAGGCGAGTATGGGTACGGAGGGTTTCATGTAGTTTTGAGAGATCTAGTCCTTTGTAGGGGCTAGGCGTGCTATGTACTCAGGTCAGTTGGGAGATTACTTCCTTTACGTGTTTTTCTCTTGCCTGATCTTGAACTCCACCCCTATCAGACATACCAGTATGGGTGAACTAAGTCCAACAGACAGTGGCTCCAAGTCCTCCAGGACAACTAGGATTAATCATTTTCCCTGTCCAATAATGAGTATTTGCATGCATGCAGAGAGTGGCAGAGTTATAGCAGTTGTGGGGCATATGGGTGTGGGCAGTGAA

>GL-9-K14_D04

TTCACTGCCCACACCCATATGCCCCACAACTGCTATAACTCTGCCACTCTTTGCATGCATGCAAATACTCATTATTGGACAGGGAAAATGATTAATCCTAGTTGTCCTGGAAGACTTGGAGCCACTGTCTGTCGGACTTACTTCACCCATACTGGTATGTTTGAGGGGGGTGGAGTTCAAGATCAGGCAAGAGAAAAACATGTAAAGGAAGTAATCTCCCAACTGACCTGAGTACATAGCACGCCTAGCCCCTACAAAGGACTAGATCTCTCAAAACTACATGAAACCCTCCGTACCCATACTCGCCTGGTAAGCCTATTTAATACCACCCTCACTGGGTTTCATGAGGTCTCGGCCCAAAACCCTACTAACTGTTGGATGTGCCTCCCTCTGCACTTAAGGCCATACATTTCAATCCCTGTACCTGAACAAGGGAACAACTTCAACACATAAACACCACTTCCGTTTTAGTAGGACCTCTTGTTTCCAATCTGGAAATAACCCATACCTCAAACCTCACCTGTGTAAAATTTAGCAATACGATAGACACAACCAACTCCCAATGCATCAGGTGGGTAACTCCCCCCCACGAATAGTCTGCCTACCCTCAGGAATATTTTTTGTCTGTGGTACCTC

>GL-9-K16_E04

TTCACTGCCCACACCCATATGCCCCGCAACTGCTATAACTCTGCCATTCTTTGCATGCATGCAAATACTCATTATTGGACAGGGAAAATGATTAATCCCAGTTGTCCTGGAGGACTTGGAGGACTCACTTCACTCATACCAGTATGTCTGATGGGGGTGGAGTTCAAGATCAGGCAACAGAAAAACACATAAAGGAAGTAACCTCCCAACTGACCCGGGGACATAGCACCCCTAGCCCCTACAAAGGACTAGTTCTCTCAAAACTACATGAAACCCTCCATACCCATACTGGCCTGGTAAGCCTATTTAATACCACCCTGACTGGGCTCCATGAGGTCTCGGCCCAAAACCCTACTAACTGTTGGATGTGCCTCCCCCTGCACTTTAGGCCATACATTTCAATCCCTATACCTGAACAATGGAACAACTTCAGCACAGAAATAAACACCACTTCTGTTTTAGTAGGTCCTCTTTCCAATCTGGAAATAACCCATACCTCAAACCTCACCTGTGTAAAATTTAGCAATACTATAGACACAGCCAACTCCCAATGCATCAGGTGGGTAACTCCTCCCACACGAATAGTCTGCCTACCCTCAGGAATATTTTTTGTCTGTGGTACCTC

>GL-9-K17_F04

TTCACTGCCCACACCCATATGCCCCGCAACTGCTATAACTCTGCCACTCTTTGCATGCATGCAAATACTCATTATTGGACAGGGAAAATGATTAATCCTAGTTGTCCTGGAGGACTTGGAGCCACTGTCTGTTGGACTTACTTCACCCATACCAGTATGTCTGATGGGGGTGGAATTCAAGGTCAGGCAAGAGAAAAACAAGTAAAGGAAGCAATCTCCCAACTGACCCGGGGACATAGCACCCCTAGCCCCTACAAAGGACTAGTTCTCTCAAAACTACATGAAACCCTCCGTACCCATACTCGCCTGGTAAGCCTATTTAATACCACCCTCACTGGGTTTCATGAGGTCTCGGCCCAAAACCCTACTAACTGTTGGATGTGCCTCCCTCTGCACTTAAGGCCATACATTTCAATCCCTGTACCTGAACAAGGGAACAACTTCAACACATAAACACCACTTCCGTTTTAGTAGGACCTCTTGTTTCCAATCTGGAAATGACCCATACCTCAAACCTCACCTGTGTAAAATTTAGCAATACGATAGACACAACCAACTCCCAATGCATCAGGTGGGTAACTCCCCCCCACGAATAGTCTGCCTACCCTCAGGAATATTTTTTGTCTGTGGTACCTC

>GL-9-K22_G04

GAGGTACCACAGACAAAAAATATTCCTGAGGGTAGGCAGACTATTCGTGGGGGGGAGTTACCCACCTGATGCATTGGGAGTTGGTTGTGTCTATCGTATTGCTAAATTTTACACAGGTGAGGTTTGAGGTATGGGTTATTTCCAGATTGGAAACAAGAGGTCCTACTAAAACGGAAGTGGTGTTTATTTCTGTGCTGTAGTTGTTCCATTGTTCAGGTACAGGGATTGAAATGCATGGCCTGAAATACAGGGGGAGGCACATCCAACAGTTAGTAGGGTTTTGGGCCGAGACCTCATGGAGCCCAGTGAGGGTGGTATTAAATAGGCTTACCAGGCAAGTATGGGTATGGAGGGTTTCATGTAGTTTTGAGAGATCTAGTCCTTTGTAGGGGCTAGGCGTGCTATGTACTCAGTCAGTTGGGAGATTACTTCCTTTACGTGTTTTTCTCTTGCCTGATCTTGAACTCCACCCCTATCAACATACCAGTAT

>GL-9-K23_H04

TTCACTGCCCACACCCATATGCCCCGCAACTGCTATCACTCTGCCACTCTTTGCATGCATGCAAATACTCATTATTGGACAGGAAAAATGATTAATCCTAGTTGTCCTGGAGGACTTGGAGTCACTGTCTGTTGGACTTACTTCACCCAAACTGGTATGTCTGATGGGGGTGGAGTTCAAGATCAGGCAAGAGAAAAACATGTAAAAGAAGTAATCTCCCAACTCACCCGGGTACATGGCACCTCTAGCCCCTACAAAGGACTAGATCTCTCAAAACTACATGAAACCCTCCGTACCCATACTCGCCTGGTAAGCCTATTTAATACCACCCTCACTGGGCTCCATGAGGTCTCGGCCCAAAACCCTACTAACTGTTGGATATGCCTCCCCCTGAACTTCAGGCCATATGTTTCAATCCCTGTACCTGAACAATGGAACAACTTCAGCACAGAAATAAACACCACTTCCGTTTTAGTAGGACCTCTTGTTTCCAATCTGGAAATAACCCATACCTCAAACCTCACCTGTGTAAAATTTAGCAATACTACATACACAACCAACTCCCAATGCATCAGGTGGGTAACTCCTCCCACACAAATAGTCTGCCTACCCTCAGGAATATTTTTTGTCTGTGGTACCTC

>GL-9-K26_A05

GAGGTACCACAGACAAAAAATATTCCTGAGGGTAGGCAGACTATTCGTGGGGGGGAGTTACCCACCTGATGCATTGGGAGTTGGTTGTGTCTATCGTATTGCTAAATTTTACACAGGTGAGGTTTGAGGTATGGGTTATTTCCAGATTGGAAACAAGAGGTCCTACTAAAACGGAAGTGGTGTTTATGTGTTGAAGTTGTTCCCTTGTTCAGGTACAGGGATTGAAATGTATGGCCTTAAGTGCAGAGGGAGGCACATCCAACAGTTAGTAGGGTTTTGGGCCGAGACCTCATGAAACCCAGTGAGGGTGGTATTAAATAGGCTTACCAGGCAAGTATGGGTATGGAGGGTTTCATGTAGTTTTAAGAGATCTAGTCCTTTGTAGGGGCTAGGGGTGCTATGTACCCGGGTCAGTTGGGAGGTTACTTCCTTTACATGTTTTTCTCTTGCCTGATCTTGAACTCCACCCCTATCAGACATACCAGTATGGGTGAACTAAGTCCAACAGACAGTGGCTCCAAGTCCTCCAGGACAACTAGGATTAATCATTTTCCCTGTCCAATAATGAGTATTTGCATGCATGCAAAGAGTGGCAGAGTTATAGCAGTTGTGGGGCATATGGGTGTGGGCAGTGAA

>GL-9-K31_B05

GGTACCACAGACAAAAAATATTCCTGAGGGTAGGCAGACTATTCGTGGGGGGGAGTTACCCACCTGATGCATTGGGAGTTGGTTGTGTCTATCGTATTGCTAAATTTTACACAGGTGAGGTTTGAGGTATGGGTTATTTCCAGATTGGAAACAAGAGGTCCTACTAAAACGGAAGTGGTGTTTATTTCTGTGCTGTAGTTGTTCCATTGTTCAGGTACAGGGATTGAAATGCATGGCCTGAAATACAGGGGGAGGCACATCCAACAGTTAGTAGGGTTTTGGGCCGAGACCTCATGAAACCCAGTGAGGGTGGTATTAAATAGGCTTACCAGGCGAGTATGGGTACGGAGGGTTTCATGTAGTTTTGAGAGATCTAGTCCTTTGTAGGGGCTAGGCGTGCTATGTACTCAGGTCAGTTGGGAGATTACTTCCTTTACGTGTTTTTCTCTTGCCTGATCTTGAACTCCACCCCTATCAGACATACCAGTATGGGTGAACTAAGTCCAACAGACAGTGGCTCCAAGTCCTCCAGGACAACTAGGATTAATCATTTTCCCTGTCCAATAATGAGTATTTGCATGCATGCAAAGAGTGGCAGAGTTATAGCAGTTGTGGGGCATATGGGTGTGGGCAGTGAA

>GL-9-K32_C05

GAGGTACCACAGACAAAAAATATTCCTGAGGGTAGGCAGACTATTCGTGGGGGGGAGTTACCCACCTGATGCATTGGGAGTTGGTTGTGTCTATCGTATTGCTAAATTTTACACAGGTGAGGTTTGAGGTATGGGTTATTTCCAGATTGGAAACAAGAGGTCCTACTAAAACGGAAGTGGTGTTTATGTGTTGAAGTTGTTCCCTTGTTCAGGTACAGGGATTGAAATGTATGGCCTTAAGTGCAGAGGGAGGCACATCCAACAGTTAGTAGGGTTTTGGGCCGAGACCTCATGAAACCCAGTGAGGGTGGTATTAAATAGGCTTACCAGGCGAGTATGGGTACGGAGGGTTTCATGTAGTTTTGAGAGATCTAGTCCTTTGTAGGGGCTAGGCGTGCTATGTACTCAGGTCAGTTGGGAGATTACTTCCTTTACGTGTTTTTCTCTTGCCTGATCTTGAACTCCACCCCTATCAGACATACCAGTATGGGTGAACTAAGTCCAACAGACAGTGGCTCCAAGTCCTCCAGGACAACTAGGATTAATCATTTTCCCTGTCCAATAATGAGTATTTGCATGCATGCAAAGAGTGGCAGAGTTATAGCAGTTGTGGGGCATATGGGTGTGGGCAGTGAA

>GL-9-K33_D05

TTCACTGCCCACACCCATATGCCCCACAACTGCTATAACTCTGCCACTCTTTGCATGCATGCAAATACTCATTATTGGACAGGGAAAATGATTAATCCTAGTTGTCCTGGAGGACTTGGAGCCACTGTCTGTTGGACTTAGTTCACCCATACTGGTATGTCTGATAGGGGTGGAGTTCAAGATCAGGCAAGAGAAAAACACGTAAAGGAAGTAATCTCCCAACTGACCTGAGTACATAGCACGCCTAGCCCCTACAAAGGACTAGATCTCTCAAAACTACATGAAACCCTCCGTACCCATACTCGCCTGGTAAGCCTATTTAATACCACCCTCACTGGGTTTCATGAGGTCTCGGCCCAAAACCCTACTAACTGTTGGATGTGCCTCCCTCTGCACTTAAGGCCATACATTTCAATCCCTGTACCTGAACAAGGGAACAACTTCAACACATAAACACCACTTCCGTTTTAGTAGGACCTCTTGTTTCCAATCTGGAAATAACCCATACCTCAAACCTCACCTGTGTAAAATTTAGCAATACGATAGACACAACCAACTCCCAATGCATCAGGTGGGTAACTCCCCCCCACGAATAGTCTGCCTACCCTCAGGAATATTTTTTGTCTGTGGTACCTC

>GL-9-K34_E05

GAGGTACCACAGACAAAAAATATTCCTGAGGGTAGGCGGACTATTCGTGGGGGGGAGTTACCCACCTGATGCATTGGGAGTTGGTTGTGTCTATCGTATTGCTAAATTTTACACAGGTGAGGTTTGAGGTATGGGTTATTTCCAGATTGGAAACAAGAGGTCCTACTAAAACGGAAGCGGTGTTTATGTGTTGAAGTTGTTCCCTTGTTCAGGTACAGGGATTGAAATGTATGGCCTTAAGTGCAGAGGGAGGCACATCCAACAGTTAGTAGGGTTTTGGGCCGAGACCTCATGAAACCCAGTGAGGGTGGTATTAAATAGGCTTACCAGGCGAGTATGGGTACGGAGGGTTTCATGTAGTTTTGAGAGATCTAGTCCTTTGTAGGGGCTAGGCGTGCTATGTACTCAGGTCAGTTGGGAGATTACCTCCTTTACGTGTTTTTCTCTTGCCTGATCTTGAACTCCACCCCTATCAGACATACCAGTATGGGTGAACTAAGTCCAACAGACAGTGGCTCCAAGTCCTCCAGGACAACTAGGATTAATCATTTTCCCTGTCCAATAATGAGTATTTGCATGCATGCAAAGAGTGGCAGAGTTATAGCAGTTGTGGGGCATATGGGTGTGGGCAGTGAA

>GL_9_K35_F05

TTCACTGCCCACACCCATATGCCCCACAACTGCTATAACTCTGCCACTCTTTGCATGCATGCAAATACTCATTATTGGACAGGGAAAATGATTAATCCTAGTTGTCCTGGAAGACTTGGAGCCACTGTCTGTCGGACTTACTTCACCCATACTGGTATGTCTGAAGGGGGTGGAGTTCAAGATCAGGCAAGAGAGAAACACGTAAAGGAAGTAATCTCCCAACTGACCTGAGTACATAGCACGCCTAGCCCCTACAAAGGACTAGATCTCTCAAAACTACATGAAACCCTCCGTACCCATACTCGCCTGGTAAGCCTATTTAATACCACCCTCACTGGGTTTCATGAGGTCTCGGCCCAAAACCCTACTAACTGTTGGATGTGCCTCCCTCTGCACTTAAGGCCATACATTTCAATCCCTGTACCTGAACAAGGGAACAACTTCAACACATAAACACCACTTCCGTTTTAGTAGGACCTCTTGTTTCCAATCTGGAAATAACCCATACCTCAAACCTCACCTGTGTAAAATTTAGCAATACGATAGACACAACCAACTCCCAATGCATCAGGTGGGTAACTCCCCCCCACGAATAGTCTGCCTACCCTCAGGAATATTTTTTGTCTGTGGTACCTC

>GL-9-K36_G05

GAGGTACCACAGACAAAAAATATTCCTGAGGGTAGGCAGACTATTCGTGGGGGGGAGTTACCCACCTGATGCATTGGGAGTTGGTTGTGTCTATCGTATTGCTAAATTTTACACAGGTGAGGTTTGAGGTATGGGTTATTTCCAGATTGGAAACAAGAGGTCCTACTAAAACGGAAGTGGTGTTTATGTGTTGAAGTTGTTCCCTTGTTCAGGTACAGGGATTGAAATGTATGGCCTTAAGTGCAGAGGGAGGCACATCCAACAGTTAGTAGGGTTTTGGGCCGAGACCTCATGAAACCCAGTGAGGGTGGTATTAAATAGGCTTACCAGGCGAGTATGGGTACGGAGGGTTTCATGTAGTTTTGAGAGATCTAGTCCTTTGTAGGGGCTAGGCGTGCTATGTACTCAGGTCAGTTGGGAGATTACTTCCTTTACGTGTTTTTCTCTTGCCTGATCTTGAACTCCACCCCTATCAGACATACCAGTATGGGTGAAGTAAGTCCAACAGACAGTGGCTCCAAGTCCTCCAGGACAACTAGGATTAATCATTTTCCCTGTCCAATAATGAGTATTTGCATGCATGCAAAGAGTGGCAGAGTTATAGCAGTTGCGGGGCATATGGGTGTGGGCAGTGAA

>GL-9-K40_H05

TTCACTGCCCACACCCATATGCCCCACAACTGCTATAACTCTGCCACTCTTTGCATGCATGCAAATACTCATTATTGGACAGGGAAAATGATTAATCCTAGTTGTCCTGGAGGACTTGGAGCCACTGTCTGTTGGACTTAGTTCACCCATACTGGTATGTCTGATAGGGGTGGAGTTCAAGATCAGGCAAGAGAAAAACACGTAAAGGAAGTAATCTCCCAACTGACCTGAGTACATAGCACGCCTAGCCCCTACAAAGGACTAGATCTCTCAAAACTACATGAAACCCTCCGTACCCATACTCGCCTGGTAAGCCTATTTAATACCACCCTCACTGGGTTTCATGAGGTCTCGGCCCAAAACCCTACTAACTGTTGGATGTGCCTCCCTCCGCACTTAAGGCCATACATTTCAATCCCTGTACCTGAACAAGGGAACAACTTCAACACATAAACACCACTTCCGTTTTAGTAGGACCTCTTGTTTCCAATCTGGAAATAACCCATACCTCAAACCTCACCTGTGTAAAATTTAGCAATACGATAGACACAACCAACTCCCAATGCATCAGGTGGGTAACTCCCCCCCACGAATAGTCTGCCTACCCTCAGGAATATTTTTTGTCTGTGGTACCTC

>GL-9-K43_A06

TTCACTGCCCACACCCATATGCCCCACAACTGCTATAACTCTGCCACTCTTTGCATGCATGCAAATACTCATTATTGGACAGGAAAAATGATTAATCCTAGTTGTCCTGGAGGACTTGGAGTCACTGTCTGTTGGACTTACTTCACCCAAACTGGTATGTCTGATGGGGGTGGAGTTCAAGATCAGGCAAGAGAAAAACATGTAAAAGAAGTAATCTCCCAACTCACCCGGGTACATGGCACCTCTAGCCCCTACAAAGGACTAGATCTCTCAAAACTACATGAAACCCTCCATACCCATACTTGCCTGGTAAGCCTATTTAATACCACCCTCACTGGGCTCCATGAGGTCTCGGCCCAAAACCCTACTAACTGTTGGATGTGCCTCCCCCTGTATTTCAGGCCATGCATTTCAATCCCTGTACCTGAACAATGGAACAACTACAGCACAGAAATAAACACCACTTCCGTTTTAGTAGGACCTCTTGTTTCCAATCTGGAAATAACCCATACCTCAAACCTCACCTGTGTAAAATTTAGCAATACTGTAGACACAACCAACTCCCAATGCATCAGGTGGGTAACTCCTCCCACACGAATAGTCTGCCTACCCTCAGGAATATTTTTTGTCTGTGGTACCTC

>GL-9-K46_C06

GAGGTACCACAGACAAAAAATATTCCTGAGGGTAGGCAGACTATTCGTGGGGGGGAGTTACCCACCTGATGCATTGGGAGTTGGTTGTGTCTATCGTATTGCTAAATTTTACACAGGTGAGGTTTGAGGTATGGGTTATTTCCAGATTGGAAACAAGAGGTCCTACTAAAACGGAAGTGGTGTTTATGTGTTGAAGTTGTTCCCTTGTTCAGGTACAGGGATTGAAATGTATGGCCTTAAGTGCAGAGGGAGGCACATCCAACAGTTAGTAGGGTTTTGGGCCGAGACCTCATGAAACCCAGTGAGGGTGGTATTAAATAGGCTTACCAGGCGAGTATGGGTACGGAGGGTTTCATGTAGTTTTGAGAGATCTAGTCCTTTGTAGGGGCTAGGCGTGCTATGTACTCAGGTCAGTTGGGAGATTACTTCCTTTACGTGTTTTTCTCTTGCCTGATCTTGAACTCCACCCCCTTCAGACATACCAGTATGGGTGAACTAAGTCCAACAGACAGTGGCTCCAAGTCCTCCAGGACAACTAGGATTAATCATTTTCCCTGTCCAATAATGAGTATTTGCATGCATGCAAAGAGTGGCAGAGTTATAGCAGTTGTGGGGCATATGGGTGTGGGCAGTGAA

>GL-9-K57_D06

GAGGTACCACAGACAAAAAATATTCCTGAGGGTAGGCAGACTATTCGTGGGGGGGAGTTACCCACCTGATGCATTGGGAGTTGGTTGTGTCTATCGTATTGCTAAATTTTACACAGGTGAGGTTTGAGGTATGGGTTATTTCCAGATTGGAAACAAGAGGTCCTACTAAAACGGAAGTGGTGTTTATGTGTTGAAGTTGTTCCCTTGTTCAGGTACAGGGATTGAAATGTATGGCCTTAAGTGCAGAGGGAGGCACATCCAACAGTTAGTAGGGTTTTGGGCCGAGACCTCATGAAACCCAGTGAGGGTGGTATTAAATAGGCTTACCAGGTGAGTATGGGTACGGAGGGTTTCATGTAGTTTTGAGAGAACTAGTCCTTTGTAGGGGCTAGGGGTGCTATGTCCCCGGGTCAGTTGGGAGATTGCTTCCTTTACTCGTTTTTCTCTTGCCTGACCTTGAATTCCACCCCCATCAGACATACTGGTATGGGTGAAGTAAGTCCAACAGACAGTGGCTCCAAGTCCTCCAGGACAACTAGGATTAATCATTTTCCCTGTCCAATAATGAGTATTTGCATGCATGCAAAGAGTGGCAGAGTTATAGCAGTTGTGGGGCATATGGGTGTGGGCAGTGAA

>GL-9-K59_E06

TTCACTGCCCACACCCATATGCCCCACAACTGCTATAACTCTGCCACTCTTTGCATGCATGCAAATACTCATTATTGGACAGGGAAAATGATTAATCCTAGTTGTCCTGGAGGACTTGGAGCCACTGTCTGTTGGACTTAGTTCACCCATACTGGTATGTCTGATAGGGGTGGAGTTCAAGATCAGGCAAGAGAAAAACACGTAAAGGAAGTAATCTCCCAACTGACCTGAGTACATAGCACGCCTAGCCCCTACAAAGGACTAGATCTCTCAAAACTACATGAAACCCTCCGTACCCATACTCGCCTGGTAAGCCTATTTAATACCACCCTCACTGGGTTTCATGAGGTCTCGGCCCAAAACCCTACTAACTGTTGGATGTGCCTCCCTCTGCACTTAAGGCCATACATTTCAATCCCTGTACCTGAACAAGGGAACAACTTCAACACATAAACACCACTTCCGTTTTAGTAGGACCTCTTGTTTCCAATCTGGAAATAACCCATACCTCAAACCTCACCTGTGTAAAATTTAGCAATACGATAGACACAACCAACTCCCAATGCATCAGGTGGGTAACTCCCCCCCACGAATAGTCTGCCTACCCTCAGGAATATTTTTTGTCTGTGGTACCTC

>GL-9-K61_F06

GAGGTACCACAGACAAAAAATATTCCTGAGGGTAGGCAGACTATTCGTGGGGGGGAGTTACCCACCTGATGCATTGGGAGTTGGTTGTGTCTATCGTATTGCTAAATTTTACACAGGTGAGGTTTGAGGTATGGGTTATTTCCAGATTGGAAACAAGAGGTCCTACTAAAACGGAAGTGGTGTTTATGTGTTGAAGTTGTTCCCTTGTTCAGGTACAGGGATTGAAATGTATGGCCTTAAGTGCAGAGGGAGGCACATCCAACAGTTAGTAGGGTTTTGGGCCGAGACCTCATGAAACCCAGTGAGGGTGGTATTAAATAGGCTTACCAGGCGAGTATGGGTACGGAGGGTTTCATGTAGTTTTGAGAGATCTAGTCCTTTGTAGGGGCTAGGCGTGCTATGTACTCAGGTCAGTTGGGAGATTACTTCCTTTACGTGTTTTTCTCTTGCCTGATCTTGAACTCCACCCCTATCAGACATACCAGTATGGGTGAACTAAGTCCAACAGACAGTGGCTCCAAGTCCTCCAGGACAACTAGGATTAATCATTTTCCCTGTCCAATAATGAGTATTTGCATGCATGCAAAGAGTGGCAGAGTTATAGCAGTTGTGGGGCATATGGGTGTGGGCAGTGAA

>GL-9-K63_G06

TTCACTGCCCACACCCATATGCCCCACAACTGCTATAACTCTGCCACTCTTTGCATGCATGCAAATACTCATTATTGGACAGGGAAAATGATTAATCCTAGTTGTCCTGGAGGACTTGGAGCCACTGTCTGTTGGACTTAGTTCACCCATACTGGTATGTCTGATAGGGGTGGAGTTCAAGATCAGGCAAGAGAAAAACACGTAAAGGAAGTAATCTCCCAACTGACCTGAGTACATAGCACGCCTAGCCCCTACAAAGGACTAGATCTCTCAAAACTACATGAAACCCTCCGTACCCATACTCGCCTGGTAAGCCTATTTAATACCACCCTCACTGGGTTTCATGAGGTCTCGGCCCAAAACCCTACTAACTGTTGGATGTGCCTCCCTCTGCACTTAAGGCCATACATTTCAATCCCTGTACCTGAACAAGGGAACAACTTCAACACATAAACACCACTTCCGTTTTAGTAGGACCTCTTGTTTCCAATCTGGAAATAACCCATACCTCAAACCTCACCTGTGTAAAATTTAGCAATACGATAGACACAACCAACTCCCAATGCATCAGGTGGGTAACTCCCCCCCACGAATAGTCTGCCTACCCTCAGGAATATTTTTTGTCTGTGGTACCTC

>GL_9_K64_H06

TTCACTGCCCACACCCATATGCCCCACAACTGTTATAACTCTGCCACTCTTTGCATGCATGCAAATACTCATTATTGGACAGGGAAAATGATTAATCCTAGTTGTCCTGGAAGACTTGGAGCCACTGTCTGTCGGACTTACTTCACCCATACTGGTATGTCTGAAGGGGGTGGAGTTCAAGATCAGGCAAGAGAAAAACATGTAAAGGAAGTAACCTCCCAACTGACCCGGGTACATAGCACCCCTAGCCCCTACAAAGGACTAGATCTCTCAAAACTACATGAAACCCTCCGTACCCATACTCGCCTGGTAAGCCTATTTAATACCACCCTCACTGGGTTTCATGAGGTCTCGGCCCAAAACCCTACTAACTGTTGGATGTGCCTCCCTCTGCACTTAAGGCCATACATTTCAATCCCTGTACCTGAACAAGGGAACAACTTCAACACATAAACACCACTTCCGTTTTAGTAGGACCTCTTGTTTCCAATCTGGAAATAACCCATACCTCAAACCTCACCTGTGTAAAATTTAGCAATACGATAGACACAACCAACTCCCAATGCATCAGGTGGGTAACTCCCCCCCACGAATAGTCTGCCTACCCTCAGGAATATTTTTTGTCTGTGGTACCTC

>GL-9-K65_A07

GAGGTACCACAGACAAAAAATATTCCTGAGGGTAGGCAGACTATTCGTGGGGGGGAGTTACCCACCTGATGCATTGGGAGTTGGTTGTGTCTATCGTATTGCTAAATTTTACACAGGTGAGGTTTGAGGTATGGGTTATTTCCAGATTGGAAACAAGAGGTCCTACTAAAACGGAAGTGGTGTTTATGTGTTGAAGTTGTTCCCTTGTTCAGGTACAGGGATTGAAATGTATGGCCTTAAGTGCAGAGGGAGGCACATCCAACAGTTAGTAGGGTTTTGGGCCGAGACCTCATGAAACCCAGTGAGGGTGGTATTAAATAGGCTTACCAGGCGAGTATGGGTACGGAGGGTTTCATGTAGTTTTGAGAGATCTAGTCCTTTGTAGGGGCTAGGCGTGCTATGTACTCAGGTCAGTTGGGAGATTACTTCCTTTACGTGTTTTTCTCTTGCCTGATCTTGAACTCCACCCCTATCAGACATACCAGTATGGGTGAACTAAGTCCAACAGACAGTGGCTCCAAGTCCTCCAGGACAACTAGGATTAATCATTTTCCCTGTCCAATAATGAGTATTTGCATGCATGCAAAGAGTGGCAGAGTTATAGCAGTTGCGGGGCATATGGGTGTGGGCAGTGAA

>GL-9-K67_B07

TTCACTGCCCACACCCATATGCCCCGCAACTGCTATCACTCTGCCACTCTTTGCATGCATGCAAATACTCATTATTGGACAGGAAAAATGATTAATCCTAGTTGTCCTGGAGGACTTGGAGTCACTGTCTGTTGGACTTACTTCACCCAAACTGGTATGTCTGATGGGGGTGGAGTTCAAGATCAGGCAAGAGAAAAACATGTAAAAGAAGTAATCTCCCAACTCACCCGGGTACATGGCACCTCTAGCCCCTACAAAGGACTAGATCTCTCAAAACTACATGAAACCCTCCGTACCCATACTCGCCTGGTAAGCCTATTTAATACCACCCTCACTGGGCTCCATGAGGTCTCGGCCCAAAACCCTACTAACTGTTGGATATGCCTCCCCCTGAACTTCAGGCCATATGTTTCAATCCCTGTACCTGAACAATGGAACAACTTCAGCACAGAAATAAACACCACTTCCGTTTTAGTAGGACCTCTTGTTTCCAATCTGGAAATAACCCATACCTCAAACCTCACCTGTGTAAAATTTAGCAATACTACATACACAACCAACTCCCAATGCATCAGGTGGGTAACTCCTCCCACACAAATAGTCTGCCTACCCTCAGGAATATTTTTTGTCTGTGGTACCTC

>GL_9_K69_C07

TTCACTGCCCACACCCATATGCCCCGCAACTGCTATCACTCTGCCACTCTTTGCATGCATGCAAATACTCATTATTGGACAGGAAAAATGATTAATCCTAGTTGTCCTGGAGGACTTGGAGTCACTGTCTGTTGGACTTACTTCACCCAAACTGGTATGTCTGATGGGGGTGGAGTTCAAGATCAGGCAAGAGAAAAACATGTAAAAGAAGTAATCTCCCAACTCACCCGGGTACATGGCACCTCTAGCCCCTACAAAGGACTAGATCTCTCAAAACTACATGAAACCCTCCGTACCCATACTCGCCTGGTAAGCCTATTTAATACCACCCTCACTGGGCTCCATGAGGTCTCGGCCCAAAACCCTACTAACTGTTGGATATGCCTCCCCCTGAACTTCAGGCCATATGTTTCAATCCCTGTACCTGAACAATGGAACAACTTCAGCACAGAAATAAACACCACTTCCGTTTTAGTAGGACCTCTTGTTTCCAATCTGGAAATAACCCATACCTCAAACCTCACCTGTGTAAAATTTAGCAATACGATAGACACAACCAACTCCCAATGCATCAGGTGGGTAACTCCCCCCCACGAATAGTCTGCCTACCCTCAGGAATATTTTTTGTCTGTGGTACCTC

>GL-9-K70_D07

TTCACTGCCCACACCCATATGCCCCACAACTGCTATAACTCTGCCACTCTTTGCATGCATGCAAATACTCATTATTGGACAGGGAAAATGATTAATCCTAGTTGTCCTGGAGGACTTGGAGCCACTGTCTGTTGGACTTAGTTCACCCATACTGGTATGTCTGATAGGGGTGGAGTTCAAGATCAGGCAAGAGAAAAACACGTAAAGGAAGTAATCTCCCAACTGACCTGAGTACATAGCACGCCTAGCCCCTACAAAGGACTAGATCTCTTAAAACTACATGAAACCCTCCATACCCATACTTGCCTGGTAAGCCTATTTAATACCACCCTCACTGGGCTCCATGAGGTCTCGGCCCAAAACCCTACTAACTGTTGGATGTGCCTCCCCCTGTATTTCAGGCCATGCATTTCAATCCCTGTACCTGAACAATGGAACAACTACAGCACAGAAATAAACACCACTTCCGTTTTAGTAGGACCTCTTGTTTCCAATCTGGAAATAACCCATACCTCAAACCTCACCTGTGTAAAATTTAGCAATACTGTAGACACAACCAACTCCCAATGCATCAGGTGGGTAACTCCCCCCCACGAATAGTCTGCCTACCCTCAGGAATATTTTTTGTCTGTGGTACCTC

>GL-9-K72_E07

GAGGTACCACAGACAAAAAATATTCCTGAGGGTAGGCAGACTATTCGTGGGGGGGAGTTACCCACCTGATGCATTGGGAGTTGGTTGTGTCTATCGTATTGCTAAATTTTACACAGGTGAGGTTTGAGGTATGGGTTATTTCCAGATTGGAAACAAGAGGTCCTACTAAAACGGAAGTGGTGTTTATGTGTTGAAGTTGTTCCCTTGTTCAGGTACAGGGATTGAAATGTATGGCCTTAAGTGCAGAGGGAGGCACATCCAACAGTTAGTAGGGTTTTGGGCCGAGACCTCATGAAACCCAGTGAGGGTGGTATTAAATAGGCTTACCAGGCGAGTATGGGTACGGAGGGTTTCATGTAGTTTTGAGAGATCTAGTCCTTTGTAGGGGCTAGGCGTGCTATGTACTCAGGTCAGTTGGGAGATTACTTCCTTTACGTGTTTTTCTCTTGCCTGATCTTGAACTCCACCCCTATCAGACATACCAGTATGGGTGAACTAAGTCCAACAGACAGTGGCTCCAAGTCCTCCAGGACAACTAGGATTAATCATTTTCCCTGTCCAATAATGGGTATTTGCATGCTTGCAAAGAGTGGCAGAGTTATAGCAATTGTGGGGCATATGGGTGTGAGCAGTGAA

>GL_9_K74_F07

TTCACTGCCCACACCCATATGCCCCACAACTGCTATAACTCTGCCACTCTTTGCATGCATGCAAATACTCATTATTGGACAGGGAAAATGATTAATCCTAGTTGTCCTGGAGGACTTGGAGCCACTGTCTGTTGGACTTAGTTCACCCATACTGGTATGTCTGATAGGGGTGGAGTTCAAGATCAGGCAAGAGAAAAACACGTAAAGGAAGTAATCTCCCAACTGACCTGAGTACATAGCACGCCTAGCCCCTACAAAGGACTAGATCTCTCAAAACTACATGAAACCCTCCGTACCCATACTCGCCTGGTAAGCCTATTTAATACCACCCTCACTGGGTTTCATGAGGTCTCGGCCCAAAACCCTACTAACTGTTGGATGTGCCTCCCTCTGCACTTAAGGCCATACATTTCAATCCCTGTACCTGAACAAGGGAACAACTTCAACACATAAACACCACTTCCGTTTTAGTAGGACCTCTTGTTTCCAATCTGGAAATAACCCATACCTCAAACCTCACCTGTGTAAAATTTAGCAATACGATAGACACAACCAACTCCCAATGCATCAGGTGGGTAACTCCCCCCCACGAATAGTCTGCCTACCCTCAGGAATATTTTTTGTCTGTGGTACCTC

**KO-II**

>GL-13-K1_T7_F08

GAGGTACCACAGACAAAAAATATTCCTGAGGGTAGGCAGACTATTCGTGGGGGGGAGTTACCCACCTGATGCATTGGGAGTTGGTTGTGTCTATCGTATTGCTAAATTTTACACAGGTGAGGTTTGAGGTATGGGTTATTTCCAGATTGGAAACAAGAGGTCCTACTAAAACGGAAGTGGTGTTTATGTGTTGAAGTTGTTCCCTTGTTCAGGTACAGGGATTGAAATGTATGGCCTTAAGTGCAGAGGGAGGCACATCCAACAGTTAGTAGGGTTTTGGGCCGAGACCTCATGAAACCCAGTGAGGGTGGTATTAAATAGGCTTACCAGGCGAGTATGGGTACGGAGGGTTTCATGTAGTTTTGAGAGATCTAGTCCTTTGTAGGGGCTAGGCGTGCTATGTACTCAGGTCAGTTGGGAGATTACTTCCTTTACGTGTTTTTCTCTTGCCTGATCTTGAACTCCACCCCTATCAGACATACCAGTATGGGTGAACTAAGTCCAACAGACAGTGGCTCCAAGTCCTCCAGGACAACTAGGATTAATCATTTTCCCTGTCCAATAATGAGTATTTGCATGCATGCAAAGAGTGGCAGAGTTATAGCAGTTGTGGGGCATATGGGTGTGGGCAGTGAA

>GL-13-K2_T7_G08

TTCACTGCCCACACCCATATGCCCCACAACTGCTATAACTCTGCCACTCTTTGCATGCATGCAAATACTCATTATTGGACAGGGAAAATGATTAATCCTAGTTGTCCTGGAGGACTTGGAGCCACTGTCTGTTGGACTTAGTTCACCCATACTGGTATGTCTGATAGGGGTGGAGTTCAAGATCAGGCAAGAGAAAAACACGTAAAGGAAGTAATCTCCCAACTGACCTGAGTACATAGCACGCCTAGCCCCTACAAAGGACTAGATCTCTCAAAACTACATGAAACCCTCCGTACCCATACTCGCCTGGTAAGCCTATTTAATACCACCCTCACTGGGTTTCATGAGGTCTCGGCCCAAAACCCTACTAACTGTTGGATGTGCCTCCCTCTGCACTTAAGGCCATACATTTCAATCCCTGTACCTGAACAAGGGAACAACTTCAACACATAAACACCACTTCCGTTTTAGTAGGACCTCTTGTTTCCAATCTGGAAATAACCCATACCTCAAACCTCACCTGTGTAAAATTTAGCAATACGATAGACACAACCAACTCCCAATGCATCAGGTGGGTAACTCCCCCCCACGAATAGTCTGCCTACCCTCAGGAATATTTTTTGTCTGTGGTACCTC

>GL-13-K4_T7_H08

GAGGTACCACAGACAAAAAATATTCCTGAGGGTAGGCAGACTATTCGTGGGGGGGAGTTACCCACCTGATGCATTGGGAGTTGGTTGTGTCTATCGTATTGCTAAATTTTACACAGGTGAGGTTTGAGGTATGGGTTATTTCCAGATTGGAAACAAGAGGTCCTACTAAAACGGAAGTGGTGTTTATGTGTTGAAGTTGTTCCCTTGTTCAGGTACAGGGATTGAAATGTATGGCCTTAAGTGCAGAGGGAGGCACATCCAACAGTTAGTAGGGTTTTGGGCCGAGACCTCATGAAACCCAGTGAGGGTGGTATTAAATAGGCTTACCAGGCGAGTATGGGTACGGAGGGTTTCATGTAGTTTTGAGAGATCTAGTCCTTTGTAGGGGCTAGGCGTGCTATGTACTCAGGTCAGTTGGGAGATTACTTCCTTTACGTGTTTTTCTCTTGCCTGATCTTGAACTCCACCCCTATCAGACATACCAGTATGGGTGAAGTAAGTCCGACAGACAGTGGCTCCAAGTCTTCCAGGACAACTAGGATTAATCATTTTCCCTGTCCAATAATGAGTATTTGCATGCATGCAAAGAGTGGCAGAGTTATAGCAGTTGTGGGGCATATGGGTGTGGGCAGTGAA

>GL-13-K5_T7_A09

GAGGTACCACAGACAAAAAATATTCCTGAGGGTAGGCAGACTATTCGTGGGGGGGAGTTACCCACCTGATGCATTGGGAGTTGGTTGTGTCTATCGTATTGCTAAATTTTACACAGGTGAGGTTTGAGGTATGGGTTATTTCCAGATTGGAAACAAGAGGTCCTACTAAAACGGAAGTGGTGTTTATGTGTTGAAGTTGTTCCCTTGTTCAGGTACAGGGATTGAAATGTATGGCCTTAAGTGCAGAGGGAGGCACATCCAACAGTTAGTAGGGTTTTGGGCCGAGACCTCATGAAACCCAGTGAGGGTGGTATTAAATAGGCTTACCAGGCGAGTATGGGTACGGAGGGTTTCATGTAGTTTTGAGAGATCTAGTCCTTTGTAGGGGCTAGGCGTGCTATGTACTCAGGTCAGTTGGGAGATTACTTCCTTTACGTGTTTTTCTCTTGCCTGATCTTGAACTCCACCCCTATCAGACATACCAGTATGGGTGAACTAAGTCCAACAGACAGTGGCTCCAAGTCCTCCAGGACAACTAGGATTAATCATTTTCCCTGTCCAATAATGAGTATTTGCATGCATGCAAAGAGTGGCAGAGTTATAGCAGTTGTGGGGCATATGGGTGTGGGCAGTGAA

>GL-13-K6_T7_B09

TTCACTGCCCACACCCATATGCCCCACAACTGCTATAACTCTGCCACTCTTTGCATGCATGCAAATACTCATTATTGGACAGGGAAAATGATTAATCCTAGTTGTCCTGGAAGACTTGGAGCCACTGTCTGTCGGACTTACTTCACCCATACTGGTATGTCTGAGGGGGGTGGAGTTCAAGATCAGGCAAGAGAAAAACATGTAAAGGAAGTAACCTCCCAACTGACCCGGGTACATAGCACCCCTAGCCCCTACAAAGGACTAGATCTCTTAAAACTACATGAAACCCTCCATACCCATACTTGCCTGGTAAGCCTATTTAATACCACCCTCACTGGGTTTCATGAGGTCTCGGCCCAAAACCCTACTAACTGTTGGATGTGCCTCCCTCTGCACTTAAGGCCATACATTTCAATCCCTGTACCTGAACAAGGGAACAACTTCAACACATAAACACCACTTCCGTTTTAGTAGGACCTCTTGTTTCCAATCTGGAAATAACCCATACCTCAAACCTCACCTGTGTAAAATTTAGCAATACGATAGACACAACCAACTCCCAATGCATCAGGTGGGTAACTCCCCCCCACGAATAGTCTGCCTACCCTCAGGAATATTTTTTGTCTGTGGTACCTC

>GL-13-K7_T7_C09

GAGGTACCACAGACAAAAAATATTCCTGAGGGTAGGCAGACTATTCGTGGGGGGGAGTTACCCACCTGATGCATTGGGAGTTGGTTGTGTCTATCGTATTGCTAAATTTTACACAGGTGAGGTTTGAGGTATGGGTTATTTCCAGATTGGAAACAAGAGGTCCTACTAAAACGGAAGTGGTGTTTATTTCTGTGCTGTAGTTGTTCCATTGTTCAGGTACAGGGATTGAAATGCATGGCCTGAAATACAGGGGGAGGCACATCCAACAGTTAGTAGGGTTTTGGGCCGAGACCTCATGGAGCCCAGTGAGGGTGGTATTAAATAGGCTTACCAGGCAAGTATGGGTATGGAGGGTTTCATGTAGTTTTAAGAGATCTAGTCCTTTGTAGGGGCTAGGGGTGCTATGTACCCGGGTCAGTTGGGAGGTTACTTCCTTTACATGTTTTTCTCTTGCCTGATCTTGAACTCCACCCCCCTCAGACATACCAGTATGGGTGAAGTAAGTCCGACAGACAGTGGCTCCAAGTCTTCCAGGACAACTAGGATTAATCATTTTCCCTGTCCAATAATGAGTATTTGCATGCATGCAAGGAGTGGCAGAGTTATAGCAGTTGTGGGGCATATGGGTGTGGGCAGTGAA

>GL-13_K9_T7_E09

GAGGTACCACAGACAAAAAATATTCCTGAGGGTAGGCAGACTATTCGTGTGGGAGGTGTTACCCACCTGATGCATTGGGAGCTGGTTGTGTCTATAGTATTGCTAAACTTTACACAGGTGAGGTTTGAGGTATGGGTTATTTCCAGATTGGAAACAAGAGGTCCTACTAAAGCGGAAGTGGTGTTTATGTGTTGAAGTTGTTCCCTTGTTCAGGTACAGGGATTGAAATGTATGGCCTTAAGTGCAGAGGGAGGCACATCCAACAGTTAGTAGGGTTTTGGGCCGAGACCTCATGAAACCCAGTGAGGGTGGTATTAAATAGGCTTACCAGGCGAGTATGGGTACGGAGGGTTTCATGTAGTTTTGAGAGATCTAGTCCTTTGTAGGGGCTAGAGGTGCCATGTACCCGGGTGAGTTGGGAGATTACTTCTTTTACATGTTTTTCTCTTGCCTGATCTTGAACTCCACCCCTATCAGACATACCAGTTTGGGTGAAGTAAGTCCAACAGACAGTGACTCCAAGTCCTCCAGGACAACTAGGATTAATCATTTTTCCTGTCCAATAATGAGTATTTGCATGCATGCAAAGAGTGGCAGAGTTATAGCAGTTGTGGGGCATATGGGTGTGGGCAGTGAA

>GL-13-K10_T7_F09

GAGGTACCACAGACAAAAAATATTCCTGAGGGTAGGCAGACTATTCGTGGGGGGGAGTTACCCACCTGATGCATTGGGAGTTGGTTGTGTCTACAGTATTGCTAAATTTTACACAGGTGAGGTTTGAGGTATGGGTTATTTCCAGATTGGAAACAAGAGGTCCTACTAAAACGGAAGTGGTGTTTATTTCTGTGCTGTAGTTGTTCCATTGTTCAGGTACAGGGATTGAAACATATGGCCTGAAGTTCAGGGGGAGGCATATCCAACAGTTAGTAGGGTTTTGGGCCGAGACCTCATGGAGCCCAGTGAGGGTGGTATTAAATAGGCTTACCAGGCGAGTATGGGTACGGAGGGTTTCATGTAGTTTTGAGAGATCTAGTCCTTTGTAGGGGCTAGAGGTGCCATGTACCCGGGTGAGTTGGGAGATTACTTCTTTTACATGTTTTTCTCTTGCCTGATCTTGAACTCCACCCCCATCAGACATACCAGTTTGGGTGAAGTAAGTCCAACAGACAGTGACTCCAAGTCCTCCAGGACAACTAGGATTAATCATTTTTCCTGTCCAATAATGAGTATTTGCATGCATGCAAAGAGTGGCAGAGTGATAGCAGTTGCGGGGCATATGGGTGTGGGCAGTGAA

>GL-13_K11_T7_G09

TTCACTGCCCACACCCATATGCCCCACAACTGCTATAACTCTGCCACTCTTTGCATGCATGCAAATACTCATTATTGGACAGGGAAAATGATTAATCCTAGTTGTCCTGGAAGACTTGGAGCCACTGTCTGTCGGACTTACTTCACCCATACTGGTATGTCTGAGGGGGGTGGAGTTCAAGATCAGGCAAGAGAAAAACATGTAAAGGAAGTAACCTCCCAACTGACCCGGGTACATAGCACCCCTAGCCCCTACAAAGGACTAGATCTCTTAAAACTACATGAAACCCTCCATACCCATACTTGCCTGGTAAGCCTATTTAATACCACCCTCACTGGGCTCCATGAGGTCTCGGCCCAAAACCCTACTAACTGTTGGATGTGCCTCCCCCTGTATTTCAGGCCATGCATTTCAATCCCTGTACCTGAACAATGGAACAACTACAGCACAGAAATAAACACCACTTCCGTTTTAGTAGGACCTCTTGTTTCCAATCTGGAAATAACCCATACCTCAAACCTCACCTGTGTAAAATTTAGCAATACTGTAGACACAACCAACTCCCAATGCATCAGGTGGGTAACTCCTCCCACACGAATAGTCTGCCTACCCTCAGGAATATTTTTTGTCTGTGGTACCTC

>GL-13-K12_T7_H09

GAGGTACCACAGACAAAAAATATTCCTGAGGGTAGGCAGACTATTCGTGTGGGAGGAGTTACCCACCTGATGCATTGGGAGTTGGTTGTGTCTACAGTATTGCTAAATTTTACACAGGTGAGGTTTGAGGTATGGGTTATTTCCAGATTGGAAACAAGAGGTCCTACTAAAACGGAAGTGGTGTTTATTTCTGTGCTGTAGTTGTTCCATTGTTCAGGTACAGGGATTGAAATGCATGGCCTGAAATACAGGGGGAGGCACATCCAACAGTTAGTAGGGTTTTGGGCCGAGACCTCATGAAACCCAGTGAGGGTGGTATTAAATAGGCTTACCAGGCGAGTATGGGTACGGAGGGTTTCATGTAGTTTTGAGAGATCTAGTCCTTTGTAGGGGCTAGGCGTGCTATGTACTCAGGTCAGTTGGGAGATTACTTCCTTTACGTGTTTTTCTCTTGCCTGATCTTGAACTCCACCCCTATCAGACATACCAGTATGGGTGAACTAAGTCCAACAGACAGTGGCTCCAAGTCCTCCAGGACAACTAGGATTAATCATTTTCCCTGTCCAATAATGAGTATTTGCATGCATGCAAAGAGTGGCAGAGTTATAGCAGTTGTGGGGCATATGGGTGTGGGCAGTGAA

>GL-13-K15_T7_A10

GAGGTACCACAGACAAAAAATATTCCTGAGGGTAGGCAGACTATTTGTGTGGGAGGAGTTACCCACCTGATGCATTGGGAGTTGGTTGTGTATGTAGTATTGCTAAATTTTACACAGGTGAGGTTTGAGGTATGGGTTATTTCCAGATTGGAAACAAGAGGTCCTACTAAAACGGAAGTGGTGTTTATGTGTTGAAGTTGTTCCCTTGTTCAGGTACAGGGATTGAAATGTATGGCCTTAAGTGCAGAGGGAGGCACATCCAACAGTTAGTAGGGTTTTGGGCCGAGACCTCATGAAACCCAGTGAGGGTGGTATTAAATAGGCTTACCAGGCGAGTATGGGTACGGAGGGTTTCATGTAGTTTTGAGAGATCTAGTCCTTTGTAGGGGCTAGGCGTGCTATGTACTCAGGTCAGTTGGGAGATTACTTCCTTTACGTGTTTTTCTCTTGCCTGATCTTGAACTCCACCCCTATCAGACATACCAGTATGGGTGAACTAAGTCCAACAGACAGTGGCTCCAAGTCCTCCAGGACAACTAGGATTAATCATTTTCCCTGTCCAATAATGAGTATTTGCATGCATGCAAAGAGTGGCAGAGTTATAGCAGTTGTGGGGCATATGGGTGTGGGCAGTGAA

>GL-13-K16_T7_B10

GAGGTACCACAGACAAAAAATATTCCTGAGGGTAGGCAGACTATTCGTGGGGGGGAGTTACCCACCTGATGCATTGGGAGTTGGTTGTGTCTATCGTATTGCTAAATTTTACACAGGTGAGGTTTGAGGTATGGGTTATTTCCAGATTGGAAACAAGAGGTCCTACTAAAACGGAAGTGGTGTTTATGTGTTGAAGTTGTTCCCTTGTTCAGGTACAGGGATTGAAATGTATGGCCTTAAGTGCAGAGGGAGGCACATCCAACAGTTAGTAGGGTTTTGGGCCGAGACCTCATGAAACCCAGTGAGGGTGGTATTAAATAGGCTTACCAGGCGAGTATGGGTACGGAGGGTTTCATGTAGTTTTGAGAGATCTAGTCCTTTGTAGGGGCTAGGCGTGCTATGTACTCAGGTCAGTTGGGAGATTACTTCCTTTACGTGTTTTTCTCTTGCCTGATCTTGAACTCCACCCCTATCAGACATACCAATATGGGTGAACTAAGTCCAACAGACAGTGGCTCCAAGTCCTCCAGGACAACTAGGATTAATCATTTTCCCTGTCCAATAATGAGTATTTGCATGCATGCAAAGAGTGGCAGAGTTATAGCAGTTGTGGGGCATATGGGTGTGGGCAGTGAA

>GL-13-K17_T7_C10

TTCACTGCCCACACCCATATGCCCCGCAACTGCTATCACTCTGCCACTCTTTGCATGCATGCAAATACTCATTATTGGACAGGAAAAATGATTAATCCTAGTTGTCCTGGAGGACTTGGAGTCACTGTCTGTTGGACTTACTTCACCCAAACTGGTATGTCTGATGGGGGTGGAGTTCAAGATCAGGCAAGAGAAAAACATGTAAAAGAAGTAATCTCCCAACTCACCCGGGTACATGGCACCTCTAGCCCCTACAAAGGACTAGATCTCTCAAAACTACATGAAACCCTCCGTACCCATACTCGCCTGGTAAGCCTATTTAATACCACCCTCACTGGGCTCCATGAGGTCTCGGCCCAAAACCCTACTAACTGTTGGATATGCCTCCCCCTGAACTTCAGGCCATATGTTTCAATCCCTGTACCTGAACAATGGAACAACTTCAGCACAGAAATAAACACCACTTCTGTTTTAGTAGGTCCTCTTTCCAATCTGGAAATAACCCATACCTCAAACCTCACCTGTGTAAAATTTAGCAATACTATAGACACAGCCAACTCCCAATGCATCAGGTGGGTAACTCCTCCCACACGAATAGTCTGCCTACCCTCAAGAATATTTTTTTGTCTGTGGTACCTC

>GL-13-K18_T7_D10

GAGGTACCACAGACAAAAAATATTCCTGAGGGTAGGCAGACTATTCGTGGGGGGGAGTTACCCACCTGATGCATTGGGAGTTGGTTGTGTCTATCGTATTGCTAAATTTTACACAGGTGAGGTTTGAGGTATGGGTTATTTCCAGATTGGAAACAAGAGGTCCTACTAAAACGGAAGTGGTGTTTATGTGTTGAAGTTGTTCCCTTGTTCAGGTACAGGGATTGAAATGTATGGCCTTAAGTGCAGAGGGAGGCACATCCAACAGTTAGTAGGGTTTTGGGCCGAGACCTCATGAAACCCAGTGAGGGTGGTATTAAATAGGCTTACCAGGCGAGTATGGGTACGGAGGGTTTCATGTAGTTTTGAGAGATCTAGTCCTTTGTAGGGGCTAGGGGTGCTATGTCCCCGGGTCAGTTGGGAGATTGCTTCCTTTACTTGTTTTTCTCTTGCCTGACCTTGAATTCCACCCCCATCAGACATACTGGTATGGGTGAAGTAAGTCCAACAGACAGTGGCTCCAAGTCCTCCAGGACAACTAGGATTAATCATTTTCCCTGTCCAATAATGAGTATTTGCATGCATGCAAAGAGTGGCAGAGTTATAGCAGTTGCGGGGCATATGGGTGTGGGCAGTGAA

>GL-13-K21_T7_E10

TTCACTGCCCACACCCATATGCCCCGCAACTGCTGTCACTCTGCCACTCTTTGCATGCATGCAAATACTCATTATTGGACAGGAAAAATGATTAATCCTAGTTGTCCTGGAGGACTTGGAGTCACTGTCTGTTGGACTTACTTCACCCAAACTGGTATGTCTGATGGGGGTGGAGTTCAAGATCAGGCAAGAGAAAAACATGTAAAAGAAGTAATCTCCCAACTCACCCGGGTACATGGCACCTCTAGCCCCTACAAAGGACTAGATCTCTCAAAACTACATGAAACCCTCCGTACCCATACTCGCCTGGTAAGCCTATTTAATACCACCCTCACTGGGCTCCATGAGGTCTCGGCCCAAAACCCTACTAACTGTTGGATATGCCTCCCCCTGAACTTCAGGCCATATGTTTCAATCCCTGTACCTGAACAATGGAACAACTTCAGCACAGAAATAAACACCACTTCCGTTTTAGTAGGACCTCTTGTTTCCAATCTGGAAATAACCCATACCTCAAACCTCACCTGTGTAAAATTTAGCAATACTACATACACAACCAACTCCCAATGCATCAGGTGGGTAACTCCTCCCACACAAATAGTCTGCCTACCCTCAGGAATATTTTTTGTCTGTGGTACCTC

>GL-13-K23_T7_F10

GAGGTACCACAGACAAAAAATATTCCTGAGGGTAGGCAGACTATTCGTGTGGGAGGAGTTACCCACCTGATGCATTGGGAGTTGGTTGTGTCTACAGTATTGCTAAATTTTACACAGGTGAGGTTTGAGGTATGGGTTATTTCCAGATTGGAAACAAGAGGTCCTACTAAAACGGAAGTGGTGTTTATTTCTGTGCTGTAGTTGTTCCATTGTTCAGGTACAGGGATTGAAATGTATGGCCTTAAGTGCAGAGGGAGGCACATCCAACAGTTAGTAGGGTTTTGGGCCGAGACCTCATGAAACCCAGTGAGGGTGGTATTAAATAGGCTTACCAGGCGAGTATGGGTACGGAGGGTTTCATGTAGTTTTGAGAGATCTAGTCCTTTGTAGGGGCTAGGCGTGCTATGTACTCAGGTCAGTTGGGAGATTACTTCCTTTACGTGTTTTTCTCTTGCCTGATCTTGAACTCCACCCCTATCAGACATACCAGTATGGGTGAACTAAGTCCAACAGACAGTGGCTCCAAGTCCTCCAGGACAACTAGGATTAATCATTTTCCCTGTCCAATAATGAGTATTTGCATGCATGCAAAGAGTGGCAGAGTTATAGCAGTTGTGGGGCATATGGGTGTGGGCAGTGAA

>GL-13-K24_T7_G10

GAGGTACCACAGACAAAAAATATTCCTGAGGGTAGGCAGACTATTCGTGGGGGGGAGTTACCCACCTGATGCATTGGGAGTTGGTTGTGTCTATCGTATTGCTAAATTTTACACAGGTGAGGTTTGAGGTATGGGTTATTTCCAGATTGGAAACAAGAGGTCCTACTAAAACGGAAGTGGTGTTTATGTGTTGAAGTTGTTCCCTTGTTCAGGTACAGGGATTGAAATGTATGGCCTTAAGTGCAGAGGGAGGCACATCCAACAGTTAGTAGGGTTTTGGGCCGAGACCTCATGAAACCCAGTGAGGGTGGTATTAAATAGGCTTACCAGGCGAGTATGGGTACGGAGGGTTTCATGTAGTTTTGAGAGATCTAGTCCTTTGTAGGGGCTAGGCGTGCTATGTACTCAGGTCAGTTGGGAGATTACTTCCTTTACGTGTTTTTCTCTTGCCTGATCTTGAACTCCACCCCTATCAGACATACCAGTATGGGTGAACTAAGTCCAACAGACAGTGGCTCCAAGTCCTCCAGGACAACTAGGATTAATCATTTTCCCTGTCCAATAATGAGTATTTGCATGCATGCAAAGAGTGGCAGAGTTATAGCAGTTGTGGGGCATATGGGTGTGGGCAGTGAA

>GL-13-K25_T7_H10

TTCACTGCCCACACCCATATGCCCCACAACTGCTATAACTCTGCCACTCTTTGCATGCATGCAAATACTCATTATTGGACAGGGAAAATGATTAATCCTAGTTGTCCTGGAAGACTTGGAGCCACTGTCTGTCGGACTTACTTCACCCATACTGGTATGTCTGAGGGGGGTGGAGTTCAAGATCAGGCAAGAGAAAAACATGTAAAGGAAGTAACCTCCCAACTGACCCGGGTACATAGCACCCCTAGCCCCTACAAAGGACTAGATCTCTTAAAACTACATGAAACCCTCCATACCCATACTTGCCTGGTAAGCCTATTTAATACCACCCTCACTGGGCTCCATGAGGTCTCGGCCCAAAACCCTACTAACTGTTGGATGTGCCTCCCCCTGTATTTCAGGCCATGCATTTCAATCCCTGTACCTGAACAATGGAACAACTACAGCACAGAAATAAACACCACTTCCGTTTTAGTAGGACCTCTTGTTTCCAATCTGGAAATAACCCATACCTCAAACCTCACCTGTGTAAAATTTAGCAATACTACATACACAACCAACTCCCAATGCATCAGGTGGGTAGCTCCTCCCACACAAATAGTCTGCCTACCCTCAGGAATATTTTTTGTCTGTGGTACCTC

>GL-13.K26_T7_A11

GACTCCGAAGGTTCCAATGCCTCCCGGCCGCCATGGCCGCGGGATTGAGGTACCACAGACAAAAAATATTCCTGAGGGTAGGCAGACTATTCGTGTGGGAGGAGTTACCCACCTGATGCATTGGGAGTTGGTTGTGTCTACAGTATTGCTAAATTTTACACAGGTGAGGTTTGAGGTATGGGTTATTTCCAGATTGGAAACAAGAGGTCCTACTAAAACGGAAGTGGTGTTTATGTGTTGAAGTTGTTCCCTTGTTCAGGTACAGGGATTGAAATGTATGGCCTTAAGTGCAGAGGGAGGCACATCCAACAGTTAGTAGGGTTTTGGGCCGAGACCTCATGAAACCCAGTGAGGGTGGTATTAAATAGGCTTACCAGGCGAGTATGGGTACGGAGGGTTTCATGTAGTTTTGAGAGATCTAGTCCTTTGTAGGGGCTAGAGGTGCCATGTACCCGGGTGAGTTGGGAGATTACTTCTTTTACATGTTTTTCTCTTGCCTGATCTTGAACTCCACCCCCATCAGACATACCAGTTTGGGTGAAGTAAGTCCAACAGACAGTGACTCCAGTCCTCCAGGACAACTAGGATTAATCATTTTTCCTGT

>GL-13_K27_T7_B11

GAGGTACCACAGACAAAAAATATTCCTGAGGGTAGGCAGACTATTCGTGGGGGGGGGTTACCCACCTGATGCATTGGGAGTTGGTTGTGTCTATCGTATTGCTAAATTTTACACAGGTGAGGTTTGAGGTATGGGTTATTTCCAGATTGGAAACAAGAGGTCCTACTAAAACGGAAGTGGTGTTTATGTGTTGAAGTTGTTCCCTTGTTCAGGTACAGGGATTGAAATGTATGGCCTTAAGTGCAGAGGGAGGCACATCCAACAGTTAGTAGGGTTTTGGGCCGAGACCTCATGAAACCCAGTGAGGGTGGTATTAAATAGGCTTACCAGGCGAGTATGGGTACGGAGGGTTTCATGTAGTTTTGAGAGATCTAGTCCTTTGTAGGGGCTAGGCGTGCTATGTACTCAGGTCAGTTGGGAGATTACTTCCTTTACGTGTTTTTCTCTTGCCTGATCTTGAACTCCACCCCTATCAGACATACCAGTATGGGTGAACTAAGTCCAACAGACAGTGGCTCCAAGTCCTCCAGGACAACTAGGATTAATCATTTTCCCTGTCCAATAATGAGTATTTGCATGCATGCAAAGAGTGGCAGA

>GL-13-K28_T7_C11

TTCACTGCCCACACCCATATGCCCCGCAACTGCTATCACTCTGCCACTCTTTGCATGCATGCAAATACTCATTATTGGACAGGAAAAATGATTAATCCTAGTTGTCCTGGAGGACTTGGAGTCACTGTCTGTTGGACTTACTTCACCCAAACTGGTATGTCTGATGGGGGTGGAGTTCAAGATCAGGCAAGAGAAAAACATGTAAAAGAAGTAATCTCCCAACTCACCCGGGTACATGGCACCTCTAGCCCCTACAAAGGACTAGATCTCTCAAAACTACATGAAACCCTCCGTACCCATACTCGCCTGGTAAGCCTATTTAATACCACCCTCACTGGGCTCCATGAGGTCTCGGCCCAAAACCCTACTAACTGTTGGATATGCCTCCCCCTGAACTTCAGGCCATATGTTTCAATCCCTGTACCTGAACAATGGAACAACTTCAGCACAGAAATAAACACCACTTCCGTTTTAGTAGGACCTCTTGTTTCCAATCTGGAAATAACCCATACCTCAAACCTCACCTGTGTAAAATTTAGCAATACTACATACACAACCAACTCCCAATGCATCAGGTGGGTAACTCCTCCCACACAAATAGTCTGCCTACCCTCAGGAATATTTTTTGTCTGTGGTACCTC

>GL-13-K30_T7_D11

AGGTACCACAGACAAAAAATATTCCTGAGGGTAGGCAGACTATTCGTGGGGGGGAGTTACCCACCTGATGCATTGGGAGTTGGTTGTGTCTATCGTATTGCTAAATTTTACACAGGTGAGGTTTGAGGTATGGGTTATTTCCAGATTGGAAACAAGAGGTCCTACTAAAACGGAAGTGGTGTTTATGTGTTGAAGTTGTTCCCTTGTTCAGGTACAGGGATTGAAATGTATGGCCTTAAGTGCAGAGGGAGGCACATCCAACAGTTAGTAGGGTTTTGGGCCGAGACCTCATGAAACCCAGTGAGGGTGGTATTAAATAGGCTTACCAGGCGAGTATGGGTACGGAGGGTTTCATGTAGTTTTGAGAGATCTAGTCCTTTGTAGGGGCTAGGCGTGCTATGTACTCAGGTCAGTTGGGAGATTACTTCCTTTACGTGTTTTTCTCTTGCCTGATCTTGAACTCCACCCCTATCAGACATACCAGTACGGGTGAACTAAGTCCAACAGACAGTGGCTCCAAGTCCTCCAGGACAACTAGGATTAATCATTTTCCCTGTCCAATAATGAGTATTTGCATGCATGCAAAGAGTGGCAGAGTTATAGCAGTTGTGGGGCATATGGGTGTGGGCAGTGAA

>GL-13-K32_T7_E11

GAGGTACCACAGACAAAAAATATTCCTGAGGGTAGGCAGACTATTCGTGGGGGGGAGTTACCCACCTGATGCATTGGGAGTTGGTTGTGTCTATCGTATTGCTAAATTTTACACAGGTGAGGTTTGAGGTATGGGTTATTTCCAGATTGGAAACAAGAGGTCCTACTAAAACGGAAGTGGTGTTTATGTGTTGAAGTTGTTCCCTTGTTCAGGTACAGGGATTGAAATGTATGGCCTTAAGTGCAGAGGGAGGCACATCCAACAGTTAGTAGGGTTTTGGGCCGAGACCTCATGAAACCCAGTGAGGGTGGTATTAAATAGGCTTACCAGGCGAGTATGGGTACGGAGGGTTTCATGTAGTTTTGAGAGATCTAGTCCTTTGTAGGGGCTAGGCGTGCTATGTACTCAGGTCAGTTGGGAGATTACTTCCTTTACGTGTTTTTCTCTTGCCTGATCTTGAACTCCACCCCTATCAGACATACCAGTATGGGTGAACTAAGTCCAACAGACAGTGGCTCCAAGTCCTCCAGGACAACTAGGATTAATCATTTTCCCTGTCCAATAATGAGTATTTGCATGCATGCAAAGAGTGGCAGAGTTATAGCAGTTGTGGGGCATATGGGTGTGGGCAGTGAA

>GL-13-K33_T7_F11

TTCACTGCCCACACCCATATGCCCCACAACTGCTATAACTCTGCCACTCTTTGCATGCATGCAAATACTCATTATTGGACAGGAAAAACGATTAATCCCAGTTGTCCTGGAGGACTTGGAGGACTCACTTCACTCATACCAGTATGTCTGATGGGGGTGGAGTTCAAGATCAGGCAACAGAAAAACACATAAAGGAAGTAATCTCCCAACTGACCTGGGTACATAGCACCCCTGGCCCCTACAAAGGACTAGATCTCTCAAAACTACATGAAACCCTCCATACCCATACTGGCCTGGTAAGCCTATTTAATACCACCCTGACTGGGCTCCATGAGGTCTCGGCCCAAAACCCTACTAACTGTTGGATGTGCCTCCCCCTGCACTTTAGGCCATACATTTCAATCCCTATACCTGAACAATGGAACAACTTCAGCACAGAAATAAACACCACTTCTGTTTTAGTAGGTCCTCTTTCCAATCTGGAAATAACCCATACCTCAAACCTCACCTGTGTAAAATTTAGCAATACTATAGACACAGCCAACTCCCAATGCATCAGGTGGGTAACTCCTCCCACACGAATAGTCTGCCTACCCTCAGGAATATTTTTTGTCTGTGGTACCTC

>GL-13-K36_T7_G11

GAGGTACCACAGACAAAAAATATTCCTGAGGGTAGGCAGACTATTCGTGGGGGGGAGTTACCCACCTGATGCATTGGGAGTTGGTTGTGTCTATCGTATTGCTAAATTTTACACAGGTGAGGTTTGAGGTATGGGTTATTTCCAGATTGGAAACAAGAGGTCCTACTAAAACGGAAGTGGTGTTTATGTGTTGAAGTTGTTCCCTTGTTCAGGTACAGGGATTGAAATGTATGGCCTTAAGTGCAGAGGGAGGCACATCCAACAGTTAGTAGGGTTTTGGGCCGAGACCTCATGAAACCCAGTGAGGGTGGTATTAAATAGGCTTACCAGGCGAGTATGGGTACGGAGGGTTTCATGTAGTTTTGAGAGATCTAGTCCTTTGTAGGGGCTAGGCGTGCTATGTACTCAGGTCAGTTGGGAGATTACTTCCTTTACGTGTTTTTCTCTTGCCTGATCTTGAACTCCACCCCTATCAGACATACCAGTATGGGTGAACTAAGTCCAACAGACAGTGGCTCCAAGTCCTCCAGGACAACTAGGATTAATCATTTTCCCTGTCCAATAATGAGTATTTGCATGCATGCAAAGAGTGGCAGAGTTATAGCAGTTGTGGGGCATATGGGTGTGGGCAGTGAA

>GL-13-K37_T7_H11

CACTGCCCACACCCATATGCCCCACAACTGCTATAACTCTGCCACTCTTTGCATGCATGCAAATACTCATTATTGGACAGGGAAAATGATTAATCCTAGTTGTCCTGGAAGACTTGGAGCCACTGTCTGTCGGACTTACTTCACCCATACTGGTATGTCTGAGGGGGGTGGAGTTCAAGATCAGGCAAGAGAAAAACATGTAAAGGAAGTAACCTCCCAACTGACCCGGGTACATAGCACCCCTAGCCCCTACAAAGGACTAGATCTCTTAAAACTACATGAAACCCTCCATACCCATACTTGCCTGGTAAGCCTATTTAATACCACCCTCACTGGGCTCCATGAGGTCTCGGCCCAAAACCCTACTAACTGTTGGATGTGCCTCCCCCTGTATTTCAGGCCATGCATTTCAATCCCTGTACCTGAACAATGGAACAACTACAGCACAGAAATAAACACCACTTCCGTTTTAGTAGGACCTCTTGTTTCCAATCTGGAAATAACCCATACCTCAAACCTCACCTGTGTAAAATTTAGCAATACTGTAGACACAACCAACTCCCAATGCATCAGGTGGGTAACTCCTCCCACACGAATAGTCTGCCTACCCTCAGGAATATTTTTTGTCTGTGGTACCTC

>GL-13-K38_T7_A12

GAGGTACCACAGACAAAAAATATTCCTGAGGGTAGGCAGACTATTCGTGGGGGGGAGTTACCCACCTGATGCATTGGGAGTTGGTTGTGTCTATCGTATTGCTAAATTTTACACAGGTGAGGTTTGAGGTATGGGTTATTTCCAGATTGGAAACAAGAGGTCCTACTAAAACGGAAGTGGTGTTTATGTGTTGAAGTTGTTCCCTTGTTCAGGTACAGGGATTGAAATGTATGGCCTTAAGTGCAGAGGGAGGCACATCCAACAGTTAGTAGGGTTTTGGGCCGAGACCTCATGAAACCCAGTGAGGGTGGTATTAAATAGGCTTACCAGGCGAGTATGGGTACGGAGGGTTTCATGTAGTTTTGAGAGATCTAGTCCTTTGTAGGGGCTAGGCGTGCTATGTACTCAGGTCAGTTGGGAGATTACTTCCTTTACGTGTTTTTCTCTTGCCTGATCTTGAACTCCACCCCTATCAGACATACCAGTATGGGTGAACTAAGTCCAACAGACAGTGGCTCCAAGTCCTCCAGGACAACTAGGATTAATCATTTTCCCTGTCCAATAATGAGTATTTGCATGCATGCAAAGAGTGGCAGAGTTATAGCAGTTGTGGGGCATATGGGTGTGGGCAGTGAA

>GL-13-K39_T7_B12

TTCACTGCCCACACCCATATGCCCCACAACTGCTATAACTCTGCCACTCTTTGCATGCATGCAAATACTCATTATTGGACAGGGAAAATGATTAATCCTAGTTGTCCTGGAGGGCTTGGAGCCACTGTCTGTTGGACTTAGTTCACCCATACTGGTATGTCTGATAGGGGTGGAGTTCAAGATCAGGCAAGAGAAAAACACGTAAAGGAAGTAATCTCCCAACTGACCTGAGTACATAGCACGCCTAGCCCCTACAAAGGACTAGATCTCTCAAAACTACATGAAACCCTCCGTACCCATACTCGCCTGGTAAGCCTATTTAATACCACCCTCACTGGGTTTCATGAGGTCTCGGCCCAAAACCCTACTAACTGTTGGATGTGCCTCCCTCTGCACTTAAGGCCATACATTTCAATCCCTGTACCTGAACAAGGGAACAACTTCAACACATAAACACCACTTCCGTTTTAGTAGGACCTCTTGTTTCCAATCTGGAAATAACCCATACCTCAAACCTCACCTGTGTAAAATTTAGCAATACGATAGACACAACCAACTCCCAATGCATCAGGTGGGTAACTCCCCCCCACGAATAGTCTGCCTACCCTCAGGAATATTTTTTGTCTGTGGTACCTC

>GL-13-K40_T7_C12

TTCACTGCCCACACCCATATGCCCCACAACTGCTATAACTCTGCCACTCTTTGCATGCATGCAAATACTCATTATTGGACAGGGAAAATGATTAATCCTAGTTGTCCTGGAAGACTTGGAGCCACTGTCTGTCGGACTTACTTCACCCATACTGGTATGTCTGAGGGGGGTGGAGTTCAAGATCAGGCAAGAGAAAAACATGTAAAGGAAGTAACCTCCCAACTGACCCGGGTACATAGCACCCCTAGCCCCTACAAAGGACTAGATCTCTTAAAACTACATGAAACCCTCCATACCCATACTTGCCTGGTAAGCCTATTTAATACCACCCTCACTGGGCTCCATGAGGTCTCGGCCCAAAACCCTACTAACTGTTGGATGTGCCTCCCCCTGTATTTCAGGCCATGCATTTCAATCCCTGTACCTGAACAATGGAACAACTACAGCACAGAAATAAACACCACTTCCGTTTTAGTAGGACCTCTTGTTTCCAATCTGGAAATAACCCATACCTCAAACCTCACCTGTGTAAAATTTAGCAATACTGTAGACACAACCAACTCCCAATGCATCAGGTGGGTAACTCCTCCCACACAAATAGTCTGCCTACCCTCACGAATATTTTGTGTCTGTGGTACCTC

>GL-13-K42_T7_D12

GAGGTACCACAGACAAAAAATATTCCTGAGGGTAGGCAGACTATTCGTGGGGGGGAGTTACCCACCTGATGCATTGGGAGTTGGTTGTGTCTATCGTATTGCTAAATTTTACACAGGTGAGGTTTGAGGTATGGGTTATTTCCAGATTGGAAACAAGAGGTCCTACTAAAACGGAAGTGGTGTTTATGTGTTGAAGTTGTTCCCTTGTTCAGGTACAGGGATTGAAATGTATGGCCTTAAGTGCAGAGGGAGGCACATCCAACAGTTAGTAGGGTTTTGGGCCGAGACCTCATGAAACCCAGTGAGGGTGGTATTAAATAGGCTTACCAGGCGAGTATGGGTACGGAGGGTTTCATGTAGTTTTGAGAGATCTAGTCCTTTGTAGGGGCTAGGCGTGCTATGTACTCAGGTCAGTTGGGAGATTACTTCCTTTACGTGTTTTTCTCTTGCCTGATCTTGAACTCCACCCCTATCAGACATACCAGTATGGGTGAACTAAGTCCAACAGACAGTGGCTCCAAGTCCTCCTGGACTACTAGGATTAATCATTTTCCCTG

>GL-13-K43_T7_E12

GAGGTACCACAGACAAAAAATATTCCTGAGGGTAGGCAGACTATTCGTGTGGGAGGAGTTACCCACCTGATGCATTGGGAGTTGGTTGTGTCTACAGTATTGCTAAATTTTACACAGGTGAGGTTTGAGGTATGGGTTATTTCCAGATTGGAAACAAGAGGTCCTACTAAAACGGAAGTGGTGTTTATTTCTGTGCTGTAGTTGTTCCATTGTTCAGGTACAGGGATTGAAATGCATGGCCTGAAATACAGGGGGAGGCACATCCAACAGTTAGTAGGGTTTTGGGCCGAGACCTCATGGAGCCCAGTGAGGGTGGTATTAAATAGGCTTACCAGGCAAGTATGGGTATGGAGGGTTTCATGTAGTTTTGAGAGATCTAGTCCTTTGTAGGGGCTAGGCGTGCTATGTACTCAGGTCAGTTGGGAGATTACTTCCTTTACGTGTTTTTCTCTTGCCTGATCTTGAACTCCACCCCTATCAGACATACCAGTATGGGTGAACTAAGTCCAACAGACAGTGGCTCCAAGTCCTCCAGGACAACTAGGATTAATCATTTTCCCTGTCCAATAATGAGTATTTGCATGCATGCAAAGAGTGGCAGAGTTATAGCAGTTGTGGGGCATATGGGTGTGGGCAGTGAA

>GL-13-K46_T7_F12

GAGGTACCACAGACAAAAAATATTCCTGAGGGTAGGCAGACTATTCGTGGGGGGGAGTTACCCACCTGATGCATTGGGAGTTGGTTGTGTCTATCGTATTGCCAAATTTTACACAGGTGAGGTTTGAGGTATGGGTTATTTCCAGATTGGAAACAAGAGGTCCTACTAAAACGGAAGTGGTGTTTATGTGTTGAAGTTGTTCCCTTGTTCAGGTACAGGGATTGAAATGTATGGCCTTAAGTGCAGAGGGAGGCACATCCAACAGTTAGTAGGGTTTTGGGCCGAGACCTCATGAAACCCAGTGAGGGTGGTATTAAATAGGCTTACCAGGCGAGTATGGGTACGGAGGGTTTCATGTAGTTTTGAGAGATCTAGTCCTTTGTAGGGGCTAGGCGTGCTATGTACTCAGGTCAGTTGGGAGATTACTTCCTTTACGTGTTTTTCTCTTGCCTGATCTTGAACTCCACCCCTATCAGACATACCAGTATGGGTGAACTAAGTCCAACAGACAGTGGCTCCAAGTCCTCCAGGACAACTAGGATTAATCATTTTCCCTGTCCAATAATGAGTATTTGCATGCATGCAAAGAGTGGCAGAGTTATAGCAGTTGTGGGGCATATGGGTGTGGGCAGTGAA

>GL-13-K47_T7_G12

GAGGTACCACAGACAAAAAATATTCCTGAGGGTAGGCAGACTATTCGTGGGGGGGAGTTACCCACCTGATGCATTGGGAGTTGGTTGTGTCTATCGTATTGCTAAATTTTACACAGGTGAGGTTTGAGGTATGGGTTATTTCCAGATTGGAAACAAGAGGTCCTACTAAAACGGAAGTGGTGTTTATGTGTTGAAGTTGTTCCCTTGTTCAGGTACAGGGATTGAAATGTATGGCCTTAAGTGCAGAGGGAGGCACATCCAACAGTTAGTAGGGTTTTGGGCCGAGACCTCATGGAGCCCAGTGAGGGTGGTATTAAATAGGCTTACCAGGCGAGTATGGGTACGGAGGGTTTCATGTAGTTTTGAGAGATCTAGTCCTTTGTAGGGGCTAGAGGTGCCATGTACCCGGGTGAGTTGGGAGATTACTTCTTTTACATGTTTTTCTCTTGCCTGATCTTGAACTCCACCCCCATCAGACATACCAGTTTGGGTGAAGTAAGTCCAACAGACAGTGACTCCAAGTCCTCCAGGACAACTAGGATTAATCATTTTTCCTGTCCAATAATGAGTATTTGCATGCATGCAAAGAGTGGCAGAGTGATAGCAGTTGCGGGGCATATGGGTGTGGGCAGTGAA

>GL-13-K48_T7_H12

GAGGTACCACAGACAAAAAATATTCCTGAGGGTAGGCAGACTATTCGTGGGGGGGAGTTACCCACCTGATGCATTGGGAGTTGGTTGTGTCTATCGTATTGCTAAATTTTACACAGGTGAGGTTTGAGGTATGGGTTATTTCCAGATTGGAAACAAGAGGTCCTACTAAAACGGAAGTGGTGTTTATGTGTTGAAGTTGTTCCCTTGTTCAGGTACAGGGATTGAAATGTATGGCCTTAAGTGCAGAGGGAGGCACATCCAACAGTTAGTAGGGTTTTGGGCCGAGACCTCATGAAACCCAGTGAGGGTGGTATTAAATAGGCTTACCAGGCGAGTATGGGTACGGAGGGTTTCATGTAGTTTTGAGAGATCTAGTCCTTTGTAGGGGCTAGGCGTGCTATGTACTCAGGTCAGTTGGGAGATTACTTCCTTTACGTGTTTTTCTCTTGCCTGATCTTGAACTCCACCCCCCTCAGACATACCAGTATGGGTGAAGTAAGTCCGACAGACAGTGGCTCCAAGTCTTCCAGGACAACTAGGATTAATCATTTTCCCTGTCCAATAATGAGTATTTGCATGCATGCAAAGAGTGGCAGAGTTATAGCAGTTGTGGGGCATATGGGTGTGGGCAGTGAA

>GL-13-K50_A03

GAGGTACCACAGACAAAAAATATTCCTGAGGGTAGGCAGACTATTCGTGGGGGGGAGTTACCCACCTGATGCATTGGGAGTTGGTTGTGTCTATCGTATTGCTAAATTTTACACAGGTGAGGTTTGAGGTATGGGTTATTTCCAGATTGGAAACAAGAGGTCCTACTAAAACGGAAGTGGTGTTTATGTGTTGAAGTTGTTCCCTTGTTCAGGTACAGGGATTGAAATGTATGGCCTTAAGTGCAGAGGGAGGCACATCCAACAGTTAGTAGGGTTTTGGGCCGAGACCTCATGAAACCCAGTGAGGGTGGTATTAAATAGGCTTACCAGGCGAGTATGGGTACGGAGGGTTTCATGTAGTTTTGAGAGATCTAGTCCTTTGTGGGGGCTAGGCGTGCTATGTACTCAGGTCAGTTGGGAGATTACTTCCTTTACGTGTTTTTCTCTTGCCTGATCTTGAACTCCACCCCTATCAGACATACCAGTATGGGTGAACTAAGTCCAACAGACAGTGGCTCCAAGTCCTCCAGGACAACTAGGATTAATCATTTTCCCTGTCCAATAATGAGTATTTGCATGCATGCAAAGAGTGGCAGAGTTATAGCAGTTGTGGGGCATATGGGTGTGGGCAGTGAA

>GL-13-K52_B03

TTCACTGCCCACACCCATATGCCCCGCAACTGCTATAACTCTGCCACTCTTTGCATGCATGCAAATACTCATTATTGGACAGGGAAAATGATTAATCCTAGTTGTCCTGGAGGACTTGGAGCCACTGTCTGTTGGACTTACTTCACCCATACCAGTATGTCTGATGGGGGTGGAATTCAAGGTCAGGCAAGAGAAAAACAAGTAAAGGAAGCAATCTCCCAACTGACTCGGGGACATAGCACCCCTAGCCCCTACAAAGGACTAGTTCTCTCAAAACTACATGAAACCCTCCGTACCCATACTCGCCTGGTGAGCCTATTTAATACTACCCTCACTCGGCTCCATGAGGTCTCAGCCCAAAACCCTACTAACTGTTGGATGTGCCTCCCCCTGCACTTCAGGCCATACATTTCAATCCCTGTTCCTGAACAATGGAACAACTTCAGCACAGAAATAAACACCACTTCCGTTTTAGTAGGACCTCTTGTTTCCAATCTGGAAATAACCCATACCTCAAACCTCACCTGTGTAAAATTTAGCAATACTATAGACACAACCAGCTCCCAATGCATCAGGTGGGTAACACCTCCCACACGAATAGTCTGCCTACCCTTAGGAATATTTTTTGTCTGTGGTACCTC

>GL-13-K53_C03

GAGGTACCACAGACAAAAAATATTCCTGAGGGTAGGCAGACTATTCGTGGGGGGGAGTTACCCACCTGATGCATTGGGAGTTGGTTGTGTCTATCGTATTGCTAAATTTTACACAGGTGAGGTTTGAGGTATGGGTTATTTCCAGATTGGAAACAAGAGGTCCTACTAAAACGGAAGTGGTGTTTATGTGTTGAAGTTGTTCCCTTGTTCAGGTACAGGGATTGAAATGTATGGCCTTAAGTGCAGAGGGAGGCACATCCAACAGTTAGTAGGGTTTTGGGCCGAGACCTCATGAAACCCAGTGAGGGTGGTATTAAATAGGCTTACCAGGCGAGTATGGGTACGGAGGGTTTCATGTAGTTTTGAGAGATCTAGTCCTTTGTAGGGGCTAGGCGTGCTATGTACTCAGGTCAGTTGGGAGATTACTTCCTTTACGTGTTTTTCTCTTGCCTGATCTTGAACTCCACCCCTATCAGACATACCAGTATGGGTGAACTAAGTCCAACAGACAGTGGCTCCAAGTCCTCCAGGACAACTAGGATTAATCATTTTCCCTGTCCAATAATGAGTATTTGCATGCATGCAAAGAGTGGCAGAGTTATAGCAGTTGTGGGGCATATGGGTGTGGGCAGTGAA

>GL-13-K54_D03

GAGGTACCACAGACAAAAAATATTCCTGAGGGTAGGCAGACTATTCGTGGGGGGGAGTTACCCACCTGATGCATTGGGAGTTGGTTGTGTCTATCGTATTGCTAAATTTTACACAGGTGAGGTTTGAGGTATGGGTTATTTCCAGATTGGAAACAAGAGGTCCTACTAAAACGGAAGTGGTGTTTATGTGTTGAAGTTGTTCCCTTGTTCAGGTACAGGGATTGAAATGTATGGCCTTAAGTGCAGAGGGAGGCACATCCAACAGTTAGTAGGGTTTGGGCCGAGACCTCATGAAACCCAGTGAGGGTGTTTATTAAATATGCTTACCA

>GL-13-K55_E03

TTCACTGCCCACACCCATATGCCCCACAACTGCTATAACTCTGCCACTCTTTGCATGCATGCAAATACTCATTATTGGACAGGGAAAATGATTAATCCTAGTTGTCCTGGAGGACTTGGAGCCACTGTCTGTTGGACTTAGTTCACCCATACTGGTATGTCTGATAGGGGTGGAGTTCAAGATCAGGCAAGAGAAAAACACGTAAAGGAAGTAATCTCCCAACTGACCTGAGTACATAGCACGCCTAGCCCCTACAAAGGACTAGATCTCTCAAAACTACATGAAACCCTCCGTACCCATACTCGCCTGGTAAGCCTATTTAATACCACCCTCACTGGGTTTCATGAGGTCTCGGCCCAAAACCCTACTAACTGTTGGATGTGCCTCCCTCTGCACTTAAGGCCATACATTTCAATCCCTGTACCTGAACAAGGGAACAACTTCAACACATAAACACCACTTCCGTTTTAGTAGGACCTCTTGTTTCCAATCTGGAAATAACCCATACCTCAAACCTCACCTGTGTAAAATTTAGCAATACGATAGACACAACCAACTCCCAATGCATCAGGTGGGTAACTCCCCCCCACGAATAGTCTGCCTACCCTCAGGAATATTTTTTGTCTGTGGTACCTC

>GL-13-K56_F03

TTCACTGCCCACACCCATATGCCCCACGACTGCTATAACTCTGCCACTCTTTGCATGCATGCAAATACTCATTATTGGACAGGGAAAATGATTAATCCTAGTTGTCCTGGAAGACTTGGAGCCACTGTCTGTCGGACTTACTTCACCCATACTGGTATGTCTGAGGGGGGTGGAGTTCAAGATCAGGCAAGAGAAAAACATGTAAAGGAAGTAACCTCCCAACTGACCCGGGTACATAGCACCCCTAGCCCCTACAAAGGACTAGTTCTCTCAAAACTACATGAAACCCTCCGTACCCATACTCGCCTGGTGAGCCTATTTAATACCACCCTCACTCGGCTCCATGAGGTCTCAGCCCAAAACCCTACTAACTGTTGGATGTGCCTCCCCCTGCACTTCAGGCCATACATTTCAATCCCTGTTCCTGAACAATGGAACAACTTCAGCACAAAAATAAACACCACTTCCGTTTTAGTAGGACCTCTTGTTTCCAATCTGGAAATAACCCATACCTCAAACCTCACCTGTGTAAAATTTATCAATACTATAGACACAACCAGCTCCCAATGCATCAGGTGGGTAACACCTCCCACACGAATAGTCTGCCTACCCTCAGGAATATTTTTTGTCTGTGGTACCTC

>GL-13-K57_G03

GAGGTACCACAGACAAAAAATATTCCTGAGGGTAGGCAGACTATTCGTGGGGGGGAGTTACCCACCTGATGCATTGGGAGTTGGTTGTGTCTATCGTATTGCTAAATTTTACACAGGTGAGGTTTGAGGTATGGGTTATTTCCAGATTGGAAACAAGAGGTCCTACTAAAACGGAAGTGGTGTTTATGTGTTGAAGTTGTTCCCTTGTTCAGGTACAGGGATTGAAATGTATGGCCTTAAGTGCAGAGGGAGGCACATCCAACAGTTAGTAGGGTTTTGGGCCGAGACCTCATGAAACCCAGTGAGGGTGGTATTAAATAGGCTTACCAGGCGAGTATGGGTACGGAGGGTTTCATGTAGTTTTGAGAGATCTATTCCTTTGTACGGGCTAGGCGTGCTA

>GL-13-K59_H03

TTCACTGCCCACACCCATATGCCCCGCAACTGCTATCACTCTGCCACTCTTTGCATGCATGCAAATACTCATTATTGGACAGGAAAGATGATTAATCCTAGTTGTCCTGGAGGACTTGGAGTCACTGTCTGTTGGACTTACTTCACCCAAACTGGTATGTCTGATGGGGGTGGAGTTCAAGATCAGGCAAGAGAAAAACATGTAAAAGAAGTAATCTCCCAACTCACCCGGGTACATGGCACCTCTAGCCCCTACAAAGGACTAGATCTCTCAAAACTACATGAAACCCTCCGTACCCATACTCGCCTGGTAAGCCTATTTAATACCACCCTCACTGGGCTCCATGAGGTCTCGGCCCAAAACCCTACTAACTGTTGGATATGCCTCCCCCTGAACTTCAGGCCATATGTTTCAATCCCTGTACCTGAACAATGGAACAACTTCAGCACAGAAATAAACACCACTTCCGTTTTAGTAGGACCTCTTGTTTCCAATCTGGAAATAACCCATACCTCAAACCTCACCTGTGTAAAATTTAGCAATACTACATACACAACCAACTCCCAATGCATCAGGTGGTAACTCCTCCCACACAAATAGTCTGCCTACCCTCAGGAATATTTTTTGTCTGTGGTACCTC

**KO-III**

>GL-15-K1_A01

GAGGTACCACAGACAAAAAATATTCCTGAGGGTAGGCAGACTATTCGTGGGGGGGAGTTACCCACCTGATGCATTGGGAGTTGGTTGTGTCTATCGTATTGCTAAATTTTACACAGGTGAGGTTTGAGGTATGGGTTATTTCCAGATTGGAAACAAGAGGTCCTACTAAAACGGAAGTGGTGTTTATGTGTTGAAGTTGTTCCCTTGTTCAGGTACAGGGATTGAAATGTATGGCCTTAAGTGCAGGGGGAGGCACATCCAACAGTTAGTAGGGTTTTGGACCGAGACCTCATGAAACCCAGTGAGGGTGGTATTAAATAGGCTTACCAGGCAAGTATGGGTATGGAGGGTTTCATGTAGTTTTAGGAGATCTAGTCCTTTGTAGGGGCTAGGGGTGCTATGTACCCGGGTCAGTTGGGAGATTACTTCCTTTACATGTTTTTCTCTTGCCTGATCTTGAACTCCACCCCCCTCAGACATACCAGTATGGGTGAAGTAAGTCCGACAGACAGTGGCTCCAAGTCTTCCAGGACAACTAGGATTAATCATTTTCCCTGTCCAATAATGAGTATTTGCATGCATGCAAAGAGTGGCAGAGTTATAGCAGTTGTGGGGCATATGGGTGTGGGCAGTGAA

>GL-15-K2_B01

GAGGTACCACAGACAAAAAATATTCCTGAGGGTAGGCAGACTATTCGTGGGGGGGAGTTACCCACCTGATGCATTGGGAGTTGGTTGTGTCTATCGTATTGCTAAATTTTACACAGGTGAGGTTTGAGGTATGGGTTATTTCCAGATTGGAAACAAGAGGTCCTACTAAAACGGAAGTGGTGTTTATGTGTTGAAGTTGTTCCCTTGTTCAGGTACAGGGATTGAAATGTATGGCCTTAAGTGCAGAGGGAGGCACATCCAACAGTTAGTAGGGTTTTGGGCCGAGACCTCATGAAACCCAGTGAGGGTGGTATTAAATAGGCTTACCAGGCGAGTATGGGTACGGAGGGTTTCATGTAGTTTTGAGAGATCTAGTCCTTTGTAGGGGCTAGGCGTGCTATGTACTCAGGTCAGTTGGGAGATTACTTCCTTTACGTGTTTTTCTCTTGCCTGATCTTGAACTCCACCCCTATCAGACATACCAGTATGGGTGAAGTAAGTCCAACAGACAGTGGCTCCAAGTCCTCCAGGACAACTAGGATTAATCATTTTCCCTGTCCAATAATGAGTATTTGCATGCATGCAAAGAGTGGCAGAGTTATAGCAGTTGTGGGGCATATGGGTGTGGGCAGTGAA

>GL-15-K3_C01

GAGGTACCACAGACAAAAAATATTCCTGAGGGTAGGCAGACTATTCGTGGGGGGGAGTTACCCACCTGATGCATTGGGAGTTGGTTGTGTCTATCGTATTGCTAAATTTTACACAGGTGAGGTTTGAGGTATGGGTTATTTCCAGATTGGAAACAAGAGGTCCTACTAAAACGGAAGTGGTGTTTATGTGTTGAAGTTGTTCCCTTGTTCAGGTACAGGGATTGAAATGTATGGCCTTAAGTGCAGAGGGAGGCACATCCAACAGTTAGTAGGGTTTTGGGCCGAGACCTCATGAAACCCAGTGAGGGTGGTATTAAATAGGCTTACCAGGCGAGTATGGGTACGGAGGGTTTCATGTAGTTTTGAGAGATCTAGTCCTTTGTAGGGGCTAGGCGTGCTATGTACTCAGGTCAGTTGGGAGATTACTTCCTTTACGTGTTTTTCTCTTGCCTGATCTTGAACTCCACCCCTATCAGACATACCAGTATGGGTGAACTAAGTCCAACAGACAGTGGCTCCAAGTCCTCCAGGACAACTAGGATTAATCATTTTCCCTGTCCAATAATGAGTATTTGCATGCATGCAAAGAGTGGCAGAGCTATAGCAGTTGTGGGGCATATGGGTGTGGGCAGTGAA

>GL-15-K9_D01

TTCACTGCCCACACCCATATGCCCCGCAACTGCTATCACTCTGCCACTCTTTGCATGCATGCAAATACTCATTATTGGACAGGAAAAATGATTAATCCTAGTTGTCCTGGAGGACTTGGAGTCACTGTCTGTTGGACTTACTTCACCCAAACTGGTATGTCTGATGGGGGTGGAGTTCAAGATCAGGCAAGAGAAAAACATGTAAAAGAAGTAATCTCCCAACTCACCCGGGTACATGGCACCTCTAGCCCCTACAAAGGACTAGATCTCTCAAAACTACATGAAACCCTCCGTACCCATACTCGCCTGGTAAGCCTATTTAATACCACCCTCACTGGGCTCCATGAGGTCTCGGCCCAAAACCCTACTAACTGTTGGATATGCCTCCCCCTGAACTTCAGGCCATATGTTTCAATCCCTGTACCTGAACAATGGAACAACTTCAGCACAGAAATAAACACCACTTCCGTTTTAGTAGGACCTCTTGTTTCCAATCTGGAAATAACCCATACCTCAAACCTCACCTGTGTAAAATTTAGCAATACTACATACACAACCAACTCCCAATGCATCAGGTGGGTAACTCCTCCCACACAAATAGTCTGCCTACCCTCAGGAATATTTTTTGTCTGTGGTACCTC

>GL-15-K11_E01

TTCACACAGGAAACAGCTATGACCATGATTACGCCAAGCTATTTAGGTGACACTATAGAATACTCAAGCTATGCATCCAACGCGTTGGGAGCTCTCCCATATGGTCGACCTGCAGGCGGCCGCACTAGTGATTTCACTGCCCACACCCATATGCCCCACAACTGCTATAACTCTGCCACTCTTTGCATGCATGCAAATACTCATTATTGGACAGGGAAAATGATTAATCCTAGTTGTCCTGGAGGGCTTGGAGCCACTGTCTGTTGGACTTAGTTCACCCATACTGGTATGTCTGATAGGGGTGGAGTTCAAGATCAGGCAAGAGAAAAACACGTAAAGGAAGTAATCTCCCAACTGACCTGAGTACATAGCACGCCTAGCCCCTACAAAGGACTAGATCTCTCAAAACTACATGAAACCCTCCGTACCCATACTCGCCTGGTAAGCCTATTTAATACCACCCTCACTGGGTTTCATGAGGTCTCGGCCCAAAACCCTACTAACTGTTGGATGTGCCTCCCTCTGCACTTAAGGCCATACATTTCAATCCCTGTACCTGAACAAGGGAACAACTTCAACACATAAACACCACTTCCGTTTTAGTAGGACCTCTTGTTTCCAATCTGGAAATAACCCATACCTCAAACCTCACCTGTGTAAAATTTAGCAATACGATAGACACAACCAACTCCCAATGCATCAGGTGGGTAACTCCCCCCCACGAATAGTCTGCCTACCCTCAGGAATATTTTTTGTCTGTGGTACCTC

>GL-15-K21_F01

GAGGTACCACAGACAAAAAATATTCCTGAGGGTAGGCAGACTATTCGTGGGGGGGAGTTACCCACCTGATGCATTGGGAGTTGGTTGTGTCTATCGTATTGCTAAATTTTACACAGGTGAGGTTTGAGGTATGGGTTATTTCCAGATTGGAAACAAGAGGTCCTACTAAAACGGAAGTGGTGTTTATGTGTTGAAGTTGTTCCCTTGTTCAGGTACAGGCATTGAAATGTATGGCCTTAAGTGCAGAGGGAGGCACATCCAACAGTTAGTAGGGTTTTGGGCTGAGACCTCATGGAGCCGAGTGAGGGTGGTATTAAATAGGCTCACCAGGCGAGTATGGGTACGGAGGGTTTCATGTAGTTTTGAGAGAACTAGTCCTTTGTAGGGGCTAGGGGTGCTATGTCCCCGGGTCAGTTGGGAGATTGCTTCCTTTACTTGTTTTTCTCTTGCCTGATCTTGAACTCCACCCCCCTCAGACATACCAGTATGGGTGAAGTAAGTCCGACAGACAGTGGCTCCAAGTCTTCCAGGACAACTAGGATTAATCATTTTCCCTGTCCAATAATGAGTATTTGCATGCATGCAAAGAGTGGCAGAGTTATAGCAGTTGTGGGGCATATGGGTGTGGGCAGTGAA

>GL-15-K22_G01

TTCACTGCCCACACCCATATGCCCCACAACTGCTATAACTCTGCCACTCTTTGCATGCATGCAAATACTCATTATTGGACAGGGAAAATGATTAATCCTAGTTGTCCTGGAAGACTTGGAGCCACTGTCTGTCGGACTTACTTCACCCATACTGGTATGTCTGAGGGGGGTGGAGTTCAAGATCAGGCAAGAGAAAAACATGTAAAGGAAGTAATCTCCCAACTGACCCGGGTACATAGCACCCCTAGCCCCTACAAAGGACTAGATCTCCTAAAACTACATGAAACCCTCCATACCCATACTCGCCTGGTAAGCCTATTTAATACCACCCTCACTGGGCTCCATGAGGTCTCGGCCCAAAACCCTACTAACTGTTGGATGTGCCTCCCCCTGTATTTCAGGCCATGCATTTCAATCCCTGTACCTGAACAATGGAACAACTACAGCACAGAAATAAACACCACTTCCGTTTTAGTAGGACCTCTTGTTTCCAATCTGGAAATAACCCATACCTCAAACCTCACCTGTGTAAAATTTAGCAATACTGTAGACACAACCAACTCCCAATGCATCAGGTGGGTAACTCCTCCCACACGAATAGTCTGCCTACCCTCAGGAATATTTTTTGTCTGTGGTACCTC

>GL-15-K41_H01

GAGGTACCACAGACAAAAAATATTCTGAAGGAGGGCAGACTATTCGTGTGGGAGGTGTTACCCACCTGAAGCATTGGGAGCTGGTTGTGTCTATAGTATTGCTAAATTTTACACAGGTGAGGTTTGAGGTATGGGTTATTTCCAGATTGGAAACAAGAGGTCCTACTAAAACGGAAGTGGTGTTTATTTCTGTGCTGAAGTTGTTCCATTGTTCAGGAACAGGGATTGAAATGTATGGCCTGAAGTGCAGGGGGAGGCACATCCAACAGTTAGTAGGGTTTTGGGCTGAGACCTCATGGAGCCGAGTGAGGGTGGTATTAAATAGGCTCACCAGGCGAGTATGGGTACGGAGGGTTTCATGTAGTTTTGAGAGAACTAGTCCTTTGTAGGGGCTAGGGGTGCTATGTCCCCGGGTCAGTTGGGAGATTGCTTCCTTTACTTGTTTTTCTCTTGCCTGACCTTGAATTCCACCCCCATCAGACATACTGGTATGGGTGAAGTAAGTCCAACAGACAGTGGCTCCAAGTCCTCCAGGACAACTAGGATTAATCATTTTCCCTGTCCAATAATGAGTATTTGCATGCATGCAAAGAGTGGCAGAGTTATAGCAGTTTGCGGGGCATATGGGTGTGGGCAGTGAA

>GL-15-K42_A02

TTCACTGCCCACACCCATATGCCCCGCAACTGCTATCACTCTGCCACTCTTTGCATGCATGCAAATACTCATTATTGGACAGGAAAAATGATTAATCCTAGTTGTCCTGGAGGACTTGGAGTCACTGTCTGTTGGACTTACTTCACCCAAACTGGTATGTCTGATGGGGGTGGAGTTCAAGATCAGGCAAGAGAAAAACATGTAAAAGAAGTAATCTCCCAACTCACCCGGGTACATGGCACCTCTAGCCCCTACAAAGGACTAGATCTCTCAAAACTACATGAAACCCTCCGTACCCATACTCGCCTGGTAAGCCTATTTAATACCACCCTCACTGGGCTCCATGAGGTCTCGGCCCAAAACCCTACTAACTGTTGGATATGCCTCCCCCTGAACTTCAGGCCATATGTTTCAATCCCTGTACCTGAACAATGGAACAACTTCAGCACAGAAATAAACACCACTTCCGTTTTAGTAGGACCTCTTGTTTCCAATCTGGAAATAACCCATACCTCAAACCTCACCTGTGTAAAATTTAGCAATACTACATACACAACCAACTCCCAATGCATCAGGTGGGTAACTCCTCCCACACAAATAGTCTGCCTACCCTCAGGAATATTTTTTGTCTGTGGTACCTC

>GL-15-K43_B02

GAGGTACCACAGACAAAAAATATTCCTGAGGGTAGGCAGACTATTCGTGTGGGAGGTGTTACCCACCTGATGCATTGGGAGCTGGTTGTGTCTATAGTATTGCTAAATTTTACACAGGTGAGGTTTGAGGTATGGGTTATTTCCAGATTGGAAACAAGAGGTCCTACTAAAACGGAAGTGGTGTTTATTTCTGTGCTGAAGTTGTTCCATTGTTCAGGAACAGGGATTGAAATGTATGGCCTGAAGTGCAGGGGGAGGCACATCCAACAGTTAGTAGGGTTTTGGGCCGAGACCTCATGGAGCCCAGTGAGGGTGGTATTAAATAGGCTTACCAGGCGAGTATGGGTACGGAGGGTTTCATGTAGTTTTGAGAGATCTAGTCCTTTGTAGGGGCTAGAGGTGCCATGTACCCGGGTGAGTTGGGAGATTACTTCTTTTACATGTTTTTCTCTTGCCTGATCTTGAACTCCACCCCCATCAGACATACCAGTTTGGGTGAAGTAAGTCCAACAGACAGTGACTCCAAGTCCTCCAGGACAACTAGGATTAATCATTTTTCCTGTCCAATAATGAGTATTTGCATGCATGCAAAGAGTGGCAGAGTGATAGCAGTTGCGGGGCATATGGGTGTGGGCAGTGAA

>GL-15-K51_C02

GAGGTACCACAGACAAAAAATATTCCTGAGGGTAGGCAGACTATTCGTGTGGGAGGTGTTACCCACCTGATGCATTGGGAGCTGGTTGTGTCTATAGTATTGCTAAATTTTACACAGGTGAGGTTTGAGGTATGGGTTATTTCCAGATTGGAAACAAGAGGTCCTACTAAAACGGAAGTGGTGTTTATGTGTTGAAGTTGTTCCCTTGTTCAGGTACAGGGATTGAAATGTATGGCCTTAAGTGCAGAGGGAGGCACATCCAACAGTTAGTAGGGTTTTGGGCCGAGACCTCATGAAACCCAGTGAGGGTGGTATTAAATAGGCTTACCAGGCGAGTATGGGTACGGAGGGTTTCATGTAGTTTTGAGAGATCTAGTCCTTTGTAGGGGCTAGGCGTGCTATGTACTCAGGTCAGTTGGGAGATTACTTCCTTTACGTGTTTTTCTCTTGCCTGATCTTGAACTCCACCCCTATCAGACATACCAGTATGGGTGAACTAAGTCCAACAGACAGTGGCTCCAAGCCCTCCAGGACAACTAGGATTAATCATTTTCCCTGTCCAATAATGAGTATTTGCATGCATGCAAAGAGTGGCAGAGTTATAGCAGTTGTGGGGCATATGGGTGTGGGCAGTGAA

>GL-15-K52_D02

TTCACTGCCCACACCCATATGCCCCGCAACTGCTATCACTCTGCCACTCTTTGCATGCATGCAAATACTCATTATTGGACAGGAAAAATGATTAATCCTAGTTGTCCTGGAGGACTTGGAGTCACTGTCTGTTGGACTTACTTCACCCAAACTGGTATGTCTGATGGGGGTGGAGTTCAAGATCAGGCAAGAGAAAAACATGTAAAAGAAGTAATCTCCCAACTCACCCGGGTACATGGCACCTCTAGCCCCTACAAAGGACTAGATCTCTCAAAACTACATGAAACCCTCCGTACCCATACTCGCCTGGTAAGCCTATTTAATACCACCCTCACTGGGCTCCATGAGGTCTCGGCCCAAAACCCTACTAACTGTTGGATATGCCTCCCCCTGAACTTCAGGCCATATGTTTCAATCCCTGTACCTGAACAATGGAACAACTTCAGCACAGAAATAAACACCACTTCCGTTTTAGTAGGACCTCTTGTTTCCAATCTGGAAATAACCCATACCTCAAACCTCACCTGTGTAAAATTTAGCAATACTACATACACAACCAACTCCCAATGCATCAGGTGGGTAACTCCTCCCACACAAATAGTCTGCCTACCCTCAGGAATATTTTTTGTCTGTGGTACCTC

>GL-15-K53_E02

GAGGTACCACAGACAAAAAATATTCCTGAGGGTAGGCAGACTATTCGTGGGGGGGAGTTACCCACCTGATGCATTGGGAGTTGGTTGTGTCTATCGTATTGCTAAATTTTACACAGGTGAGGTTTGAGGTATGGGTTATTTCCAGATTGGAAACAAGAGGTCCTACTAAAACGGAAGTGGTGTTTATGTGTTGAAGTTGTTCCCTTGTTCAGGTACAGGGATTGAAATGTATGGCCTTAAGTGCAGAGGGAGGCACATCCAACAGTTAGTAGGGTTTTGGGCCGAGACCTCATGAAACCCAGTGAGGGTGGTATTAAATAGGCTTACCAGGCGAGTATGGGTACGGAGGGTTTCATGTAGTTTTGAGAGATCTAGTCCTTTGTAGGGGCTAGGCGTGCTATGTACTCAGGTCAGTTGGGAGATTACTTCCTTTACGTGTTTTTCTCTTGCCTGATCTTGAACTCCACCCCTATCAGACATACCAGTATGGGTGAACTAAGTCCAACAGACAGTGGCTCCAAGTCCTCCAGGACAACTAGGATTAATCATTTTCCCTGTCCAATAATGAGTATTTGCATGCATGCAAAGAGTGGCAGAGTTATAGCAGTTGTGGGGCATATGGGTGTGGGCAGTGAA

>GL-15-K55_F02

GAGGTACCACAGACAAAAAATATTCCTGAGGGTAGGCAGACTATTCGTGTGGGAGGAGTTACCCACCTGATGCATTGGGAGTTGGTTGTGTCTACAGTATTGCTAAATTTTACACAGGTGAGGTTTGAGGTATGGGTTATTTCCAGATTGGAAACAAGAGGTCCTACTAAAACGGAAGTGGTGTTTATTTCTGTGCTGTAGTTGTTCCATTGTCCAGGTACAGGGATTGAAATGCATGGCCTGAAATACAGGGGGAGGCACATCCAACAGTTAGTAGGGTTTTGGGCCGAGACCTCATGGAGCCCAGTGAGGGTGGTATTAAATAGGCTTACCAGGCAAGTATGGGTATGGAGGGTTTCATGTAGTTTTAGGAGATCTAGTCCTTTGTAGGGGCTAGGGGTGCTATGTACCCGGGTCAGTTGGGAGATTACTTCCTTTACATGTTTTTCTCTTGCCTGATCTTGAACTCCACCCCCCTCAGACATACCAGTATGGGTGAAGTAAGTCCGACAGACAGTGGCTCCAAGTCTTCCAGGACAACTAGGATTAATCATTTTCCCTGTCCAATAATGAGTATTTGCATGCATGCAAAGAGTGGCAGAGTTATAGCAGTTGTGGGGCATATGGGTGTGGGCAGTGAA

>GL15.K56_G02

GAGGTACCACAGACAAAAAATATTCCTGAGGGTAGGCAGACTATTTGTGTGGGAGGAGTTACCCACCTGATGCATTGGGAGTTGGTTGTGTATGTAGTATTGCTAAATTTTACACAGGTGAGGTTTGAGGTATGGGTTATTTCCAGATTGGAAACAAGAGGTCCTACTAAAACGGAAGTGGTGTTTATTTCTGTGCTGAAGTTGTTCCATTGTTCAGGTACAGGGATTGAAACATATGGCCTGAAGTTCAGGGGGAGGCATATCCAACAGTTAGTAGGGTTTTGGGCCGAGACCTCATGGAGCCCAGTGAGGGTGGTATTAAATAGGCTTACCAGGCGAGTATGGGTACGGAGGGTTTCATGTAGTTTTGAGAGATCTAGTCCTTTGTAGGGGCTAGAGGTGCCATGTACCCGGGTGAGTTGGGAGATTACTTCTTTTACATGTTTTTCTCTTGCCTGATCTTGAACTCCACCCCCATCAGACATACCAGTTTGGGTGAAGTAAGTCCAACAGACAGTGACTCCAAGTCCTCCAGGACAACTAGGATTAATCATTTTTCCTGTCCAATAATGAGTATTTGCATGCATGCAAAGAGTGGCAGAGTGATAGCAGTTGCGGGGCATATGGGTGTGGGCAGTGAA

>GL-15-K58_H02

GATGTACCACAGACAAAAAATATTCCTGAGGGTAGGCAGACTATTCGTGGGGGGGAGTTACCCACCTGATGCATTGGGAGTTGGTTGTGTCTATCGTATTGCTAAATTTTACACAGGTGAGGTTTGAGGTATGGGTTATTTCCAGATTGGAAACAAGAGGTCCTACTAAAACGGAAGTGGTGTTTATGTGTTGAAGTTGTTCCCTTGTTCAGGTACAGGGATTGAAATGTATGGCCTTAAGTGCAGAGGGAGGCACATCCAACAGTTAGTAGGGTTTTGGGCCGAGACCTCATGAAACCCAGTGAGGGTGGTATTAAATAGGCTTACCAGGCGAGTATGGGTACGGAGGGTTTCATGTAGTTTTGAGAGATCTAGTCCTTTGTAGGGGCTAGGCGTGCTATGTACTCAGGTCAGTTGGGAGATTACTTCCTTTACGTGTTTTTCTCTTGCCTGATCTTGAACTCCACCCCTATCAGACATACCAGTATGGGTGAACTAAGTCCAACAGACAGTGGCTCCAAGTCCTCCAGGACAACTAGGATTAATCATTTTCCCTGTCCAATAATGAGTATTTGCATGCATGCAAAGAGTGGCAGAGTTATAGCAGTTGTGGGGCATATGGGTGTGGGCAGTGAA

>GL-15-K63_A03

TATTTCACTGCCCACACCCATATGCCCCGCAACTGCTATAACTCTGCCACTCTTTGCATGCATGCAAATACTCATTATTGGACAGGGAAAATGATTAATCCTAGTTGTCCTGGAAGACTTGGAGCCACTGTCTGTCGGACTTACTTCACCCATACTGGTATGTCTGAGGGGGGTGGAGTTCAAGATCAGGCAAGAGAAAAACATGTAAAGGAAGTAATCTCCCAACTGACCCGGGGACATAGCACCCCTAGCCCCTACAAAGGACTAGTTCTCTCAAAACTACATGAAACCCTCCGTACCCATACTCGCCTGGTGAGCCTATTTAATACCACCCTCACTCGGCTCCATGAGGTCTCAGCCCAAAACCCTACTAACTGTTGGATGTGCCTCCCCCTGCACTTCAGGCCATACATTTCAATCCCTGTTCCTGAACAATGGAACAACTTCAGCACAGAAATAAACACCACTTCCGTTTTAGTAGGACCTCTTGTTTCCAATCTGGAAATAACCCATACCTCAAACCTCACCTGTGTAAAATTTAGCAATACTATAGACACAACCAGCTCCCAATGCATCAGGTGGGTAACACCTCCCACACGAATAGTCTGCCTACCCTCAGGAATATTTTTTGTCTGTGGTACCTC

>GL-15-K64_B03

GAGGTACCACAGACAAAAAATATTCCTGAGGGTAGGCAGACTATTCGTGTGGGAGGAGTTACCCACCTGATGCATTGGGAGTTGGTTGTGTCTACAGTATTGCTAAATTTTGCACAGGTGAGGTTTGAGGTATGGGTTATTTCCAGATTGGAAACAAGAGGTCCTACTAAAACGGAAGTGGTGTTTATTTCTGTGCTGTAGTTGTTCCATTGTTCAGGTACAGGGATTGAAATGCATGGCCTGAAATACAGGGGGAGGCACATCCAACAGTTAGTAGGGTTTTGGGCCGAGACCTCATGGAGCCCAGTGAGGGTGGTATTAAATAGGCTTACCAGGCGAGTATGGGTACGGAGGGTTTCATGTAGTTTTGAGAGATCTAGTCCTTTGTAGGGGCTAGGCGTGCTATGTACTCAGGTCAGTTGGGAGATTACTTCCTTTACGTGTTTTTCTCTTGCCTGATCTTGAACTCCACCCCTATCAGACATACCAGTTTGGGTGAACTAAGTCCAACAGACAGTGGCTCCAAGTCCTCCAGGACAACTAGGATTAATCATTTTCCCTGTCCAATAATGAGTATTTGCATGCATGCAAAGAGTGGCAGAGTTATAGCAGTTGTGGGGCATATGGGTGTGGGCAGTGAA

>GL-35-K11_T7_G07

GAGGTACCACAGACAAAAAATATTCCTGAGGGTAGGCAGACTATTCGTGTGGGAGGAGTTACCCACCTGATGCATTGGGAGTTGGCTGTGTCTATAGTATTGCTAAATTTTACACAGGTGAGGTTTGAGGTATGGGTTATTTCCAGATTGGAAAGAGGACCTACTAAAACAGAAGTGGTGTTTATTTCTGTGCTGAAGTTGTTCCATTGTTCAGGTATAGGGATTGAAATGTATGGCCTAAAGTGCAGGGGGAGGCACATCCAACAGTTAGTAGGGTTTTGGGCCGAGACCTCATGGAGCCCAGTCAGGGTGGTATTAAATAGGCTTACCAGGCCAGTATGGGTATGGAGGGTTTCATGTAGTTTTGAGAGATCTAGTCCTTTGTAGGGGCTAGGGGTGCTATGTACCCGGGTCAGTTGGGAGGTTACTTCCTTTACATGTTCTTCTCTTGCCTGATCTTGAACTCCACCCCCCTCAGACATACCAGTATGGGTGAAGTAAGTCCGACAGACAGTGGCTCCAAGTCTTCCAGGACAACTAGGATTAATCATTTTCCCTGTCCAATAATGAGTATTTGCATGCATGCAAAGAGTGGCAGAGTTATAGCAGTTGTGGGGCATATGGGTGTGGGCAGTGAA

>GL-35-K1_T7_A07

GAGGTACCACAGACAAAAAATATTCCTGAGGGTAGGCAGACTATTCGTGTGGGAGGAGTTACCCACCTGATGCATTGGGAGTTGGTTGTGTCTACAGTATTGCTAAATTTTACACAGGTGAGGTTTGAGGTATGGGTTATTTCCAGATTGGAAACAAGAGGTCCTACTAAAACGGAAGTGGTGTTTATTTCTGTGCTGTAGTTGTTCCATTGTTCAGGTACAGGGATTGAAATGCATGGCCTGAAATACAGGGGGAGGCACATCCAACAGTTAGTAGGGTTTTGGGCCGAGACCTCATGGAGCCCAGTGAGGGTGGTATTAAATAGGCTTACCAGGCAAGTATGGGTATGGAGGGTTTCATGTAGTTTTAGGAGATCTAGTCCTTTGTAGGGGCTAGGGGTGCTATGTACCCGGGTCAGTTGGGAGATTACTTCTTTTACATGTTTTTCTCTTGCCTGATCTTGAACTCCACCCCTATCAGACATACCAGTATGGGTGAACTAAGTCCAACAGACAGTGGCTCCAAGTCCTCCAGGACAACTAGGATTAATCATTTTCCCTGTCCAATAATGAGTATTTGCATGCATGCAAAGAGTGGCAGAGTTATAGCAGTTGTGGGGCATATGGGTGTGGGCAGTGAA

>GL-35-K2_T7_B07

GAGGTACCACAGACAAAAAATATTCCTGAGGGTAGGCAGACTATTCGTGGGGGGGAGTTACCCACCTGATGCATTGGGAGTTGGTTGTGTCTATCGTATTGCTAAATTTTACACAGGTGAGGTTTGAGGTATGGGTTATTTCCAGATTGGAAACAAGAGGTCCTACTAAAACGGTAGTGGTGTTTATGTGTTGAAGTTGTTCCCTTGTTCAGGTACAGGGATTGAAATGTATAGCCTTAAGTGCAGAGGGAGGCACATCCAACAGTTAGTAGGGTTTTGGGCCGAGACCTCATGAAACCCAGTGAGGGTGGTATTAAATAGGCTTACCAGGCGAGTATGGGTACGGAGGGTTTCATGTAGTTTTGAGAGATCTAGTCCTTTGTAGGGGCTAGGCGTGCTATGTACTCAGGTCAGTTGGGAGATTACTTCCTTTACGTGTTTTTCTCTTGCCTGATCTTGAACTCCACCCCTATCAGACATACCAGTATGGGTGAACTAAGTCCAACAGACAGTGGCTCCAAGTCCTCCAGGACAACTAGGATTAATCATTTTCCCTGTCCAATAATGAGTATTTGCATGCATGCAAAGAGTGGCAGAGTTATAGCAGTTGTGGGGCATATGGGTGTGGGCAGTGAA

>GL-35-K3_T7_C07

GAGGTACCACAGACAAAAAATATTCCTGAGGGTAGGCAGACTATTCGTGTGGGAGGAGTTACCCACCTGATGCATTGGGAGTTGGCTGTGTCTATAGTATTGCTAAATTTTACACAGGTGAGGTTTGAGGTATGGGTTATTTCCAGATTGGAAAGAGGACCTACTAAAACAGAAGTGGTGTTTATTTCTGTGCTGAAGTTGTTCCATTGTTCAGGTATAGGGATTGAAATGTATGGCCTAAAGTGCAGGGGGAGGCACATCCAACAGTTAGTAGGGTTTTGGGCCGAGACCTCATGGAGCCCAGTCAGGGTGGTATTAAATAGGCTTACCAGGCCAGTATGGGTATGGAGGGTTTCATGTAGTTTTGAGAGATCTAGTCCTTTGTAGGGGCCAGGGGTGCTATGTACCCAGGTCAGTTGGGAGATTACTTCCTTTATGTGTTTTTCTGTTGCCTGATCTTGAACTCCACCCCCATCAGACATACTGGTATGAGTGAAGTGAGTCCTCCAAGTCCTCCAGGACAACTGGGATTAATCGTTTTTCCTGTCCAATAATGAGTATTTGCATGCATGCAAAGAGTGGCAGAGTTATAGCAGTTGTGGGGCATATGGGTGTGGGCAGTGAA

>GL-35-K4_T7_D07

GAGGTACCACAGACAAAAAATATTCCTGAGGGTAGGCAGACTATTCGTGGGGGGGAGTTACCCACCTGATGCATTGGGAGTTGGTTGTGTCTATCGTATTGCTAAATTTTACACAGGTGAGGTTTGAGGTATGGGTTATTTCCAGATTGGAAACAAGAGGTCCTACTAAAACGGAAGTGGTGTTTATGTGTTGAAGTTGTTCCCTTGTTCAGGTACAGGGATTGAAATGTATGGTCTTAAGTGCAGAGGGAGGCACATCCAACAGTTAGTAGGGTTTTGGGCCGAGACCTCATGAAACCCAGTGAGGGTGGTATTAAATAGGCTTACCAGGCGAGTATGGGTACGGAGGGTTTCATGTAGTTTTGAGAGATCTAGTCCTTTGTAGGGGCTAGGCGTGCTATGTACTCAGGTCAGTTGGGAGATTACTTCCTTTACGTGTTTTTCTCTTGCCTGATCTTGAACTCCACCCCTACCAGACATACCAGTATGGGTGAACTAAGTCCAACAGACAGTGGCTCCAAGTCCTCCAGGACAACTAGGATTAATCATTTTCCCTGTCCAATAATGAGTATTTGCATGCATGCAAAGAGTGGCAGAGTTATAGCAGTTGTGGGGCATATGGGTGTGGGCAGTGAA

>GL-35-K9_T7_E07

GAGGTACCACAGACAAAAAATATTCCTGAGGGTAGGCAGACTATTCGTGTGGGAGGCGTTACCCACCTGATGCATTGGGAGTTGGCTGTGTCTACAGTATTGCTAAATTTTACACAGGTGAGGTTTGAGGTATGGGTTATTTCCAGATTGGAAACAAGAGGTCCTACTAAAACGGAAGTGGTGTTTATTTCTGTGCTGTAGTTGTTCCATTGTTCAGGTACAGGGATTGAAATGCATGGCCTGAAATACAGGGGGAGGCACATCCAACAGTTAGTAGGGTTTTGGGCCGAGACCTCATGGAGCCCAGTGAGGGTGGTATTAAATAGGCTTACCAGGCAAGTATGGGTATGGAGGGTTTCATGTAGTTTTAGGAGATCTAGTCCTTTGTAGGGGCTAGGGGTGCTATGTACCCGGGTCAGTTGGGAGATTACTTCCTTTACATGTTTTTCTCTTGCCTGATCTTGAACTCCACCCCCCTCAGACATACCAGTATGGGTGAAGTAAGTCCGACAGACAGTGGCTCCAAGTCTTCCAGGACAACTAGGATTAATCATTTTCCCTGTCCAATAATGAGTATTTGCATGCATGCAAAGAGTGGCAGAGTTATAGCAGTTGCGGGGCATATGGGTGTGGGCAGTGAA

>GL-35-K10_T7_A0

6GAGGTACCACAGACAAAAAATATTCCTGAGGGTAGGCAGACTATTCGTGTGGGAGGTGTTACCCACCTGATGCATTGGGAGCTGGTTGTGTCTATAGTATTGCTAAATTTTACACAGGTGAGGTTTGAGGTATGGGTTATTTCCAGATTGGAAACAAGAGGTCCTACTAAAACGGAAGTGGTGTTTATTTCTGTGCTGAAGTTGTTCCATTGTTCAGGTACAGGGATTGAAACATATGGCCTGAAGTTCAGGGGGAGGCATATCCAACAGTTAGTAGGGTTTTGGGCCGAGACCTCATGGAGCCCAGTGAGGGTGGTATTAAATAGGCTTACCAGGCGAGTATGGGTACGGAGGGTTTCATGTAGTTTTGAGAGATCTAGTCCTTTGTAGGGGCTAGAGGTGCCATGTACCCGGGTGAGTTGGGAGATTACTTCTTTTACATGTTTTTCTCTTGCCTGATCTTGAACTCCACCCCCATCAGACATACCAGTTTGGGTGAAGTAAGTCCAACAGACAGTGACTCCAAGTCCTCCAGGACAACTAGGATTAATCATTTTTCCTGTCCAATAATGAGTATTTGCATGCATGCAAAGAGTGGCAGAGTGATAGCAGTTGCGGGGCATATGGGTGTGGGCAGTGAA

>GL-35-K12_T7_H07

GAGGTACCACAGACAAAAAATATTCCTGAGGGTAGGCAGACTATTCGTGTGGGAGGAGTTACCCACCTGATGCATTGGGAATTGGTTGTGTCTATAGTATTGCTAAATTTTACACAGGTGAGGTTTGAGGTATGGGTTGTTTCCAGATTGGAAACAAGAGGTCCTACTAAAAAGGAAGTAGTATTTCTGTGCTGAAGTTGTTCCATTGTTCGGCTACAGAGATTGAAATGTGTGGCCTGAAGTGCAGGGGGAGGCACATCCAACAGTTAGTAGGGTTTTGGGCTGAGACCTCATGGAGCCCAGTGAGGGTGGTATTAAATAGGCTTACCAGGCAAGTATGGGTATGGAGGGTTTCATGTAGTTTTGAGAGATCTAGTCCTTTGTAGGGGCTAGGGGTGCTATGTACCCGGGTCAGTTGGGAGATTACTTCCTTTACGTGTTTTTCTCTTGCCTGATCTTGAACTCCACCCCCATCAGACAACAATATGCGTGAAGTAAGCCCAACAGACAGTGGTTCCCAGTCCTCCAGAACAACTAGGATTAATCATTTTCCCTGTCCAATAATGAGTATTTGCATGCATGCAAAGAGTGGCAGAGTTATAGCAATTGCGGGACATATGGGTGTGGGCAGTGAA

>GL-35-K13_T7_A08

GAGGTACCACGGACAAAAAATATTCCTGAGGGTAGGCAGACTATTCGTGTGGGAGGTGTTACCCACCTGATGCATTGGGAGCTGGTTGTGTCTATAGTATTGCTAAATTTTACACAGGTGAGGTTTGAGGTATGGGTTATTTCCAGATTGGAAACAAGAGGTCCTACTAAAACGGAAGTGGTGTTTATTTCTGTGCTGAAGTTGTTCCATTGTTCAGGTACAGGGATTGAAACATATGGCCTGAAGTTCAGGGGGAGGCATATCCAACAGTTAGTAGGGTTTTGGGCCGAGACCTCATGGAGCCCAGTGAGGGTGATATTAAATAGGCTTACCAGGCGAGTATGGGTACGGAGGGTTTCATGTAGTTTTGAGAGATCTAGTCCTTTGTAGGGGCTAGAGGTGCCATGTACCCGGGTGAGTTGGGAGATTACTTCTTTTACATGTTTTTCTCTTGCCTGATCTTGAACTCCACCCCCATCAGACATACCAGTTTGGGTGAAGTAAGTCCAACAGGCAGTGACTCCAAGTCCTCCAGGACAACTAGGATTAATCATTTTTCCTGTCCAATAATGAGTATTTGCATGCATGCAAAGAGTGGCAGAGTGATAGCAGTTGCGGGGCATATGGGTGTGGGCAGTGAA

>GL-35-K16_T7_C08

GAGGTACCACAGACAAAAAATATTCCTGAGGGTAGGCAGACTATTCGTGTGGGAGGTGTTACCCACCTGATGCATTGGGAGCTGGTTGTGTCTATAGTATTGCTAAATTTTACACAGGTGAGGTTTGAGGTATGGGTTATTTCCAGATTGGAAACAAGAGGTCCTACTAAAACGGAAGTGGTGTTTATTTCTGTGCTGAAGTTGTTCCATTGTTCAGGAACAGGGATTGAAATGTATGGCCTGAAGTGCAGGGGGAGGCACATCCAACAGTTAGTAGGGTTTTGGGCTGAGACCTCATGGAGCCGAGTGAGGGTGGTATTAAATAGGCTCACCAGGCAAGTATGGGTACGGAGGGTTTCATGTAGTTTTGAGAGAACTAGTCCTTTGTAGGGGCTAGGGGTGCTATGTCCTCGGGTCAGTTGGGAGATTGCTTCCTTTACTTGTTTTTCTCTTGCCTGACCTTGAATTCCACCCCCATCAGACATACTGGTATGGGTGAAGTAAGTCCAACAGACAGTGGCTCCAAGTCCTCCAGGACAACTAGGATTAATCATTTTCCCTGTCCAATAATGAGTATTTGTATGCATGCAAAGAGTGGCAGAGTTATAGCAGTTGCGGGGCATATGGGTGTGGGCAGTGAA

>GL-35-K17_T7_D08

GAGGTACCACAGACAAAAAATATTCCTGAGGGTAGGCAGACTATTTGTGTGGGAGGAGTTACCCACCTGATGCATTGGGAGTTGGTTGTGTATGTAGTATTGCTAAATTTTACACAGGTGAGGTTTGAGGTATGGGTTATTTCCAGATTGGAAACAAGAGGTCCTACTAAAACGGAAGTGGTGTTTATTTCTGTGCTGAAGTTGTTCCATTGTTCAGGTACAGGGATTGAAACATATGGCCTGAAGTTCAGGGGGAGGCATATCCAACAGTTAGTAGGGTTTTGGGCCGAGACCTCATGGAGCCCAGTGAGGGTGGTATTAAATAGGCTTACCAGGCGAGTATGGGTACGGAGGGTTTCATGTAGTTTTGAGAGATCTAGTCCTTTGTAGGGGCTAGAGGTGCCATGTACCCGGGTGAGTTGGGAGATTACTTCTTTTACATGTTTTTCTCTTGCCTGATCTTGAACTCCACCCCCATCAGACATACCAGTTTGGGTGAAGTAAGTCCAACAGACAGTGACTCCAAGTCCTCCAGGACAACTAGGATTAATCATTTTTCCTGTCCAATAATGAGTATTTGCATGCATGCAAAGAGTGGCAGAGTGATAGCAGTTGCGGGGCATATGGGTGTGGGCAGTGAA

>GL-35-K18_T7_E08

GAGGTACCACAGACAAAAAATATTCCTGAGGGTAGGCAGACTATTCGTGTGGGAGGTGTTACCCACCTGATGCATTGGGAGCTGGTTGTGTCTATAGTATTGCTAAATTTTACACAGGTGAGGTTTGAGGTATGGGTTATTTCCAGATTGGAAACAAGAGGTCCTACTAAAACGGAAGTGGTGTTTATTTCTGTGCTGAAGTTGTTCCATTGTTCAGGAACAGGGATTGAAATGTATGGCCTGAAGTGCAGGGGGAGGCACATCCAACAGTTAGTAGGGTTTTGGGCTGAGACCTCATGGAGCCGAGTGAGGGTGGTATTAAATAGGCTCACCAGGCGAGTATGGGTACGGAGGGTTTCATGTAGTTTTGAGAGAACTAGTCCTTTGTAGGGGCTAGGGGTGCTATGTCCCCGGGTCAGTTGGGAGATTGCTTCCTTTACTTGTTTTTCTCTTGCCTGACCTTGAATTCCACCCCCATCAGACATACTGGTATGGGTGAAGTAAGTCCAACAGACAGTGGCTCCAAGTCCTCCAGGACAACTAGGATTAATCATTTTCCCTGTCCAATAATGAGTATTTGCATGCATGCAAAGAGTGGCAGAGTTATAGCAGTTGCGGGGCATATGGGTGTGGGCAGTGAATTATA

>GL-35-K21_T7_F08

GAGGTACCACAGACAAAAAATATTCCTGAGGGTAGGCAGACTATTTGTGTGGGAGGAGTTACCCACCTGATGCATTGGGAGTTGGTTGTGTATGTAGTATTGCTAAATTTTACACAGGTGAGGTTTGAGGTATGGGTTATTTCCAGATTGGAAACAAGAGGTCCTACTAAAACGGAAGTGGTGTTTATTTCTGTGCTGAAGTTGTTCCATTGTTCAGGTACAGGGATTGAAACATATGGCCTGAAGTTCAGGGGGAGGCATATCCAACAGTTAGTAGGGCTTTGGGCCGAGACCTCATGGAGCCCAGTGAGGGTGGTATTAAATAGGCTTACCAGGCGAGTATGGGTACGGAGGGTTTCATGTAGTTTTGAGAGATCTAGTCCTTTGTAGGGGCTAGAGGTGCCATGTACCCGGGTGAGTTGGGAGATTACTTCTTTTACATGTTTTTCTCTTGCCTGATCTTGAACTCCACCCCCATCAGACATACCAGTTTGGGTGAAGTAAGTCCAACAGACAGTGACTCCAAGTCCTCCAGGACAACTAGGATTAATCATTTTTCCTGTCCAATAATGAGTATTTGCATGCATGCAAAGAGTGGCAGAGTGATAGCAGTTGCGGGGCATATGGGTGTGGGCAGTGAA

>GL-35-K22_T7_G08

GAGGTACCACAGACAAAAAATATTCCTGAGGGTAGGCAGACTATTCGTGTGGGAGGAGTTACCCACCTGATGCATTGGGAGTTGGTTGTGTCTACAGTATTGCTAAATTTTACACAGGTGAGGTTTGAGGTATGGGTTATTTCCAGATTGGAAACAAGAGGTCCTACTAAAACGGAAGTGGTGTTTATTTCTGTGCTGTAGTTGTTCCATTGTTCAGGTACAGGGATTGAAATGCATGGCCTGAAATACAGGGGGAGGCACATCCAACAGTTAGTAGGGTTTTGGGCCGAGACCTCATGGAGCCCAGTGAGGGTGGTATTAAATAGGCTTACCAGGCAAGTATGGGTATGGAGGGTTTCATGTAGTTTTAGGAGATCTAGTCCTTTGTGGGGGCTAGGGGTGCTATGTACCCGGGTCAGTTGGGAGATTACTTCCTTTACATGTTTTTCTCTTGCCTGATCTTGAACTCCACCCCCCTCAGACATACCAGTATGGGTGAAGTAAGTCCGACAGACAGTGGCTCCAAGTCTTCCAGGACAACTAGGATTAATCATTTTCCCTGTCCAATAATGAGTATTTGCATGCATGCAAAGAGTGGCAGAGTTATAGCAGTTGTGGGGCATATGGGTGTGGGCAGTGAA

>GL-35-K23_T7_H08

GAGGTACCACAGACAAAAAATATTCCTGAGGGTAGGCAGACTATTTGTGTGGGAGGAGTTACCCACCTGATGCATTGGGAGTTGGTTGTGTATGTAGTATTGCTAAATTTTACACAGGTGAGGTTTGAGGTATGGGTTATTTCCAGATTGGAAACAAGAGGTCCTACTAAAACGGAAGTGGTGTTTATTTCTGTGCTGAAGTTGTTCCATTGTTCAGGTACAGGGATTGAAACATATGGCCTGAAGTTCAGGGGGAGGCATATCCAACAGTTAGTAGGGTTTTGGGCCGAGACCTCATGGAGCCCAGTGAGGGTGGTATTAAATAGGCTTACCAGGCGAGTATGGGTACGGAGGGTTTCATGTAGTTTTGAGAGATCTAGTCCTTTGTAGGGGCTAGAGGTGCCATGTACCCGGGTGAGTTGGGAGATTACTTCTTTTACATGTTTTTCTCTTGCCTGATCTTGAACTCCACCCCCATCAGACATACCAGTTTGGGTGAAGTAAGTCCAACAGACAGTGACTCCAAGTCCTCCAGGACAACTAGGATTAATCATTTTTCCTGTCCAATAATGAGTATTTGCATGCATGCAAAGAGTGGCAGAGTGATAGCAGTTGCGGGGCATATGGGTGTGGGCAGTGAA

>GL-35-K25_T7_A09

GAGGTACCACAGACAAAAAATATTCCTGAGGGTAGGCAGACTATTCGTGGGGGGGAGTTACCCACCTGATGCATTGGGAGTTGGTTGTGTCTATCGTATTGCTAAATTTTACACAGGTGAGGTTTGAGGTATGGGTTATTTCCAGATTGGAAACAAGAGGTCCTACTAAAACGGAAGTGGTGTTTATGTGTTGAAGTTGTTCCCTTGTTCAGGTACAGGGATTGAAATGTATGGCCTTAAGTGCAGAGGGAGGCACATCCAACAGTTAGTAGGGTTTTGGGCCGAGACCTCATGAAACCCAGTGAGGGTGGTATTAAATAGGCTTACCAGGCGAGTATAGGTACGGAGGGTTTCATGTAGTTTTGAGAGATCTAGTCCTTTGTAGGGGCTAGGCGTGCTATGTACTCAGGTCAGTTGGGAGATTACTTCCTTTACGTGTTTTTCTCTTGCCTGATCTTGAACTCCACCCCTATCAGACATACCAGTATGGGTGAACTAAGTCCAACAGACAGTGGCTCCAAGTCCTCCAGGACAACTAGGATTAATCATTTTCCCTGTCCAATAATGAGTATTTGCATGCATGCAAAGAGTGGCAGAGTTATAGCAGTTGTGGGGCATATGGGTGTGGGCAGTGAA

>GL-35-K26_T7_B09

GAGGTACCACAGACAAAAAATATTCCTGAGGGTAGGCAGACTATTTGTGTGGGAGGAGTTACCCACCTGATGCATTGGGAGTTGGTTGTGTATGTAGTATTGCTAAATTTTACACAGGTGAGGTTTGAGGTATGGGTTATTTCCAGATTGGAAACAAGAGGTCCTACTAAAACGGAAGTGGTGTTTATTTCTGTGCTGAAGTTGTTCCATTGTTCAGGAACAGGGATTGAAATGTATGGCCTGAAGTGCAGGGGGAGGCACATCCAACAGTTAGTAGGGTTTTGGGCTGAGACCTCATGGAGCCGAGTGAGGGTGGTATTAAATAGGCTCACCAGGCGAGTATGGGTACGGAGGGTTTCATGTAGTTTTGAGAGAACTAGTCCTTTGTAGGGGCTAGGGGTGCTATGTCCCCGGGTCAGTTGGGAGATTGCTTCCTTTACTTGTTTTTCTCTTGCCTGACCTTGAATTCCACCCCCATCAGACATACTGGTATGGGTGAAGTAAATCCAACAGACAGTGGCTCCAAGTCCTCCAGGACAACTAGGATTAATCATTTTCCCTGTCCAATAATGAGTATTTGCATGCATGCAAAGAGTGGCAGAGTTATAGCAGTTGCGGGGCATATGGGTGTGGGCAGTGAA

>GL-35-K27_T7_C09

GAGGTACCACAGACAAAAAATATTCCTGAGGGTAGGCAGACTATTCGTGTGGGAGGTGTTACCCACCTGATGCATTGGGAGCTGGTTGTGTCTATAGTATTGCTAAATTTTACACAGGTGAGGTTTGAGGTATGGGTTATTTCCAGATTGGAAACAAGAGGTCCTACTAAAACGGAAGTGGTGTTTATTTCTGTGCTGAAGTTGTTCCATTGTTCAGGAACAGGGATTGAAATGTATGGCCTGAAGTGCAGGGGGAGGCACATCCAACAGTTAGTAGGGTTTTGGGCTGAGACCTCATGGAGCCGAGTGAGGGTGGTATTAAATAGGCTCACCAGGCGAGTATGGGTACGGAGGGTTTCATGTAGTTTTGAGAGAACTAGTCCTTTGTAGGGGCTAGGGGTGCTATGTCCCCGGGTCAGTTGGGAGATTGCTTCCTTTACTTGTTTTTCTCTTGCCTGACCTTGAATTCCACCCCCATCAGACATACTGGTATGGGTGAAGTAAGTCCAACAGACAGTGGCTCCAAGTCCTCCAGGACAACTAGGATTAATCATTTTCCCTGTCCAATAATGAGTATTTGCATGCATGCAAAGAGTGGCAGAGTTATAGCAGTTGCGGGGCATATGGGTGTGGGCAGTGAA

>GL-35-K28_T7_D09

GAGGTACCACAGACAAAAAATATTCCTGAGGGTAGGCAGACTATTCGTGGGGGGGAGTTACCCACCTGATGCATTGGGAGTTGGTTGTGTCTATCGTATTGCTAAATTTTACACAGGTGAGGTTTGAGGTATGGGTTATTTCCAGATTGGAAACAAGAGGTCCTACTAAAACGGAAGTGGTGTTTATGTGTTGAAGTTGTTCCCTTGTTCAGGTACAGGGATTGAAATGTATGGCCTTAAGTGCAGAGGGAGGCACATCCAACAGTTAGTAGGGTTTTGGGCCGAGACCTCATGAAACCCAGTGAGGGTGGTATTAAATAGGCTTACCAGGCGAGTATGGGTACGGAGGGTTTCATGTAGTTTTGAGAGATCTAGTCCTTTGTAGGGGCTAGGCGTGCTATGTACTCAGGTCAGTTGGGAGATTACTTCCTTTACGTGTTTTTCTCTTGCCTGATCTTGAACTCCACCCCTATCAGACATACCAGTATGGGTGAACTAAGTCCAACAGACAGTGGCTCCAAGTCCTCCAGGACAACTAGGATTAATCATTTTCCCTGTCCAATAATGAGTATTTGCATGCATGCAAAGAGTGGCAGAGTTATAGCAGTTGTGGGGCATATGGGTGTGGGCAGTGAA

>GL-35-K29_T7_E09

GAGGTACCACAGACAAAAAATATTCCTGAGGGTAGGCAGACTATTCGTGGGGGGGAGTTACCCACCTGATGCATTGGGAGTTGGTTGTGTCTATCGTATTGCTAAATTTTACACAGGTGAGGTTTGAGGTATGGGTTATTTCCAGATTGGAAACAAGAGGTCCTACTAAAACGGAAGTGGTGTTTATGTGTTGAAGTTGTTCCCTTGTTCAGGTACAGGGATTGAAATGTATGGCCTTAAGTGCAGAGGGAGGCACATCCAATAGTTAGTAGGGTTTTGGGCCGAGACCTCATGAAACCCAGTGAGGGTGGTATTAAATAGGCTTACCAGGCGAGTATGGGTACGGAGGGTTTCATGTAGTTTTGAGAGATCTAGTCCTTTGTAGGGGCTAGGCGTGCTATGTACTCAGGTCAGTTGGGAGATTACTTCCTTTACGTGTTTTTCTCTTGCCTGATCTTGAACTCCACCCCTATCAGACATACCAGTATGGGTGAACTAAGTCCAACAGACAGTGGCTCCAAGTCCTCCAGGACAACTAGGATTAATCATTTTCCCTGTCCAATAATGAGTATTTGCATGCATGCAAAGAGTGGCAGAGTTATAGCAGTTGTGGGGCATATGGGTGTGGGCAGTGAA

>GL-35-K30_T7_B06

GAGGTACCACAGACAAAAAATATTCCTGAGGGTAGGCAGACTATTTGTGTGGGAGGAGTTACCCACCTGATGCATTGGGAGTTGGTTGTGTATGTAGTATTGCTAAATTTTACACAGGTGAGGTTTGAGGTATGGGTTATTTCCAGATTGGAAACAAGAGGTCCTACTAAAACGGAAGTGGTGTTTATTTCTGTGCTGAAGTTGTTCCATTGTTCAGGTACAGGGATTGAAACATATGGCCTGAAGTTCAGGGGGAGGCATATCCAACAGTTAGTAGGGTTTTGGGCCGAGACCTCATGGAGCCCAGTGAGGGTGGTATTAAATAGGCTTACCAGGCGAGTATGGGTACGGAGGGTTTCATGTAGTTTTGAGAGATCTAGTCCTTTGTAGGGGCTAGAGGTGCCATGTACCCGGGTGAGTTGGGAGATTACTTCTTTTACATGTTTTTCTCTTGCCTGATCTTGAACTCCACCCCCATCAGACATACCAGTTTGGGTGAAGTAAGTCCAACAGACAGTGGTTCCCAGTCCTCCAGAACAACTAGGATTAATCATTTTCCCTGTCCAATAATGAGTATTTGCATGCATGCAAAGAGTGGCAGAGTTATAGCAATTGCGGGACATATGGGTGTGGGCAGTGAA

>GL-35-K31_T7_G09

GAGGTACCACAGACAAAAAATATTCCTGAGGGTAGGCAGACTATTCGTGTGGGAGGAGTTACCCACCTGATGCATTGGGAGTTGGTTGTGTCTACAGTATTGCTAAATTTTACACAGGTGAGGTTTGAGGTATGGGTTATTTCCAGATTGGAAACAAGGGGTCCTACTAAAACGGAAGTGGTGTTTATTTCTGTGCTGTAGTTGTTCCATTGTTCAGGTACAGGGATTGAAATGCATGGCCTGAAATACAGGGGGAGGCACATCCAACAGTTAGTAGGGTTTTGGGCCGAGACCTCATGGAGCCCAGTGAGGGTGGTATTAAATAGGCTTACCAGGCAAGTATGGGTATGGAGGGCTTCATGTAGTTTTAGGAGATCTAGTCCTTTGTAGGGGCTAGGGGTGCTATGTCCCCGGGTCAGTTGGGAGATTGCTTCCTTTACTTGTTTTTCTCTTGCCTGACCTTGAATTCCACCCCCATCAGACATACTGGTATGGGTGAAGTAAGTCCAACAGACAGTGGCTCCAAGTCCTCCAGGACAACTAGGATTAATCATTTTCCCTGTCCAATAATGAGTATTTGCATGCATGCAAAGAGTGGCAGAGTTATAGCAGTTGCGGGGCATATGGGTGTGGGCAGTGAA

>GL-35-K33_T7_A10

TTCACTGCCCACACCCATATGCCCCGCAACTGCTATCACTCTGCCACTCTTTGCATGCATGCAAATACTCATTATTGGACAGGAAAAATGATTAATCCTAGTTGTCCTGGAGGACTTGGAGTCACTGTCTGTTGGACTTACTTCACCCAAACTGGTATGTCTGATGGGGGTGGAGTTCAAGATCAGGCAAGAGAAAAACATGTAAAAGAAGTAATCTCCCAACTCACCCGGGTACATGGCACCTCTAGCCCCTACAAAGGACTAGATCTCTCAAAACTACATGAAACCCTCCGTACCCATACTCGCCTGGTAAGCCTATTTAATACCACCCTCACTGGGCTCCATGAGGTCTCGGCCCAAAACCCTACTAACTGTTGGATATGCCTCCCCCTGAACTTCAGGCCATATGTTTCAATCCCTGTACCTGAACAATGGAACAACTTCAGCACAGAAATAAACACCACTTCCGTTTTAGTAGGACCTCTTGTTTCCAATCTGGAAATAACCCATACCTCAAACCTCACCTGTGTAAAATTTAGCAATACTACATACACAACCAACTCCCAATGCATCAGGTGGGTAACTCCTCCCACACAAATAGTCTGCCTACCCTCAGGAATATTTTTTGTCTGTGGTACCTC

>GL-35-K35_T7_B10

GAGGTACCACAGACAAAAAATATTCCTGAGGGTAGGCAGACTATTCGTGTGGGAGGAGTTACCCACCTGATGCATTGGGAGTTGGTTGTGTCTACAGTATTGCTAAATTTTACACAGGTGAGGTTTGAGGTATGGGTTATTTCCAGATTGGAAACAAGAGGTCCTACTAAAACGGAAGTGGTGTTTATTTCTGTGCTGTAGTTGTTCCATTGTTCAGGTACAGGGATTGAAATGCATGGCCTGAAGTGCAGGGGGAGGCACATCCAACAGTTAGTAGGGTTTTGGGCTGAGACCTCATGGAGCCGAGTGAGGGTGGTATTAAATAGGCTCACCAGGCGAGTATGGGTACGGAGGGTTTCATGTAGTTTTGAGAGAACTAGTCCTTTGTAGGGGCTAGGGGTGCTATGTCCCCGGGTCAGTTGGGAGATTGCTTCCTTTACTTGTTTTTCTCTTGCCTGACCTTGAATTCCACCCCCATCAGACATACTGGTATGGGTGAAGTAAGACCAACAGACAGTGGCTCCAAGTCCTCCAGGACAACTAGGATTAATCATTTTCCCTGTCCAATAATGAGTATTTGCATGCATGCAAAGAGTGGCAGAGTTATAGCAGTTGCGGGGCATATGGGTGTGGGCAGTGAA

>GL-35-K36_T7_C10

TTCACTGCCCACACCCATATGCCCCACAACTGCTATAACTCTGCCACTCTTTGCATGCATGCAAATACTCATTATTGGACAGGGAAAATGATTAATCCTAGTTGTCCTGGAAGACTTGGAGCCACTGTCTGTTGGACTTACTTCACCCATACCAGTATGTCTGATGGGGGTGGAATTCAAGGTCAGGCAAGAGAAAAACAAGTAAAGGAAGCAATCTCCCAACTGACCCGGGGACATAGCACCCCTAGCCCCTACAAAGGACTAGTTCTCTCAAAACTACATGAAACCCTCCGTACCCATACTCGCCTGGTGAGCCTATTTAATACCACCCTCACTCGGCTCCATGAGGTCTCAGCCCAAAACCCTACTAACTGTTGGATGTGCCTCCCCCTGCACTTCAGGCCATACATTTCAATCCCTGTTCCTGAACAATGGAACAACTTCAGCACAGAAATAAACACCACTTCCGTTTTAGTAGGACCTCTTGTTTCCAATCTGGAAATAACCCATACCTCAAACCTCACCTGTGTAAAATTTAGCAATACTATAGACACAACCAGCTCCCAATGCATCAGGTGGGTAACACCTCCCACACGAATAGTCTGCCTACCCTCAGGAATATTTTTTGTCTGTGGTACCTC

**KO-IV**

>GL-38-K1_F06

TTCACTGCCCACACCCATATGCCCCACAACTGCTATAACTCTGCCACTCTTTGCATGCATGCAAATACTCATTATTGGACAGGGAAAATGATTAATCCTAGTTGTCCTGGAAGACTTGGAGCCACTGTCTGTCGGACTTACTTCACCCATACTGGTATGTCTGAGGGGGGTGGAGTTCAAGATCAGGCAAGAGAAAAACATGTAAAGGAAGTAACCTCCCAACTGACCCGGGTACATAGCACCCCTAGCCCCTACAAAGGACTAGATCTCTTAAAACTACATGAAACCCTCCATACCCATACTTGCCTGGTAAGCCTATTTAATACCACCCTCACTGGGCTCCATGAGGTCTCGGCCCAAAACCCTACTAACTGTTGGATGTGCCTCCCCCTGTATTTCAGGCCATGCACTTCAATCCCTGTACCTGAACAATGGAACAACTACAGCACAGAAATAAACACCACTTCCGTTTTAGTAGGACCTCTTGTTTCCAATCTGGAAATAACCCATACCTCAAACCTCACCTGTGTAAAATTTAGCAATACTGTAGACACAACCAACTCCCAATGCATCAGGTGGGTAACTCCTCCCACACGAATAGTCTGCCTACCCTCAGGAATATTTTTTGTCTGTGGTACCTC

>GL-38-K2_G06

TCTCGGCCCAAAACCCTACTAACTGTTGGATGTGCCTCCCCCTGTATTTCAGGCCATGCATTTCAATCCCTGTACCTGAACAATGGAACAACTACAGCACAGAAATAAACACCACTTCCGTTTTAGTAGGACCTCTTGTTTCCAATCTGGAAATAACCCATACCTCAAACCTCACCTGTGTAAAATTTAGCAATACTGTAGACACAACCAACTCCCAATGCATCAGGTGGGTAACTCCTCCCACACGAATAGTCTGCCTACCCTCAGGAATATTTTTTGTCTGTGGTACCTC

>GL-38-K4_H06

TTCACTGCCCACACCCATATGCCCCACAACTGCTATAACTCTGCCACTCTTTGCATGCATGCAAATACTCATTATTGGACAGGGAAAATGATTAATCCTAGTTGTCCTGGAAGACTTGGAGCCACTGTCTGTCGGACTTACTTCACCCATACTGGTATGTCTGAGGGGGGTGGAGTTCAAGATCAGGCAAGAGAAAAACATGTAAAGGAAGTAACCTCCCAACTGACCCGGGTACATAGCACCCCTAGCCCCTACAAAGGACTAGATCTCTTAAAACTACATGAAACCCTCCATACCCATACTTGCCTGGTAAGCCTATTTAATACCACCCTCACTGGGCTCCATGAGGTCTCGGCCCAAAACCCTACTAACTGTTGGATGTGCCTCCCCCTGTATTTCAGGCCATGCATTTCAATCCCTGTACCTGAACAATGGAACAACTACAGCACAGAAATAAACACCACTTCCGTTTTAGTAGGACCTCTTGTTTCCAATCTGGAAATAACCCATACCTCAAACCTCACCTGTGTAAAATTTAGCAATACTGTAGATACAACCAACTCCCAATGCATCAGGTGGGTAACTCCTCCCACACGAATAGTCTGCCTACCCTCAGGAATATTTTTTGTCTGTGGTACCTC

>GL-38-K5_A07

TTCACTGCCCACACCCATATGCCCCGCAACTGCTATAACTCTGCCACTCTTTGCATGCATGCAAATACTCATTATTGGACAGGGAAAATGATTAATCCTAGTTGTCCTGGAGGACTTGGAGCCACTGTCTGTTGGACTTACTTCACCCATACCAGTATGTCTGATGGGGGTGGAATTCAAGGTCAGGCAAGAGAAAAACGAGTAAAGGAAGCAATCTCCCAACTGACCCGGGGACATAGCACCCCTAGCCCCTACAAAGGACTAGTTCTCTCAAAACTACATGAAACCCTCCGTACCCATACTCGCCTGGTGAGCCTATTTAATACCACCCTCACTCGGCTCCATGAGGTCTCAGCCCAAAACCCTACTAACTGTTGGATGTGCCTCCCCCTGCACTTCAGGCCATACATTTCAATCTCTGTTCCTGAACAATGGAACAACTTCAGCACAGAAATAAACACCACTTCCGTTTTAGTAGGACCTCTTGTTTCCAATCTGGAAATAACCCATACCTCAAACCTCACCTGTGTAAAATTTAGCAATACTATAGACACAACCAGCTCCCAATGCATCAGGTGGGTAACACCTCCCACACGAATAGTCTGCCTACCCTCAGGAATATTTTTTGTCTGTGGTACCTC

>GL-38-K6_B07

TTCACTGCCCACACCCATATGCCCCACAACTGCTATAACTCTGCCACTCTTTGCATGCATGCAAATACTCATTATTGGACAGGAAAAACGATTAATCCCAGTTGTCCTGGAGGACTTGGAGGACTCACTTCACTCATACCAGTATGTCTGATGGGGGTGGAGTTCAAGATCAGGCAACAGAAAAACACATAAAGGAAGTAATCTCCCAACTGACCTGGGTACATAGCACCCCTGGCCCCTACAAAGGACTAGATCTCTCAAAACTACATGAAACCCTCCGTACCCATACTCGCCTGGTAAGCCTATTTAATACCACCCTCACTGGGTTTCATGAGGTCTCGGCCCAAAACCCTACTAACTGTTGGATATGCCTCCCCCTGAACTTCAGGCCATATGTTTCAATCCCTGTACCTGAACAATGGAACAACTTCAGCACAGAAATAAACACCACTTCCGTTTTAGTAGGACCTCTTGTTTCCAATCTGGAAATAACCCATACCTCAAACCTCACCTGTGTAAAATTTAGCAATACTACATACACAACCAACTCCCAATGCATCAGGTGGGTAACTCCTCCCACACAAATAGTCTGCCTACCCTCAGGAATATTTTTTGTCTGTGGTACCTC

>GL-38_K7_C07

TTCACTGCCCACACCCATATGCCCCACAACTGCTATAACTCTGCCACTCTTTGCATGCATGCAAATACTCATTATTGGACAGGGAAAATGATTAATCCTAGTTGTCCTGGAAGACTTGGAGCCACTGTCTGTCGGACTTACTTCACCCATACTGGTATGTCTGAGGGGGGTGGAGTTCAAGATCAGGCAAGAGAAAAACATGTAAGGGAAGTAACCTCCCAACTGACCCGGGTACATAGCACCCCTAGCCCCTACAAAGGACTAGATCTCTTAAAACTACATGAAACCCTCCATACCCATACTTGCCTGGTAAGCCTATTTAATACCACCCTCACTGGGCTCCATGAGGTCTCGGCCCAAAACCCTACTAACTGTTGGATGTGCCCCCCCCTGTATTTCAGGCCATGCATTTCAATCCCTGTACCTGAACAATGGAACAACTACAGCACAGAAATAAACACCACTTCCGTTTTAGTAGGACCTCTTGTTTCCAATCTGGAAATAACCCATACCTCAAACCTCACCTGTGTAAAATTTAGCAATACTATAGACACAACCAGCTCCCAATGCATCAGGTGGGTAACACCTCCCACACGAATAGTCTGCCTACCCTCAGGAATATTTTTTGTCTGTGGTACCTC

>GL-38-K8_D07

TTCACTGCCCACACCCATATGCCCCACAACTGCTATAACTCTGCCACTCTTTGCATGCATGCAAATACTCATTATTGGACAGGGAAAATGATTAATCCTAGTTGTCCTGGAAGACTTGGAGCCACTGTCTGTCGGACTTACTTCACCCATACTGGTATGTCTGAGGGGGGTGGAGTTCAAGATCAGGCAAGAGAAAAACATGTAAAGGAAGTAACCTCCCAACTGACCCGGGTACATAGCACCCCTAGCCCCTACAAAGGACTAGATCTCTTAAAACTACATGAAACCCTCCATACTCATACTTGCCTGGTAAGCCTATTTAATACCACCCTCACTGGGCTCCATGAGGTCTCGGCCCAAAACCCTACTAACTGTTGGATGTGCCTCCCCCTGTATTTCAGGCCATGCATTTCAATCCCTGTACCTGAACAATGGAACAACTACAGCACAGAAATAAACACCACTTCCGTTTTAGTAGGACCTCTTGTTTCCAATCTGGAAATAACCCATACCTCAAACCTCACCTGTGTAAAATTTAGCAATACTGTAGACACAACCAACTCCCAATGCATCAGGTGGGTAACTCCTCCCACACGAATAGTCTGCCTACCCTCAGGAATATTTTTTGTCTGTGGTACCTC

>GL-38-K10_E07

TTCACTGCCCACACCCATATGCCCCGCAACTGCTATAACTCTGCCACTCTTTGCATGCATGCAAATACTCATTATTGGACAGGGAAAATGATTAATCCTAGTTGTCCTGGAGGACTTGGAGCCACTGTCTGTTGGACTTACTTCACCCATACCAGTATGTCTGATGGGGGTGGAATTCAAGGTCAGGCAAGAGAAAAACAAGTAAAGGAAGCAATCTCCCAACTGACCCGGGGACATAGCACCCCTAGCCCCTACAAAGGACTAGTTCTCTCAAAACTACATGAAACCCTCCGTACCCATACTCGCCTGGTGAGCCTATTTAATACCACCCTCACTCGGCTCCATGAGGTCTCAGCCCAAAACCCTACTAACTGTTGGATGTGCCTCCCCCTGCACTTCAGGCCATACATTTCAATCCCTGTTCCTGAACAATGGAACAACTTCAGCACAGAAATAAACACCACTTCCGTTTTAGTAGGACCTCTTGTTTCCAATCTGGAAATAACCCATACCTCAAACCTCACCTGTGTAAAATTTAGCAATACTATAGACACAACCAGCTCCCAATGCATCAGGTGGGTAACACCTCCCACACGAATAGTCTGCCTACCCTCAGGAATATTTTTTGTCTGTGGTACCTC

>GL-38-K11_F07

TTCACTGCCCACACCCATATGCCCCGCAACTGCTATAACTCTGCCACTCTTTGCATGCATGCAAATACACATTATTGGACAGGGAAAATGATTAATCCTAGTTGTCCTGGAGGACTTGGAGCCACTGTCTGTTGGACTTACTTCACCCATACCAGTATGTCTGATGGGGGTGGAATTCAAGGTCAGGCAAGAGAAAAACAAGTAAAGGAAGCAATCTCCCAACTGACCCGGGGACATAGCACCCCTAG

>GL-38-K12_G07

TTCACTGCCCACACCCATATGCCCCGCAACTGCTATAACTCTGCCACTCTTTGCATGCATGCAAATACTCATTATTGGACAGGGAAAATGATTAATCCTAGTTGTCCTGGAGGACTTGGAGCCACTGTCTGTTGGACTTACTTCACCCATACCAGTATGTCTGATGGGGGTGGAATTCAAGGTCAGGCAAGAGAAAAACAAGTAAAGGAAGCAATCTCCCAACTGACCCGGGGACATAGCACCCCTAGCCCCTACAAAGGACTAGTTCTCTCAAAACTACATGAAACCCTCCGTACCCATACTCGCCTGGTGAGCCTATTTAATACCACCCTCACTCGGCTCCATGAGGTCTCAGCCCAAAACCCTACTAACTGTTGGATGTGCCTCCCCCTGCACTTCAGGCCATACATTTCAATCCCTGTTCCTGAACAATGGAACAACTTCAGCACAGAAATAAACACCACTTCCGTTTTAGTAGGACCTCTTGTTTCCAATCTGGAAATAACCCATACCTCAAACCTCACCTGTGTAAAATTTAGCAATACTATAGACACAACCAGCTCCCAATGCATCAGGTGGGTAACACCTCCCACACGAATAGTCTGCCTACCCTCAGGAATATTTTTTGTCTGTGGTACCTC

>GL-38-K13_H07

TTCACTGCCCACACCCATATGCCCCACAACTGCTATAACTCTGCCACTCTTTGCATGCATGCAAATACTCATTATTGGACAGGGAAAATGATTAATCCTAGTTGTCCTGGAAGACTTGGAGCCACTGTCTGTCGGACTTACTTCACCCATACTGGTATGTCTGAGGGGGGTGGAGTTCAAGATCAGGCAAGAGAAAAACATGTAAAGGAAGTAACCTCCCAACTGACCCGGGTACATAGCACCCCTAGCCCCTACAAAGGACTAGATCTCTTAAAACTACATGAAACCCTCCATACCCATACTTGCCTGGTAAGCCTATTTAATACCACCCTCACTGGGCTCCATGAGGTCTCGGCCCAAAACCCTACTAACTGTTGGATGTGCCTCCCCCTGTATTTCAGGCCATGCATTTCAATCCCTGTACCTGAACAATGGAACAACTACAGCACAGAAATAAACACCACTTCCGTTTTAGTAGGACCTCTTGTTTCCAATCTGGAAATAACCCATACCTCAAACCTCACCTGTGTAAAATTTAGCAATACTGTAGACACAACCAACTCCCAATGCATCAGGTGGGTAACTCCTCCCACACGAATAGTCTGCCTACCCTCAGGAATATTTTTTGTCTGTGGTACCTC

>GL-38-K14_A08

TTCACTGCCCACACCCATATGCCCCACAACTGCTATAACTCTGCCACTCTTTGCATGCATGCAAATACTCATTATTGGACAGGGAAAATGATTAATCCTAGTTGTCCTGGAAGACTTGGAGCCACTGTCTGTCGGACTTACTTCACCCATACTGGTATGTCTGAGGGGGGTGGAGTTCAAGATCAGGCAAGAGAAAAACATGTAAAGGAAGTAACCTCCCAACTGACCCGGGTACATAGCACCCCTAGCCCCTACAAAGGACTAGATCTCTTAAAACTACATGAAACCCTCCATACCCATACTTGCCTGGTAAGCCTATTTAATACCACCCTCACTGGGCTCCATGAGGTCTCGGCCCAAAACCCTACTAACTGTTGGATGTGCCTCCCCCTGTATTTCAGGCCATGCATTTCAATCCCTGTACCTGAACAATGGAACAACTACAGCACAGAAATAAACACCACTTCCGTTTTAGTAGGACCTCTTGTTTCCAATCTGGAAATAACCCATACCTCAAACCTCACCTGTGTAAAATTTAGCAATACTGTAGACACAACCAACTCCCAATGCATCAGGTGGGTAACTCCTCCCACACGAATAGTCTGCCTACCCTCAGGAATATTTTTTGTCTGTGGTACCTC

>GL-38-K16_C08

TTCACTGCCCACACCCATATGCCCCGCAACTGCTATAACTCTGCCACTCTTTGCATGCATGCAAATACTCATTATTGGACAGGGAAAATGATTAATCCTAGTTGTCCTGGAGGACTTGGAGCCACTGTCTGTTGGACTTACTTCACCCATACCAGTATGTCTGATGGGGGTGGAATTCAAGGTCAGGCAAGAGAAAAACAAGTAAAGGAAGCAATCTCCCAACTGACCCGGGGACATAGCACCCCTAGCCCCTACAAAGGACTAGTTCTCTCAAAACTACATGAAACCCTCCGTACCCATACTCGCCTGGTGAGCCTATTTAATACCACCCTCACTCGGCTCCATGAGGTCTCAGCCCAAAACCCTACTAACTGTTGGATGTGCCTCCCCCTGCACTTCAGGCCATACATTTCAATCCCTGTTCCTGAACAATGGAACAACTTCAGCACAGAAATAAACACCACTTCCGTTTTAGTAGGACCTCTTGTTTCCAATCTGGAAATAACCCATACCTCAAACCTCACCTGTGTAAAATTTAGCAATACTATAGACACAACCAGCTCCCAATGCATCAGGTGGGTAACACCTCCCACACGAATAGTCTGCCTACCCTCAGGAATATTTTTTGTCTGTGGTACCTC

>GL-38-K17_D08

TTCACTGCCCACACCCATATGCCCCACAACTGCTATAACTCTGCCACTCTTTGCATGCATGCAAATACTCATTATTGGACAGGAAAAACGATTAATCCCAGTTGTCCTGGAGGACTTGGAGGACTCACTTCACTCATACCAGTATGTCTGATGGGGGTGGAGTTCAAGATCAGGCAACAGAAAAACACATAAAGGAAGTAATCTCCCAACTGACCTGGGTACATAGCACCCCTGGCCCCTACAAAGGACTAGATCTCTCAAAACTACATGAAACCCTCCATACCCATACTGGCCTGGTAAGCCTATTTAATACCACCCTGACTGGGCTCCATGAGGTCTCGGCCCAAAACCCTACTAACTATTGGATGTGCCTCCCCCTGCACTTTAGGCCATACATTTCAATCCCTATACCTGAACAATGGAACAACTTCAGCACAGAAATAAACACCACTTCTGTTTTAGTAGGTCCTCTTTCCAATCTGGAAATAACCCATACCTCAAACCTCACCTGTGTAAAATTTAGCAATACTATAGACACAGCCAACTCCCAATGCATCAGGTGGGTAACTCCTCCCACACGAATAGTCTGCCTACCCTCAGGAATATTTTTTGTCTGTGGTACCTC

>GL-38-K18_E08

TTCACTGCCCACACCCATATGCCCCACAACTGCTATAACTCTGCCACTCTTTGCATGCATGCAAATACTCATTATTGGACAGGGAAAATGATTAATCCTAGTTGTCCTGGAAGACTTGGAGCCACTGTCTGTCGGACTTACTTCACCCATACTGGTATGTCTGAGGGGGGTGGAGTTCAAGATCAGGCAAGAGAAAAACATGTAAAGGAAGTAACCTCCCAACTGACCCGGGTACATAGCACCCCTAGCCCCTACAAAGGACTAGATCTCTTAAAACTACATGAAACCCTCCATACCCATACTTGCCTGGTAGGCCTATTTAATACCACCCTCACTGGGCTCCATGAGGTCTCGGCCCAAAACCCTACTAACTGTTGGATGTGCCTCCCCCTGTATTTCAGGCCATGCATTTCAATCCCTGTACCTGAACAATGGAACAACTACAGCACAGAAATAAACACCACTTCCGTTTTAGTAGGACCTCTTGTTTCCAATCTGGAAATAACCCATACCTCAAACCTCACCTGTGTAAAATTTAGCAATACTGTAGACACAACCAACTCCCAATGCATCAGGTGGGTAACTCCTCCCACACGAATAGTCTGCCTACCCTCAGGAATATTTTTTGTCTGTGGTACCTC

>GL-38-K19_F08

TTCACTGCCCACACCCATATGCCCCGCAACTGCTATCACTCTGCCACTCTTTGCATGCATGCAAATACTCATTATTGGACAGGAAAAATGATTAATCCTAGTTGTCCTGGAGGACTTGGAGTCACTGTCTGTTGGACTTACTTCACCCAAACTGGTATGTCTGATGGGGGTGGAGTTCAAGATCAGGCAAGAGAAAAACATGTAAAAGAAGTAATCTCCCAACTCACCCGGGTACATGGCACCTCTAGCCCCTACAAAGGACTAGATCTCTCAAAACTACATGAAACCCTCCGTACCCATACTCGCCTGGTAAGCCTATTTAATACCACCCTCACTGGGCTCCATGAGGTCTCGGCCCAAAACCCTACTAACTGTTGGATATGCCTCCCCCTGAACTTCAGGCCATATGTTTCAATCCCTGTACCTGAACAATGGAACAACTTCAGCACAGAAATAAACACCACTTCCGTTTTAGTAGGACCTCTTGTTTCCAATCTGGAAATAACCCATACCTCAAACCTCACCTGTGTAAAATTTAGCAATACTACATACACAACCAACTCCCAATGCATCAGGTGGGTAACTCCTCCCACACAAATAGTCTGCCTACCCTCAGGAATATTTTTTGTCTGTGGTACCTC

>GL-38-K20_G08

CTGTACCTGAACAATGGAACAACTACAGCACAGAAATAAACACCACTTCCGTTTTAGTAGGACCTCTTGTTTCCAATCTGGAAATAACCCATACCTCAAACCTCACCTGTGTAAAATTTAGCAATACTGTAGACACAACCAACTCCCAATGCATCAGGTGGGTAACTCCTCCCACACGAATAGTCTGCCTACCCTCAGGAATATTTTTTGTCTGTGGTACCTC

>GL-38-K21_H08

TTCACTGCCCACACCCATATGCCCCACAACTGCTATAACTCTGCCACTCTTTGCATGCATGCAAATACTCATTATTGGACAGGGAAAATGATTAATCCTAGTTGTCCTGGAAGACTTGGAGCCACTGTCTGTCGGACTTACTTCACCCATACTGGTATGTCTGAGGGGGGTGGAGTTCAAGATCAGGCAAGAGAAAAACATGTAAAGGAAGTAACCTCCCAACTGACCCGGGTACATAGCACCCCTAGCCCCTACAAAGGACTAGATCTCTTAAAACTACATGAAACCCTCCATACCCATACTTGCCTGGTAAGCCTATTTAATACCACCCTCACTGGGCTCCATGAGGTCTCGGCCCAAAACCCTACTAACTGTTGGATGTGCCTCCCCCTGTATTTCAGGCCATGCATTTCAATCCCTGTACCTGAACAATGGAACAACTACAGCACAGAAATAAACACCACTTCCGTTTTAGTAGGACCTCTTGTTTCCAATCTGGAAATAACCCATACCTCAAACCTCACCTGTGTAAAATTTAGCAATACTGTAGACACAACCAACTCCCAATGCATCAGGTGGGTAACTCCTCCCACACGAATAGTCTGCCTACCCTCAGGAATATTTTTTGTCTGTGGTACCTC

>GL-38-K24_B09

TTCACTGCCCACACCCATATGCCCCACAACTGCTAATAACTCTGCCACTCTTGCATGCATGCAAATACTCATTATTGGACAGGGAAAATGATTAATCCTAGTTGTCCTGGAAGACTTGGAGCCACTGTCTGTCGGACTTACTTCACCCATACTGGTATGTCTGAGGGGGGTGGAGTTCAAGATCAGGCAAGAGAAAAACATGTAAAGGAAGTAACCTCCCAACTGACCCGGGTACATAGCACCCCTAGCCCCTACAAAGGACTAGATCTCTTAAAACTACATGAAACCCTCCATACCCATACTTGCCTGGTAAGCCTATTTAATACCACCCTCACTGGGCTCCATGAGGTCTCGGCCCAAAACCCTACTAACTGTTGGATGTGCCTCCCCCTGTATTTCAGGCCATGCATTTCAATCCCTGTACCTGAACAATGGAACAACTACAGCACAGAAATAAACACCACTTCCGTTTTAGTAGGACCTCTTGTTTCCAATCTGGAAATAACCCATACCTCAAACCTCACCTGTGTAAAATTTAGCAATACTGTAGACACAACCAACTCCCAATGCATCAGGTGGGTAACTCCTCCCACACGAATAGTCTGCCTACCCTCAGGAATATTTTTTGTCTGTGGTACCTC

>GL-38-K25_C09

TTCACTGCCCACACCCATATGCCCCACAACTGCTATAACTCTGCCACTCTTTGCATGCATGCAAATACTCATTATTGGACAGGGAAAATGATTAATCCTAGTTGTCCTGGAAGACTTGGAGCCACTGTCTGTCGGACTTACTCCACCCATACTGGTATGTCTGAGGGGGGTGGAGTTCAAGATCAGGCAAGAGAAAAACATGTAAAGGAAGTAACCTCCCAACTGACCCGGGTACACAGCACCCCTAGCCCCTACAAAGGACTAGATCTCTTAAAACTACATGAAACCCTCCATACCCATACTTGCCTGGTAAGCCTATTTAATACCACCCTCACTGGGCTCCATGAGGTCTCGGCCCAAAACCCTACTAACTGTTGGATGTGCCTCCCCCTGTATTTCAGGCCATGCATTTCAATCCCTGTACCTGAACAATGGAACAACTACAGCACAGAAATAAACACCACTTCCGTTTTAGTAGGACCTCTTGTTTCCAATCTGGAAATAACCCATACCTCAAACCTCACCTGTGTAAAATTTAGCAATACTGTAGACACAACCAACTCCCAATGCATCAGGTGGGTAACTCCTCCCACACGAATAGTCTGCCTACCCTCAGGAATATTTTTTGTCTGTGGTACCTC

>GL-38-K26_D09

TTCACTGCCCACACCCATATGCCCCGCAACTGCTATAACTCTGCCACTCTTTGCATGCATGCAAATACTCATTATTGGACAGGGAAAATGATTAATCCTAGTTGTCCTGGAGGACTTGGAGCCACTGTCTGTTGGACTTACTTCACCCATACCAGTATGTCTGATGGGGGTGGAATTCAAGGTCAGGCAAGAGAAAAACAAGTAAAGGAAGCAATCTCCCAACTGACCCGGGGACATAGCACCCCTAGCCCCTACAAAGGACTAGTTCTCTCAAAACTACATGAAACCCTCCGTACCCATACTCGCCTGGTGAGCCTATTTAATACCACCCTCACTCGGCTCCATGAGGTCTCAGCCCAAAACCCTACTAACTGTTGGATGTGCCTCCCCCTGCACTTCAGGCCATACATTTCAATCCCTGTTCCTGAACAATGGAACAACTTCAGCACAGAAATAAACACCACTTCCGTTTTAGTAGGACCTCTTGTTTCCAATCTGGAAATAACCCATACCTCAAACCTCACCTGTGTAAAATTTAGCAATACTATAGACACAACCAGCTCCCAATGCATCAGGTGGGTAACACCTCCCACACGAATAGTCTGCCTACCCTCAGGAATATTTTTTGTCTGTGGTACCTC

>GL-38-K28_E09

TTCACTGCCCACACCCATATGCCCCACAACTGCTATAACTCTGCCACTCTTTGCATGCATGCAAATACTCATTATTGGACAGGGAAAATGATTAATCCTAGTTGTCCTGGAAGACTTGGAGCCACTGTCTGTCGGACTTACTTCACCCATACTGGTATGTCTGAGGGGGGTGGAGTTCAAGATCAGGCAAGAGAAAAACATGTAAAGGAAGTAACCTCCCAACTGACCCGGGTACATAGCACCCCTAGCCCCTACAAAGGACTAGATCTCTTAAAACTACATGAAACCCTCCATACCCATACTTGCCTGGTAAGCCTATTTAATACCACCCTCACTGGGCTCCATGAGGTCTCGGCCCAAAACCCTACTAACTGTTGGATGTGCCTCCCCCTGTATTTCAGGCCATGCATTTCAATCCCTGTACCTGAACAATGGAACAACTACAGCACAGAAATAAACACCACTTCCGTTTTAGTAGGACCTCTTGTTTCCAATCTGGAAATAACCCATACCTCAAACCTCACCTGTGTAAAATTTAGCAATACTGTAGACACAACCAACTCCCAATGCATCAGGTGGGTAACTCCTCCCACACGAATAGTCTGCCTACCCTCAGGAATATTTTTTGTCTGTGGTACCTC

>GL-38-K33_F09

GAAGACTTGAGCCACTGTCTGTCGGACTTACTTCACCCATACTGGTATGTCTGAGGGGGGTGGAGTTCAAGATCAGGCAAGAGAAAAACATGTAAAGGAAGTAACCTCCCAACTGACCCGGGTACATAGCACCCCTAGCCCCTACAAAGGACTAGATCTCTTAAAACTACATGAAACCCTCCATACCCATACTTGCCTGGTAAGCCTATTTAATACCACCCTCACTGGGCTCCATGAGGTCTCGGCCCAAAACCCTACTAACTGTTGGATGTGCCTCCCCCTGTATTTCAGGCCATGCATTTCAATCCCTGTACCTGAACAATGGAACAACTACAGCACAGAAATAAACACCACTTCCGTTTTAGTAGGACCTCTTGTTTCCAATCTGGAAATAACCCATACCTCAAACCTCACCTGTGTAAAATTTAGCAATACTGTAGACACAACCAACTCCCAATGCATCAGGTGGGTAACTCCTCCCACACGAATAGTCTGCCTACCCTCAGGAATATTTTTTGTCTGTGGTACCTC

>GL-38-K34_G09

TTCACTGCCCACACCCATATGCCCCACAACTGCTATAACTCTGCCACTCTTTGCATGCATGCAAATACTCATTATTGGACAGGGAAAATGATTAATCCTAGTTGTCCTGGAGGACTTGGAGCCACTGTCTGTTGGACTTAGTTCACCCATACTGGTATGTCTGATAGGGGTGGAGTTCAAGATCAGGCAAGAGAAAAACACGTAAAGGAAGTAATCTCCCAACTGACCTGAGTACATAGCACGCCTAGCCCCTACAAAGGACTAGATCTCTCAAAACTACATGAAACCCTCCGTACCCATACTCGCCTGGTAAGCCTATTTAATACCACCCTCACTGGGTTTCATGAGGTCTCGGCCCAAAACCCTACTAACTGTTGGATGTGCCTCCCTCTGCACTTAAGGCCATACATTTCAATCCCTGTACCTGAACAAGGGAACAACTTCAACACATAAACACCACTTCCGTTTTAGTAGGACCTCTTGTTTCCAATCTGGAAATAACCCATACCTCAAACCTCACCTGTGTAAAATTTAGCAATACGATAGACACAACCAACTCCCAATGCATCAGGTGGGTAACTCCCCCCCACGAATAGTCTGCCTACCCTCAGGAATATTTTTTGTCTGTGGTACCTC

>GL-38-K35_H09

TTCACTGCCCACACCCATATGCCCCACAACTGCTACAACTCTGCCACTCTTTGCATGCATGCAAATACTCATTATTGGACAGGGAAAATGATTAATCCTAGTTGTCCTGGAAGACTTGGAGCCACTGTCTGTCGGACTTACTTCACCCATACTGGTATGTCTGAGGGGGGTGGAGTTCAAGATCAGGCAAGAGAAAAACATGTAAAGGAAGTAACCTCCCAACTGACCCGGGTACATAGCACCCCTAGCCCCTACAAAGGACTAGATCTCTTAAAACTACATGAAACCCTCCATACCCATACTTGCCTGGTAAGCCTATTTAATACCACCCTCACTGGGCTCCATGAGGTCTCGGCCCAAAACCCTACTAACTGTTGGATGTGCCTCCCCCTGTATTTCAGGCCATGCATTTCAATCCCTGTACCTGAACAATGGAACAACTACAGCACAGAAATAAACACCACTTCCGTTTTAGTAGGACCTCTTGTTTCCAATCTGGAAATAACCCATACCTCAAACCTCACCTGTGTAAAATTTAGCAATACTGTAGACACAACCAACTCCCAATGCATCAGGTGGGTAACTCCTCCCACACGAATAGTCTGCCTACCCTCAGGAATATTTTTTGTCTGTGGTACCTC

>GL-38-K36_A10

TTCACTGCCCACACCCATATGCCCCACAACTGCTATAACTCTGCCACTCTTTGCATGCATGCAAATACTCATTATTGGGCAGGGAAAATGATTAATCCTAGTTGTCCTGGAGGACTTGGAGCCACTGTCTGTTGGACTTAGTTCACCCATACTGGTATGTCTGATAGGGGTGGAGTTCAAGATCAGGCAAGAGAAAAACACGTAAAGGAAGTAATCTCCCAACTGACCTGAGTACATAGCACGCCTAGCCCCTACAAAGGACTAGATCTCTCAAAACTACATGAAACCCTCCGTACCCATACTCGCCTGGTAAGCCTATTTAATACCACCCTCACTGGGTTTCATGAGGTCTCGGCCCAAAACCCTACTAACTGTTGGATGTGCCTCCCTCTGCACTTAAGGCCATACATTTCAATCCCTGTACCTGAACAAGGGAACAACTTCAACGCATAAACACCACTTCCGTTTTAGTAGGACCTCTTGTTTCCAATCTGGAAATAACCCATACCTCAAACCTCACCTGTGTAAAATTTAGCAATACGATAGACACAACCAACTCCCAATGCATCAGGTGGGTAACTCCCCCCCACGAATAGTTTGCCTACCCTCAGGAATATTTTTTGTCTGTGGTACCTC

>GL-38-K37_B10

TTCACTGCCCACACCCATATGCCCCACAACTGCTATAACTCTGCCACTCTTTGCATGCATGCAAATACTCATTATTGGACAGGGAAAATGATTAATCCTAGTTGTCCTGGAAGACTTGGAGCCACTGTCTGTCGGACTTACTTCACCCATACTGGTATGTCTGAGGGGGTGGAGTTCAAGATCAGGCAAGAGAAAAACATGTAAAGGAAGTAACCTCCCAACTGACCCGGGTACATAGCACCCCTAGCCCCTACAAAGGACTAGATCTCTTAAAACTACATGAAACCCTCCATACCCATACTTGCCTGGTAAGCCTATTTAATACCACCCTCACTGGGCTCCATGAGGTCTCGGCCCAAAACCCTACTAACTGTTGGATGTGCCTCCCCCTGTATTTCAGGCCATGCATTTCAATCCCTGTACCTGAACAATGGAACAACTACAGCACAGAAATAAACACCACTTCTGTTTTAGTAGGTCCTCTTTCCAATCTGGAAATAACCCATACCTCAAACCTCACCTGTGTAAAATTTAGCAATACTATAGACACAGCCAACTCCCAATGCATCAGGTGGGTAACTCCTCCCACACGAATAGTCTGCCTACCCTCAGGAATATTTTTTGTCTGTGGTACCTC

>GL-38-K38_C10

TTCACTGCCCACACCCATATGCCCCACAACTGCTATAACTCTGCCACTCTTTGCATGCATGCAAATACTCATTATTGGACAGGGAAAATGATTAATCCTAGTTGTCCTGGAAGACTTGGAGCCACTGTCTGTCGGACTTACTTCACCCATACTGGTATGTCTGAGGGGGGTGGAGTTCAAGATCAGGCAAGAGAAAAACACGTAAAGGAAGTAATCTCCCAACTGACCTGAGTACATAGCACGCCTAGCCCCTACAAAGGACTAGATCTCTCAAAACTACATGAAACCCTCCGTACCCATACTCGCCTGGTAAGCCTATTTAATACCACCCTCACTGGGTTTCATGAGGTCTCGGCCCAAAACCCTACTAACTGTTGGATGTGCCTCCCTCTGCACTTAAGGCCATACATTTCAATCCCTGTACCTGAACAAGGGAACAACTTCAACACATAAACACCACTTCCGTTTTAGTAGGACCTCTTGTTTCCAATCTGGAAATAACCCATACCTCAAACCTCACCTGTGTAAAATTTAGCAATACGATAGACACAACCAACTCCCAATGCATCAGGTGGGTAACTCCCCCCCACGAATAGTCTGCCTACCCTCAGGAATATTTTTTGTCTGTGGTACCTC

>GL-38-K39_D10

TTCACTGCCCACACCCATATGCCCCACAACTGCTATAACTCTGCCACTCTTTGCATGCATGCAAATACTCATTATTGGACAGGGAAAATGATTAATCCTAGTTGTCCTGGAAGACTTGGAGCCACTGTCTGTCGGACTTACTTCACCCATACTGGTATGTCTGAGGGGGGTGGAGTTCAAGATCAGGCAAGAGAAAAACATGTAAAGGAAGTAACCTCCCAACTGACCCGGGTACATAGCACCCCTAGCCCCTACAAAGGACTAGATCTCTTAAAACTACATGAAACCCTCCATACCCATACTTGCCTGGTAAGCCTATTTAATACCACCCTCACTGGGCTCCATGAGGTCTCGGCCCAAAACCCTACTAACTGTTGGATGTGCCTCCCCCTGTATTTCAGGCCATGCATTTCAATCCCTGTACCTGAACAATGGAACAACTACAGCACAGAAATAAACACCACTTCCGTTTTAGTAGGACCTCTTGTTTCCAATCTGGAAATAACCCATACCTCAAACCTCACCTGTGTAAAATTTAGCAATACTGTAGACACAACCAACTCCCAATGCATCAGGTGGGTAACTCCTCCCACACGAATAGTCTGCCTACCCTCAGGAATATTTTTTGTCTGTGGTACCTC

>GL-38-K40_E10

TTCACTGCCCACACCCATATGCCCCACAACTGCTATAACTCTGCCACTCTTTGCATGCATGCAAATACTCATTATTGGACAGGGAAAATGATTAATCCTAGTTGTCCTGGAAGACTTGGAGCCACTGTCTGTCGGACTTACTTCACCCATACTGGTATGTCTGAGGGGGGTGGAGTTCAAGATCAGGCAAGAGAAAAACATGTAAAGGAAGTAACCTCCCAACTGACCCGGGTACATAGCACCCCTAGCCCCTACAAAGGACTAGATCTCTTAAAACTACATGAAACCCTCCATACCCATACTTGCCTGGTAAGCCTATTTAATACCACCCTCACTGGGCTCCATGAGGTCTCGGCCCAAAACCCTACTAACTGTTGGATGTGCCTCCTCCTGTATTTCAGGCCATGCATTTCAATCCCTGTACCTGAACAAGGGAACAACTTCAACACATAAACACCACTTCCGTTTTAGTAGGACCTCTTGTTTCCAATCTGGAAATAACCCATACCTCAAACCTCACCTGTGTAAAATTTAGCAATACGATAGACACAACCAACTCCCAATGCATCAGGTGGGTAACTCCCCCCCACGAATAGTCTGCCTACCTTCAGGAATATTTTTTGTCTGTGGTACCTC

>GL-38-K41_F10

TTCACTGCCCACACCCATATGCCCCACAACTGCTATAACTCTGCCACTCTTTGCATGCATGCAAATACTCATTATTGGACAGGAAAAACGATTAATCCCAGTTGTCCTGGAGGACTTGGAGGACTCACTTCACTCATACCAGTATGTCTGATGGGGGTGGAGTTCAAGATCAGGCAACAGAAAAACACATAAAGGAAGTAATCTCCCAACTGACCTGGGTACATAGCACCCCTGGCCCCTACAAAGGACTAGATCTCTCAAAACTACATGAAACCCTCCATACCCATACTGGCCTGGTAAGCCTATTTAATACCACCCTGACTGGGCTCCATGAGGTCTCGGCCCAAAACCCTACTAACTGTTGGATGTGCCTCCCCCTGCACTTTAGGCCATACATTTCAATCCCTATACCTGAACAATGGAACAACTTCAGCACAGAAATAAACACCACTTCTGTTTTAGTAGGTCCTCTTTCCAATCTGGAAATAACCCATACCTCAAACCTCACCTGTGTAAAATTTAGCAATACTATAGACACAGCCAACTCCCAATGCATCAGGTGGGTAACTCCTCCCACACGAATAGTCTGCCTACCCTCAGGAATATTTTTTGTCTGTGGTACCTC

>GL-38-K43_G10

TGGGTTTCATGAGGTCTCGGCCCAAAACCCTACTAACTGTGGGATGTGCCTCCCTCTGCACTTAAGGCCATACATTTCAATCCCTGTACCTGAACAAGGGAACAACTTCAACACATAAACACCACTTCCGTTTTAGTAGGACCTCTTGTTTCCAATCTGGAAATAACCCATACCTCAAACCTCACCTGTGTAAAATTTAGCAATACGATAGACACAACCAACTCCCAATGCATCAGGTGGGTAACTCCCCCCCACGAATAGTCTGCCTACCCTCAGGAATATTTTTTGTCTGTGGTACCTC

>GL-38-K44_H10

TTCACTGCCCACACCCATATGCCCCGCAACTGCTATAACTCTGCCACTCTTTGCATGCATGCAAATACTCATTATTGGACAGGGAAAATGATTAATCCTAGTTGTCCTGGAGGACTTGGAGCCACTGTCTGTTGGACTTACTTCACCCATACCAGTATGTCTGATGGGGGTGGAATTCAAGGTCAGGCAAGAGAAAAACAAGTAAAGGAAGCAATCTCCCAACTGACCCGGGGACATAGCACCCCTAGCCCCTACAAAGGACTAGTTCTCTCAAAACTACATGAAACCCTCCGTACCCATACTCGCCTGGTGAGCCTATTTAATACCACCCTCACTCGGCTCCATGAGGTCTCAGCCCAAAACCCTACTAACTGTTGGATGTGCCTCCCCCTGCACTTCAGGCCATACATTTCAATCCCTGTTCCTGAACAATGGAACAACTTCAGCACAGAAATAAACACCACTTCCGTTTTAGTAGGACCTCTTGTTTCCAATCTGGAAATAACCCATACCTCAAACCTCACCTGTGTAAAATTTAGCAATACTATAGACACAACCAGCTCCCAATGCATCAGGTGGGTAACACCTCCCACACGAATAGTCTGCCTACCCTCAGGAATATTTTTTGTCTGTGGTACCTC

>GL-38-K45_A11

TTCACTGCCCACACCCATATGCCCCACAACTGCTATAACTCTGCCACTCTTTGCATGCATGCAAATACTCATTATTGGACAGGGAAAATGATTAATCCTAGTTGTCCTGGAGGACTTGGAGCCACTGTCTGTTGGACTTAGTTCACCCATACTGGTATGTCTGATAGGGGTGGAGTTCAAGATCAGGCAAGAGAAAAACACGTAAAGGAAGTAATCTCCCAACTGACCTGAGTACATAGCACGCCTAGCCCCTACAAAGGACTAGATCTCTCAAAACTACATGAAACCCTCCGTACCCATACTCGCCTGGTAAGCCTATTTAATACCACCCTCACTGGGTTTCATGAGGTCTCGGCCCAAAACCCTACTAACTGTTGGATGTGCCTCCCTCTGCACTTAAGGCCATACATTTCAATCCCTGTACCTGAACAAGGGAACAACTTCAACACATAAACACCACTTCCGTTTTAGTAGGACCTCTTGTTTCCAATCTGGAAATAACCCATACCTCAAACCTCACCTGTGTAAAATTTAGCAATACGATAGACACAACCAACTCCCAATGCATCAGGTGGGTAACTCCCCCCCACGAATAGTCTGCCTACCCTCAGGAATATTTTTTGTCTGTGGTACCTC

>GL-38-K46_B11

TTCACTGCCCACACCCATATGCCCCACAACTGCTATAACTCTGCCACTCTTTGCATGCATGCAAATACTCATTATTGGACAGGAAAAACGATTAATCCCAGTTGTCCTGGAGGACTTGGAGGACTCACTTCACTCATACCAGTATGTCTGATGGGGGTGGAGTTCAAGATCAGGCAACAGAAAAACACATAAAGGAAGTAATCTCCCAACTGACCTGGGTACATAGCACCCCTGGCCCCTACAAAGGACTAGATCTCTCAAAACTACATGAAACCCTCCATACCCATACTGGCCTGGTAAGCCTATTTAATACCACCCTGACTGGGCTCCATGAGGTCTCGGCCCAAAACCCTACTAACTGTTGGATGTGCCTCCCCCTGCACTTTAGGCCATACATTTCAATCCCTATACCTGAACAATGGAACAACTTCAGCACAGAAATAAACACCACTTCTGTTTTAGTAGGTCCTCTTTCCAATCTGGAAATAACCCATACCTCAAACCTCACCTGTGTAAAATTTAGCAATACTATAGACACAGCCAACTCCCAATGCATCAGGTGGGTAACTCCTCCCACACGAATAGTCTGCCTACCCTCAGGAATATTTTTTGTCTGTGGTACCTC

>GL-38-K47_C11

TTCACTGCCCACACCCATATGCCCCACAACTGCTATAACTCTGCCACTCTTTGCATGCATGCAAATACTCATTATTGGACAGGGAAAATGATTAATCCTAGTTGTCCTGGAAGACTTGGAGCCACTGTCTGTCGGACTTACTTCACCCATACTGGTATGTCTGAGGGGGGTGGAGTTCAAGATCAGGCAAGAGAAAAACATGTAAAGGAAGTAACCTCCCAACTGACCCGGGTACATAGCACCCCTAGCCCCTACAAAGGACTAGATCTCTTAAAACTACATGAAACCCTCCATACCCATACTTGCCTGGTAAGCCTATTTAATACCACCCTCACTGGGCTCCATGAGGTCTCGGCCCAAAACCCTACTAACTGTTGGATGTGCCTCCCCCTGTATTTCAGGCCATGCATTTCAATCCCTGTACCTGAACAATGGAACAACTACAGCACAGAAATAAACACCACTTCCGTTTTAGTAGGACCTCTTGTTTCCAATCTGGAAATAACCCATACCTCAAACCTCACCTGTGTAAAATTTAGCAATACTGTAGACACAACCAACTCCCAATGCATCAGGTGGGTAACTCCTCCCACACGAATAGTCTGCCTACCCTCAGGAATATTTTTTGTCTGTGGTACCTC

>GL-38-K52_D11

TTCACTGCCCACACCCATATGCCCCACAACTGCTATAACTCTGCCACTCTTTGCATGCATGCAAATACTCATTATTGGACAGGGAAAATGATTAATCCTAGTTGTCCTGGAGGACTTGGAGCCACTGTCTGTTGGACTTAGTTCACCCATACTGGTATGTCTGATAGGGGTGGAGTTCAAGATCAGGCAAGAGAAAAACACGTAAAGGAAGTAATCTCCCAACTGACCTGAGCACATAGCACGCCTAGCCCCTACAAAGGACTAGATCTCTCAAAACTACATGAAACCCTCCGTACCCATACTCGCCTGGTAAGCCTATTTAATACCACCCTCACTGGGTTTCATGAGGTCTCGGCCCAAAACCCTACTAACTGTTGGATGTGCCTCCCTCTGCACTTAAGGCCATACATTTCAATCCCTGTACCTGAACAAGGGAACAACTTCAACACATAAACACCACTTCCGTTTTAGTAGGACCTCTTGTTTCCAATCTGGAAATAACCCATACCTCAAACCTCACCTGTGTAAAATTTAGCAATACGATAGACACAACCAACTCCCAATGCATCAGGTGGGTAACTCCCCCCCACGAATAGTCTGCCTACCCTCAGGAATATTTTTTGTCTGTGGTACCTC

>GL-38-K54_E11

TTCACTGCCCACACCCATATGCCCCACAACTGCTATAACTCTGCCACTCTTTGCATGCATGCAAATACTCATTATTGGACAGGAAAAACGATTAATCCCAGTTGTCCCGGAGGACTTGGAGGACTCACTTCACTCATACCAGTATGTCTGATGGGGGTGGAGTTCAAGATCAGGCAACAGAAAAACACATAAAGGAAGTAATCTCCCAACTGACCTGGGTACATAGCACCCCTGGCCCCTACAAAGGACTAGATCTCTCAAAACTACATGAAACCCTCCATACCCATACTGGCCTGGTAAGCCTATTTAATACCACCCTGACTGGGCTCCATGAGGTCTCGGCCCAAAACCCTACTAACTGTTGGATGTGCCTCCCCCTGCACTTTAGGCCATACATTTCAATCCCTGTACCTGAACAATGGAACAACTTCAGCACAGAAATAAACACCACTTCTGTTTTAGTAGGTCCTCTTTCCAATCTGGAAATAACCCATACCTCAAACCTCACCTGTGTAAAATTTAGCAATACTATAGACACAGCCAACTCCCAATGCATCAGGTGGGTAACTCCTCCCACACGAATAGTCTGCCTACCCTCAGGAATATTTTTTGTCTGTGGTACCTC

>GL-38-K57_G11

TTCACTGCCCACACCCATATGCCCCACAACTGCTATAACTCTGCCACTCTTTGCATGCATGCAAATACTCATTATTGGACAGGGAAAATGATTAATCCTAGTTGTCCTGGAGGACTTGGAGCCACTGTCTGTTGGACTTAGTTCACCCATACTGGTATGTCTGATAGGGGTGGAGTTCAAGATCAGGCAAGAGAAAAACACGTAAAGGAAGTAATCTCCCAACTGACCTGAGTACATAGCACGCCTAGCCCCTACAAAGGACTAGATCTCTCAAAACTACATGAAACCCTCCGTACCCATACTCGCCTGGTAAGCCTATTTAATACCACCCTCACTGGGTTTCATGAGGTCTCGGCCCAAAACCCTACTAACTGTTGGATGTGCCTCCCTCTGCACTTAAGGCCATACATTTCAATCCCTGTACCTGAACAAGGGAACAACTTCAACACATAAACACCACTTCCGTTTTAGTAGGACCTCTTGTTTCCAATCTGGAAATAACCCATACCTCAAACCTCACCTGTGTAAAATTTAGCAATACTGTAGACACAACCAACTCCCAATGCATCAGGTGGGTAACTCCTCCCACACGAATAGTCTGCCTACCCTCAGGAATATTTTTTGTCTGTGGTACCTC

>GL-38-K58_H11

TTCACTGCCCACACCCATATGCCCCGCAACTGCTATAACTCTGCCACTCTTTGCATGCATGCAAATACTCATTATTGGACAGGGAAAATGATTAATCCTAGTTGTCCTGGGGGACTTGGAGCCACTGTCTGTTGGACTTACTTCACCCATACCAGTATGTCTGATGGGGGTGGAATTCAAGGTCAGGCAGGAGAAAAACAAGTAAAGGAAGCAATCTCCCAACTGACCCGGGGGCATAGCACCCCTAGCCCCTACAAAGGACTAGTTCTCTCAAAACTACATGAAACCCTCCGTACCCATACTCGTCTGGTGAGCCTATTTAATACCACCCTCACTCGGCTCCATGAGGTCTCAGCCCAAAACCCTACTAACTGTTGGATGTGCCTCCCCCTGCACTTCAGGCCATACATTTCAATCCCTGTTCCTGAACAATGGAACAACTTCAACACATAAACACCACTTCCGTTTTAGTAGGACCTCTTGTTTCCAATCTGGAAATAACCCATACCTCAAACCTCACCTGTGTAAAATTTAGCAATACGATAGACACAACCAACTCCCAATGCATCAGGTGGGTAACACCTCCCACACGAATAGTCTGCCTACCCTCAGGAATATTTTTTGTCTGTGGTACCTC

>GL-38-K60_A12

TTCACTGCCCACACCCATATGCCCCGCAACTGCTATAACTCTGCCACTCTTTGCATGCATGCAAATACTCATTATTGGACAGGGAAAATGATTAATCCTAGTTGTCCTGGAGGACTTGGAGCCACTGTCTGTTGGACTTACTTCACCCATACCAGTATGTCTGATGGGGGTGGAATTCAAGGTCAGGCAAGAGAAAAACAAGTAAAGGAAGCAATCTCCCAACTGACCCGGGGACATAGCACCCCTAGCCCCTACAAAGGACTAGTTCTCTCAAAACTACATGAAACCCTCCGTACCCATACTCGCCTGGTGAGCCTATTTAATACCACCCTCACTGGGTTTCATGAGGTCTCGGCCCAAAACCCTACTAACTGTTGGATGTGCCTCCCTCTGCACTTAAGGCCATACATTTCAATCCCTGTACCTGAACAAGGGAACAACTTCAACACATAAACACCACTTCCGTTTTAGTAGGACCTCTTGTTTCCAATCTGGAAATAACCCATACCTCAAACCTCACCTGTGTAAAATTTAGCAATACTGTAGACACAACCAACTCCCAATGCATCAGGTGGGTAACTCCTCCCACACGAATAGTCTGCCTACCCTCAGGAATATTTTTTGTCTGTGGTACCTC

>GL-38-K63_B12

TTCACTGCCCACACCCATATGCCCCACAACTGCTATAACTCTGCCACTCTTTGCATGCATGCAAATACTCATTATTGGACAGGGAAAATGATTAATCCTAGTTGTCCTGGAGGACTTGGAGCCACTGTCTGTCGGACTTACTTCACCCATACCAGTATGTCTGATGGGGGTGGAATTCAAGGTCAGGCAAGAGAAAAACAAGTAAAGGAAGCAATCTCCCAACTGACCCGGGGACATAGCACCCCTAGCCCCTACAAAGGACTAGTTCTCTCAAAACTACATGAAACCCTCCGTACCCATACTCGCCTGGTAAGCCTATTTAATACCACCCTCACTGGGCTCCATGAGGTCTCGGCCCAAAACCCTACTAACTGTTGGATGTGCCTCCCCCTGTATTTCAGGCCATGCATTTCAATCCCTGTACCTGAACAATGGAACAACTACAGCACAGAAATAAACACCACTTCCGTTTTAGTAGGACCTCTTGTTTCCAATCTGGAAATAACCCATACCTCAAACCTCACCTGTGTAAAATTTAGCAATACTGTAGACACAACCAACTCCCAATGCATCAGGTGGGTAACTCCTCCCACACGAATAGTCTGCCTACCCTCAGGAATATTTTTTGTCTGTGGTACCTC
