# Supplementary material for: Analysis of transcribed human endogenous retrovirus W env loci clarifies the origin of multiple sclerosis-associated retrovirus env sequences
Source: Retrovirology. 2009 Apr 15;6:37. doi: 10.1186/1742-4690-6-37 (PMC2672075; doi:10.1186/1742-4690-6-37)
Supplement: Additional file 2 — Pustell matrix comparisons of the seven HERV-W env loci identified as transcriptionally active in human PBMC in this study. This file contains Pustell matrix comparisons of the Repbase HERV-W reference sequence with the seven HERV-W env loci identified as transcriptionally active in human PBMC in this work. [file 1742-4690-6-37-S2.ppt]

## Slide 1
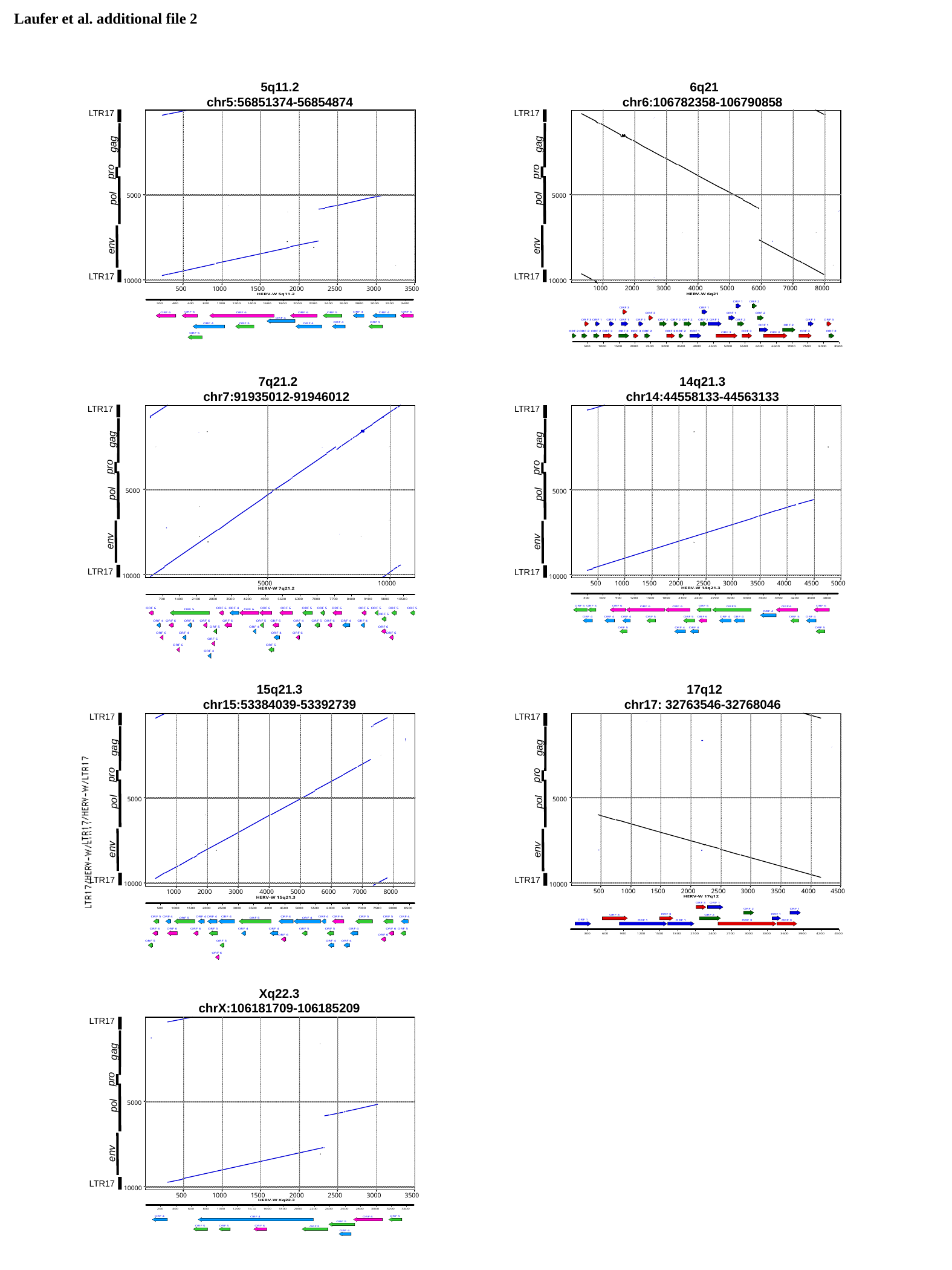

Laufer et al. additional file 2
5q11.2
chr5:56851374-56854874
6q21
chr6:106782358-106790858
LTR17
gag
pro
5000
pol
env
LTR17
10000
LTR17
gag
pro
5000
pol
env
LTR17
10000
1000
2000
3000
4000
5000
6000
7000
8000
500
1000
1500
2000
2500
3000
3500
7q21.2
chr7:91935012-91946012
14q21.3
chr14:44558133-44563133
LTR17
gag
pro
5000
pol
env
LTR17
10000
LTR17
gag
pro
5000
pol
env
LTR17
10000
5000
10000
500
1000
1500
2000
2500
3000
3500
4000
4500
5000
*
15q21.3
chr15:53384039-53392739
17q12
chr17: 32763546-32768046
LTR17
gag
pro
5000
pol
env
LTR17
10000
LTR17
gag
pro
5000
pol
env
LTR17
10000
500
1000
1500
2000
2500
3000
3500
4000
4500
1000
2000
3000
4000
5000
6000
7000
8000
Xq22.3
chrX:106181709-106185209
LTR17
gag
pro
5000
pol
env
LTR17
10000
500
1000
1500
2000
2500
3000
3500
**

## Slide 2
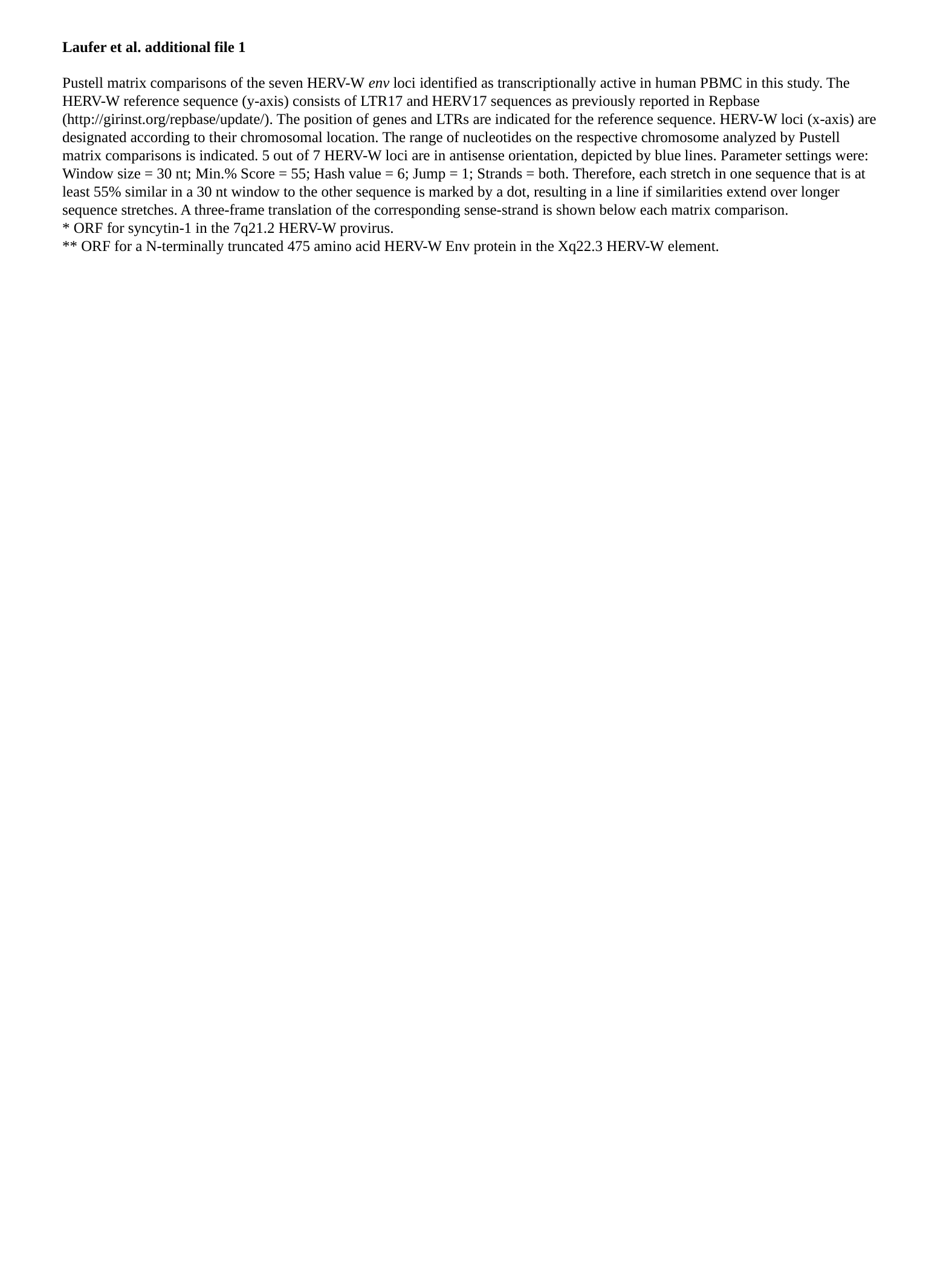

Laufer et al. additional file 1
Pustell matrix comparisons of the seven HERV-W env loci identified as transcriptionally active in human PBMC in this study. The HERV-W reference sequence (y-axis) consists of LTR17 and HERV17 sequences as previously reported in Repbase (http://girinst.org/repbase/update/). The position of genes and LTRs are indicated for the reference sequence. HERV-W loci (x-axis) are designated according to their chromosomal location. The range of nucleotides on the respective chromosome analyzed by Pustell matrix comparisons is indicated. 5 out of 7 HERV-W loci are in antisense orientation, depicted by blue lines. Parameter settings were: Window size = 30 nt; Min.% Score = 55; Hash value = 6; Jump = 1; Strands = both. Therefore, each stretch in one sequence that is at least 55% similar in a 30 nt window to the other sequence is marked by a dot, resulting in a line if similarities extend over longer sequence stretches. A three-frame translation of the corresponding sense-strand is shown below each matrix comparison.
* ORF for syncytin-1 in the 7q21.2 HERV-W provirus.
** ORF for a N-terminally truncated 475 amino acid HERV-W Env protein in the Xq22.3 HERV-W element.
