# Supplementary material for: Analysis of transcribed human endogenous retrovirus W env loci clarifies the origin of multiple sclerosis-associated retrovirus env sequences
Source: Retrovirology. 2009 Apr 15;6:37. doi: 10.1186/1742-4690-6-37 (PMC2672075; doi:10.1186/1742-4690-6-37)
Supplement: Additional file 3 — Alignments of previously published MSRV env and gag sequences with their corresponding genomic HERV-W elements. This file contains annotated alignments of previously published MSRV env and gag sequences with the genomic HERV-W elements from which the respective MSRV sequences are most likely derived. [file 1742-4690-6-37-S3.doc]

**Laufer et al. additional file 3**

Alignments of previously published MSRV *env* and *gag* sequences with their corresponding genomic HERV-W elements

MSRV *env* sequences:

**AF127227(CL4)***

chr3q23 1 CCTGGTAAGCCTATTTAATACCGCCCTCACTGGGCTCCATGAGGTCTCAGCCCAAAACCC

AF127227 1 ......G...............A...................C.................

chr3q23 61 TACTAACTGTTGGATGTGCCTCCCCCTGCACTTCAGGTCATACATTTCAATCCCTGTACC

AF127227 61 ............................................................

chr3q23 121 TGAACAATGGAACAACTTCAGCACAGAAATAAACACCACTTCCGTTTTGGTAGGACCTCT

AF127227 121 ............................................................

chr3q23 181 TGTTTCCAATCTGGAAATAACCCATACCTCAAACCTCACCTATGTAAAATTTAGCAATAC

AF127227 181 ............................................................

chr3q23 241 TATAGACACAACCAACTCCCAATGCATCAGGTGAATAACTCCTCCCACAGGAATAGTCTG

AF127227 241 ............................................................

chr3q23 301 CCTACCCCCAGGAATATTTTTGTCTGTGGCACCTCAGCCTATCGTTGTTTGAATGGCTCT

AF127227 301 ............................................................

chr3q23 361 TCAGAATCTATGTGCTTCCTCTCATTCTTAGTGCCCCCTATGACCATCTACACTGAACAA

AF127227 361 ............................................................

chr3q23 421 GATTTATACAATTATGTTGTACCTAAGCCCTGCAACAAAAGAGTACCCATTCTTCCTTTT

AF127227 421 ............................................................

chr3q23 481 GTTATCAGAGCAGGAGTGCTAGGTGGACTAGGTGCTGGCATTGGCAGTATCACAACCTCT

AF127227 481 ............................................................

chr3q23 541 ACTC

AF127227 541 ....

* The GenBank accession number as well as the MSRV clone designation according to Komurian-Pradel et al. [1] is indicated. Nucleotide differences between the MSRV sequence and the corresponding HERV-W element are highlighted in yellow. The primer sequences used for the amplification of the MSRV clone AF127227 as reported in Ref. [1] are underlined. Note that nucleotide differences that are located within the primer sequence do not represent true nucleotide mismatches because they are imposed upon the sequence by the primer. Nucleotide differences within the primer binding site were therefore not included when the nucleotide mismatches between MSRV sequences and the best matching HERV-W locus were enumerated (see Table 3).

**AF127229 (CL6)***

chr18q21.32 1 AAAGAAACTCAGAAAGCCAATACCCATTTAGTAAGATGGACACCTGAAGCAGAAGTGGCT

chr3p12.3 1 ..G.........................................A..........CA...

AF127229 1 ..G.........................................A..........CA...

chr18q21.32 61 TTCCAGGCCCTAAAGAAGGCCCTAACCCAAGCCCCAGTGTTAAGCTTGCCAACGGGGCAA

chr3p12.3 61 .................AT.........................................

AF127229 61 .................AT.........................................

chr18q21.32 121 GACTTTTCTTTATATGTCACAGAAAAAAACAGGAATAGCTCTAGAAGTCCTTACACAGGT

chr3p12.3 121 ...........................--...............G...............

AF127229 121 ...........................--...............G...............

chr18q21.32 181 CCAAAGGACAAGCTTGCAACCCATGGCATACCTGAATAAGGAAATTGATGTAGTGGCAAA

chr3p12.3 179 ....G................TG............G........C...............

AF127229 179 ....G................TG............G........C...............

chr18q21.32 241 GGTTTGGCCTCATTGATTATGGGTAGTGACAGCAGTAGCAGTCTTAGTATCTGAAGCAGT

chr3p12.3 239 ..G............T...CA.....G.G...................T......A....

AF127229 239 ..G............T...CA.....G.G...................T......A....

chr18q21.32 301 TAAAATAATACAGGGAAGAGATCTTACTGTGTAGACATCTAATGATGTGAACGGCACACT

chr3p12.3 299 ................................G.......C...............T...

AF127229 299 ................................G.......C...............T...

chr18q21.32 361 CACTGCTAAAGGAGACTTGTGGCTGTCAGACAACTGTTTACTTAAATATCAGGCTCTATT

chr3p12.3 359 ...........A......................CA............G....T......

AF127229 359 ...........AG.....................CA............G....T......

chr18q21.32 421 ACTTGAAGGTCCAGTGCTGTGACTGCGCACTTGTGCACCTCTTAACCCAGCCACATTTCT

chr3p12.3 419 ........TG.........C......A..T.......A......................

AF127229 419 ........TG.........C......A..T.......A......................

chr18q21.32 481 TACAGACAATGAAGAAAAGATAGAACATAACTGTCAACAAATAATTGCTCAAACCTATGC

chr3p12.3 479 .C......................................G...................

AF127229 479 .C......................................G...................

chr18q21.32 541 CGCTCAAGGGGACCTTTTAGAGGTTCCCTTGACTGATCCCAACCTCAACTTGTATACTGA

chr3p12.3 539 T....G..........C.......................G...................

AF127229 539 T....G..........C.......................G...................

chr18q21.32 601 TGGAAGTTCCTTTGTAGAAAAAGGACTTCAAAAAGTGGGGTATGGCAGTGGTCAGTGATA

chr3p12.3 599 ............G.C.............TG.....C........-.....A.........

AF127229 599 ............G.C.............TG.....C........-.....A.........

chr18q21.32 661 ATGGAATACTTGAAAGTAATCCCCTCACTCCAGGAACTACTGCTCAGCTGGCAGAACTAA

chr3p12.3 658 .....................G.................G......C.............

AF127229 658 .....................G.................G......C.............

chr18q21.32 721 TAGCTCTCACTCAGGAACTAGAATTAGGAGAAGGGAAAAGGGTAAATATATATACAGCCT

chr3p12.3 718 ....C......TG..C..................A..................T...A..

AF127229 718 ....C......TG..C..................A..................T...A..

chr18q21.32 781 CTAAGTATGCTTGCCTAGTCCTTCATGCCCATGCAGCAATATGGAGAGAAAGGGAATTCC

chr3p12.3 778 ............A.........C..........................G..........

AF127229 778 ............A.........C..........................G..........

chr18q21.32 841 TAACTTCCAAGGGAACACCAGTCAAACATCAGGAAGCTATTAGGAGATTATAACTGCCTG

chr3p12.3 838 .......TG..........TA....C...........C.............T.T..G...

AF127229 838 .......TG..........TA....C...........C.............T.T..G...

chr18q21.32 901 TACAGAAACCTAAAGAGGTGACAGTCTTACACTGCCAGGGTCATCAGAAAG-----GAAA

chr3p12.3 898 ....................G..............................AAGAG....

AF127229 898 ....................G..........................G...AAGAG....

chr18q21.32 956 GGGAAGTAGAAAGGAACTGCCAAGTGGATATTGAAGTCAAAAGAGC-ACAAGGCAGGACC

chr3p12.3 958 .....A.....G.C..TC......C...........CA....A...CG...........T

AF127229 958 .....A.....G.C..TC......C...........CA....A...CG...........T

chr18q21.32 1015 TTCCATTAGAAATGCTTATAGAAGGATGCCTAGTATAGGGTAATCCCCTCTGGGAAACCA

chr3p12.3 1018 C.........................CC........G.......................

AF127229 1018 C.........................CC........G.......................

chr18q21.32 1075 AGCCCCAGTACTCAGCAGGAAAAATAGAATAGGAAACCTCACAAGGACATACTTTCCTCC

chr3p12.3 1078 .......A..........A.G.........G....-.....TG.A......G........

AF127229 1078 ............................................................

chr18q21.32 1135 CCTCCAGATGGCTAGCCACTGAGGAAGGAAAAATACTTTCACCTGCAGCTAACCAACAGA

chr3p12.3 1137 ....AG.............CA.A................TG.T.............TG..

AF127229 1138 ............................................................

chr18q21.32 1195 AATTACTTAAAACCCTTCACCAAACCTTCCACTTAGGCATTGATAGCACCCATCAGATGG

chr3p12.3 1197 ............................T..............C...............A

AF127229 1198 ............................................................

chr18q21.32 1255 CCAAATTATTATTTACTGGACCAGGCCTTTTCAAAACTATCAAGAAGATAGTCAGGGGCT

chr3p12.3 1257 .....C..................A...................C............C..

AF127229 1258 ............................................................

chr18q21.32 1315 GTGAAGTGTGCCAAAGAAATAATCCCCTGCACTGCAGGCCATACATTTCAATCCCTGTAT

chr3p12.3 1317 ..................................T.....................C...

AF127229 1318 ............................................................

chr18q21.32 1375 CTTTAACCTCCTTGTTAAGTTTGTCTCTTCCAGAATCAAAACCGTAAAACTACAAATTGT

chr3p12.3 1377 T.....T.......A...C.....................G.T..............C..

AF127229 1378 ..........................................T.................

chr18q21.32 1435 TCTTCAAATGGAGCACCAGATGGAGTCCATGACTAAGATCCACCGTGGACCCCTGGACCG

chr3p12.3 1437 ..............C.......C.................T....CA............A

AF127229 1438 ............................................................

chr18q21.32 1495 GCCTGCTAGCCCATGCTCCGATGTTAATGACATTGAAGGCACCCCTCCCGAGGAAATCTC

chr3p12.3 1497 ..................T..............C.........................G

AF127229 1498 ............................................................

chr18q21.32 1555 AACTGCACAACCCCTACTATGCCCCAATTCAGCGGGAAGCAGTTAGAGCGGTCATCAGCC

chr3p12.3 1557 .................................A............T..A.A......T.

AF127229 1558 ............................................................

chr18q21.32 1615 AACCTCCCCAACAGCACTTGGGTTTTCCTGTTGAGAGGGGGGACTGAGAGACAGGACTAG

chr3p12.3 1617 G..T.................................A...T..................

AF127229 1618 ............................................................

chr18q21.32 1675 CTGGATTTCCTAGGCCAACGAAGAATCCCTAAGCCTAGCTGGGAAGGTGACTGCATCCAC

chr3p12.3 1677 ...................T...............................C........

AF127229 1678 ............................................................

chr18q21.32 1735 CTCTAAACATGGGGCTTGCAACTTAGCTCACACCCGACCAATCAG---------AGAGCT

chr3p12.3 1737 ..T......C................AG...G..-A..A......GTAGTAAAG.C....

AF127229 1738 .............................................---------......

chr18q21.32 1786 CACTAAAATGCTAATTAGGCAAAAATAGGAGGTAAAGAAATAGCCAATCATCTATTGCCT

chr3p12.3 1796 .G.......................C.............................CA...

AF127229 1789 ............................................................

chr18q21.32 1846 GAGAGCACAGCGGGAGGGACAAGGATCGGGATATAAACCCAGGCATTCGAGCCGGCAACG

chr3p12.3 1856 ..C.........A......A..T....A.A.................T..A..AT.....

AF127229 1849 ............................................................

chr18q21.32 1906 GCAACCCCCTTTGGGTCCCCTCCCTTTGTATGGGCGCTCTGTTTTCACTCTATTTCACTC

chr3p12.3 1916 ..T....T......A...................A.............A.....------

AF127229 1909 ............................................................

chr18q21.32 1966 TATTAAATCTTGCAACTGAAAAAAAAAAAAAGAAAA

chr3p12.3 1970 ----..............C.................

AF127229 1969 ...............................A....

* The GenBank accession number as well as the MSRV clone designation according to Komurian-Pradel et al. [1] is indicated. Nucleotide differences between the MSRV sequence and the corresponding HERV-W elements are highlighted in yellow. The AF127229 MSRV sequence can be explained as a recombination between two different HERV-W elements. The region in which the recombination event must have taken place is marked in gray. The A at nucleotide position 2000 of AF 127229 is very likely to be imposed upon the sequence by the primer and was therefore not counted as a nucleotide mismatch.

**AF123882 (87-23)***

chr15q21.3 1 CAGCAGTAGCAGTCTTAGTATCTGAAGCAGTTTAAATAATACAGGGAAGAGATTTTACTG

AF123882 1 .G..........................................................

chr15q21.3 61 TGTGGACATCTCATGATGTGAACGGCATACTGACTGCTAAAGGAGACTTGTGGCTGTCAG

AF123882 61 .............C..............................................

chr15q21.3 121 ACAACCGTTTACTTAAATATCAGGCTCTATTACTTGAAGGGCCAGTGCTGCGACTGCGCA

AF123882 121 ............................................................

chr15q21.3 181 TTTGTGCAACTCTTAACCCAGTCCCATTTCTTCCGGACAATGAAGAAAAGATAGAACATA

AF123882 181 ............................................................

chr15q21.3 241 ACTGTCAACAAGTAATTGCTCAAACCTACACCGCTCGAGGGGGACCCTTTAGAGGTTCCC

AF123882 241 ............................................................

chr15q21.3 301 TTGACTGATCCTGACCTCAACTTGTATACTGATGGAAGTTCCTTTGTAGAAAAAGGACTT

AF123882 301 ............................................................

chr15q21.3 361 CAAAAAGCAGGGTATGCAGTGGTCAGTGATAATGGAATACTTGAAAGTAATCTCCTCACT

AF123882 361 ............................................................

chr15q21.3 421 CCGGGAACTAGTGCTCAGCTGGCAGAACTAATAGTCCTCACTCGGGCACTAGAATTAGGA

AF123882 421 ............................................................

chr15q21.3 481 GAAGGAAAAAGGGTAAATATATATACAGACTCTAAGTATGCTTACCTAGTCCTCCATGCC

AF123882 481 ............................................................

chr15q21.3 541 CATGCAGCAATATGGAGAGAAGGGGAATTCCTAATTTCTGAGGGAACACCTATCAAACAT

AF123882 541 ............................................................

chr15q21.3 601 CAGGAAGCCATTAGGAGATTATTATTCGCTGTACAGCAACCTAAAGAAGTGACAGTCTTA

AF123882 601 ............................................................

chr15q21.3 661 CACTGCCGGGGTCATCAGAAAGGAAAGGAAAGGGAAATAGAAGGGAACCGCCAAGAGGAT

AF123882 661 ............................................................

chr15q21.3 721 ATTGAAGCCAAAAGAGCCGCAAGGCAGGACCCTCCATTAGAAATGCTTATAAAAGGACCC

AF123882 721 ............................................................

chr15q21.3 781 GTAGTATGGGGTAATCCCCTCCGGGAAACCAAGCCCCAGTACTCAGAAGAAGAAATAGAA

AF123882 781 ............................................................

chr15q21.3 841 TGGGGAACCTCACGAGGACATAGTTTCCTCCCCTCAGGATGGCTAGCCACTGAAGAAGGA

AF123882 841 .....................G......................................

chr15q21.3 901 AAAATACTTTTGCTGGCAGCTAACCAATGGAAATTACTTAAAACCCTTCAGCAAACCTTT

AF123882 901 ...........................................................C

chr15q21.3 961 CACTTAGGCATTGATAGCACCCATCAGATAGCCAAATCATTATTTACTGGACCAGGCCTT

AF123882 961 ............................................................

chr15q21.3 1021 TTCAAAACTATCAAGCAGATAGTCAGGGCCTGTGAAGTGTGCCAAAGAAATAATCCCCTG

AF123882 1021 ............................................................

chr15q21.3 1081 CCTTATCGCCAAGCTCCTTCAGGAGAACAAAGAACAGGCAATTACCCAAGAGAAGACTGG

AF123882 1081 ............................................................

chr15q21.3 1141 CAACTAGATTTTATCCACATGCCAAAATCACAGGGATTTCAGTGTCTACTAGTCTGGGTA

AF123882 1141 ............................................................

chr15q21.3 1201 GATACTTTCACTGGTTGGGCAGAGGCCTTCCCCTGTAGGACAGAAAAGTTCCAAGAGGTA

AF123882 1201 ............................................................

chr15q21.3 1261 ATAAAGGCACTAGTTCATGAAGTAATTCCCAGATTCGGACTTCCCTGAGGCTTACAGAGT

AF123882 1261 ............................................................

chr15q21.3 1321 GACAATGGTCCTGCTTTCAAGGCCACAGTAACCCAGGGAGTATCCCAGGCGTTAGGTATA

AF123882 1321 ............................................................

chr15q21.3 1381 GAATATCACTTACACTGCACCTAGAGGCCACAATCCTCAGGGAAGGTTGAGAAAATGAAA

AF123882 1381 ............................................................

chr15q21.3 1441 CACTCAAACGACATCTAAACAAGCTAACCCAGGAAACCCACCTCGCATGGTCTGCTCTGT

AF123882 1441 ............................................................

chr15q21.3 1501 TGTCTATAGCCTTACTAAGAATCCAAAACTCTCCCCAAAAAGCAGGACTTAGCCCATACA

AF123882 1501 ........................................G...................

chr15q21.3 1561 GAATGCTGTATGGACGGTCCTTCCTAACCAATGACCTTCTGCTTGACCAAGAGATGGCCA

AF123882 1561 ............................................................

chr15q21.3 1621 ACTTAGTTGCAGACATCACCTCCTTAGCCAAATATCAACAAGTTCTTAAAACATTACAAG

AF123882 1621 ............................................................

chr15q21.3 1681 GAGCCTGTCCCCGAGAGGAGGGAAAAGAAATATTCCACCCTGGTGTCATGGTATTAGTCA

AF123882 1681 ............................................................

chr15q21.3 1741 AGTCCCTTCCCTCTAATTCCCCATCCCTAGACACATCCTGGGGAGGACCCTACCCAGTCA

AF123882 1741 ............................................................

chr15q21.3 1801 TTTTATCTATCCCAACTGCGGTTAAAGTGGCTGGAGTGGAGTCTTGGATACATCACACTC

AF123882 1801 ............................................................

chr15q21.3 1861 GAATCAAACCCTGGATACTGCCGAAGGAACCCGAAAATCCAGGAGACAACGCTAGCTATT

AF123882 1861 ...........................................G................

chr15q21.3 1921 TCTTTGAACCTCTAGAGGATCTGTGCCTGCTCTTCAAGCAACAACCGTGAGGAAAGTAAC

AF123882 1921 ............................................................

chr15q21.3 1981 TAAAATCGTAAATCCCCATGGCCCTCCCTTATTGTATTTTTCTCTTTACTGTTCTCTCAC

AF123882 1981 ............................................................

chr15q21.3 2041 CACCTTTCAGTCTCACTGCACCCTCTCCATGCCACTGTAGGACCAGTAGCTCCCCTTACC

AF123882 2041 ............................................................

chr15q21.3 2101 AAGCGTTTCTATGGAGAATGCGGCGTCCCAGACATATTGATGCCCCATCGTATAGGAGTT

AF123882 2101 ............................................................

chr15q21.3 2161 TATCTAAGGGAAACCCCGCCTTCACCGCCCACACCCATATGCCCCACAACTGCTATAACT

AF123882 2161 ............................................................

chr15q21.3 2221 CTGCCACTCTTTGCATGCATGCAAATACTCATTATTGGACAGGGAAAATGATTAATCCTA

AF123882 2221 ............................................................

chr15q21.3 2281 GTTGTCCTGGAGGACTTGGAGCCACTGTCTGTTGGACTTAGTTCACCCATACTGGTATGT

AF123882 2281 ............................................................

chr15q21.3 2341 CTGATAGGGGTGGAGTTCAAGATCAGGCAAGAGAAAAACACGTAAAGGAAGTAATCTCCC

AF123882 2341 ............................................................

chr15q21.3 2401 AACTGACCTGAGTACATAGCACGCCTAGCCCCTACAAAGGACTAGATCTCTCAAAACTAC

AF123882 2401 ..................................................C.........

chr15q21.3 2461 ATGAAACCCTCCGTACC

AF123882 2461 .................

* The GenBank accession number as well as the MSRV clone designation according to Komurian-Pradel et al. [1] is indicated. Nucleotide differences between the MSRV sequence and the corresponding HERV-W locus are highlighted in yellow. The primers used for the amplification of the AF123882 clone as reported in Ref. [1] are underlined.

**AF331500 and AF127228 (C15)***

chrXq22.3 1 GAAATAGCCAGACCATTATATACACTAATTAAGGAAACTCAGAAAGCCAATACCATTTAG

AF331500 1 ------------------------------------------------------------

AF127228 1 CC..........................................................

chrXq22.3 61 TAAGATGGGCACCTGAAGCAGAAGCAGCTTTCCAGGCCCTAAAGAAGGCCCTAACCCAAG

AF331500 1 ------------------------------------------------------------

AF127228 61 ............................................................

chrXq22.3 121 CCCCAGTGTTAAGCTTGCCAGTGGGGCAGACTTTTCTTTATATGTCACAGAAAAAAACAG

AF331500 1 ------------------------------------------------------------

AF127228 121 ............................................................

chrXq22.3 181 GAATAGCTCTAGGAGTCCTTACACAGGTCCGAAGGAT--GAGCTTGCAACCCATGGCGTA

AF331500 1 ------------------------------------------------------------

AF127228 181 .....................................--.....................

chrXq22.3 239 C-CTGAGTAAGGAAATTGATGTAGTGGCAAAGGATTGGCCTCATTGTTTATGGGTAGTGG

AF331500 1 ------------------------------------------------------------

AF127228 239 .-..............................A...........................

chrXq22.3 298 CAGCAGTAGGAGTCTTAGTATCTGAAGCAGTTAAAATAATACAGGGA-AGAGATCTTACT

AF331500 1 ------------------------------------------------------------

AF127228 298 ...............................................-............

chrXq22.3 357 GTGTGGAATCTCATG--ATGTGAACGGCATGCTCACTGCTAAAGG---AGACTTGTGA--

AF331500 1 ------------------------------------------------------------

AF127228 357 ...............--............................---..........--

chrXq22.3 410 -CTATCAGACAACTGTGAGGAAAGTAACAAATCA-TAAATCCCCATGGCCCTCCCTTATC

AF331500 1 --------------------------------------------................

AF127228 410 -.................................-.........................

chrXq22.3 468 ATATTTTTCTCTTTACTGTTCTCTTACCCCCTTTCGCTCTCACTGCACCCCCTCCATGCT

AF331500 17 ...C........................................................

AF127228 468 ...C........................................................

chrXq22.3 528 GCTGT--ACAACCAGTAGCTCCCCTTACCAAGAGTTTCTATGAAGAACGCGGCTTCCTGG

AF331500 77 .....--...................................G.................

AF127228 528 .....--.....................................................

chrXq22.3 586 AAATATTGATGCCCCATCATATAGGAGTTTATCTAAGGGAAACTCCACCTTCACTGCCCA

AF331500 135 ............................................................

AF127228 586 ............................................................

chrXq22.3 646 CACCCATATGCCCCGCAACTGCTATAACTCTGCCACTCTTTGCATGCATGCAAATACTCA

AF331500 195 ............................................................

AF127228 646 ............................................................

chrXq22.3 706 TTATTGGACAGGGAAAATGATTAATCCTAGTTGTCCTG---GAGGACTTGGAGCCACTGT

AF331500 255 ......................................---...................

AF127228 706 ......................................---...................

chrXq22.3 763 CTGTTGGACTTACTTCACCCATACCAGTATGTCTGATGGGGGTGGAATTCAAGGTCAGGC

AF331500 312 ............................................................

AF127228 763 ............................................................

chrXq22.3 823 AAGAGAAAAACAAGTAAAGGAAGCAATCTCCCAACTG-ACC---CGGGGACATAGCACCC

AF331500 372 .....................................-...---................

AF127228 823 .....................................-...---................

chrXq22.3 879 CTAGCCCCTACAAAGGACTAGTTCTCTCAAAACTACATGAAACCCTCCGTACCCATACTC

AF331500 428 ............................................................

AF127228 879 ............................................................

chrXq22.3 939 GCCTGGTGAGCCTATTTAATACCACCCTCACTCGGCTCCATGAGGTCTCAGCCCAAAACC

AF331500 488 ............................................................

AF127228 939 ............................................................

chrXq22.3 999 CTACTAACTGT---TGGATGTGCCTCCCCCTGCACTTCAGGCCATACATTTCAATCCCTG

AF331500 548 ...........---..............................................

AF127228 999 ...........---..............................................

chrXq22.3 1056 TTCCTGAACAATGGAACAACTTCAGCACAGAAATAAACACCACTTCCGTTTTAGTAGGAC

chr5p12 1 .A........................C.......G............A............

AF331500 605 ............................................................

AF127228 1056 ............................................................

chrXq22.3 1116 CTCTTGTTTCCAATCTGGAAATAACCCATACCTCAAACCTCACCTGTGTAAAATTTAGCA

chr5p12 61 ..............................T.............................

AF331500 665 ............................................................

AF127228 1116 ............................................................

chrXq22.3 1176 ATACTATAGACACAACCAGCTCCCAATGCATCAGGTGGGTAACACCTCCCACACGAATAG

chr5p12 121 ............A...A.A.......G................TG.........A.....

AF331500 725 ............................................................

AF127228 1176 ............................................................

chrXq22.3 1236 TCTGCCTACCCTCAGGAATATTTTTTGTCTGTGGTACCTCAGCCTATCATTGTTTGAATG

chr5p12 181 ..........T.................................................

AF331500 785 ............................................................

AF127228 1236 ............................................................

chrXq22.3 1296 GCTCTTCAGAATCTATGTGCTTCCTCTCATTCTTAGTGCCCCCTATGACCATCTACACTG

chr5p12 241 A..........AG................................C..............

AF331500 845 ............................................................

AF127228 1296 ............................................................

chrXq22.3 1356 AACAAGATTTATACAATCATGTCGTACCTAAGCCCCACAACAAAAGAGTACCCATTCTTC

chr5p12 301 .................T.....A...........TG.......................

AF331500 905 ............................................................

AF127228 1356 ............................................................

chrXq22.3 1416 CTTTTGTTATCAGAGCAGGAGTGCTAGGCAGACTAGGTACTGGCATTGGCAGTATCACAA

chr5p12 361 ..C.......TG................T.....................G.........

AF331500 965 ............................................................

AF127228 1416 ............................................................

chrXq22.3 1476 CCTCTACTCAGTTCTACTACAAACTATCTCAAGAAATAAATGGTGACATGGAACAGGTCA

chr5p12 421 ...........................T.......C................G.......

AF331500 1025 ............................................................

AF127228 1476 ............................................................

chrXq22.3 1536 CTGACTCCCTGGTCACCTTGCAAGATCAACTTAACTCCCTAGCAGCAGTAGTCCTTCAAA

chr5p12 481 .CA...............A.G...................................T...

AF331500 1085 ............................................................

AF127228 1536 ............................................................

chrXq22.3 1596 ATCGAAGAGCTTTAGACTTGCTAACCGCCAAAAGAGGGGGAACCTGTTTATTTTTAGGAG

chr5p12 541 ..T.......................................................G.

AF331500 1145 ............................................................

AF127228 1596 ............................................................

chrXq22.3 1656 AAGAATGCTGTTATTATGTTAATCAATCCAGAATTGTCACTGAGAAAGTTAAAGAAATTC

chr5p12 601 .............G..............TG....CA.T.....................T

AF331500 1205 .....C......................................................

AF127228 1656 .....C......................................................

chrXq22.3 1716 GAGATCGAATACAATGTAGAGCAGAGGAGCTTCAAAACACCGAACACTGGGGCCTCCTCA

chr5p12 661 ...........T................C...........T.C..C..............

AF331500 1265 .............................................G..............

AF127228 1716 .............................................G..............

chrXq22.3 1776 GCCAATGGATGCCCTGGGTTCTCCCCTTCTTAGGACCTCTAGCAGCTCTAATATTGTTAC

chr5p12 721 .................AC............................A.......T....

AF331500 1325 .................AC............................A.......T....

AF127228 1776 ............................................................

chrXq22.3 1836 TCCTCTTTGGACCCTGTATCTTTAACCTCCTTGTTAAGTTTGTCTCTTCCAGAATTGAAG

chr5p12 781 ..........................T.................................

AF331500 1385 ......................C...T.................................

AF127228 1836 ............................................................

chrXq22.3 1896 CTGTAAAGCTACAAATGGTTCTTCAAATGGAGCCCCAGATGCAGTCCATGACTAAAATCT

chr5p12 841 ................A..............A............................

AF331500 1445 ................A..............A............................

AF127228 1896 .............G.....CT.A........A.....-----------------------

chrXq22.3 1956 ACCACGGACCCCTGGACCAGCCTGCTAGCCCTTGTTCCGATGTTAATGACATCAAAGGCA

chr5p12 901 ...GT.............G.........A.TA..C..T..............TG...T..

AF331500 1505 ...GT.............G.........A.TA..C..T..............TG...T..

AF127228 ------------------------------------------------------------

chrXq22.3 2016 CCCCTCCTGAGGAAATCTCAACTGCACAACCCCTACCATGCCCCATTTCAGCAGGAAGCA

chr5p12 961 .......C............................T.CA.T...A.....T........

AF331500 1565 .......C............................T.CA.T...A.....T........

AF127228 ------------------------------------------------------------

chrXq22.3 2076 GGTAG

chr5p12 1021 .T...

AF331500 1625 .T...

AF127228 -----

* The GenBank accession numbers as well as the MSRV clone designation according to Komurian-Pradel et al. [1] are indicated. Nucleotide differences between the AF331500 [2] and AF127228 MSRV *env* sequence and the best matching HERV-W *env* loci are highlighted in blue and yellow, respectively. The sequences of the primers used for the amplification of the AF127228 clone as reported in Ref. [1] are underlined. Since nucleotide differences that are located within the primer binding region are imposed upon the amplified sequence by the primer they do not represent true nucleotide mismatches and were not counted as such (see Table 3). Note that the MSRV *env* clone AF331500 appears to be a recombined sequence involving two genomic HERV-W *env* loci (Xq22.3 and 5p12). The region in which the recombination event must have taken place is marked in gray. The premature stop codon in the Xq22.3 HERV-W *env* locus that disrupts an otherwise complete HERV-W *env* open reading frame is highlighted in red. The 5p12 HERV-W *env* sequence has a deletion in its 5’ end and is therefore not represented in the 5’ end of the alignment.

MSRV *gag* sequence:

**AF123881 (CL2)***

3q26.32 1 CCTAAGATGTATTCTGGAGAATTGGGACCAATGTGACACTCAGACGCTAAGAAAGAAACG

AF123881 1 ....GA.C....................................................

3q26.32 61 ATTTATATTCTTCTGCAGTACCGCCTGGCCACAATATCCTCTTCAAGGGAGAGAAACCTG

AF123881 61 ............................................................

3q26.32 121 GCTTCCTGAGGGAAGTATAAATTATAACATCATCTTACAGCTAGACCTCTTCTGTAGAAA

AF123881 121 ............................................................

3q26.32 181 GGAGGGCAAATGGAGTGAAGTGCCATATGTGCAAACTTTCTTTTCATTAAGAGACAACTC

AF123881 181 ............................................................

3q26.32 241 ACAATTATGTAAAAAGTGTGGTTTATGCCCTACAGGAAGCCCTCAGAGTCCACCTCCCTA

AF123881 241 ............................................................

3q26.32 301 CCCCAGCGTCCCCTCCCCGACTCCTTCCTCAACTAATAAGGACCCCCCTTTAACCCAAAC

AF123881 301 ............................................................

3q26.32 361 GGTCCAAAAGGAGATAGACAAAGGGGTAAACAATGAACCAAAGAGTGCCAATATTCCCCG

AF123881 361 ............................................................

3q26.32 421 ATTATGCCCCCTCCAAGCAGTGAGAGGAGGAGAATTCGGCCCAGCCAGAGTGCCTGTACC

AF123881 421 ............................................................

3q26.32 481 TTTTTCTCTCTCAGACTTAAAGCAAATTAAAATAGACCTAGGTAAATTCTCAGATAACCC

AF123881 481 ............................................................

3q26.32 541 TGACGGCTATATTGATGTTTTACAAGGGTTAGGACAATCCTTTGATCTGACATGGAGAGA

AF123881 541 ............................................................

3q26.32 601 TATAATGTTACTACTAAATCAGACACTAACCCCAAATGAGAGAAGTGCCGCTGTAACTGC

AF123881 601 ............................................................

3q26.32 661 AGCCCGAGAGTTTGGCGATCTTTGGTATCTCAGTCAGGTCAACAATAGGATGACAACAGA

AF123881 661 ......................................C.....................

3q26.32 721 GGAAAGAACAACTCCCACAGGCCAGCAGGCAGTTCCCAGTGTAGACCCTCATTGGGACAC

AF123881 721 ............................................................

3q26.32 781 AGAATCAGAACATGGAGATTGGTGCCACAAACATTTGCTAACTTGCGTGCTAGAAGGACT

AF123881 781 ............................................................

3q26.32 841 GAGGAAAACTAGGAAGAAGCCTATGAATTACTCAATGATGTCCACTATAACACAGGGAAA

AF123881 841 ............................................................

3q26.32 901 GGAAGAAAATCCTACTGCTTTTCTGGACAGACTAAGGGAGGCATTGAGGAAGCATACCTC

AF123881 901 ...........T................................................

3q26.32 961 CCTGTCACCTGACTCTATTGAAGGCCAACTAATCTTAAAGGATAAGTTTATCACTCAGTC

AF123881 961 ............................................................

3q26.32 1021 AGCTGCAGACATTAGAAAAAACTTCAAAAGTCTGCCTTAGGCCCGGAGCAGAACTTAGAA

AF123881 1021 ............................................................

3q26.32 1081 ACCCTATTTAACTTGGCATCCTCAGTTTTTTATAATAGAGATCAGGAGGAGCAGGCGAAA

AF123881 1081 ............................................................

3q26.32 1141 CGGGACAAACGGGATAAAAAAAAAAGGGGGGGTCCACTACTTTAGTCATGGCCCTCAGGC

AF123881 1141 ............................................................

3q26.32 1201 AAGCAGACTTTGGAGGCTCTGCAAAAGGGAAAAGCTGGGCAAATCAAATGCCTAATAGGG

AF123881 1201 ............................................................

3q26.32 1261 CTGGCTTCCAGTGCGGTCTACAAGGACACTTTAAAAAAGATTATCCAAGTAGAAATAAGC

AF123881 1261 ............................................................

3q26.32 1321 CGCCCCCTTGTCCATGCCCCTTACGTCAAGGGAATCACTGGAAGGCCCACTGCCCCAGGG

AF123881 1321 ............................................................

3q26.32 1381 GATGAAGATACTCTGAGTCAGAAGCCATTAACCAGATGATCCAGCAGCAGGACTGAGGGT

AF123881 1381 ............................................................

3q26.32 1441 GCCCGGGGCGAGCGCCAGCCCATGCCATCACCCTCACAGAGCCCCGGGTATGCTTGACCA

AF123881 1441 ....................................................T.......

3q26.32 1501 TTGAGAGCCAG

AF123881 1501 ..........A

* The GenBank accession number as well as the MSRV clone designation according to Komurian-Pradel et al. [1] is indicated. Nucleotide differences between the AF123881 MSRV *gag* sequence and the corresponding HERV-W *gag* locus are highlighted in yellow. The sequences of the primers used for the amplification of the AF123881 clone as reported in Ref. [1] are underlined. Since nucleotide differences that are located within the primer binding region are imposed upon the amplified sequence by the primer they do not represent true nucleotide mismatches and were not counted as such (see Table 3).

**References**

1. Komurian-Pradel F, Paranhos-Baccala G, Bedin F, Ounanian-Paraz A, Sodoyer M, et al. (1999) Molecular cloning and characterization of MSRV-related sequences associated with retrovirus-like particles. Virology 260: 1-9.

2. Perron H, Jouvin-Marche E, Michel M, Ounanian-Paraz A, Camelo S, et al. (2001) Multiple sclerosis retrovirus particles and recombinant envelope trigger an abnormal immune response in vitro, by inducing polyclonal Vbeta16 T-lymphocyte activation. Virology 287: 321-332.
